# APPLICATION OF TIME-AVERAGED AND INTEGRAL-BASED MEASURE FOR MEASUREMENT RESULTS VARIABILITY REDUCTION IN GSM/DCS/UMTS SYSTEMS

**DOI:** 10.1093/rpd/ncz154

**Published:** 2019-07-12

**Authors:** Darko Šuka, Predrag Pejović, Mirjana Simić-Pejović

**Affiliations:** 1 University of East Sarajevo, Faculty of Electrical Engineering, East Sarajevo, Bosnia and Herzegovina; 2 University of Belgrade, School of Electrical Engineering, Belgrade, Serbia

## Abstract

Since EMF levels from wireless telecommunication networks are non-stationary and exhibit large temporal variations, the use of continuous measurements during extended periods (preferably 24 h or longer) with a data-logging system is required. Because of the short-term variations of E field, the 6-min measurements and 6-min averaged results to obtain the mean level strength at a given place appear to be dependent on the time of measurements during the day. This paper presents a new (integral-based) measure to evaluate electromagnetic exposure. The new measure is a pure physical descriptor of the amount of exposed energy density (a parameter accumulated from instantaneous power density values in time). To confirm previous observations, continuous measurements with personal exposure metre were recorded 24 h a day for two weeks at every location in urban area, 14 different locations in total. Additionally, to check temporal variations and repeatability of exposure assessment, a week of prolonged measurements was taken 6 months later, making in total three weeks of measurements at 2 locations. Day-to-day repeatability of RF-EMF exposure was analysed through the time-averaged and integral-based measure. The analysis is based on approximately 5.1 million data samples (1.7 million for each band). The ratio between the maximum and minimum instantaneous (maximum and minimum 6-min averaged) E field values during the day could reach up to 25 dB (20 dB). Therefore, great variability in the results may occur. By applying the 24 h time-averaged and integral-based measure on a 24 h data set of measurements, the variability of daily exposure could stay within ±20% of the week mean level obtained either with the time-averaged or integral-based measure. Both, the time-averaged E field and integral-based power density exposures of the general public in all locations were found to be well below the general public exposure limits of the ICNIRP guidelines.

## INTRODUCTION

In modern society, there are many sources of electromagnetic (EM) radiation. All of them contribute to increasing of EM interference^(1)^. That leads to an increased amount of absorbed radiation energy, both by the occupational and general public. For this reason, daily assessments of exposure levels to EM fields (EMF), especially from telecommunication and broadcast systems (i.e. mobile phone base stations) in human environments as well as the compatibility of these levels with the appropriate standards, are required. From an exposure point of view to EMF, a great number of standards, recommendations, and measures for the protection of all categories of the population are developed worldwide^(2–6)^. All of them basically prescribe maximum limits of human exposure to non-ionising EMF levels (in terms of internal rates of EM energy deposition, i.e. SAR (Specific Absorption Rate), at radiofrequency (RF) and microwave (MW) frequencies, and of induced electric fields or current densities at lower frequencies up to 10 MHz). Most standards are related to the frequency dependence of the electric field strength and power density of radiation, and exposures are considered with 6-min or 30-min averaging times. Basically, the standards are satisfied when the value averaged over the averaging time is below the maximum value. The maximum value in standards is related to the frequency been measured. In general, the exposure to EMF in everyday life is not a recent occurrence. Human beings have lived with natural radio-electric emissions from the beginning of time, and with artificial radio-electric emissions for more than one century; microwaves and radio-electric emissions are presented in nature from the Sun, galaxies, and our own planet Earth^(7)^. EM spectrum extends from static electric and magnetic fields, domestic electric power frequencies (50/60 Hz) through radio-frequency, infrared, and visible light to gamma-rays^(8)^. Human exposure to naturally occurring sources is negligible compared to that from the artificial ones^(1, 9)^. RF-EMF radiation falls within the non-ionising part of the EM spectrum with a frequency range 3 kHz–300 GHz^(10, 11)^. The fact that RF-EMF is non-ionising does not necessarily mean it may be completely harmless to humans as its interaction with living systems and duration of exposure has been reported to induce the numerous biological effects^(2, 12)^. It has been known for many years that continuous exposure to sufficiently high levels of radio signals can heat biological tissue and potentially cause tissue damage if the human body cannot cope with an extra heat^(8, 13)^. In the range above 100 kHz, the relevant health effect which can be observed is a rise in the body temperature (up to 100 kHz, short-term effects that occur reflect themselves as stimulation of the peripheral nerves and muscles^(2)^). A temperature increase of more than 1°C results from exposure of individuals to a whole-body SAR of 4 W/kg under moderate environmental conditions for about 30 min. Therefore, a whole-body average SAR of 0.4 W/kg (after applying a safety reduction factor of 10) was chosen for the occupational exposure and 0.08 W/kg (an additional factor of 5 making a total of 50) for the general public to prevent the whole-body heat stress^(2, 14)^. The reduction factors for the general public and workers are designed to account for any scientific uncertainties, variations in the population health and environmental conditions (like preheated situations during the exercise, increased thermal environment, humidity, thermal conditions in infants, sensitive people, or even the ingestion of drugs and alcohol^(7)^). Much of the public concern relates to the possibility of health hazards from the long-term exposures at levels too low to produce the measurable heating^(13)^.

Regarding cumulative exposure to non-ionising radiation, the assessment in the multiple source environment, according to the existing standards, requires the calculation of the cumulative exposure (in some standards also called the total exposure ratio), where all the operating frequencies must be considered in a weighted sum (each source is pre-rated according to the limit applicable to its frequency). For compliance with the regulations, the total exposure ratio should be less than 1. Also, much attention in the literature is given to the analysis of multi transmitters (i.e. mobile phones) causing high and periodic short-term exposures (i.e. the doses in the brain and whole body in terms of the time-averaged SAR)^(15, 16)^. Further in the EM spectrum, considering the amount of exposure, the cumulative concept is also well-established in ionising radiation research and occupational epidemiology^(17, 18)^. For the sake of clarity and better understanding the basic idea for the new cumulative measure in non-ionising radiation in this study gained from the cumulative measure in ionising radiation, a brief overview of the second one will be given in the following.

Cumulative exposure, which is the product of intensity and duration, is one of the most commonly used quantitative summary measures of exposure history of a person and can be understood as the integral of intensity or quantity of exposure accumulated over an observed time profile. Cumulative dose is, thus, the total dose resulting from repeated exposures of ionising radiation to an occupationally exposed worker or the general public to the same portion of the body, or the whole body, over a period of time. The unit that describes the cumulative dose in ionising radiation is Sievert. McLean A.R. *et al.*^(19)^. states that ‘the sievert (Sv) is a non-physical derived unit used in the context of radiological protection, which weights the amount of energy deposited in tissue (the absorbed dose measured in grey (Gy), where 1 Gy = 1 Jkg^−1^) by different types of radiation (giving equivalent dose – whole body exposure effects from the external source, in Sv), and the relative sensitivity of tissues irradiated (giving effective dose, in Sv) to probabilistic (stochastic) effects such as cancer induction by low doses or low dose-rates’. Usually, the sievert is not used for high dose-rates of radiation that produce deterministic effects, which is the severity of acute tissue damage that is certain to happen; such effects are compared to the physical quantity absorbed dose measured in grey (Gy). In medicine, for example, the cumulative concept, i.e. the total dose of radiation is given to a patient in a series of radiation treatments^(20–28)^. Computed Tomography (CT) is a major concern to the medical community from the perspective of accumulated radiation dose^(29)^. In Brenner and Hall^(30)^, an estimated 1.5% to 2.0% of all tumours were the attributed CT-associated radiation exposure. While exposure to ionising radiation during individual procedures for patients is typically low and often within existing regulatory limits for acute exposure, the occupational hazards arise from the cumulative effect of exposure over time, both for patients and medical personnel^(31–33)^, where the most of the absorbed dose comes from the photons in the energy range of 10–60 keV^(34)^. Sustained exposure to low-dose radiation from procedures performed daily throughout a career may cause the serious, long-term and possibly fatal adverse health consequences. Low-level, long-term ionising radiation exposure causes stochastic-type health effects that alter the underlying DNA structure, increasing one’s risk of developing cancer, which often materialises 10–20 years post exposure^(35)^. Although controversial, data suggest that the cumulative effect of the fractionated low-dose exposures to ionising radiation has a similar biologic impact to a single acute dose of the same magnitude^(36–38)^. Current thinking is that the stochastic effect occurrence follows a linear no-threshold (LNT) hypothesis. It means that although there is a no-threshold level for these effects, the risk of an effect occurring increases linearly as the dose increases. One sievert carries a 5.5% probability of eventually developing cancer and genetic damage based on the LNT^(39)^. Caution should be exercised in the multiple radiation exposures; however, given that stochastic effects are cumulative. The use of the LNT model is fundamental to the additive system of radiation protection, and it allows the use of effective dose as a surrogate for risk^(40)^. The maximum permissible dose for occupational radiation exposure is 20 mSv per year averaged over 5 years (100 mSv over 5 years) with no more than 50 mSv in a single year. This contrasts sharply with the general public exposure, where 1 mSv per year averaged over 5 years is considered as an acceptable limit^(39)^.

It is beyond the scope of this study to further discuss the various dose-response relationships in ionising radiation that may be found. The purpose of above stated is that there is a justification for the introduction of ‘exposed energy density’ (a parameter accumulated from instantaneous power density values in time) on a daily or week level basis (or even longer periods), expressed in J/m^2^, as a new integral-based (cumulative) measure to characterise the amount of exposure to non-ionising radiation in a long-term sense as a paradigm to cumulative dose concept expressed in sieverts in ionising radiation (used for low dose, long-term exposures). The motivation for the introduction of new measure was to provide a single number more stable against the short-term variations assessing the level of exposure on a specific place under surveillance where the cumulative effects of emissions of single or various sources, with the same frequencies or with different spectra, may occur.

To the best of authors’ knowledge, this kind of approach for quantification of EM radiation, mainly from GSM/DCS/UMTS systems, has not been published yet. It, also, allows making a fair assessment of the measurement results variability reduction and checks compliance with the ICNIRP (International Commission on Non-Ionising Radiation Protection) guidelines for the general public exposure^(2)^.

This paper is structured as follows. At first, a brief overview of the basic standards and current exposure assessment metrics is given, as well as some other related issues, showing the differences with the proposed platform and methodology. After that, the measurements and equipment used in such process are described, as well as a brief characterisation of the EMF exposure induced on the population in some other studies available in the literature. The results and related discussion are depicted in the next section, considering strengths, limitations, and implications for further research. Finally, the last section concludes the paper, highlighting its most relevant results and some aspects that might be of interest for elaborating the existing standards.

## MATERIALS

### EMF exposure assessment metrics

To protect the public from the known health effects of EMFs, there are many documents that provide safety limits for human exposure. Although these documents differ in particulars, most of them have several basic principles in common. According to ITU-T K.52^(41)^ these include the use of basic restrictions and reference levels, the use of two-tier exposure limits (specified for the uncontrolled/general public and controlled/occupational exposure), averaging times, and separate consideration for exposure to low-frequency and high-frequency fields. It is important to emphasise that exposure limits are not emission limits; they apply to locations accessible to workers or members of the general public. ICNIRP guidelines^(2)^ define basic restrictions that limit SAR, expressed in W/kg for RF between 100 kHz and 10 GHz, and characterises the RF human absorption. As the basic quantities are difficult to measure directly, ICNIRP also define the reference levels (reference levels for electric and magnetic fields given by ICNIRP or other institutions were derived for plane wave exposure which indicates a uniform E field distribution in the space occupied by the human without the human body being present) that limit incident field strength to the level inducing an exposure compliant with the basic restrictions. Compliance tests, however, are based on the worst-case assumptions (i.e. the maximum power emitted) and do not represent day-to-day exposure of the population^(42)^. However, the worst-case assessment is adopted by some countries to ensure reliable and reproducible exposure assessments in varying traffic conditions, which guarantees the compliance^(6, 43)^.

According to LEXNET D2.1^(44)^ (Low EMF Exposure Networks), there are different basic external and internal physical parameters or metrics as well as methods used for the assessment of exposure to RF-EMF. These metrics are divided into four categories:

• incident field metrics (such as electric field, magnetic field, and power density – in the far field of antennas),

• exposure ratios (which are the measure for the proportion of the exposure of single wireless communication technology into the total exposure due to frequency dependency of the reference levels in different frequency bands),

• absorption metrics (SAR - the rate of RF energy absorption in the human body), and

• dose metrics (this metric takes the time into account by multiplying the absorption (SAR) or incident field metric with time^(45–47)^).

Further, different methods exist to assess the exposure depending on the aim of assessment. In general, the compliance testing aims at the worst-case exposure assessment^(42, 48)^, while the epidemiological studies focus on the realistic exposure assessment^(49)^. According to Wiart J.^(50)^, ‘the versatile use of wireless communication systems has induced a new challenge with the statistical description of the exposure; even though the FDTD (Finite Difference Time Domain) is a proven computationally efficient technique for modelling problems in bioelectromagnetism, this method is not able to be used to characterise the statistical distribution of the exposure. Conventional numerical techniques such as the Monte Carlo Method, typically used to solve such problems, are not useful in this case since the computation effort needed is inordinately large’.

SAR measurements are the most direct way to demonstrate the compliance, but this is inconvenient in many cases, as they are mainly conducted by the specialised laboratories since the measurements are complex and require special equipment. SAR measurement methodologies make use of the phantoms simulating the human head or body which have been developed to provide the conservative results concerning the exposure in the real humans^(51)^. In the literature, numerical SAR assessments have been reported in a number of studies using various techniques^(52–57)^. Results from numerical SAR simulations, evaluated against the basic restrictions, have been compared with power density results, evaluated concerning the reference levels^(58–64)^. Compliance assessments based on SAR are in general required for RF sources used close to the body^(65–67)^. The physical significance of the SAR concept is that it is the source of heating. This may be of more significance in the mobile phone case, but less significant in the base station case.

Further, limits are usually expressed as root mean square (RMS) values of a continuous wave averaged over a defined time period. For example, ICNIRP reference limits^(2)^ (i.e. E field) are to be averaged over any 6-min time below 10 GHz and over a 68/f^1.05^-minute time (f is the frequency in GHz) for frequencies exceeding 10 GHz (ranging downwards from 6-min to 10 s at 300 GHz where the limits meet up with exposure limits for infrared energy), and it should be continuous in effect (using a rectangular window), in order to avoid losing the data^(68, 69)^. Therefore, for the strongly time-dependent signals, an elaboration of the measurement results (post-processing procedure) may be necessary to be compared with the limit^(69)^. However, according to IEC 62232^(67)^ (International Electrotechnical Commission), for evaluation of the strength of EMF around base stations, it is prohibited to perform time-averaging when evaluating compliance (when the theoretical maximum of E field is to be calculated).

According to Foster *et al.*^(70)^. ‘averaging times in present limits have generally been set on the basis of ad hoc approximations and back-of-the-envelope calculations.’ Choosing an appropriate averaging time in standards is important in the design of exposure limits to protect against thermal hazards (6-min averaging is more highlighted and based on the heating phenomena (i.e. SAR measurements), not by the time variation of the radio communication signals). Chosen averaging times reflects the fact that it takes time for the body temperature to rise the during exposure to RF-EMF (time-averaging adds a safety margin in most cases). The 6-min time in ICNIRP is also expanded for the EMF measurements to obtain a mean level strength at a given place. But, as it will be presented later, ‘any’ 6-min averaged results and measurements during the day may be very different and time-dependent. The variations of E field levels during the day are rather high, depending on several factors. To overcome that, the variability of the measurement results has to be reduced.

## METHODS

### Factors influencing temporal variations

Assessment of EMF levels from RF emission sources, especially in urban environments, may not be an easy task. Some of the scatterers involved in multipath propagation may also vary their position or their scattering characteristics with time, producing additional temporal variations on the total EMF at a given location. Therefore, the short-term variations are stochastic and they depend on several factors:^(43, 71–75)^ transmitted power, antenna radiation pattern, traffic activity, discontinuous transmissions, dynamic power control, modulation, frequency, etc. As a result, EMF levels are not stationary, but very randomly in time^(6, 71, 76–80)^. For example, variations of BCCH (Broadcast Control Channel) over time, due to environmental changes (e.g. movement of the people, the weather) and the fact that the specifications for the stability of the BCCH are not strict, might be typically 4–8 dB^(78, 81)^.

The main problem that stands is how to characterise and reduce the variations and increase the repeatability of measurements. Under these conditions, a single short-term measurement of 6-min might not be sufficient enough to properly check whether or not the safety limits are met in exact proportion^(82)^. The measurement results can be both under- and over-estimated depending on whether the extreme value (which might either be a value of minimum or maximum) is caught during the measurement time. However, the 6-min measurement and averaged results will still be valid for testing the compliance with safety regulations^(6, 41, 69, 83, 84)^.

### Measurement equipment and procedure

To obtain the results of temporal variations of E field levels, our measurement campaign was conducted in densely populated urban areas during nine months (January to September 2016). The measurements were recorded 24 h a day, for 2 weeks at every location, 14 different locations in total. Additionally, at first 2 locations (referred as L1 and L2), a week of prolonged measurements was taken 6 months later (from August 8−14 and August 15−21 2016, respectively), making in total 3 weeks of measurements at those locations, in order to additionally capture the temporal variations and check repeatability of the exposure assessment. All locations were of the indoor type and in the far field region.

The measurements consisted of the following. Before each continuous measurement with personal exposure metre (PEM) EME Spy 140 (SATIMO, EMF Measurement & Simulation Tools, Brest, France), the maximum total E field for all observed relevant sources at particular measurement location was determined by sweeping the area. For that purpose, we used the measuring system that consisted of triaxial R&S TS-EMF-B1 measuring probe (type 1158.9295K03-100097) in the form of an isotropic radiator (dynamic range 1 mVm^−1^–100 Vm^−1^ specifically designed to measure electric field strength in the frequency range from 30 MHz to 3 GHz) in the combination with the spectrum analyzer R&S FSH6 (type 1145.5850.26-102096, frequency range from 100 kHz to 6 GHz). The uncertainty of measurement for the electric field and the considered setup is ± 3 dB (−29% to 41%) for normal distribution^(6)^. This uncertainty represents the expanded uncertainty evaluated using a confidence interval of 95%. Settings to check the compliance of different signals with the ICNIRP guidelines were in agreement with those proposed by Verloock *et al.*^(85)^, and Joseph *et al.*^(86)^. It should be noted that these measurements do not replace the continuous ones, but they complement them (time period between such measurements may vary, being limited by the need of a human presence in the area^(87)^). Further, after determining the maximum EMF level in the room, continuous measurement with the PEM was initiated. However, we could not obey to place the PEM at some places of the maximum found in the first step due to indoor room facilities (the height was lower or higher than 1.5 m). Then, similar to the procedure described in Vermeeren *et al.*^(73)^, and Markakis *et al.*^(88)^, the PEM was placed at an available position in the rooms and was standing alone. That way, we wanted to avoid the influence due to shielding of the body that may occur, like when the PEMs are carried on the body (underestimations in such cases might be up to 6.5 dB^(89–93)^). The measurement cycle of the PEM was configured to be 10 s to collect a large number of data samples, generating the robust data sets. Therefore, each of these measurements during a day resulted in 8640 samples per observed band. During the whole measurement period, each location was secured, and only authorised technical personnel had access to such places to provide the measurement conditions of ‘unperturbed field’^(94, 95)^. Further, all measurements were made within the range of temperature and humidity stated by the manufacturer of all equipment used. In addition, the distances between the signal sources and receiving positions were not considered; we concentrated on the general exposure assessment. Finally, the conducted study (based on a repetitive data collection) demonstrated that the measurements were reproducible for the base station exposure, despite the high temporal variations. The issue of reproducibility of the measurements is of crucial importance to have the reliable measurement results enabling that way a consistent comparison over time.

### Exposure metres measurement issues

Characterising RF-EMF exposure with the PEMs has shown to be feasible for quantifying exposure levels and investigating the temporal trends (which may be highly influenced by daily or weekly recurring events). The PEMs allow collecting large amounts of data with the little effort and may be used in a variety of different indoor/outdoor environments. They, also, allow differentiating between the different sources, including all relevant telecommunication signals such as GSM900 (GSM), GSM1800 (DCS) and UMTS2100 (UMTS). It is worth highlighting that the PEM, although able to measure the aforementioned bands, cannot distinguish the contribution of various technologies within the same band. For instance, in the GSM downlink band, it cannot separate the E field caused by the GSM and UMTS technologies (band VIII) but will provide the overall value.

So far, the PEMs have been widely used in various studies^(49, 77, 79, 89–91, 96–115)^. These studies found that RF-EMF levels in the everyday environment are far below the regulatory limits. Despite easier applicability of the PEMs, the limitations still exist. Changing the batteries of the PEMs can cause incorrect fluctuations of the E field. Further, the lower detection limit of EME Spy 140 differs from band to band. This might lead to overestimation of signal levels in some frequency bands, as the real signal might be much lower than the threshold level for the observed band. Another problem is that these devices have significant crosstalk (power that is emitted in a certain band and registered in another), which perturbs the data recorded by the PEMs^(116)^. The uncertainty of the measurement accuracy of such portable devices due to shadowing of the body and variability of their position on the body has been investigated before^(91, 93, 117–121)^. Against the limitations of the PEMs stated above, the employed EME Spy 140, regarding the sensitivity, has a higher sensitivity range at the lower detection limit of 0.000067 to upper detection limit of 66.3 mW/m^2^ (E field between 0.005 and 5 V/m), resulting in few non-detects (censored values) in the DCS and UMTS downlink bands (considering the measurements conducted in this study). Therefore, we did not impute the measurement values below the detection limit, as previously done by others^(49, 79, 90, 107, 114, 115, 122)^. According to the procedure proposed by Röösli *et al.*^(103)^, the ROS (Regression on Order Statistics) method is used when a large proportion (following normal distribution or lognormal, if logs are used) of the measurement data is censored, i.e. below the lower detection limit, to produce the more reliable results when calculating the mean values from the PEM measurements. In addition to ROS and according to Bhatt *et al.*^(123)^, it should be noted that, there are other approaches to deal with the censored data: the substitution methods (by LOD, LOD/2 and LOD/ √2^(124, 125)^), maximum likelihood estimation (MLE) methods, and Kaplan–Meier methods^(125)^. Due to the relatively high sensitivity of the device probe, the number of non-detects in this study was very limited. For GSM, none of the data was below the detection limit and for DCS and UMTS, 0.7% and 0.5%, respectively (considering all locations). Additionally, DCS band was not present at 6 and UMTS at 2 locations. Considering the previously stated, if the field strength was lower or higher than the measurement range of the PEM, the measurement value stored in the PEM memory was the value of the respective (lower or higher) detection limit. Values below the lower detection limit are of crucial importance, as the major part of the data in everyday situations might be below that limit. At the upper detection limit, the question arises on how frequently high E field values occur and whether they fail to be recorded due to the sampling rate of the PEM. During the measurement campaign in this study, the telecommunication networks were not forced to operate in any specific mode to observe the temporal variations of E field.

## RESULTS AND DISCUSSION

### Studies and aspects of temporal trends in the literature

In the literature, some interesting comparisons of exposures from the different sources (in a unit of V/m as the most used quantity for the EMF exposure assessment) in time were made. According to Neubauer *et al.*^(99)^, a 24 h exposure from a base station (1–2 V/m) corresponded to about 30 min of mobile phone use. Similar, according to Regel *et al.*^(126)^, the local brain’s exposure from the first 4 s of a phone call corresponded to the cumulative exposure over the 24 h at 1 V/m incident field on 2150 MHz from a base station. So far, different studies analysed the temporal variations during shorter time periods, like 24 hours^(43, 49, 72, 127, 128)^, or several days^(71, 73, 78)^, and up to several weeks^(129)^.

Long-Term monitoring of the temporal variations is needed to identify high exposure areas and to anticipate critical development of RF-EMF exposure at public places. For such purposes, the monitoring systems have been firstly implemented in various cities in Europe, such as in Greece^(87)^, Italy^(130)^ and Portugal^(131)^. Newer solutions and equipment for the monitoring issues are implemented in Hungary (NNMH), Romania (ANCOM), Serbia (RATEL), Spain (SmartSantander), and elsewhere (more details in the Acknowledgement). Additional information (links) to the official websites for some other EMF monitoring networks are given in Appendix 1 of Recommendation ITU-T K.83-A1^(132)^. Recommendation ITU-T K.83^(68)^ gives the guidance on how to make the long-term measurements for the monitoring of EMF in the selected areas that are under the general public concern. It also specifies the methods and characteristics of the monitoring system to be used for continuous monitoring of the EMFs, both in the broadband and frequency selective measurement systems, in the band of 9 kHz-300 GHz.

### Interpretation

**Figure 1.**
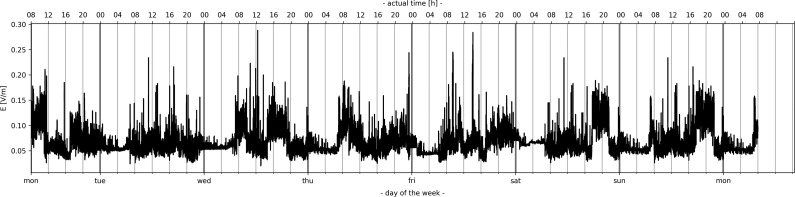
Temporal variations of E field strength for GSM downlink band over the first week.

**Figure 2.**
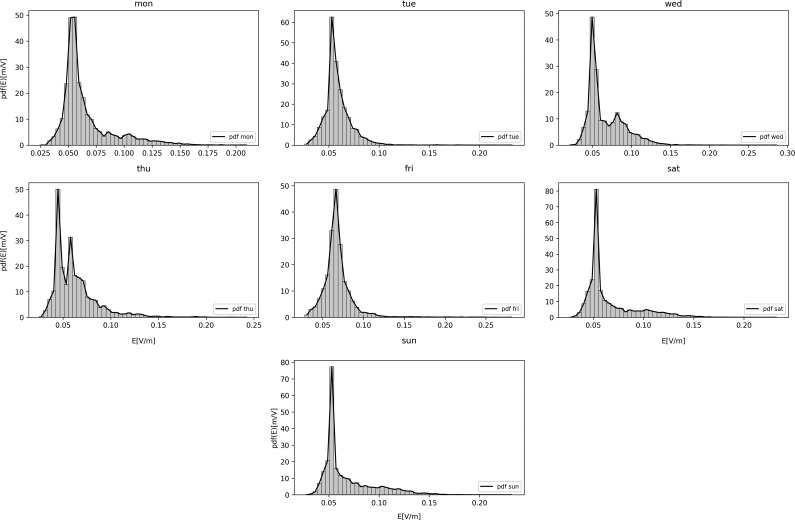
Probability density functions (PDFs) of the instantaneous E field for GSM downlink band for days of the first week.

**Figure 3.**
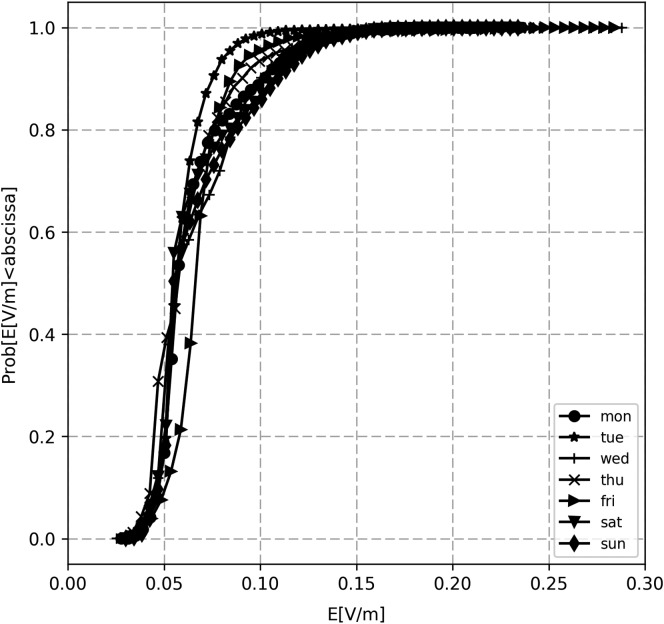
Cumulative distribution functions (CDFs) of the instantaneous E field for GSM downlink band for days of the first week.

**Figure 4.**
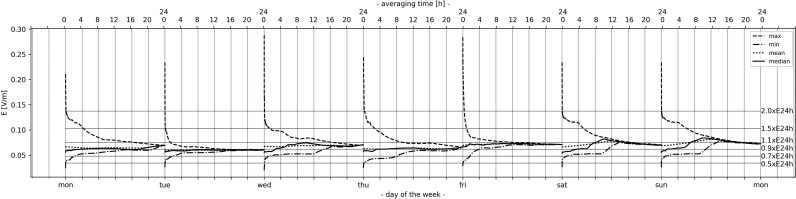
Time-averaged E field strength for GSM downlink band with variable averaging time (10 s to 24 h, time-step: 10 s) over the first week.

**Figure 5.**
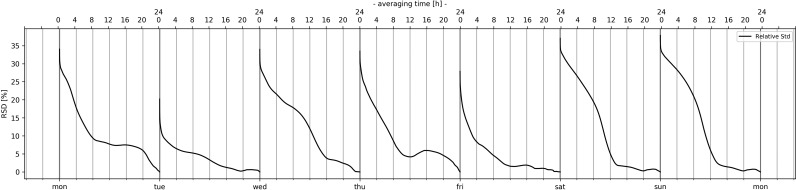
Time-averaged relative standard deviation for GSM downlink band with variable averaging time (10 s to 24 h, time-step: 10 s) over the first week.

**Figure 6.**
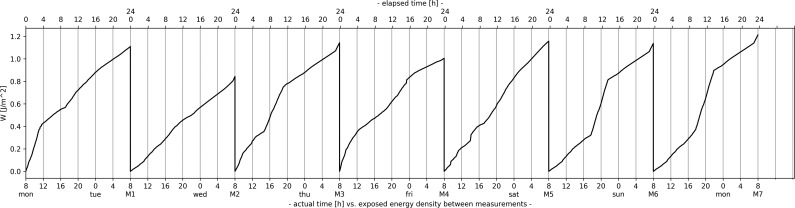
Integral-based (cumulative) measure of instantaneous power density values for GSM downlink band over the first week.

**Figure 7.**
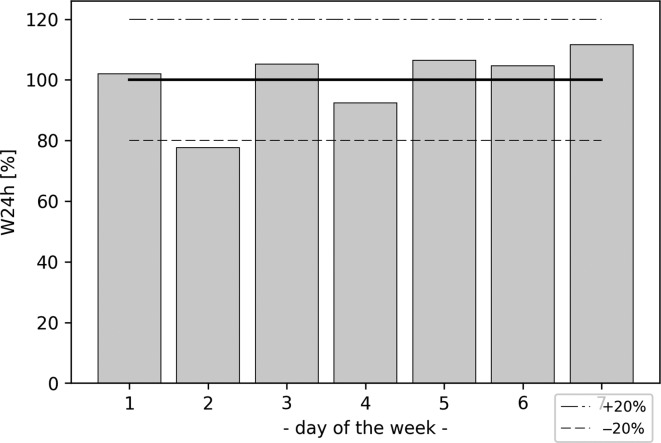
Exposed energy density normalised to first week mean level of exposed energy density (W_24hmean_week_) for GSM downlink band.

For the sake of clarity, in this paper, the authors decided to present the results at Location 1 (L1) for all three downlink frequency bands (Tables 1–8) and to illustrate graphically only the GSM band, which exists in most environments (Figures 1–7). Additional figures for the first week (DCS and UMTS), and full results (figures and tables) for the second and third week (all three bands), are given in an online supplementary (see Supplementary material), in the same order as discussed here (only the band names are changed in figure and table captions). Even though the measurement results in this study are given for one location only, the discussions and conclusions are based on the behaviour observed at all 14 locations.

**Table 1.** Summary statistic on instantaneous electric field parameters (V/m), first week.

Service	Day	E_min_	E_max_	E_mean_	p_50_	p_95_	σ	ICNIRP	ER^2^ (%)	AC^3^ (%)
GSM (900 MHz)	1	0.024	0.211	0.065	0.056	0.118	0.023	41.3^1^	0.511	57.779
	2	0.026	0.234	0.059	0.056	0.083	0.014		0.567	51.476
	3	0.020	0.288	0.067	0.055	0.113	0.023		0.697	58.676
	4	0.025	0.244	0.062	0.057	0.108	0.023		0.591	49.464
	5	0.028	0.284	0.068	0.066	0.096	0.019		0.688	58.127
	6	0.026	0.234	0.066	0.054	0.122	0.025		0.567	60.902
	7	0.026	0.234	0.068	0.055	0.123	0.026		0.567	55.572
DCS (1800 MHz)	1	0.008	0.090	0.031	0.029	0.044	0.007	58.3^1^	0.154	12.238
	2	0.008	0.092	0.031	0.029	0.049	0.011		0.158	15.138
	3	0.006	0.089	0.028	0.024	0.050	0.012		0.153	10.873
	4	0.006	0.093	0.027	0.029	0.043	0.009		0.160	9.118
	5	0.006	0.088	0.028	0.030	0.042	0.010		0.151	10.320
	6	0.009	0.090	0.029	0.027	0.044	0.009		0.154	10.979
	7	0.009	0.090	0.029	0.027	0.048	0.011		0.154	10.101
UMTS (2100 MHz)	1	0.022	0.156	0.048	0.043	0.077	0.015	61^1^	0.256	29.983
	2	0.021	0.179	0.047	0.043	0.073	0.015		0.293	33.386
	3	0.026	0.148	0.049	0.047	0.074	0.013		0.243	30.451
	4	0.021	0.179	0.057	0.058	0.092	0.020		0.293	41.417
	5	0.022	0.148	0.051	0.047	0.076	0.013		0.243	31.553
	6	0.028	0.106	0.047	0.045	0.068	0.011		0.174	28.118
	7	0.024	0.129	0.055	0.053	0.085	0.014		0.211	34.327

^1^Regulatory exposure limit for cumulative RF-EMF exposure.

^2^Exposure Ratio = maximum field value/ICNIRP reference level.

^3^Average Contribution at L1.

**Table 2.** The ratio of electric field strength (in dB) due to averaging for GSM downlink band, first week.

Service		Day	10 s/30 s	10 s/1 m	10 s/3 m	10 s/6 m	10 s/15 m	10 s/30 m	10 s/1 h	10 s/10 h	10 s/24 h
GSM (900 Mhz)	E_max_10s_/E_max_i_	1	0.727	1.319	2.734	3.830	4.066	4.448	4.850	8.373	9.644
		2	1.517	2.732	5.521	6.851	7.613	8.112	9.441	11.164	11.731
		3	2.209	4.487	5.551	7.290	7.805	8.252	8.736	10.753	12.214
		4	0.432	1.197	1.774	2.810	5.044	5.497	6.486	10.297	11.335
		5	1.189	1.601	2.409	3.061	5.179	7.279	9.028	11.409	12.040
		6	1.517	2.732	4.657	4.915	5.286	5.523	5.735	8.758	10.434
		7	1.517	2.732	4.657	4.915	5.286	5.523	5.735	8.329	10.154
	E_min_10s_/E_min_i_	1	−2.941	−3.072	−3.615	−4.015	−4.256	−4.315	−4.629	−7.517	−9.238
		2	−1.160	−1.688	−2.679	−3.072	−3.999	−4.204	−4.715	−6.536	−7.354
		3	−1.263	−2.000	−2.660	−5.012	−6.611	−6.940	−7.579	−8.360	−10.954
		4	−1.488	−1.786	−2.177	−2.559	−3.078	−3.246	−3.800	−6.803	−8.454
		5	−0.622	−1.242	−1.861	−2.310	−2.495	−2.890	−3.726	−7.682	−8.083
		6	−1.346	−1.922	−3.302	−3.833	−4.632	−4.797	−5.225	−6.570	−8.651
		7	−2.377	−3.075	−3.693	−4.041	−4.632	−4.797	−5.373	−6.765	−8.931
	E_mean_10s_/E_mean_i_	1	−0.059	−0.080	−0.103	−0.115	−0.124	−0.122	−0.095	0.129	−0.522
		2	−0.061	−0.085	−0.107	−0.117	−0.123	−0.111	−0.102	−0.139	−0.234
		3	−0.056	−0.078	−0.104	−0.117	−0.124	−0.118	−0.086	−0.303	−0.503
		4	−0.051	−0.072	−0.099	−0.112	−0.126	−0.111	−0.069	−0.254	−0.560
		5	−0.042	−0.061	−0.089	−0.110	−0.154	−0.205	−0.261	−0.528	−0.328
		6	−0.050	−0.070	−0.092	−0.105	−0.122	−0.135	−0.153	−1.306	−0.609
		7	−0.054	−0.076	−0.100	−0.114	−0.136	−0.150	−0.167	−1.377	−0.594
	E_median_10s_/E_median_i_	1	−0.268	−0.323	−0.364	−0.410	−0.442	−0.436	−0.480	−1.100	−1.878
		2	−0.155	−0.160	−0.183	−0.167	−0.155	−0.174	−0.292	−0.686	−0.690
		3	−0.223	−0.287	−0.314	−0.318	−0.269	−0.245	−0.335	−2.628	−2.167
		4	−0.152	−0.165	−0.230	−0.266	−0.288	−0.278	−0.259	−1.029	−1.296
		5	−0.044	−0.028	−0.044	−0.044	−0.061	−0.079	−0.249	−0.890	−0.635
		6	−0.056	−0.118	−0.175	−0.299	−0.308	−0.396	−0.511	−3.608	−2.303
		7	−0.272	−0.353	−0.408	−0.415	−0.430	−0.602	−0.572	−3.704	−2.423

i – the size of running average.

**Table 3.** The ratio of electric field strength (in dB) due to averaging for DCS downlink band, first week.

Service		Day	10s/30 s	10 s/1 m	10 s/3 m	10 s/6 m	10 s/15 m	10 s/30 m	10 s/1 h	10 s/10 h	10 s/24 h
DCS (1800 MHz)	E_max_10s_/E_max_i_	1	0.452	0.846	2.353	4.326	5.642	5.747	5.946	7.817	8.983
		2	2.964	3.709	4.640	5.026	5.200	5.398	5.767	6.882	8.937
		3	1.605	2.243	2.582	3.124	3.602	3.824	4.354	7.945	9.334
		4	2.346	2.736	4.436	5.125	5.677	6.076	6.113	9.208	10.301
		5	0.953	1.579	1.973	2.157	2.591	3.644	5.356	8.557	9.371
		6	0.452	0.846	2.353	4.326	5.642	5.747	5.946	7.817	9.574
		7	1.792	2.408	2.919	3.456	4.014	4.438	4.799	7.449	9.259
	E_min_10s_/E_min_i_	1	−2.232	−2.902	−3.739	−4.234	−5.209	−5.325	−5.782	−10.843	−12.040
		2	−1.350	−2.299	−3.261	−3.615	−4.419	−5.096	−6.425	−9.285	−12.277
		3	0.000	0.000	−1.019	−6.798	−8.395	−9.096	−9.713	−13.006	−15.674
		4	−0.937	−1.761	−2.170	−2.728	−3.954	−4.599	−6.240	−10.821	−13.506
		5	0.000	−0.645	−2.041	−2.942	−5.150	−6.756	−7.295	−13.676	−15.539
		6	−0.327	−1.276	−2.238	−4.505	−5.921	−6.230	−6.503	−7.901	−10.426
		7	−0.327	−1.276	−2.238	−4.505	−5.921	−6.230	−6.503	−8.262	−10.741
	E_mean_10s_/E_mean_i_	1	−0.032	−0.045	−0.062	−0.074	−0.092	−0.104	−0.131	0.063	−0.241
		2	−0.033	−0.046	−0.063	−0.075	−0.095	−0.120	−0.182	−1.069	−0.531
		3	−0.049	−0.068	−0.090	−0.097	−0.096	−0.083	−0.062	−0.802	−0.685
		4	−0.042	−0.060	−0.079	−0.086	−0.084	−0.062	−0.006	0.172	−0.468
		5	−0.071	−0.100	−0.131	−0.149	−0.181	−0.224	−0.285	−0.328	−0.477
		6	−0.035	−0.048	−0.061	−0.066	−0.063	−0.038	0.006	0.196	−0.403
		7	−0.054	−0.074	−0.094	−0.104	−0.112	−0.114	−0.105	−1.280	−0.534
	E_median_10s_/E_median_i_	1	−0.199	−0.199	−0.202	−0.241	−0.308	−0.326	−0.304	−0.308	−0.854
		2	0.000	−0.005	−0.053	−0.123	−0.170	−0.193	−0.254	−1.992	−1.091
		3	−0.015	−0.081	−0.056	−0.044	−0.232	−0.148	−0.235	−2.936	−2.049
		4	0.194	0.245	0.264	0.195	0.414	0.612	0.810	0.662	0.179
		5	−0.210	−0.285	−0.289	−0.331	−0.341	−0.288	−0.444	0.110	0.024
		6	−0.243	−0.301	−0.321	−0.334	−0.342	−0.414	−0.264	−0.163	−0.883
		7	−0.150	−0.227	−0.336	−0.371	−0.404	−0.419	−0.340	−2.316	−1.198

i – the size of running average

**Table 4.** The ratio of electric field strength (in dB) due to averaging for UMTS downlink band, first week.

Service		Day	10s/30 s	10 s/1 m	10 s/3 m	10 s/6 m	10 s/15 m	10 s/30 m	10 s/1 h	10 s/10 h	10 s/24 h
UMTS (2100 MHz)	E_max_10s_/E_max_i_	1	0.990	1.192	3.670	4.956	5.243	5.551	5.733	8.729	9.869
		2	2.312	2.663	3.950	4.396	4.641	5.236	7.315	10.257	11.285
		3	1.892	2.184	2.697	3.490	4.733	5.072	5.438	7.966	9.279
		4	2.312	2.663	3.950	4.396	4.641	5.236	7.315	7.939	9.416
		5	1.892	2.184	2.697	3.490	4.733	5.072	5.438	7.767	9.033
		6	0.963	1.829	2.367	2.829	3.174	3.343	4.161	5.939	6.912
		7	0.484	0.638	2.021	3.091	3.696	3.895	4.235	6.002	7.073
	E_min_10s_/E_min_i_	1	−0.906	−2.462	−3.168	−4.091	−4.182	−4.253	−4.367	−5.599	−7.145
		2	−0.855	−2.602	−3.355	−3.425	−3.468	−3.536	−3.636	−5.407	−7.328
		3	−0.332	−1.603	−2.221	−2.365	−2.509	−2.562	−2.614	−3.925	−5.826
		4	−2.816	−3.243	−3.355	−3.425	−3.468	−3.536	−3.636	−6.559	−9.196
		5	−1.783	−3.054	−4.497	−4.642	−4.762	−4.810	−4.882	−5.891	−7.524
		6	−1.340	−1.473	−1.577	−1.722	−1.865	−1.919	−1.970	−3.281	−4.651
		7	−1.447	−1.852	−2.385	−2.447	−2.656	−3.054	−3.766	−6.633	−7.535
	E_mean_10s_/E_mean_i_	1	−0.057	−0.076	−0.096	−0.104	−0.103	−0.085	−0.043	0.119	−0.390
		2	−0.053	−0.069	−0.079	−0.076	−0.050	0.002	0.064	0.223	−0.422
		3	−0.051	−0.069	−0.088	−0.097	−0.109	−0.119	−0.138	−0.598	−0.312
		4	−0.061	−0.080	−0.094	−0.096	−0.086	−0.063	−0.053	−0.777	−0.486
		5	−0.051	−0.069	−0.088	−0.098	−0.112	−0.129	−0.160	−0.630	−0.273
		6	−0.051	−0.068	−0.083	−0.089	−0.093	−0.090	−0.081	−0.547	−0.233
		7	−0.063	−0.086	−0.107	−0.118	−0.132	−0.142	−0.145	−0.584	−0.284
	E_median_10s_/E_median_i_	1	−0.264	−0.323	−0.434	−0.459	−0.497	−0.502	−0.490	−0.777	−1.324
		2	−0.231	−0.312	−0.385	−0.466	−0.564	−0.537	−0.621	−0.456	−1.103
		3	−0.246	−0.363	−0.509	−0.523	−0.558	−0.544	−0.746	−1.223	−0.684
		4	−0.355	−0.515	−0.707	−0.783	−0.866	−1.056	−1.110	−1.267	−0.372
		5	−0.281	−0.399	−0.535	−0.590	−0.615	−0.626	−0.900	−1.533	−0.931
		6	−0.259	−0.376	−0.478	−0.518	−0.591	−0.659	−0.596	−1.124	−0.530
		7	−0.109	−0.214	−0.341	−0.375	−0.385	−0.497	−0.555	−0.928	−0.653

i – the size of running average.

**Table 5.** Maximum to minimum ratio of electric field strength (in dB) due to averaging for GSM/DCS/UMTS downlink band, first week.

Service		Day	10 s	30 s	1 min	3 min	6 min	15 min	30 min	1 h	10 h
GSM (900 MHz)	E_max_i/_E_min_i_	1	18.881	15.214	14.491	12.532	11.036	10.56	10.119	9.402	2.991
		2	19.085	16.407	14.664	10.884	9.162	7.474	6.769	4.929	1.384
		3	23.167	19.696	16.680	14.956	10.865	8.751	7.976	6.852	4.054
		4	19.789	17.869	16.806	15.838	14.420	11.667	11.046	9.503	2.689
		5	20.123	18.313	17.281	15.853	14.752	12.449	9.955	7.369	1.032
		6	19.085	16.221	14.431	11.126	10.337	9.167	8.765	8.125	3.756
		7	19.085	15.19	13.277	10.735	10.129	9.167	8.765	7.977	3.991
DCS (1800 MHz)	E_max_i/_E_min_i_	1	21.023	18.339	17.275	14.930	12.463	10.172	9.951	9.295	2.363
		2	21.214	16.90	15.206	13.313	12.574	11.595	10.719	9.022	5.047
		3	25.008	23.404	22.766	21.408	15.086	13.012	12.089	10.941	4.057
		4	23.807	20.524	19.310	17.201	15.954	14.175	13.131	11.454	3.778
		5	24.91	23.957	22.687	20.896	19.811	17.169	14.510	12.26	2.677
		6	20.00	19.221	17.878	15.408	11.169	8.436	8.023	7.550	4.282
		7	20.00	17.881	16.316	14.843	12.040	10.064	9.332	8.698	4.289
UMTS (2100 MHz)	E_max_i/_E_min_i_	1	17.014	15.118	13.360	10.176	7.967	7.589	7.210	6.915	2.687
		2	18.613	15.446	13.348	11.308	10.792	10.504	9.841	7.662	2.949
		3	15.106	12.882	11.318	10.188	9.250	7.864	7.472	7.054	3.215
		4	18.613	13.486	12.707	11.308	10.792	10.504	9.841	7.662	4.115
		5	16.557	12.882	11.318	9.363	8.425	7.062	6.675	6.237	2.898
		6	11.563	9.260	8.261	7.619	7.012	6.523	6.301	5.432	2.343
		7	14.608	12.677	12.117	10.202	9.070	8.256	7.659	6.607	1.973

i – the size of running average.

**Table 6.** The ratio of the standard deviation (in dB) due to averaging for GSM/DCS/UMTS downlink band, first week.

Service		Day	10 s/30 s	10 s/1 m	10 s/3 min	10 s/6 m	10 s/15 m	10 s/30 m	10 s/1 h	10 s/10 h
GSM (900 MHz)	σ__10 s_/σ__i_	1	0.495	0.694	0.929	1.114	1.411	1.631	2.042	12.184
		2	1.295	1.972	2.873	3.448	4.367	5.478	6.589	12.949
		3	0.487	0.710	1.006	1.194	1.458	1.719	2.102	6.691
		4	0.403	0.583	0.843	1.037	1.428	1.997	2.704	16.375
		5	0.572	0.864	1.295	1.639	2.406	3.386	4.755	20.271
		6	0.351	0.502	0.682	0.793	0.942	1.098	1.386	9.057
		7	0.393	0.56	0.757	0.881	1.060	1.209	1.432	8.699
DCS (1800 MHz)	σ__10 s_/σ__i_	1	0.603	0.884	1.260	1.553	2.077	2.566	3.440	9.905
		2	0.256	0.362	0.487	0.562	0.677	0.802	1.065	6.259
		3	0.305	0.444	0.640	0.759	0.953	1.192	1.681	7.849
		4	0.399	0.581	0.834	0.995	1.240	1.559	2.134	8.494
		5	0.656	0.960	1.310	1.522	1.895	2.359	3.124	11.477
		6	0.386	0.554	0.759	0.913	1.159	1.361	1.644	6.317
		7	0.440	0.619	0.815	0.928	1.064	1.170	1.301	7.541
UMTS (2100 MHz)	σ__10s_/σ__i_	1	0.679	0.951	1.280	1.467	1.713	1.908	2.246	12.205
		2	0.577	0.802	1.081	1.280	1.742	2.458	3.043	10.43
		3	0.742	1.045	1.374	1.532	1.713	1.867	2.083	6.690
		4	0.560	0.761	0.974	1.092	1.299	1.564	1.734	7.419
		5	0.865	1.220	1.605	1.795	2.029	2.234	2.533	8.186
		6	1.045	1.464	1.915	2.097	2.280	2.430	2.681	8.986
		7	1.047	1.503	1.985	2.203	2.455	2.630	2.887	12.145

i – the size of running average.

**Table 7.** Time-averaged mean E field (at 24 h), first week.

Service	GSM	DCS	UMTS
day of the week	E_24hmean_ (V/m)	E_24hmean_ (V/m)	E_24hmean_ (V/m)
1	0.069	0.032	0.050
2	0.061	0.033	0.048
3	0.071	0.030	0.051
4	0.066	0.028	0.060
5	0.071	0.029	0.052
6	0.070	0.030	0.048
7	0.073	0.031	0.057

**Table 8.** Exposed energy density (at 24h), first week.

Service	GSM	DCS	UMTS
day of the week	W_24 h_ (J/m^2^)	W_24 h_ (J/m^2^)	W_24 h_ (J/m^2^)
1	1.108	0.235	0.575
2	0.843	0.248	0.547
3	1.143	0.212	0.593
4	1.004	0.185	0.841
5	1.156	0.205	0.628
6	1.137	0.205	0.525
7	1.212	0.220	0.749

### Analysis of measurements

#### Temporal exposure patterns

Figure 1 presents the temporal variations of E field over the first week for the GSM downlink band. All measurements aimed at starting at 8 a.m. until 8 a.m. the next day. Similar to the results in Mahfouz *et al.*^(43)^, Joseph and Verloock^(71)^, Manassas *et al.*^(72)^, Vermeeren *et al.*^(73)^, and Joseph *et al.*^(78)^, we also found higher short-term variations during the daytime (referred in literature as diurnal or active hours, i.e. 8 a.m−11 p.m.) than at night (nocturnal or night hours, i.e. 11 p.m.−8 a.m.) due to presence of the traffic. For example, in a personal RF-EMF measurement study conducted by Bolte and Eikelboom^(49)^, the total mean exposure during the day was found to be about twice the exposure than at night. In this study, such ratio varied from 2.9 to 3.8 for L1 over the week (with a mean ratio of 3.3). At least partly, this difference is likely to be explained by different types of activities of the population at different times of the day. Figure 1, thus, illustrates such a trend. It can be seen close to periodic day and night behaviour between the days (Monday to Sunday) while that trend might be different regarding the peak-hours.

In Figure 2 normalised histograms and the probability density functions (PDFs) of the instantaneous E field for the observed days are presented, illustrating the variability of the E field values. Cumulative distribution functions (CDFs) for different days of the week are presented in Figure 3. It should be noted that, for continuous distributions, the CDF is a continuous curve. The PDF and CDF, generally, give a complete description of the probability distribution of a random variable (i.e. E field). CDFs allow comparison on how much variations of the traffic are encountered daily. The largest variation of exposure is indicated by the widest CDF. Clearly, the distribution situated most to the left is for the lowest exposures. Based on Figure 3, for all days of the first week, 95% (p_95_) of measured E field values was below 0.123 for GSM (0.05 and 0.092 V/m for DCS and UMTS). In addition to all 14 considered locations, the lowest reference level according to ICNIRP^(2)^ in this study was for GSM band (41.3 V/m), while the maximum measured p_95_ value (with a 5% chance to occur), was 0.46 V/m. Therefore, the dominating source at all locations was GSM, following DCS and UMTS with p_95_ of 0.181 and 0.143 V/m, respectively.

#### Descriptive statistics of instantaneous values

Table 1 summarises the values of the instantaneous measurements for all three downlink frequency bands at L1: E_max_, E_min_, E_mean_, the 50^th^ percentile (p_50_ or median), the 95^th^ percentile (p_95_) and the standard deviation (σ).

Confidence interval of 95% was in range 0.5 to 0.8%, (mean 0.7%) of the daily mean for GSM and DCS and 0.5 to 0.7%, (mean 0.6%) for UMTS. E_min_ (given in Table 1) denotes the minimum E field values above the lower sensitivity level of equipment (EME Spy 140). The standard deviation was within 3 dB (DCS), 5 dB (UMTS) and 5.5 dB (GSM) on the week level. According to Plets *et al.*^(133)^, typical standard deviations for large sets of the field values might even be in the range from 7 to 10 dB. The ratio between the maximum and minimum instantaneous values varied from 18 to 23 dB (mean 20 dB) for GSM, 20 to 25 dB (mean 22 dB) for DCS and 11 to 18 dB (mean 16 dB) for UMTS, over the week. Exposure ratios (ER) for the maximum E field values over 14 locations (calculated according to Joseph *et al.*^(86)^), concerning the ICNIRP reference levels, ranged from 0.6% (DCS) over 1.8% (UMTS) to 2.2% (GSM). Specifically, ER at Location 4 (L4), where access for the general public is restricted, was found to be up to 12.2% for GSM (measurements were recorded at rooftop terrace where three mobile operators have deployed three base stations with 18 different antennas in total). According to Bornkessel *et al.*^(76)^, exposure in the areas surrounding GSM, DCS, and UMTS base stations are mainly in the range below 2% of ICNIRP field strength limits but may reach more than 10%. This agrees well with the results obtained in this study. Also, the mean levels of the GSM exposures at all locations were found to be mostly higher comparing to those of UMTS (i.e. for L1 ER varied from 0.7 to 3 dB (mean 2.3 dB), while DCS exposure was lower 3.5 to 6.5 dB (mean 4.7 dB) over the week).

Additionally, the average contribution (AC) to the total E field from these three services (calculated according to Joseph *et al.*^(86)^, and considering all 14 locations, was more than 74% for GSM and up to 11% and 15% for DCS and UMTS, respectively. Maximal contributions (MC) at particular locations reached up to 98% (L4), 47% (L7) and 38% (L4), for GSM, DCS, and UMTS.

### Assessment of variations in exposures due to averaging

To reduce the short-term variations and to emphasise the trend of the signal during longer time periods, an averaging method should be applied on the dataset. According to the international exposure guidelines, three averaging times are necessary to be considered, i.e. less than 6-min^(74)^, 6-min^(2)^ and 30-min^(3, 5)^. According to ITU-T K.100^(74)^, a shorter averaging time is acceptable if a ‘larger uncertainty is allowed or if conservatively large margin to the exposure limit due to averaging is introduced.’ In addition, the reference levels itself are evaluated in the absence of a human body, so the 6-min averaging might not always be necessary for all far-field assessments^(74)^. Considering the measurements, the time duration (which might be equal to averaging time) is related to the time constant for the partial body heating to reach the steady-state against external stimulation.

The effects of using different averaging times were investigated earlier in Colombi *et al.*^(134)^ where was found that time-averaging over 6-min and 15-min resulted in very similar statistics, indicating that the obtained measurement results are relevant for the realistic EMF exposure assessments. Similar to these results, in ITU-T K.91^(51)^ was stated that ‘a comparison of measurement results for 1-min and 6-min averaging time shows that the difference in results is on the level lower than 1 dB’. According to Kim *et al.*^(135)^, if any time-averaged value between 1-min and 6-min is very small (under 0.5 dB in standard deviation averaged over each minute), then 1-min is considered enough to evaluate the level of human exposure to RF-EMF in South Korea. However, if the standard deviation is larger than 0.5 dB, the 6-min averaging time is applied. In Foster *et al.*^(70)^. was stated that ‘the excessively long averaging times might permit excessive short-term exposures, while too-short times might be overly conservative by excluding short-term variations in exposure that are thermally innocuous.’ The results in Foster *et al.*^(70)^. generally confirmed the 6-min averaging time in the current RF exposure limits, however, suggesting the need for additional limits on fluence for brief high-fluence pulses at mm-wave frequencies.

#### Time-averaged exposure patterns

Averaging over short times still contain large variations in the results and much white noise. The powerful tool to extract the trend from the random variations of the non-stationary signals may be averaging over longer times. Therefore, we calculated the running average (in literature also referred as moving, sliding or rolling) with the variable averaging time (the size of averaging windows correspond to averaging time starting at 10 s to 24 h, with 10 s time-step) obtaining the dependence of how much the measurement result is dependent on the time of measurement. It means that we calculated the maximum, minimum, mean and median over each data set of averaged values. The results of such analysis on a daily level, for different days of the week, is presented in Figure 4. For example, when the 6-min averaging (which included 36 successive measurement samples corresponding to the measurement cycle of 10 s) was applied on the instantaneous values, then 8604 different 6-min datasets of RMS values was generated resulting in the same number of averaged values (one per each running dataset) to calculate the maximum, minimum, mean and median of them. As expected, by increasing the averaging time (i.e. up to 6-min), the maximum and minimum of 6-min averaged values showed lower variability in results compared to those of instantaneous ones (note that in case of 24 h averaging time the instantaneous values are equal to 10 s time-averaged ones or the initial ones on the curves from Figure 4), but the variability of readings for the maximum (E_max_) and minimum (E_min_) of averaged values still remained very high. This means, if we perform the 6-min mean measurement anytime during a day, we do not know if we either could catch the maximum or the minimum value of the day.

In order to give a better alignment to exposure standards and clarify the previous claim further, the ratio of 10 s and some other specific averaging times that can be found in the literature (i.e. 30 s, 1-min, 3-min, 6-min, 15-min, 30-min, 1 h, 10 h, 24 h) for the maximum, minimum, mean and median of averaged values are given in Table 2, 3 and 4 for GSM, DCS and UMTS, respectively.

As can be seen from the tables, when the averaging time is varied from 10 s to 6-min, the ratio of the maximum of averaged values varied from 5 dB (DCS and UMTS) to 7 dB (GSM) during the week. Ratio of the minimum of averaged values for all bands was 6 dB or lower. It was observed that the ratios of maximum (minimum) of averaged values at 6-min might be up to 4 dB lower (5 dB higher) than those at 1-min, ratios at 1 h 3 dB lower (3 higher) than those at 6-min, ratios at 10 h up to 6 dB lower (3.5 higher) than those at 1 h, etc. It can be concluded: if the averaging time is shorter, the number of measured samples (acquired in the averaging time) becomes smaller; thus the averaged values seem to differ greatly from those at longer averaging times. Such a trend might be observed between 1-min and 6-min averaged values. Large variations of averaged values over short times may be heavily caused by the changes in the output level of the base station due to traffic conditions or other factors discussed earlier. Therefore, the variations between the maximum and minimum of the instantaneous values (i.e. ratio of field size E_max_/E_min_) at L1 could eventually reach up to 23 dB (GSM), 25 dB (DCS) and 19 dB (UMTS). Those ratios at 6-min are confirmed to be lower and up to 15, 20 and 11 dB, respectively. By increasing the averaging time (up to 24 h) the ratios between the maximum and minimum averaged curves (values) from Figure 4 tend to be much smaller from those at 6-min (i.e. within 5 dB or less after 10 h of averaging) after which both curves converge slowly to an averaged E field value of the whole day (namely E_24 h_). Also, the curves of mean and median converge to the same 24 h averaged value (E_24 h_) like the maximum and minimum ones. Therefore, all the 24 h averaged values (E_24hmax_, E_24hmin_, E_24hmean_ and E_24hmedian_) are much more stable in terms of variations over days of the week (up to 1.5 dB of the week mean level obtained with the 24 h averaging) compared to variations of maximum and minimum of the instantaneous values, where the ratio between them was found to be in relation 18:1 (or 25 dB for DCS) on a daily level. Having that in mind, all the curves averaged over the 24 h from Figure 4. illustrate a kind of periodicity and repetition. Further, Figure 4 shows that the averaged curves also change from day-to-day. Some of the curves might be more variable at the lower averaging times, in some cases until the end of the day and, therefore, the averaging of the whole day (24 h) is needed. To give a better overview of the previous discussion regarding the ratio between the maximum and minimum of averaged values, the results are obtained in Table 5.

Additionally, from Figure 4, it can be observed that, for each day, there is a ‘knee-value’, estimated to happen between approximately 1.5E_24hmax_ and 2E_24hmax_ (for the maximum averaged curve) and between 0.5E_24hmin_ and 0.7E_24hmin_ (for the minimum averaged curve) to which the maximum (minimum) averaged curve drastically decrease (increase). After that, both curves slowly converge to E_24 h_ value. This sharp decrease (increase) happens for shorter averaging times, mostly up to 6-min.

The mean and median of averaged values are not mainly influenced by averaging as those of the maximum and minimum. Based on Figure 4, for shorter averaging times (i.e. from 10 s to 6-min), larger variations of the mean and median of averaged values occur (up to 0.2 dB for mean and 0.8 dB for median) comparing to those with much larger averaging times (less than 0.1 dB for mean and 0.4 dB for median) for all bands. According to Joseph *et al.*^(78)^, the median exposure values are more interesting for the epidemiological studies, while the maximum values are more often important for the authorities and legislation (e.g. Brussels Capital Region^(136)^).

Contrariwise to the mean and median of averaged values, the standard deviation always decreases with the increasing of averaging time. However, a faster or slower trend of convergence to zero after the 24 h averaging time may appear. The ratio of the standard deviation of averaged values at 10 s and standard deviation at different averaging times (σ__10s_/σ__i_, i = the size of running average) was, also, determined and given in Table 6. Figure 5 illustrates the relative standard deviation (RSD) of the averaged values for variable averaging times (10 s to 24 h, time step 10 s). Standard deviation is normalised to the 24 h mean value of the week, E_24hmean_week_:

(1) $$E_{24hmean\_week}=\frac{1}{n}\left(\overset{n}{\underset{j}{\sum }}E_{24hmean\_j}\right)$$

where: j = 1…n, n = 7. The 24 h averaged mean values (E_24hmean_) are given in Table 7. As can be seen from Figure 5, the relative standard deviation at 10 s of averaging decreased from approximately 32% to approximately 10% at 10 h. Similar behaviour can be observed for DCS (from 32–12%) and UMTS (from 27–10%).

### Integral-based (cumulative) exposure calculation

The main goal of our study, considering all the previously stated, was to find a new criterion (besides the time-averaging) that will, with the minimum deviations, under more or less same conditions, give results that show the total (24 h) level of irradiation of the measured location. The idea was, in fact, depending on the environment, to get the results of measurements that are stable (using the time-averaging we either obtained that the E_24hmean_ values for all days and all bands remained within ±20% or less of the E_24hmean_week_ value).

However, there is no a day which is the etalon to compare with and, therefore, the authors tried to find the measure for quantification which is sustainable. Based on Figure 4, the averaging process cannot completely eliminate the daily variations but will reduce them significantly. That is why we proposed the integral-based (cumulative) measure of the whole day. Therefore, the calculation of the exposed energy density (named W) transferred through an observed place (measured location) is equivalent to calculation of the instantaneous power density values (S) in a given time period (the measured E field samples are rescaled over the vacuum impedance on Poynting vector to have a physical dimension in W/m^2^). When the integration of S over the time is made (accumulated), the new dimension is obtained in J/m^2^ (note that the source contributions are only additive in the units of W/m^2^). So, power density (which is a continuous function) is linearly related to the exposed energy density as they provide the same information.

The integral-based measure is, thus:

• a cumulative sum of the instantaneous values of power density S (as exposure increases, the integral-based measure also increases),

• a monotonous non-decreasing function (because theoretically it can be zero),

• equivalent with the 24 h time-averaged measure (the difference is in dividing the E_24 h_ value with 1/Т_0_, where T_0_ is observed period of time).

If E (S) is averaged enough (or integrated) during the time, then E (S) becomes time-independent, and the problem of the measurement repeatability is solved. The repeatability of measurements is important for the predefined method of measurements to get the results that do not depend on the time when the measurements were recorded (basically, if the measurement is such that it can be recorded at any time, then it is the relevant measurement). To get that, it is necessary to apply an averaged or integral-based measure.

#### Integral-based (cumulative) exposure patterns

The results of the integral-based measure are illustrated in Figure 6. Curves in Figure 6 have a higher upslope when the instantaneous E (S) values are higher (as they add more contribution to the cumulative sum of S, especially the peak ones). According to Figure 7, integration in time gives minor deviations in daily exposure between days of the week which also tend to stay within approximately ±20% of the mean exposed energy density value of the week (W_24mean_week_) against the ratio of the maximum and minimum instantaneous values on a daily level (of up to 25 dB for DCS). The mean exposed energy density value of the week is calculated according to:

(2) $$W_{24hmean\_week}=\frac{1}{n}\left(\overset{n}{\underset{j}{\sum }}W_{24h\_j}\right)$$

where: j = 1…n, n = 7.

Exposure from GSM band seemed to be slightly higher during the weekend, indicating higher upslope between 6 and 10 p.m. in Figure 6. However, considering the relation between the peak-hours and integral-based measure, the following may be stated: if there is a peak-hour/s in terms of the traffic, in case of integration of the instantaneous values (S) in time, the peak-hour/s can be at any time, and from that side has no influence on the specific time of measurement during a day, as long as the exposure over the whole day is observed. Further, if we had our integral-based measure differentiated over the time, the curves illustrated in Figure 6 would be returned to the form of the instantaneous curves in Figure 1 (after dividing with an impedance of free space), which contain much white noise and are statistically darker. The results of the integral-based measure are given in Table 8. Here should be noted that the time-averaged and integral-based measure were, also, tested on the data which were sourced from surveys conducted in Hungary, Greece, Serbia, Romania, and Spain. More details in the Acknowledgement section.

### Strengths, limitations, and implications

Our study has several strengths. We collected the data over nine months at 14 different locations which resulted in approximately 5.1 million data samples (1.7 million for each band). We also included GSM service which was the most frequently used type of service in the past, making the results highly relevant. A further strength of the study was avoiding of the shielding due to the presence of the human body during the measurement process with the PEM. We also reduced the measurement results variability on a daily/weekly level either by using the 24 h time-averaging or integral-based measure. Also, it has been shown that all measurement results are at least 35 dB lower than the lowest reference level to protect against the RF-EMF. Having in mind the results obtained during the third week of measurements (6 months later, the material in online supplementary), it can be concluded there is a possibility that EMF variations over the week may also show the variability in exposure levels in a long-term sense, depending on the date and time of the measurements during the year, and due to the constant need for the new base station deployments.

Our study has several limitations. Our research was insufficiently large to understand the variability of the results associated with other types of RF technology (for which we also collected the data with the PEM). Some uncertainties might be related to the measurements results due to the limitation of the PEMs. Similarly, due to the dependence of the battery autonomy in regard with the sampling rate of the PEM, we could not use the higher sampling rate (i.e. to be 4 s for the PEM used in the study) to acquire more data for statistical analysis.

At present, the analysis is continuously updated, taking into consideration the latest measurements (after 2016) and emerging technologies. This concept could also be used to give the measurement results physically and possibly biological sense at the same time. Currently, both ICNIRP and IEEE are undergoing periodic revision, and this may be an appropriate time to reconsider the averaging time as it appears in these limits. Therefore, the authors believe that the results, procedures, and methodologies presented in this paper, considering the 24 h time-averaged and integral-based exposure, could be of important use for elaborating the standards and gaining insight regarding in which environments the highest exposures occur and due to which sources (especially in epidemiological studies). The new method (integral-based) could be tested to other emissions as well (besides the mobile communication systems). It may be expected that the variations of the EMF in the outdoor environments are much higher. As a result, the proposed method could also be validated in such environments in terms of its performance.

In future research, an optimal averaging/integration time (which might be somewhat lower than 24 h due to the practical reasons regarding the duration of measurements) will be examined using a data set of higher temporal resolution, having in mind that the number of samples for calculating the time-averaged and integral-based exposure may be of high importance as equally or more as the averaging or integration time. Also, the exposed energy density cycles of much longer periods should be observed as well as the patterns of the variation associated with different places of the city.

## CONCLUSION

The objective of this study was to evaluate the method for the measurement results variability reduction due to temporal variations of E field levels from the wireless telecommunication networks (GSM/DCS/UMTS) and to increase the repeatability of measurements. The authors concluded that the monitoring of exposure from the base stations with the PEM is useful due to the satisfying repeatability of exposure levels, despite the high temporal variations. Significant ratios of E field levels were found between the maximum and minimum instantaneous ones as well as between maximum and minimum of the 6-min averaged ones. That fact is crucial for the establishment of a new measure regarding the exposure expression. Even a preliminary study, such as the one reported here, has highlighted the need for another numerical approach to interpreting the variations of the measurement results correctly.

Further, an overview of the potential instabilities that could be associated with the short-term measurements and averaging is discussed, and the significance of it was questioned due to the high variability of the measurement results. However, time-averaging gives less dependence on the time when the measurement was recorded. The higher the running average, the results are more stable. Obviously, the variations are rapidly decreasing within the first few minutes of averaging. When the 6-min time was applied, the variations decreased slightly, but still remained very high (the ratio of 6-min averaged E_max_/E_min_ values was up to 20 dB). This suggests that the 6-min averaging time is too short of giving the correct readings. Therefore, the short-term exposure assessment made under standard procedural restrictions is not so reliable when it comes to conclusions on the long-term exposure levels. Even though the exposure of the general public is known to be well below the established ICNIRP guidelines, monitoring of the long-term exposure is important from an environmental assessment point of view and characterisation of the temporal behaviour of different signals (day/night, days over the week/s, type of technology, etc.). The 24 h measurements are, though, more illustrative; however, they have no complete overview of the repeatability that seems to occur in the signal. This means that the measurements should be extended to even longer periods. Having that in mind, the suggestion for accurate measurements is the use of a portable data-logging system for continuous monitoring of the EMF levels over a sufficiently long period of time (i.e. measurement for the whole day and over the weak, not at peak-hour!). Although, the long-term measurements are practically difficult to execute, time-consuming and more expensive, they provide a much clearer picture of the variation of E field strength over the time, which has recently been imposed as one of the most important factors in the area of EMF testing, as well as epidemiological studies of the effects on human health. Therefore, the long-term measurements remain necessary for validation of the true exposure assessment turning the emissions into something objective and, when presented to the public in an understandable format, help diminish the unawareness and helplessness of the public.

From the other side, in case of the short-term measurements, it is not an easy task to find the ‘worst time’ of the day to take the 6-min mean measurements, because it depends on the exact place and its temporal trend. Although each single 6-min mean measurement is associated with the uncertainties, it would still provide a good scientific basis that allows the estimation of the general public exposure levels.

In this study, the authors took a different approach to quantify the RF-EMF exposure, questioning the existing measurements and averaging time of 6-min, proposing the 24 h averaging and measurements and introducing the new integral-based measure. The originality of approach consisted in the evaluation of exposure from the GSM/DCS/UMTS downlink bands by using a procedure that delivers the 24 h averaged value and amount of the exposed energy density transferred through a certain place (measured location) over a certain time (24 h). The procedure was evaluated using the Python programming language that processed the data, enabling the consistent long-term exposure assessment over the time.

The novelty consists in: (i) approaching a different philosophy of statistical characterisation of the exposure in non-ionising radiation that came from the low-dose cumulative concept in ionising radiation (i.e. cumulative amount of the averaged/actual dose received over the time) and (ii) gaining an absolute independence from any information provided by the mobile operators regarding technical/traffic parameters. Therefore, the authors think that both the 24 h time-averaged and integral-based measures much better illustrate the exposure during the day and over the week.

## Supplementary Material

Supplementary_material_for_Radiation_Protection_Dosimetry_Manuscript_2019_ncz154

## References

[1] Vecchia P., Matthes R., Ziegelberger G., Lin J., Saunders R., Swerdlow A. Exposure to high-frequency electromagnetic fields, biological effects and health consequences (100 kHz–300 GHz). International Commission on Non-Ionizing Radiation Protection (Oberschleissheim, Germany) (2009).

[2] International Commission on Non-Ionizing Radiation Protection (ICNIRP). Guidelines for limiting exposure to time-varying electric, magnetic and electromagnetic fields (up to 300 GHz). Health Phys. 74, 494–522 (1998). 9525427

[3] Federal Communications Commission (FCC). Evaluating Compliance with FCC Guidelines for Human Exposure to Radio-Frequency Electromagnetic Fields, Supplement C to OET Bulletin 65, Washington DC (2001).

[4] Australian Radiation Protection and Nuclear Safety Agency. Radiation protection standard - maximum exposure levels to radiofrequency fields-3 kHz to 300 GHz. ARPANSA Radiation Protection Series, Publication No. 3 (2002).

[5] Institute of Electrical and Electronics Engineers (IEEE). IEEE standard for safety levels with respect to human exposure to radio frequency electromagnetic fields, 3 kHz–300 GHz. Std C95.1. New York, NY: IEEE (2005).

[6] CENELEC (European Committee for Electrotechnical Standardisation). TC 106x WG1 EN 50492 in situ. Basic standard for the in-situ measurement of electromagnetic field strength related to human exposure in the vicinity of base stations. Brussels, Belgium: CENELEC (2008).

[7] Sanchez-Hernandez D. A. High-Frequency Electromagnetic Dosimetry (London, UK; Boston, MA, USA: Artech House)274 (2009) 10.1093/rpd/ncr216.

[8] International Telecommunication Union-Telecommunication Standardization Sector (ITU-T). FG-SSC: Electromagnetic field (EMF) considerations in smart sustainable cities (2014). Available from: https://www.itu.int/en/ITU-T/focusgroups/ssc/Pages/default.aspx (Last accessed 18 November 2018).

[9] Markov M. and Grigoriev Y. G. Wi-Fi technology-an uncontrolled global experiment on the health of mankind. Electromagn. Biol. Med. 32, 200–208 (2013) 10.3109/15368378.2013.776430. 23675623

[10] Allen S. Radiofrequency field measurements and hazard assessment. J. Radiol. Prot. 11, 49–62 (1991) 10.1088/0952-4746/11/1/005.

[11] Bhatt C. R., Redmayne M., Abramson M. J. and Benke G. Instruments to assess and measure personal and environmental radiofrequency-electromagnetic field exposures. Australas. Phys. Eng. Sci. Med. 39, 29–42 (2016a) 10.1007/s13246-015-0412-z. 26684750

[12] Miyakoshi J. Cellular biology aspects of mobile phone radiation In: Advances in Electromagnetic Field in Living Systems: Health Effects of Cell Phone Radiation. vol 5, Lin J. C., Ed. (New York: Springer) pp. 1–33 (2009) 10.1007/978-0-387-92736-7.

[13] International Telecommunication Union-Telecommunication Standardization Sector (ITU-T). Recommendation K.91, Supplement 4: Electromagnetic field considerations in smart sustainable cities (2015). Available from: https://www.itu.int/ITU-T/recommendations/rec.aspx?rec=12688. (Last accessed 18 November 2018).

[14] Neubauer G., Preiner P., Cecil S., Mitrevski N., Gonter J. and Garn H. The relation between the specific absorption rate and electromagnetic field intensity for heterogeneous exposure conditions at mobile communications frequencies. Bioelectromagnetics 30, 651–662 (2009) 10.1002/bem.20519. 19551765

[15] Vrijheid M., Armstrong B.K., Bédard D., Brown J., Deltour I., Iavarone I., Krewski D., Lagorio S., Moore S., Richardson L., Giles G.G., McBride M., Parent M.E., Siemiatycki J., Cardis E. Recall bias in the assessment of exposure to mobile phones. J. Expo. Sci. Environ. Epidemiol. 19(4), 369–381 (2009) 10.1038/jes.2008.27. 18493271

[16] Mahfouz Z., Gati A., Lautru D., Wiart J. and Hanna V. F. SAR assessment and analysis of cumulative body exposure to multi transmitters from a mobile phone. IEEE Topical Conference on Biomedical Wireless Technologies, Networks, and Sensing Systems (Bio-WireleSS) (2012). 10.1109/BioWireless.2012.617272

[17] Smith T. J. Occupational exposure and dose over time, limitations of cumulative exposure. Am. J. Ind. Med. 21, 35–51 (1992) 10.1002/ajim.4700210107. 1553984

[18] Axelson O., Forastiere F. and Frederickson M. Assessing dose-response relationships by cumulative exposures in epidemiological studies. Am. J. Ind. Med. 50, 217–220 (2007) 10.1002/ajim.20377. 17315178

[19] McLean A.R., Adlen E. K., Cardis E., Elliott A., Goodhead D.T., Harms-Ringdahl M., Hendry J.H., Hoskin P., Jeggo P.A., Mackay D.J.C., Muirhead C.R., Shepherd J., Shore R.E., Thomas G.A., Wakeford R., Godfray H.C.J. A restatement of the natural science evidence base concerning the health effects of low-level ionizing radiation. Proc. Biol. Sci. 284, 20171070 (2017) 10.1098/rspb.2017.1070. 28904138 PMC5597830

[20] UN, United Nations Scientific Committee on the sources and effects of Ionizing radiation. Report on the Effects of Atomic Radiation to the General Assembly, 2000. Medical Radiation Exposures (New York: United Nations) (2001) http://www.unscear.org/unscear/en/publications/2000_1.html. (Last accessed 20 November 2018).

[21] Picano E. Sustainability of medical imaging. Education and Debate. BMJ 328, 578–580 (2004) 10.1136/bmj.328.7439.578. 15001510 PMC381057

[22] Togni M., Balmer F., Pfiffner D., Maier W., Zeiher A. M. and Meier B. Working Group, Interventional Cardiology and Coronary Pathophysiology, European Society of Cardiology. Percutaneous coronary intervention in Europe 1992–2001. Eur. Heart J. 25, 1208–1213 (2004) 10.1016/j.ehj.2004.04.024. 15246638

[23] Vano E. and Faulkner K. ICRP special radiation protection issues in interventional radiology, digital and cardiac imaging. Radiat. Prot. Dosimetry 117, 13–17 (2005) 10.1093/rpd/nci702. 16461540

[24] Regulla F. D. and Eder H. Patient exposure in medical X-Ray imaging in Europe. Radiat. Prot. Dosimetry 114, 11–25 (2005) 10.1093/rpd/nch538. 15933076

[25] Lucas F. L., DeLorenzo M. A., Sievers A. E. and Wennberg D. E. Temporal trends in the utilization of diagnostic testing and treatments for cardiovascular disease in the United States, 1993–2001. Circulation 113, 374–379 (2006) 10.1161/CIRCULATIONAHA.105.560433. 16432068 PMC2121186

[26] Einstein A. J., Moser K. W., Thompson R. C., Cerqueira M. D. and Henzlova M. J. Radiation dose to patients from cardiac diagnostic imaging. Circulation 116, 1290–1305 (2007) 10.1161/CIRCULATIONAHA.107.688101. 17846343

[27] FDA Warning. Center for Devices and Radiological Health. Public health notification: reducing radiation risk from computed tomography for pediatric and small adult patients (2001). 10.1067/mtn.2002.121511

[28] Griffey R. T. and Sodickson A. Cumulative radiation exposure and cancer risk estimates in emergency department patients undergoing repeat or multiple CT. AJR AJR Am J Roentgenol 192, 887–892 (2009) 10.2214/AJR.08.1351. 19304691

[29] Choi S. J., Kim E. Y., Kim H. S., Choi H.-Y., Cho J., Yang H. J. and Chung Y. E. Cumulative effective dose associated with computed tomography examinations in adolescent trauma patients. Pediatr. Emerg. Care 30(7), 479–482 (2014) 10.1097/PEC.0000000000000165. 24977992

[30] Brenner D. J. and Hall E. J. Computed tomography—an increasing source of radiation exposure. N. Engl. J. Med. (2007) 10.1056/NEJMra072149. 2277Y2284 . 18046031

[31] Clairand I., Clairand I., Bordy J-M., Carinou E., Daures J., Debroas J., Denozière M., Donadille L., Ginjaume M., Itiè C., Koukorava C., Krim S., Lebacq A-L., Martin P., Struelens L., Sans-Mercè M, Vanhavere F. Use of active personal dosimeters in interventional radiology and cardiology: tests in laboratory conditions and recommendations-ORAMED project. Radiat. Meas. 46, 1252–1257 (2011) 10.1016/j.radmeas.2011.07.008.

[32] Bedetti G., Botto N., Andreassi M. G., Traino C., Vano E. and Picano E. Cumulative patient effective dose in cardiology. Br. J. Radiol. 81, 699–705 (2008) 10.1259/bjr/29507259. 18508874

[33] U.S. Environmental Protection Agency Office of Radiation Protection Programs Home Page; Health Effects. https://www.epa.gov/radiation (Last accessed 22 November 2018).

[34] Ginjaurme M., Bolognese-Milsztajn T., Luszik-Bhadra M., Vanhavere F., Wahl W. and Weeks A. Overview of active personal dosimeters for individual monitoring in the European Union. Radiat. Prot. Dosimetry 125, 261–266 (2007) 10.1093/rpd/ncl136. 16980319

[35] Amis E. S. Jr., Butler P.F., Applegate K.E., Birnbaum S.B., Brateman L.F., Hevezi J.M., Mettler F.A., Morin R.L., Pentecost M.J., Smith G.G., Strauss K.J., Zeman R.K. American College of Radiology White Paper on Radiation Dose in Medicine. J Am Coll Radiol 4, 272–284 (2007) 10.1016/j.jacr.2007.03.002. 17467608

[36] Ullrich R. L., Jernigan M. C., Satterfield L. C. and Bowles N. D. Radiation carcinogenesis: time-dose relationships. Radiat. Res. 111(1), 179–184 (1987). 3602353

[37] Brenner D. J. and Elliston C. D. Estimated radiation risks potentially associated with full-body CT screening. Radiology 232, 735–738 (2004) 10.1148/radiol.2323031095. 15273333

[38] Katz S. I., Saluja S., Brink J. A. and Forman H. P. Radiation dose associated with unenhanced CT for suspected renal colic: impact of repetitive studies. AJR Am. J. Roentgenol. 186, 1120–1124 (2006) 10.2214/AJR.04.1838. 16554590

[39] International Commission on Radiological Protection. ICRP publication 82: protection of the public in situations of prolonged radiation exposure. Ann. ICRP 29(1–2), 1–124 (1999). 10962071

[40] Goodhead D. T. and Fifth Warren K. Sinclair keynote address: issues in quantifying the effects of low-level radiation. Health Phys. 97, 394–406 (2009) 10.1097/HP.0b013e3181ae8acf. 19820449

[41] International Telecommunication Union-Telecommunication Standardization Sector (ITU-T). Recommendation K.52: Guidance on complying with limits for human exposure to electromagnetic fields (2018). Available from: https://www.itu.int/rec/T-REC-K.52/en. (Last accessed 18 November 2018).

[42] Varsier N., Plets D., Corre Y., Vermeeren G., Joseph W., Aerts S., Martens L., Wiart J. A novel method to assess human population exposure induced by a wireless cellular network. Bioelectromagnetics 36, 451–463 (2015) 10.1002/bem.21928. 26113174

[43] Mahfouz Z., Gati A., Lautru D., Wong M. F., Wiart J. and Hanna V. F. Influence of traffic variations on exposure to wireless signals in realistic environments. Bioelectromagnetics 33, 288–297 (2011) 10.1002/bem.20705. 21960463

[44] LEXNET D2.1. Vermeeren G., Thielens A., Aerts S., Joseph W., Martens L., Oliveira C., Mackowiak M., Correia L.M., Pejanović-Djurišić M., Veljović Z., Nešković A., Koprivica M., Gati A., Varsier N., Hadjem A., Wiart J., Conil E. D2.1 Current metrics for EMF exposure evaluation, LEXNET project (2013). http://www.lexnet.fr/project-progress/publicdeliverables.html (Last accessed 15 November 2018).

[45] Aerts S., Plets D., Verloock L., Martens L. and Joseph W. Assessment and comparison of RF exposure dose in femtocell and macrocell scenario. Radiat. Prot. Dosimetry 162, 236–243 (2013) 10.1093/rpd/nct272. 24185915

[46] Lauer O., Frei P., Gosselin M. C., Joseph W., Röösli M. and Fröhlich J. Combining near- and far-field exposure for an organ-specific and whole-body RF-EMF proxy for epidemiological research: a reference case. Bioelectromagnetics 34, 366–374 (2013) 10.1002/bem.21782. 23417714

[47] Plets D., Joseph W., Aerts S., Vanhecke K. and Martens L. Prediction and comparison of downlink electric-field and uplink localized SAR values for realistic indoor wireless planning. Radiat. Prot. Dosimetry 162, 487–498 (2014) 10.1093/rpd/ncu019. 24553049

[48] Alhekail Z. O., Hadi M. A. and Alkanhal M. A. Public safety assessment of electromagnetic radiation exposure from mobile base stations. J. Radiol. Prot. 32, 325–337 (2012) 10.1088/0952-4746/32/3/325. 22854221

[49] Bolte J. F. B. and Eikelboom T. Personal radiofrequency electromagnetic field measurements in the Netherlands: exposure level and variability for everyday activities, times of day and types of area. Environ. Int. 48, 133–142 (2012) 10.1016/j.envint.2012.07.006. 22906414

[50] Wiart J. Radio-Frequency Human Exposure Assessment: From Deterministic to Stochastic Methods (London, UK; Hoboken, NJ, USA: John Wiley & Sons) (2016.

[51] International Telecommunication Union-Telecommunication Standardization Sector (ITU-T) Recommendation K.91. Guidance for assessment, evaluation and monitoring of the human exposure to radio frequency electromagnetic field (2012). Available from: https://www.itu.int/rec/T-REC-K.91/en. (Last accessed 18 November 2018).

[52] Cooper J., Marx B., Buhl J. and Hombach V. Determination of safety distance limits for a human near a cellular base station antenna, adopting the IEEE standard or ICNIRP guidelines. Bioelectromagnetics 23, 429–443 (2002) 10.1002/bem.10037. 12210561

[53] Joseph W., Verloock L. and Martens L. Accurate low-cost measurement technique for occupational exposure assessment of base station antennas. Electron Lett. 39, 886–887 (2003) 10.1049/el:20030621.

[54] Joseph W. and Martens L. Comparison of safety distances based on the electromagnetic field and based on the SAR for occupational exposure of a 900-MHz Base station antenna. IEEE Trans. Electromagn. Compat. 47, 977–985 (2005) 10.1109/TEMC.2005.854100.

[55] Martínez-Búrdalo M., Martín A., Anguiano M. and Villar R. On the safety assessment of human exposure in the proximity of cellular communications base-station antennas at 900, 1800 and 2170 MHz. Phys. Med. Biol. 50, 4125–4137 (2005) 10.1088/0031-9155/50/17/015. 16177535

[56] van Wyk M. J., Bingle M. and Meyer F. J. C. Antenna modeling considerations for accurate SAR calculations in human phantoms in close proximity to GSM cellular base station antennas. Bioelectromagnetics 26, 502–509 (2005) 10.1002/bem.20122. 15931680

[57] Kos B., Valič B., Kotnik T. and Gajšek P. Exposure assessment in front of a multi-band base station antenna. Bioelectromagnetics 32, 234–242 (2011) 10.1002/bem.20640. 21365667

[58] Dimbylow P. J. Fine resolution calculations of SAR in the human body for frequencies up to 3 GHz. Phys. Med. Biol. 47, 2835–2846 (2002) 10.1088/0031-9155/47/16/301. 12222849

[59] Neubauer G., Cecil S., Preiner P., Mitrevski N., Vermeeren G., Joseph W., Martens L., Kühn S. and Kuster N. The relation between SAR and the electromagnetic field distribution for heterogeneous exposure conditions. Antennas and Propagation, EuCAP 2006. pp 1–5 (2006). 10.1109/EUCAP.2006.4584850

[60] Dimbylow. P. J. and Bolch W. Whole body averaged SAR from 50 MHz to 4 GHz in the University of Florida child voxel phantoms. Phys. Med. Biol. 52, 6639–6649 (2007) 10.1088/0031-9155/52/22/006. 17975288

[61] Lacroux F., Conil E., Carrasco A., Gati A., Wong M. F. and Wiart J. Specific absorption rate assessment near a base station antenna (2140 MHz): Some key points. Ann. Telecomm. 63, 55–64 (2008) 10.1007/s12243-007-0005-2.

[62] Conil E., Hadjem A., Lacroux F., Wong M. F. and Wiart J. Variability analysis of SAR from 20 MHz to 2.4 GHz for different adult and child models using FDTD. Phys. Med. Biol. 53, 1511–1525 (2008) 10.1088/0031-9155/53/6/001. 18367785

[63] Vermeeren G., Joseph W., Olivier C. and Martens L. Statistical multipath exposure of a human in a realistic electromagnetic environment. Health Phys. 94, 345–354 (2008) 10.1097/01.HP.0000298816.66888.05. 18332726

[64] Thielens A., Vermeeren G., Kurup D., Joseph W. and Martens L. Compliance boundaries for multiple-frequency base station antennas in three directions. Bioelectromagnetics 34, 465–478 (2013) 10.1002/bem.21778. 23361516

[65] IEC 62209-2:2010, Human exposure to radio frequency fields from hand-held and body-mounted wireless communication devices – Human models, instrumentation, and procedures – Part 2: Procedure to determine the specific absorption rate (SAR) for wireless communication devices used in close proximity to the human body (frequency range of 30 MHz to 6 GHz).

[66] IEC 62209-1:2016, Measurement procedure for the assessment of specific absorption rate of human exposure to radio frequency fields from hand-held and body-mounted wireless communication devices – Part 1: Devices used next to the ear (Frequency range of 300 MHz to 6 GHz).

[67] IEC 62232:2017, Determination of RF field strength, power density and SAR in the vicinity of radiocommunication base stations for the purpose of evaluating human exposure.

[68] International Telecommunication Union-Telecommunication Standardization Sector (ITU-T). Recommendation K.83: Monitoring of electromagnetic field levels (2011). Available from: https://www.itu.int/rec/T-REC-K.83-201103-I. (Last accessed 18 November 2018).

[69] International Telecommunication Union-Telecommunication Standardization Sector (ITU-T). Recommendation K.61: Guidance to measurement and numerical prediction of electromagnetic fields for compliance with human exposure limits for telecommunication installations (2008). Available from: https://www.itu.int/rec/T-REC-K.61-200309-S. (Last accessed 18 November 2018).

[70] Foster K. R., Ziskin M. C., Balzano Q. and Hirata A. Thermal analysis of averaging times in radiofrequency exposure limits above 1 GHz. IEEE Access (2018) 10.1109/ACCESS.2018.2883175.

[71] Joseph W. and Verloock L. Influence of mobile phone traffic on base station exposure of the general public. Health Phys. 99, 631–638 (2010) 10.1097/HP.0b013e3181db264f. 20938233

[72] Manassas A., Boursianis A., Samaras T. and Sahalos J. N. Continuous electromagnetic radiation monitoring in the environment: analysis of the results in Greece. Radiat. Prot. Dosimetry 151, 437–442 (2012) 10.1093/rpd/ncs028. 22434927

[73] Vermeeren G., Markakis I., Goeminne F., Samaras T., Martens L. and Joseph W. Spatial and temporal RF electromagnetic field exposure of children and adults in indoor microenvironments in Belgium and Greece. Prog. Biophys. Mol. Biol. 113, 254–263 (2013) 10.1016/j.pbiomolbio.2013.07.002. 23872299

[74] International Telecommunication Union-Telecommunication Standardization Sector (ITU-T). Recommendation K.100: Measurement of radio frequency electromagnetic fields to determine compliance with human exposure limits when a base station is put into service (2017). Available from: https://www.itu.int/rec/T-REC-K.100/en. (Last accessed 18 November 2018).

[75] Bürgi A., Scanferla D. and Lehmann H. Time-averaged transmitter power and exposure to electromagnetic fields from mobile phone base stations. Int. J. Environ. Res. Public Health 11, 8025–8037 (2014) 10.3390/ijerph110808025. 25105551 PMC4143847

[76] Bornkessel C., Schubert M., Wuschek M. and Schmidt P. Determination of the general public exposure around GSM and UMTS base stations. Radiat. Prot. Dosimetry 128, 40–47 (2007) 10.1093/rpd/ncm373. 17933788

[77] Joseph W., Vermeeren G., Verloock L., Heredia M. M. and Martens L. Characterization of personal RF electromagnetic field exposure and actual absorption for the general public. Health Phys. 95, 317–330 (2008) 10.1097/01.HP.0000318880.16023.61. 18695413

[78] Joseph W., Verloock L., Tanghe E. and Martens L. In-situ measurement procedures for temporal RF electromagnetic field exposure of the general public. Health Phys. 96, 529–542 (2009) 10.1097/01.HP.0000341327.37310.c8. 19359846

[79] Frei P., Mohler E., Neubauer G., Theis G., Bürgi A., Fröhlich J., Braun-Fahrländer C., Bolte J., Egger M., Röösli M. Temporal and spatial variability of personal exposure to radiofrequency electromagnetic fields. Environ. Res. 109, 779–785 (2009a) 10.1016/j.envres.2009.04.015. 19476932

[80] Röösli M., Frei P., Bolte J., Neubauer G., Cardis E., Feychting M., Gajsek P., Heinrich S., Joseph W., Mann S., Martens L., Mohler E., Parslow R.C., Poulsen A.H., Radon K., Schüz J., Thuroczy G., Viel J.F., Vrijheid M. Conduct of a personal radio-frequency electromagnetic field measurement study: Proposed study protocol. Environ. Health 9, 23 (2010) 10.1186/1476-069X-9-23. 20487532 PMC2898756

[81] 3rd Generation Partnership Project. Digital Cellular Telecommunications System (Phase 2+), Radio Subsystem Link Control (Cedex, France: 3GPP Mobile Competence Centre) (2005) 3GPP TS 05.08 v. 8.23.0.

[82] Sanchez M. G., Cuinas I. and Alejos A. V. Electromagnetic field level temporal variation in urban areas. Electron. Lett. 41, 233–234 (2005) 10.1049/el:20047507.

[83] ECC, Electronic Communications Committee. European Conference of Postal and Telecommunications Administrations. Measuring non-ionizing electromagnetic radiation (9 kHz–300 GHz). Copenhagen: ECC; ECC recommendation (02)04; 7–18 (2004). Available at: http://www.ero.dk.

[84] International Telecommunication Union-Telecommunication Standardization Sector (ITU-T). Recommendation K.70: Mitigation techniques to limit human exposure to EMFs in the vicinity of radiocommunication stations (2018). Available from: https://www.itu.int/rec/T-REC-K.70. (Last accessed 18 November 2018).

[85] Verloock L., Joseph W., Vermeeren G. and Martens L. Procedure for assessment of general public exposure from WLAN in offices and in wireless sensor network testbed. Health Phys. 98, 628–638 (2010) 10.1097/HP.0b013e3181c9f372. 20220371

[86] Joseph W., Verloock L., Goeminne F., Vermeeren G. and Martens L. Assessment of RF exposures from emerging wireless communication technologies in different environments. Health Phys. 102, 161–172 (2012) 10.1097/HP.0b013e31822f8e39. 22217589

[87] Gotsis A., Papanikolaou N., Komnakos D., Yalofas A. and Constantinou P. Nonionizing electromagnetic radiation monitoring in Greece. Ann. Telecomm. 63, 109–123 (2008) 10.1007/s12243-007-0006-1.

[88] Markakis I. and Samaras T. Radiofrequency exposure in Greek indoor environments. Health Phys. 104, 293–301 (2013) 10.1097/HP.0b013e31827ca667. 23361425

[89] Knafl U., Lehmann H. and Riederer M. Electromagnetic field measurements using personal exposimeters. Bioelectromagnetics 29, 160–162 (2008) 10.1002/bem.20373. 17929265

[90] Joseph W., Frei P., Roösli M., Thuróczy G., Gajsek P., Trcek T., Bolte J., Vermeeren G., Mohler E., Juhász P., Finta V., Martens L. Comparison of personal radiofrequency electromagnetic field exposure in different urban areas across Europe. Environ. Res. 110(7), 658–663 (2010a) 10.1016/j.envres.2010.06.009. 20638656

[91] Iskra S., McKenzie R. and Cosic I. Factors influencing uncertainty in measurement of electric fields close to the body in personal RF dosimetry. Radiat. Prot. Dosimetry 140, 25–33 (2010) 10.1093/rpd/ncp309. 20123893

[92] Iskra S., McKenzie R. and Cosic I. Monte Carlo simulations of the electric field close to the body in realistic environments for application in personal radiofrequency dosimetry. Radiat. Prot. Dosimetry 147, 517–527 (2011) 10.1093/rpd/ncq580. 21242165

[93] Bolte J. F. B., Van der Zande G. and Kamer J. Calibration and uncertainties in personal exposure measurements of radio-frequency electromagnetic fields. Bioelectromagnetics 32, 652–663 (2011) 10.1002/bem.20677. 21544843

[94] EN 50492:2008, Basic standard for the in-situ measurement of electromagnetic field strength related to human exposure in the vicinity of base stations.

[95] EN 50413:2008, Basic standard on measurement and calculation procedures for human exposure to electric, magnetic and electromagnetic fields (0 Hz – 300 GHz).

[96] Mann S. M., Addison D. S., Blackwell R. P. and Khalid M. Personal dosimetry of RF radiation: laboratory and volunteer trials of an RF personal exposure meter. Health Protection Agency HPA-RPD-008, (2005).

[97] Radon K., Spegel H., Meyer N., Klein J., Brix J., Wiedenhofer A., Eder H., Praml G., Schulze A., Ehrenstein V., von Kries R., Nowak D. Personal dosimetry of exposure to mobile telephone base stations? An epidemiologic feasibility study comparing the Maschek dosimeter prototype and the Antennessa SP-090 system. Bioelectromagnetics 27, 77–81 (2006) 10.1002/bem.20175. 16304690

[98] Lehmann H., Biner J., Eicher B., Fritschi P., Hermann U., Knafl U. and Rubin M. Benchmarking personal radiofrequency exposimeters. International conference and COST 281 workshop on emerging technologies, potential sensitive groups and health. Graz, Austria (2006).

[99] Neubauer G., Feychting M., Hamnerius Y., Kheifets L., Kuster N., Ruiz I., Schuz J., Uberbacher R., Wiart J. and Röösli M. Feasibility of future epidemiological studies on possible health effects of mobile phone base stations. Bioelectromagnetics 28, 224–230 (2007) 10.1002/bem.20298. 17080459

[100] Ha M., Im H., Kwon H., Kim N., Lee A. and Choi H. A preliminary study on personal exposure characterization of mobile phone base stations in Korea. Proceedings of the 29th Annual Meeting of the Bioelectromagnetics Society. Kanazawa, Japan, pp. 337–338 (2007).

[101] Breckenkamp J., Neitzke H. P., Bornkessel C. and Berg-Beckhoff G. Applicability of an exposure model for the determination of emissions from mobile phone base stations. Radiat. Prot. Dosimetry 131, 474–481 (2008) 10.1093/rpd/ncn201. 18676976

[102] Thuróczy G., Molnár F., Jánossy G., Nagy N., Kubinyi G., Bakos J., Szabó J. Personal RF exposimetry in urban area. Ann. Telecomm. 63, 87–96 (2008) 10.1007/s12243-007-0008-z.

[103] Röösli M., Frei P., Mohler E., Braun-Fahrländer C., Burgi A., Fröhlich J., Neubauer G., Theis G. and Egger M. Statistical analysis of personal radiofrequency electromagnetic field measurements with nondetects. Bioelectromagnetics 29, 471–478 (2008) 10.1002/bem.20417. 18421711

[104] Ahlbom A., Bridges J., de Seze R., Hillert L., Juutilainen J., Mattsson M. O., Neubauer G., Schüz J., Simko M., Bromen K. Possible effects of electromagnetic fields (EMF) on human health-opinion of the scientific committee on emerging and newly identified health risks (SCENIHR). Toxicology 246(2–3), 248–250 (2008) 10.1016/j.tox.2008.02.004. 18453044

[105] Thomas S., Kühnlein A., Heinrich S., Praml G., von Kries R. and Radon K. Exposure to mobile telecommunication networks assessed using personal dosimetry and well-being in children and adolescents: The German MobilEe-study. Environ. Health 7, 54 (2008a) 10.1186/1476-069X-7-54. 18983641 PMC2614418

[106] Thomas S., Kühnlein A., Heinrich S., Praml G., Nowak D., von Kries R. and Radon K. Personal exposure to mobile phone frequencies and well-being in adults: A cross-sectional study based on dosimetry. Bioelectromagnetics 29, 463–470 (2008b) 10.1002/bem.20414. 18393264

[107] Frei P., Mohler E., Bürgi A., Fröhlich J., Neubauer G., Braun-Fahrlander C. and Röösli M., QUALIFEX team. A prediction model for personal radio frequency electromagnetic field exposure. Sci. Total Environ. 408, 102–108 (2009b) 10.1016/j.scitotenv.2009.09.023. 19819523

[108] Viel J. F., Cardis E., Moissonnier M., de Seze R. and Hours M. Radiofrequency exposure in the French general population: Band, time, location and activity variability. Environ. Int. 35, 1150–1154 (2009) 10.1016/j.envint.2009.07.007. 19656570

[109] Berg-Beckhoff G., Blettner M., Kowall B., Breckenkamp J., Schlehofer B., Schmiedel S., Bornkessel C., Reis U., Potthoff P. and Schüz J. Mobile phone base stations and adverse health effects: phase 2 of a cross-sectional study with measured radio frequency electromagnetic fields. Occup. Environ. Med. 66, 124–130 (2009) 10.1136/oem.2008.039834. 19151228

[110] Frei P., Mohler E., Bürgi A., Fröhlich J., Neubauer G., Braun-Fahrländer C. and Röösli M., QUALIFEX Team. Classification of personal exposure to radio frequency electromagnetic fields (RF-EMF) for epidemiological research: Evaluation of different exposure assessment methods. Environ. Int. 36, 714–720 (2010) 10.1016/j.envint.2010.05.005. 20538340

[111] Joseph W., Vermeeren G., Verloock L. and Martens L. Estimation of whole-body SAR from electromagnetic fields using personal exposure meters. Bioelectromagnetics 31, 286–295 (2010b) 10.1002/bem.20561. 20041435

[112] Usher K. Indigenous higher degree research students making a difference to the Indigenous health agenda. Contemp. Nurse 37, 102–106 (2010). 21591833 10.5172/conu.2011.37.1.102

[113] Urbinello D. and Röösli M. Impact of one’s own mobile phone in standby mode on personal radiofrequency electromagnetic field exposure. J. Expo. Sci. Environ. Epidemiol. 23, 545–548 (2013) 10.1038/jes.2012.97. 23093102

[114] Urbinello D., Huss A., Beekhuizen J., Vermeulen R. and Röösli M. Use of portable exposure meters for comparing mobile phone base station radiation in different types of areas in the cities of Basel and Amsterdam. Sci. Total Environ. 468–469, 1028–1033 (2014a) 10.1016/j.scitotenv.2013.09.012. 24091124

[115] Urbinello D., Joseph W., Verloock L., Martens L. and Röösli M. Temporal trends of radiofrequency electromagnetic field (RF-EMF) exposure in everyday environments across European cities. Environ. Research 134, 134–142 (2014b) 10.1016/j.envres.2014.07.003. 25127524

[116] Thielens A., Agneessens S., Verloock L., Tanghe E., Rogier H., Martens L. and Joseph W. On-body calibration and processing for a combination of two radio frequency personal exposimeters. Radiat. Prot. Dosimetry 163, 58–69 (2015) 10.1093/rpd/ncu056. 24729592

[117] Blas J., Lago F. A., Fernández P., Lorenzo R. M. and Abril E. J. Potential exposure assessment errors associated with bodyworn RF dosimeters. Bioelectromagnetics 28, 573–576 (2007) 10.1002/bem.20355. 17654534

[118] Bahillo A., Blas J., Fernández P., Lorenzo R. M., Mazuelas S. and Abril E. J. E-field assessment errors associated with RF dosimeters for different angles of arrival. Radiat. Prot. Dosimetry 132, 51–56 (2008) 10.1093/rpd/ncn275. 18927131

[119] Bornkessel C., Blettner M., Breckenkamp J. and Berg-Beckhoff G. Quality control for exposure assessment in epidemiological studies. Radiat. Prot. Dosimetry 140, 287–293 (2010) 10.1093/rpd/ncq112. 20308051

[120] Neubauer G., Cecil S., Giczi W., Petric B., Preiner P., Fröhlich J. and Röösli M. The association between exposure determined by radiofrequency personal exposimeters and human exposure: a simulation study. Bioelectromagnetics 31, 535–545 (2010) 10.1002/bem.20587. 20564178

[121] Lauer O., Neubauer G., Röösli M., Riederer M., Frei P., Mohler E., Fröhlich J. Measurement setup and protocol for characterizing and testing radio frequency personal exposure meters. Bioelectromagnetics 33, 75–85 (2012) 10.1002/bem.20687. 21755521

[122] Juhász P., Bakos J., Nagy N., Jánossy G., Finta V. and Thuróczy G. RF personal exposimetry on employees of elementary schools, kindergartens and day nurseries as a proxy for child exposures. Prog. Biophys. Mol. Biol. 107, 449–455 (2011) 10.1016/j.pbiomolbio.2011.09.020. 21986474

[123] Bhatt C. R., Thielens A., Billah B., Redmayne M., Abramson M. J., Sim M. R., Vermeulen R., Martens L., Joseph W. and Benke G. Assessment of personal exposure from radiofrequency-electromagnetic fields in Australia and Belgium using on-body calibrated exposimeters. Environ. Res. 151, 547–563 (2016b) 10.1016/j.envres.2016.08.022. 27588949

[124] Hewett P. and Ganser G. H. A comparison of several methods for analyzing censored data. Ann. Occup. Hyg. 51, 611–632 (2007) 10.1093/annhyg/mem045. 17940277

[125] Ganser G. H. and Hewett P. An accurate substitution method for analyzing censored data. J. Occup. Environ. Hyg. 7, 233–244 (2010) 10.1080/15459621003609713. 20169489

[126] Regel S., Röösli M., Negovetic S., Schuderer J., Berdinas V., Huss A., Kuster N. and Achermann P. Effects of UMTS base station like exposure on well-being and cognitive performance. Environ. Health Perspect. 114, 1270–1275 (2006) 10.1289/ehp.8934. 16882538 PMC1552030

[127] Mahfouz Z., Verloock L., Joseph W., Tanghe E., Gati A., Wiart J., Lautru D., Hanna V.F., Martens L. Comparison of temporal realistic telecommunication base station exposure with worst-case estimation in two countries. Radiat. Prot. Dosimetry 157, 331–338 (2013) 10.1093/rpd/nct155. 23771956

[128] Miclaus S., Bechet P. and Gheorghevici M. Long-term exposure to mobile communication radiation: an analysis of time-variability of electric field level in GSM900 downlink channels. Radiat. Prot. Dosimetry 154, 164–173 (2013) 10.1093/rpd/ncs169. 22908352

[129] Beekhuizen J., Vermeulen R., Kromhout H., Bürgi A. and Huss A. Geospatial modeling of electromagnetic fields from mobile phone base stations. Sci. Total Environ. 445–446, 202–209 (2013) 10.1016/j.scitotenv.2012.12.020. 23333516

[130] Troisi F., Boumis M. and Grazioso P. The Italian national electromagnetic field monitoring network. Ann. Telecomm. 63, 97–108 (2008) 10.1007/s12243-007-0011-4.

[131] Oliveira C., Sebastião D., Carpinteiro G., Correia L. M., Fernandes C. A., Serralha A., Marques N. The monIT project: electromagnetic radiation exposure assessment in mobile communications. IEEE Antennas Propag. Mag. 49, 44–53 (2007) 10.1109/MAP.2007.370981.

[132] International Telecommunication Union-Telecommunication Standardization Sector (ITU-T). Recommendation K.83-A1: Monitoring of electromagnetic field levels. Amendment 1: Updates to the Introduction and Appendix I of ITU-T K.83 2014. Available from: https://www.itu.int/rec/T-REC-K.83−201103-I. (Last accessed 18 November 2018).

[133] Plets D., Joseph W., Verloock L., Tanghe E., Martens L., Gauderis H. and Deventer E. Extensive penetration loss measurements and models for different building types for DVB-H in the UHF band. IEEE Trans. Broadcast. 55, 213–222 (2009) 10.1109/TBC.2008.2008766.

[134] Colombi D., Thors B., Persson T., Wirén N., Larsson L. E., Jonsson M. and Törnevik C. Downlink power distributions for 2G and 3G mobile communication networks. Radiat. Prot. Dosimetry 157(4), 477–487 (2013) 10.1093/rpd/nct169. 23850982

[135] Kim B. C. and Park S. O. Reduction of averaging time for evaluation of human exposure to radiofrequency electromagnetic fields from cellular base stations. IEICE Trans. Commun. E93–B, 1862–1864 (2010) 10.1587/transcom.E93.B.1862.

[136] Brussels Capital Region. Ordinance of the Brussels Capital Region of 14 March 2007: ordonnantie betreffende de bescherming van het leefmilieu tegen de schadelijke effecten en de hinder van nietioniserende stralingen (2007) (in Dutch and French).

