# Supplementary material for: APPLICATION OF TIME-AVERAGED AND INTEGRAL-BASED MEASURE FOR MEASUREMENT RESULTS VARIABILITY REDUCTION IN GSM/DCS/UMTS SYSTEMS
Source: Radiat Prot Dosimetry. 2019 Jul 12;187(2):191–214. doi: 10.1093/rpd/ncz154 (PMC7203997; doi:10.1093/rpd/ncz154)
Supplement: Supplementary_material_for_Radiation_Protection_Dosimetry_Manuscript_2019_ncz154 [file supplementary_material_for_radiation_protection_dosimetry_manuscript_2019_ncz154.zip › Supplementary material for Radiation Protection Dosimetry Manuscript 2019/Tables_2ndWeek.pdf]

Tables - Results for the 2nd week

Table 1. Summary statistic on instantaneous electric field parameters (V/m), 2nd week

Service	Day	E_{min}	E_{max}	E_{mean}	p_{50}	p_{95}	σ	ICNIRP	ER[b] (%)	AC[c] (%)
GSM (900 MHz)	1	0.033	0.241	0.063	0.055	0.116	0.022	41.3[1]	0.584	45.357
	2	0.032	0.238	0.067	0.068	0.090	0.017		0.576	55.395
	3	0.030	0.235	0.063	0.057	0.111	0.025		0.569	52.537
	4	0.029	0.224	0.059	0.056	0.084	0.017		0.542	45.780
	5	0.020	0.288	0.067	0.055	0.113	0.023		0.697	52.867
	6	0.026	0.222	0.065	0.061	0.098	0.017		0.538	49.891
	7	0.026	0.315	0.057	0.048	0.113	0.028		0.763	42.166
DCS (1800 MHz)	1	0.007	0.075	0.031	0.031	0.042	0.007	58.3[2]	0.129	10.123
	2	0.006	0.080	0.029	0.030	0.049	0.010		0.137	11.065
	3	0.009	0.092	0.034	0.035	0.051	0.011		0.158	14.457
	4	0.008	0.091	0.036	0.037	0.050	0.008		0.156	16.043
	5	0.006	0.074	0.034	0.033	0.047	0.007		0.127	12.590
	6	0.011	0.073	0.036	0.036	0.047	0.007		0.125	14.582
	7	0.008	0.092	0.031	0.029	0.049	0.011		0.158	11.127
UMTS (2100 MHz)	1	0.018	0.183	0.063	0.056	0.103	0.020	61[3]	0.300	44.520
	2	0.020	0.120	0.052	0.052	0.074	0.011		0.197	33.540
	3	0.023	0.132	0.052	0.049	0.076	0.013		0.216	33.006
	4	0.016	0.158	0.055	0.051	0.081	0.013		0.259	38.178
	5	0.027	0.109	0.056	0.053	0.079	0.011		0.179	34.543
	6	0.026	0.140	0.056	0.054	0.077	0.010		0.230	35.527
	7	0.033	0.163	0.066	0.062	0.096	0.015		0.267	46.707

[1] Regulatory exposure limit for cumulative RF-EMF exposure.

[2] Exposure Ratio = maximum field value/ICNIRP reference level.

[3] Average Contribution at L1.

Table 2. The ratio of electric field strength due to averaging (dB) for GSM downlink band, 2nd week

Service		Day	10s/30s	10s/1m	10s/3m	10s/6m	10s/15m	10s/30m	10s/1h	10s/10h	10s/24h
GSM (900 Mhz)	E_{max_10s}/E_{max_i}	1	0.155	0.562	2.492	4.650	4.820	5.048	5.263	10.63	11.111
		2	3.422	4.826	6.612	6.866	7.721	8.047	8.369	9.558	10.774
		3	0.148	1.057	3.087	5.021	5.497	5.931	6.191	10.371	10.850
		4	1.061	1.444	1.992	2.263	4.386	6.589	8.463	9.942	11.202
		5	2.209	4.487	5.551	7.290	7.805	8.252	8.736	10.753	12.214
		6	0.656	1.465	1.783	2.683	5.605	5.736	6.638	9.440	10.346
		7	1.150	1.988	2.930	3.264	4.626	6.426	7.981	11.457	13.841
	E_{min_10s}/E_{min_i}	1	-0.841	-1.325	-1.995	-2.468	-2.918	-3.170	-3.304	-4.373	-6.159
		2	-0.270	-1.100	-1.817	-2.069	-2.461	-2.606	-2.985	-4.609	-6.654
		3	-0.192	-0.333	-0.464	-1.092	-1.473	-1.920	-2.043	-4.308	-7.029
		4	-0.582	-1.018	-1.737	-1.845	-2.323	-2.976	-3.502	-4.621	-6.555
		5	-1.263	-2.000	-2.660	-5.012	-6.611	-6.940	-7.579	-8.360	-10.954
		6	-0.340	-0.913	-1.357	-2.064	-3.947	-4.674	-5.841	-7.255	-8.282
		7	-0.443	-1.074	-1.879	-2.312	-2.649	-2.703	-2.752	-3.853	-7.825
	E_{mean_10s}/E_{mean_i}	1	-0.053	-0.075	-0.102	-0.116	-0.121	-0.103	-0.041	-0.339	-0.510
		2	-0.048	-0.067	-0.084	-0.090	-0.090	-0.076	-0.046	0.347	-0.284
		3	-0.044	-0.061	-0.077	-0.082	-0.073	-0.041	0.051	0.318	-0.623
		4	-0.039	-0.056	-0.074	-0.088	-0.108	-0.122	-0.107	0.169	-0.324
		5	-0.056	-0.078	-0.104	-0.117	-0.124	-0.118	-0.086	-0.303	-0.503
		6	-0.029	-0.042	-0.057	-0.068	-0.075	-0.069	-0.049	-0.093	-0.272
		7	-0.035	-0.052	-0.077	-0.096	-0.135	-0.171	-0.232	-0.139	-0.933
	$E_{median\_10s}/E_{median\_i}$	1	-0.084	-0.107	-0.136	-0.210	-0.257	-0.329	-0.429	-1.767	-1.723
		2	-0.058	-0.114	-0.276	-0.232	-0.182	-0.255	0.006	0.768	-0.107
		3	-0.178	-0.287	-0.331	-0.398	-0.591	-0.589	-0.602	-0.640	-1.454
		4	-0.053	-0.023	-0.088	-0.018	-0.137	-0.237	-0.716	-0.144	-0.840
		5	-0.223	-0.287	-0.314	-0.318	-0.269	-0.245	-0.335	-2.628	-2.167
		6	-0.060	-0.063	-0.122	-0.148	-0.208	-0.341	-0.387	-0.644	-0.875
		7	-0.001	-0.017	-0.073	-0.174	-0.377	-0.455	-0.388	-0.461	-2.500

i – the size of running average

Table 3. The ratio of electric field strength due to averaging (dB) for DCS downlink band, 2nd week

Service		Day	10s/30s	10s/1m	10s/3m	10s/6m	10s/15m	10s/30m	10s/1h	10s/10h	10s/24h
DCS (1800 MHz)	E_{max_10s}/E_{max_i}	1	1.828	2.927	3.923	4.403	4.764	4.849	4.996	6.570	7.485
		2	2.234	2.644	2.986	3.525	3.822	3.956	4.101	7.745	8.300
		3	2.964	3.709	4.200	4.738	5.036	5.170	5.315	6.776	8.308
		4	2.832	3.261	4.275	4.610	4.788	5.508	6.142	7.164	7.931
		5	0.595	1.432	2.433	2.955	3.165	3.637	3.823	5.942	6.641
		6	1.540	1.761	2.916	3.334	3.893	4.238	4.258	5.297	6.028
		7	2.964	3.709	4.640	5.026	5.200	5.398	5.767	6.882	8.937
	E_{min_10s}/E_{min_i}	1	-6.912	-7.462	-7.607	-7.941	-8.381	-9.091	-9.257	-12.368	-13.115
		2	-1.761	-3.628	-4.297	-5.357	-6.188	-6.509	-6.952	-11.578	-14.199
		3	-0.327	-1.276	-2.238	-4.505	-5.921	-6.230	-6.503	-8.262	-11.883
		4	-5.084	-5.584	-6.706	-7.103	-7.758	-8.062	-8.279	-12.194	-13.188
		5	-11.135	-11.478	-12.064	-13.166	-13.67	-13.811	-14.115	-15.774	-16.764
		6	-3.675	-3.846	-5.315	-5.458	-5.899	-6.056	-6.489	-9.565	-10.411
		7	-1.350	-2.299	-3.261	-3.615	-4.419	-5.096	-6.425	-9.285	-12.277
	E_{mean_10s}/E_{mean_i}	1	-0.032	-0.044	-0.056	-0.063	-0.074	-0.092	-0.123	-0.665	-0.246
		2	-0.047	-0.066	-0.084	-0.090	-0.101	-0.108	-0.089	0.395	-0.499
		3	-0.028	-0.038	-0.049	-0.054	-0.062	-0.067	-0.053	-0.478	-0.465
		4	-0.041	-0.055	-0.068	-0.074	-0.081	-0.087	-0.091	-0.442	-0.231
		5	-0.045	-0.060	-0.075	-0.082	-0.089	-0.096	-0.100	-0.398	-0.203
		6	-0.025	-0.034	-0.044	-0.048	-0.054	-0.062	-0.099	-0.494	-0.141
		7	-0.033	-0.046	-0.063	-0.075	-0.095	-0.120	-0.182	-1.069	-0.531
	$E_{median\_10s}/E_{median\_i}$	1	-0.094	-0.094	-0.134	-0.125	-0.138	-0.172	-0.177	-0.797	-0.189
		2	-0.097	-0.145	-0.148	-0.181	-0.211	-0.256	-0.284	0.582	-0.220
		3	-0.083	-0.083	-0.115	-0.133	-0.182	-0.492	-0.545	-0.359	-0.087
		4	0.000	0.000	-0.026	-0.028	-0.083	-0.090	-0.149	-0.352	0.115
		5	-0.175	-0.176	-0.189	-0.206	-0.195	-0.200	-0.262	-0.820	-0.373
		6	-0.081	-0.083	-0.155	-0.191	-0.271	-0.328	-0.374	-0.715	-0.113
		7	0.000	-0.005	-0.053	-0.123	-0.170	-0.193	-0.254	-1.992	-1.091

i – the size of running average

Table 4. The ratio of electric field strength due to averaging (dB) for UMTS downlink band, 2nd week

Service		Day	10s/30s	10s/1m	10s/3m	10s/6m	10s/15m	10s/30m	10s/1h	10s/10h	10s/24h
UMTS (2100 MHz)	E_{max_10s}/E_{max_i}	1	1.546	2.286	3.132	3.606	4.293	4.415	4.617	7.156	8.800
		2	1.474	2.226	3.319	3.705	4.070	4.376	4.647	6.508	7.005
		3	0.684	1.587	2.308	3.399	3.936	4.119	4.297	6.842	7.858
		4	3.185	3.599	4.217	4.405	5.281	5.985	6.409	7.905	8.958
		5	0.551	1.282	2.763	3.148	3.466	3.780	3.981	4.828	5.622
		6	1.074	1.461	1.737	2.738	5.346	5.752	5.882	7.259	7.816
		7	0.977	1.455	2.828	3.436	4.158	4.291	4.888	6.669	7.675
	E_{min_10s}/E_{min_i}	1	-4.296	-4.576	-6.246	-7.811	-7.921	-8.174	-8.253	-9.272	-11.344
		2	-3.757	-4.568	-5.269	-5.493	-5.749	-6.201	-6.339	-7.415	-8.558
		3	-2.114	-3.100	-3.774	-3.941	-4.173	-4.337	-4.394	-5.467	-7.319
		4	-5.304	-7.753	-8.097	-8.225	-8.400	-8.515	-8.623	-9.445	-10.932
		5	-2.685	-3.143	-3.853	-3.968	-4.296	-4.558	-4.750	-5.830	-6.499
		6	-1.31	-1.747	-4.062	-4.545	-5.137	-5.337	-5.463	-6.482	-6.807
		7	-0.785	-1.79	-2.506	-3.298	-3.903	-4.057	-4.080	-5.030	-6.199
	E_{mean_10s}/E_{mean_i}	1	-0.065	-0.087	-0.109	-0.118	-0.131	-0.140	-0.149	-1.098	-0.423
		2	-0.056	-0.074	-0.090	-0.096	-0.102	-0.103	-0.099	0.142	-0.180
		3	-0.051	-0.069	-0.084	-0.091	-0.093	-0.090	-0.086	-0.269	-0.265
		4	-0.052	-0.070	-0.087	-0.096	-0.111	-0.127	-0.148	-0.300	-0.245
		5	-0.047	-0.064	-0.082	-0.092	-0.108	-0.128	-0.161	-0.495	-0.160
		6	-0.048	-0.065	-0.082	-0.092	-0.106	-0.116	-0.127	-0.396	-0.147
		7	-0.058	-0.079	-0.100	-0.111	-0.128	-0.150	-0.183	-0.539	-0.218
	$E_{median\_10s}/E_{median\_i}$	1	-0.302	-0.401	-0.514	-0.517	-0.525	-0.614	-0.561	-2.446	-1.485
		2	-0.056	-0.112	-0.149	-0.171	-0.227	-0.281	-0.288	0.036	-0.259
		3	-0.18	-0.256	-0.290	-0.287	-0.323	-0.426	-0.526	-1.007	-0.749
		4	-0.139	-0.145	-0.167	-0.250	-0.264	-0.286	-0.357	-1.090	-0.863
		5	-0.289	-0.390	-0.498	-0.564	-0.626	-0.696	-0.881	-1.141	-0.641
		6	-0.056	-0.116	-0.168	-0.158	-0.136	-0.159	-0.206	-0.827	-0.459
		7	-0.260	-0.348	-0.473	-0.506	-0.526	-0.470	-0.780	-1.428	-0.721

i – the size of running average

Table 5. Maximum to minimum ratio of electric field strength due to averaging (dB) for GSM/DCS/UMTS downlink band, 2nd week

Service		Day	10s	30s	1min	3min	6min	15min	30min	1h	10h
GSM (900 MHz)	E_{maxx_i}/E_{min_i}	1	17.270	16.274	15.382	12.783	10.152	9.532	9.052	8.702	2.268
		2	17.429	13.737	11.502	8.999	8.493	7.247	6.775	6.074	3.262
		3	17.879	17.539	16.489	14.329	11.766	10.908	10.028	9.645	3.200
		4	17.757	16.113	15.295	14.028	13.650	11.048	8.191	5.792	3.194
		5	23.167	19.696	16.680	14.956	10.865	8.751	7.976	6.852	4.054
		6	18.628	17.632	16.249	15.487	13.881	9.076	8.218	6.149	1.932
		7	21.667	20.073	18.605	16.858	16.091	14.392	12.538	10.934	6.357
DCS (1800 MHz)	E_{maxx_i}/E_{min_i}	1	20.599	11.859	10.210	9.068	8.255	7.454	6.659	6.346	1.661
		2	22.499	18.504	16.227	15.216	13.618	12.488	12.033	11.446	3.176
		3	20.191	16.900	15.206	13.753	10.948	9.233	8.791	8.373	5.153
		4	21.119	13.203	12.275	10.138	9.406	8.574	7.549	6.698	1.760
		5	23.405	11.675	10.495	8.908	7.285	6.570	5.957	5.467	1.689
		6	16.439	11.224	10.832	8.208	7.646	6.646	6.144	5.691	1.577
		7	21.214	16.900	15.206	13.313	12.574	11.595	10.719	9.022	5.047
UMTS (2100 MHz)	E_{maxx_i}/E_{min_i}	1	20.144	14.301	13.282	10.765	8.727	7.930	7.555	7.273	3.775
		2	15.563	10.332	8.768	6.975	6.365	5.744	4.986	4.577	1.640
		3	15.177	12.379	10.490	9.095	7.837	7.069	6.721	6.486	2.868
		4	19.891	11.401	8.538	7.577	7.261	6.209	5.391	4.859	2.541
		5	12.121	8.886	7.696	5.506	5.006	4.359	3.783	3.390	1.464
		6	14.623	12.239	11.415	8.824	7.341	4.140	3.534	3.279	0.883
		7	13.873	12.112	10.628	8.540	7.139	5.813	5.525	4.906	2.174

i – the size of running average

Table 6. The ratio of the standard deviation due to averaging (dB) for GSM/DCS/UMTS downlink band, 2nd week

Service		Day	10s/30s	10s/1m	10s/3min	10s/6m	10s/15m	10s/30m	10s/1h	10s/10h
GSM (900 MHz)	$\sigma_{\_10s}/\sigma_{\_i}$	1	0.456	0.666	0.960	1.181	1.666	2.317	3.643	14.944
		2	0.797	1.166	1.637	1.927	2.276	2.582	2.962	7.876
		3	0.304	0.434	0.61	0.747	0.988	1.269	1.893	12.666
		4	0.543	0.801	1.149	1.425	1.974	2.590	3.51	8.291
		5	0.487	0.710	1.006	1.194	1.458	1.719	2.102	6.691
		6	0.491	0.724	1.063	1.376	2.091	2.934	4.477	13.523
		7	0.149	0.219	0.331	0.413	0.568	0.702	0.825	4.754
DCS (1800 MHz)	$\sigma_{\_10s}/\sigma_{\_i}$	1	0.583	0.826	1.116	1.310	1.728	2.225	3.041	11.824
		2	0.416	0.597	0.815	0.932	1.071	1.192	1.490	11.112
		3	0.256	0.354	0.467	0.534	0.627	0.715	0.896	5.783
		4	0.83	1.170	1.547	1.776	2.102	2.457	2.939	10.578
		5	1.052	1.500	2.014	2.312	2.774	3.183	3.588	10.294
		6	0.848	1.191	1.581	1.790	2.121	2.435	2.906	10.635
		7	0.256	0.362	0.487	0.562	0.677	0.802	1.065	6.259
UMTS (2100 MHz)	$\sigma_{\_10s}/\sigma_{\_i}$	1	0.698	0.964	1.228	1.338	1.459	1.554	1.686	8.724
		2	1.599	2.304	3.048	3.355	3.770	4.164	4.698	13.291
		3	0.915	1.275	1.657	1.848	2.138	2.432	2.86	8.691
		4	1.002	1.384	1.759	1.918	2.133	2.342	2.588	8.626
		5	1.490	2.158	2.956	3.316	3.691	3.984	4.356	11.691
		6	1.682	2.479	3.406	3.968	4.883	5.549	6.240	14.568
		7	1.334	1.921	2.560	2.859	3.236	3.558	3.926	8.319

i – the size of running average

Table 7. Time-averaged mean E field (at 24h), 2nd week

Service	GSM	DCS	UMTS
day of the week	$E_{24hmean}$ (V/m)	$E_{24hmean}$ (V/m)	$E_{24hmean}$ (V/m)
1	0.067	0.032	0.066
2	0.069	0.031	0.054
3	0.067	0.035	0.053
4	0.062	0.037	0.056
5	0.071	0.034	0.057
6	0.067	0.036	0.057
7	0.064	0.033	0.067

Table 8. Exposed energy density (at 24h), 2nd week

Service	GSM	DCS	UMTS
day of the week	W_{24h} (J/m^2)	W_{24h} (J/m^2)	W_{24h} (J/m^2)
1	1.031	0.230	1.012
2	1.087	0.217	0.659
3	1.042	0.287	0.654
4	0.873	0.306	0.728
5	1.143	0.272	0.747
6	1.044	0.305	0.743
7	0.940	0.248	1.041
