# Supplementary material for: APPLICATION OF TIME-AVERAGED AND INTEGRAL-BASED MEASURE FOR MEASUREMENT RESULTS VARIABILITY REDUCTION IN GSM/DCS/UMTS SYSTEMS
Source: Radiat Prot Dosimetry. 2019 Jul 12;187(2):191–214. doi: 10.1093/rpd/ncz154 (PMC7203997; doi:10.1093/rpd/ncz154)
Supplement: Supplementary_material_for_Radiation_Protection_Dosimetry_Manuscript_2019_ncz154 [file supplementary_material_for_radiation_protection_dosimetry_manuscript_2019_ncz154.zip › Supplementary material for Radiation Protection Dosimetry Manuscript 2019/Tables_3rdWeek.pdf]

Tables - Results for the 3rd week

Table 1. Summary statistic on instantaneous electric field parameters (V/m), 3rd week

Service	Day	E_{min}	E_{max}	E_{mean}	p_{50}	p_{95}	σ	ICNIRP	ER[b] (%)	AC[c] (%)
GSM (900 MHz)	1	0.036	0.290	0.131	0.094	0.221	0.064	41.3[1]	0.702	88.869
	2	0.039	0.290	0.139	0.106	0.220	0.056		0.702	89.572
	3	0.040	0.330	0.127	0.099	0.249	0.063		0.799	84.679
	4	0.033	0.283	0.111	0.085	0.210	0.051		0.685	83.357
	5	0.043	0.298	0.113	0.098	0.176	0.036		0.722	83.026
	6	0.036	0.303	0.135	0.116	0.211	0.056		0.734	84.925
	7	0.056	0.301	0.136	0.103	0.214	0.049		0.729	86.457
DCS (1800 MHz)	1	0.011	0.086	0.026	0.027	0.036	0.007	58.3[2]	0.148	2.907
	2	0.017	0.077	0.031	0.029	0.042	0.006		0.132	3.956
	3	0.014	0.096	0.029	0.026	0.054	0.011		0.165	4.011
	4	0.013	0.096	0.031	0.030	0.045	0.008		0.165	5.652
	5	0.015	0.083	0.028	0.027	0.038	0.005		0.142	4.832
	6	0.006	0.096	0.033	0.028	0.057	0.012		0.165	4.771
	7	0.014	0.084	0.029	0.026	0.042	0.008		0.144	3.626
UMTS (2100 MHz)	1	0.018	0.120	0.042	0.039	0.065	0.013	61[3]	0.197	8.224
	2	0.014	0.123	0.038	0.035	0.062	0.012		0.202	6.473
	3	0.018	0.156	0.049	0.041	0.083	0.017		0.256	11.310
	4	0.020	0.139	0.041	0.038	0.071	0.015		0.228	10.991
	5	0.020	0.119	0.043	0.039	0.069	0.014		0.195	12.142
	6	0.013	0.148	0.047	0.038	0.085	0.020		0.243	10.304
	7	0.020	0.139	0.047	0.042	0.074	0.014		0.228	9.916

[1] Regulatory exposure limit for cumulative RF-EMF exposure.

[2] Exposure Ratio = maximum field value/ICNIRP reference level.

[3] Average Contribution at L1.

Table 2. The ratio of electric field strength due to averaging (dB) for GSM downlink band, 3rd week

Service		Day	10s/30s	10s/1m	10s/3m	10s/6m	10s/15m	10s/30m	10s/1h	10s/10h	10s/24h
GSM (900 Mhz)	E_{max_10s}/E_{max_i}	1	0.841	1.427	1.854	2.122	2.351	2.440	2.644	3.223	5.967
		2	0.841	1.427	1.854	2.122	2.351	2.440	2.644	3.223	5.749
		3	0.384	0.938	1.319	1.758	2.098	2.216	2.350	4.728	7.301
		4	0.677	0.929	1.274	1.581	1.700	1.988	2.584	4.89	7.331
		5	0.791	1.248	1.424	1.627	1.986	2.252	3.425	6.433	8.019
		6	1.162	1.489	2.035	2.388	2.827	3.162	3.354	3.895	6.326
		7	1.081	1.554	2.177	2.508	2.664	2.870	3.172	4.128	6.358
	E_{min_10s}/E_{min_i}	1	-2.321	-2.412	-3.294	-4.014	-4.249	-5.007	-5.707	-6.129	-12.155
		2	-2.180	-2.509	-3.376	-3.857	-4.400	-4.762	-4.985	-7.504	-11.677
		3	-2.884	-3.506	-3.889	-4.131	-4.523	-4.760	-5.194	-6.762	-11.028
		4	-4.619	-4.663	-5.555	-6.029	-6.682	-6.787	-6.818	-7.421	-11.335
		5	-1.154	-1.546	-2.130	-2.511	-3.011	-3.345	-3.940	-7.020	-8.796
		6	-1.089	-1.508	-3.096	-3.241	-4.180	-4.652	-5.634	-7.548	-12.177
		7	-0.206	-0.885	-1.499	-1.767	-2.157	-2.235	-4.318	-4.726	-8.250
	E_{mean_10s}/E_{mean_i}	1	-0.014	-0.020	-0.024	-0.024	-0.023	-0.038	-0.014	0.237	-0.935
		2	-0.014	-0.020	-0.028	-0.035	-0.045	-0.041	-0.018	0.193	-0.668
		3	-0.020	-0.029	-0.042	-0.057	-0.091	-0.140	-0.231	-0.572	-0.96
		4	-0.020	-0.029	-0.047	-0.065	-0.107	-0.159	-0.262	0.017	-0.827
		5	-0.013	-0.020	-0.032	-0.046	-0.087	-0.147	-0.254	-0.313	-0.420
		6	-0.012	-0.017	-0.026	-0.033	-0.049	-0.065	-0.124	-0.590	-0.684
		7	-0.009	-0.014	-0.021	-0.027	-0.040	-0.058	-0.080	0.014	-0.537
	$E_{median\_10s}/E_{median\_i}$	1	0.090	0.079	0.082	0.107	-0.388	-1.033	-0.562	-2.420	-3.818
		2	0.053	0.065	0.036	0.057	0.067	0.085	0.175	-1.784	-2.992
		3	0.000	0.004	0.004	0.013	0.018	-0.011	-0.045	-2.544	-3.156
		4	-0.011	-0.039	-0.091	-0.108	-0.336	-0.640	-1.031	-1.601	-3.117
		5	0.029	0.029	0.018	0.012	-0.004	-0.054	-0.234	-1.573	-1.640
		6	-0.052	-0.050	-0.087	-0.151	-0.165	0.035	-0.428	-2.168	-2.014
		7	-0.029	-0.017	-0.031	-0.053	-0.157	-0.681	-1.592	-2.186	-2.957

i – the size of running average

Table 3. The ratio of electric field strength due to averaging (dB) for DCS downlink band, 3rd week

Service		Day	10s/30s	10s/1m	10s/3m	10s/6m	10s/15m	10s/30m	10s/1h	10s/10h	10s/24h
DCS (1800 MHz)	E_{max_10s}/E_{max_i}	1	2.791	4.668	5.830	6.273	6.342	6.800	7.419	9.188	10.262
		2	1.512	1.894	2.097	2.912	4.493	4.964	5.202	7.187	7.782
		3	1.122	1.379	2.203	2.527	2.831	3.437	4.903	7.798	9.822
		4	1.675	3.584	3.821	3.887	4.081	4.370	6.535	8.695	9.628
		5	2.637	4.049	4.909	5.377	5.590	5.825	6.916	8.828	9.268
		6	1.049	1.972	2.154	2.215	2.493	2.961	3.467	7.470	8.846
		7	2.541	3.290	4.605	5.102	5.403	5.746	6.247	7.914	9.046
	E_{min_10s}/E_{min_i}	1	-2.304	-2.503	-2.952	-3.152	-3.479	-3.807	-4.118	-5.235	-7.600
		2	-1.268	-1.492	-1.662	-1.827	-2.320	-3.638	-3.933	-4.690	-5.339
		3	-1.518	-1.686	-1.979	-2.077	-2.130	-2.179	-2.389	-3.554	-6.900
		4	-2.667	-2.831	-3.151	-3.481	-4.808	-5.041	-5.241	-6.643	-7.739
		5	-0.382	-1.584	-1.878	-2.260	-2.639	-2.894	-3.268	-5.159	-5.591
		6	-8.519	-8.702	-9.056	-9.368	-9.837	-10.279	-10.459	-12.375	-15.236
		7	-3.098	-3.098	-3.317	-3.391	-3.747	-3.873	-4.033	-4.335	-6.517
	E_{mean_10s}/E_{mean_i}	1	-0.036	-0.052	-0.068	-0.074	-0.079	-0.088	-0.089	-0.442	-0.288
		2	-0.024	-0.032	-0.038	-0.036	-0.031	-0.038	-0.036	-0.344	-0.151
		3	-0.060	-0.084	-0.109	-0.118	-0.123	-0.116	-0.085	0.083	-0.635
		4	-0.025	-0.038	-0.058	-0.073	-0.103	-0.140	-0.189	-0.634	-0.270
		5	-0.032	-0.044	-0.055	-0.059	-0.060	-0.052	-0.041	-0.166	-0.155
		6	-0.025	-0.036	-0.050	-0.057	-0.062	-0.057	-0.049	0.011	-0.538
		7	-0.038	-0.054	-0.072	-0.080	-0.087	-0.090	-0.081	-0.220	-0.297
	$E_{median\_10s}/E_{median\_i}$	1	-0.213	-0.217	-0.302	-0.318	-0.400	-0.412	-0.505	-0.330	0.199
		2	-0.100	-0.100	-0.150	-0.182	-0.167	-0.177	-0.147	-1.089	-0.700
		3	-0.340	-0.400	-0.402	-0.522	-0.670	-0.730	-0.851	-0.693	-1.524
		4	-0.078	-0.049	-0.101	-0.098	-0.133	-0.203	-0.237	-0.984	-0.475
		5	-0.213	-0.214	-0.236	-0.224	-0.218	-0.160	-0.231	-0.458	-0.486
		6	-0.188	-0.223	-0.232	-0.408	-0.332	-0.285	-0.292	-1.192	-1.856
		7	-0.114	-0.180	-0.288	-0.358	-0.527	-0.542	-0.806	-1.220	-1.140

i – the size of running average

Table 4. The ratio of electric field strength due to averaging (dB) for UMTS downlink band, 3rd week

Service		Day	10s/30s	10s/1m	10s/3m	10s/6m	10s/15m	10s/30m	10s/1h	10s/10h	10s/24h
UMTS (2100 MHz)	E_{max_10s}/E_{max_i}	1	2.691	2.972	3.818	4.310	5.011	5.240	5.442	6.779	8.639
		2	1.379	2.394	3.440	3.840	4.686	4.751	5.132	8.449	9.711
		3	1.234	2.423	4.139	4.609	5.168	5.690	5.961	7.620	9.537
		4	2.313	3.033	3.721	4.297	5.084	5.438	5.793	8.025	9.955
		5	1.567	2.112	2.902	3.561	4.049	4.229	4.883	6.573	8.395
		6	1.431	2.677	3.347	3.773	4.203	4.348	4.457	6.827	9.262
		7	2.313	3.033	3.721	4.297	5.084	5.438	5.793	7.982	9.051
	E_{min_10s}/E_{min_i}	1	-1.619	-2.125	-3.683	-3.913	-4.009	-4.027	-4.093	-5.004	-7.839
		2	-0.807	-2.971	-4.483	-6.058	-6.281	-6.324	-6.338	-7.173	-9.165
		3	-0.772	-1.065	-1.299	-1.586	-2.701	-4.153	-6.277	-6.721	-9.220
		4	-1.589	-1.823	-2.084	-2.437	-2.711	-2.890	-2.976	-3.552	-6.885
		5	-2.064	-2.243	-2.874	-2.938	-3.092	-3.497	-3.604	-4.484	-7.095
		6	-1.450	-3.615	-5.127	-5.937	-6.925	-6.968	-6.981	-7.689	-11.864
		7	-1.828	-2.130	-2.385	-2.634	-3.151	-3.712	-4.104	-5.864	-7.788
	E_{mean_10s}/E_{mean_i}	1	-0.051	-0.069	-0.083	-0.086	-0.088	-0.088	-0.078	0.037	-0.385
		2	-0.056	-0.075	-0.092	-0.095	-0.092	-0.077	-0.056	-0.375	-0.402
		3	-0.076	-0.103	-0.129	-0.140	-0.159	-0.189	-0.237	-0.383	-0.513
		4	-0.064	-0.085	-0.107	-0.117	-0.131	-0.136	-0.136	0.363	-0.548
		5	-0.060	-0.080	-0.100	-0.109	-0.128	-0.147	-0.164	-0.125	-0.413
		6	-0.057	-0.077	-0.096	-0.104	-0.109	-0.115	-0.156	-0.335	-0.742
		7	-0.060	-0.081	-0.103	-0.115	-0.134	-0.154	-0.198	-0.325	-0.353
	$E_{median\_10s}/E_{median\_i}$	1	-0.215	-0.236	-0.256	-0.199	-0.137	-0.352	-0.464	-0.569	-1.123
		2	-0.102	-0.098	-0.068	-0.100	-0.056	-0.082	-0.064	-1.793	-1.206
		3	-0.457	-0.517	-0.524	-0.517	-0.563	-0.570	-0.613	-1.894	-2.070
		4	0.063	0.099	0.077	0.010	-0.096	-0.191	-0.203	-0.001	-1.310
		5	-0.203	-0.233	-0.284	-0.353	-0.282	-0.242	-0.136	-0.962	-1.294
		6	0.100	0.206	0.248	0.253	0.238	0.307	0.070	-1.988	-2.547
		7	-0.284	-0.323	-0.386	-0.434	-0.507	-0.526	-0.582	-1.399	-1.344

i – the size of running average

Table 5. Maximum to minimum ratio of electric field strength due to averaging (dB) for GSM/DCS/UMTS downlink band, 3rd week

Service		Day	10s	30s	1min	3min	6min	15min	30min	1h	10h
GSM (900 MHz)	E_{maxx_i}/E_{min_i}	1	18.122	14.959	14.283	12.975	11.987	11.523	10.675	9.771	8.770
		2	17.427	14.405	13.491	12.197	11.448	10.676	10.224	9.797	6.700
		3	18.329	15.061	13.886	13.121	12.440	11.708	11.354	10.786	6.839
		4	18.665	13.369	13.074	11.836	11.056	10.283	9.891	9.263	6.355
		5	16.815	14.870	14.021	13.261	12.677	11.818	11.218	9.450	3.361
		6	18.503	16.252	15.505	13.371	12.874	11.496	10.689	9.514	7.060
		7	14.608	13.320	12.168	10.932	10.333	9.787	9.502	7.117	5.753
DCS (1800 MHz)	E_{maxx_i}/E_{min_i}	1	17.862	12.767	10.691	9.080	8.437	8.041	7.255	6.325	3.439
		2	13.121	10.341	9.735	9.362	8.382	6.308	4.519	3.986	1.244
		3	16.723	14.083	13.657	12.541	12.119	11.761	11.107	9.430	5.371
		4	17.367	13.025	10.951	10.394	9.999	8.477	7.955	5.591	2.029
		5	14.860	11.841	9.227	8.073	7.223	6.631	6.140	4.676	0.873
		6	24.082	14.514	13.408	12.873	12.500	11.752	10.842	10.156	4.238
		7	15.563	9.924	9.175	7.641	7.070	6.413	5.945	5.283	3.314
UMTS (2100 MHz)	E_{maxx_i}/E_{min_i}	1	16.478	12.169	11.381	8.978	8.255	7.458	7.212	6.942	4.696
		2	18.876	16.690	13.511	10.953	8.978	7.908	7.801	7.405	3.254
		3	18.757	16.752	15.270	13.319	12.562	10.888	8.914	6.520	4.416
		4	16.840	12.938	11.983	11.034	10.106	9.046	8.512	8.070	5.262
		5	15.490	11.859	11.135	9.714	8.991	8.350	7.764	7.003	4.433
		6	21.126	18.245	14.834	12.653	11.416	9.998	9.810	9.689	6.610
		7	16.840	12.699	11.677	10.733	9.909	8.605	7.689	6.943	2.954

i – the size of running average

Table 6. The ratio of the standard deviation due to averaging (dB) for GSM/DCS/UMTS downlink band, 3rd week

Service		Day	10s/30s	10s/1m	10s/3min	10s/6m	10s/15m	10s/30m	10s/1h	10s/10h
GSM (900 MHz)	$\sigma_{\_10s}/\sigma_{\_i}$	1	0.062	0.091	0.134	0.176	0.287	0.435	0.589	3.282
		2	0.082	0.115	0.167	0.217	0.329	0.481	0.663	3.684
		3	0.080	0.109	0.149	0.179	0.236	0.304	0.421	3.758
		4	0.092	0.132	0.202	0.271	0.426	0.630	0.966	4.579
		5	0.130	0.187	0.285	0.396	0.703	1.178	2.326	7.501
		6	0.072	0.105	0.163	0.214	0.336	0.480	0.644	3.400
		7	0.070	0.101	0.155	0.210	0.363	0.620	1.075	4.496
DCS (1800 MHz)	$\sigma_{\_10s}/\sigma_{\_i}$	1	0.577	0.859	1.215	1.439	1.760	1.990	2.320	7.272
		2	0.778	1.160	1.717	2.210	2.926	3.463	3.982	12.144
		3	0.409	0.594	0.834	0.975	1.241	1.592	2.210	6.205
		4	0.401	0.620	0.968	1.234	1.699	2.221	3.141	12.032
		5	0.994	1.474	2.076	2.426	3.048	3.846	5.424	14.992
		6	0.194	0.288	0.424	0.508	0.640	0.751	0.879	9.359
		7	0.578	0.867	1.209	1.404	1.679	1.937	2.379	7.013
UMTS (2100 MHz)	$\sigma_{\_10s}/\sigma_{\_i}$	1	0.594	0.819	1.020	1.095	1.172	1.237	1.302	3.820
		2	0.624	0.866	1.114	1.221	1.416	1.694	2.158	6.309
		3	0.654	0.916	1.171	1.268	1.425	1.619	1.839	4.644
		4	0.508	0.698	0.903	1.014	1.167	1.285	1.399	5.137
		5	0.651	0.901	1.162	1.297	1.503	1.690	1.906	4.982
		6	0.321	0.441	0.571	0.633	0.722	0.793	0.851	3.709
		7	0.776	1.079	1.404	1.568	1.774	1.958	2.317	8.881

i – the size of running average

Table 7. Time-averaged mean E field (at 24h), 3rd week

Service	GSM	DCS	UMTS
day of the week	$E_{24hmean}$ (V/m)	$E_{24hmean}$ (V/m)	$E_{24hmean}$ (V/m)
1	0.146	0.026	0.044
2	0.150	0.031	0.040
3	0.142	0.031	0.052
4	0.122	0.032	0.044
5	0.118	0.029	0.045
6	0.146	0.034	0.050
7	0.145	0.030	0.049

Table 8. Exposed energy density (at 24h), 3rd week

Service	GSM	DCS	UMTS
day of the week	W_{24h} (J/m^2)	W_{24h} (J/m^2)	W_{24h} (J/m^2)
1	4.882	0.160	0.452
2	5.132	0.227	0.371
3	4.649	0.220	0.621
4	3.396	0.230	0.448
5	3.213	0.187	0.470
6	4.907	0.276	0.595
7	4.806	0.202	0.551
