# Supplementary figures and images for: APPLICATION OF TIME-AVERAGED AND INTEGRAL-BASED MEASURE FOR MEASUREMENT RESULTS VARIABILITY REDUCTION IN GSM/DCS/UMTS SYSTEMS (part 2 of 2)
Source: Radiat Prot Dosimetry. 2019 Jul 12;187(2):191–214. doi: 10.1093/rpd/ncz154 (PMC7203997; doi:10.1093/rpd/ncz154)

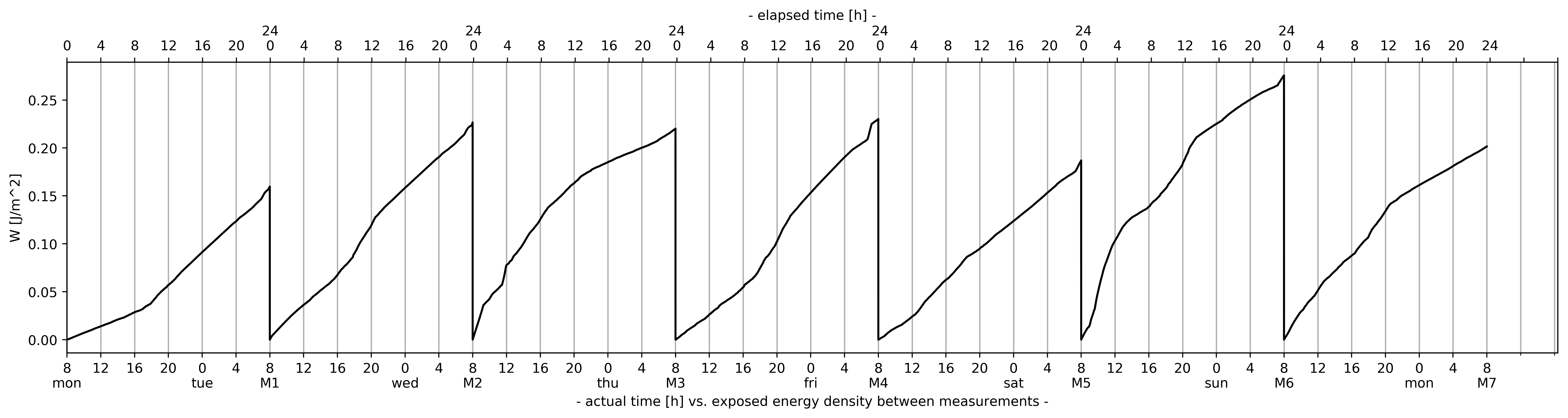

Supplement: Supplementary_material_for_Radiation_Protection_Dosimetry_Manuscript_2019_ncz154 [file supplementary_material_for_radiation_protection_dosimetry_manuscript_2019_ncz154.zip › Supplementary material for Radiation Protection Dosimetry Manuscript 2019/Location1_Figures_3rdWeek/Figure6_DCS_3rdWeek.jpg]

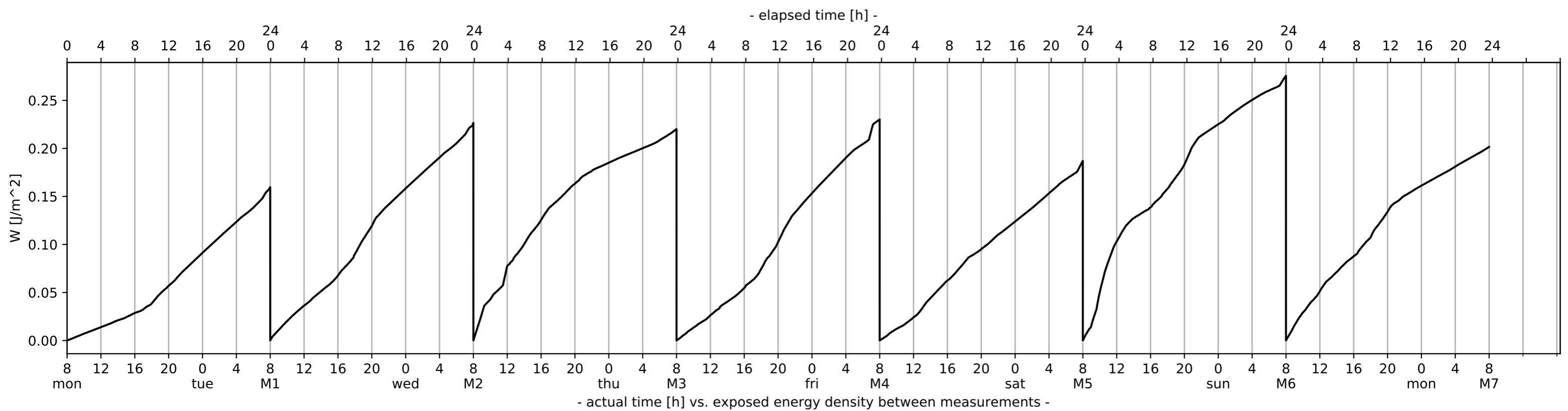

Supplement: Supplementary_material_for_Radiation_Protection_Dosimetry_Manuscript_2019_ncz154 [file supplementary_material_for_radiation_protection_dosimetry_manuscript_2019_ncz154.zip › Supplementary material for Radiation Protection Dosimetry Manuscript 2019/Location1_Figures_3rdWeek/Figure6_DCS_3rdWeek.pdf]

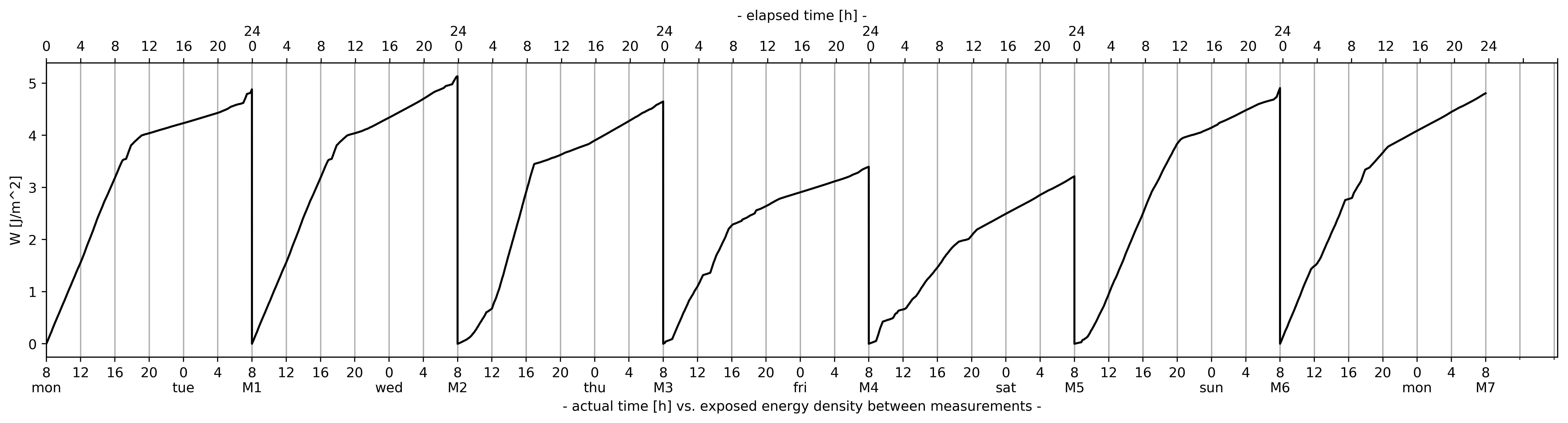

Supplement: Supplementary_material_for_Radiation_Protection_Dosimetry_Manuscript_2019_ncz154 [file supplementary_material_for_radiation_protection_dosimetry_manuscript_2019_ncz154.zip › Supplementary material for Radiation Protection Dosimetry Manuscript 2019/Location1_Figures_3rdWeek/Figure6_GSM_3rdWeek.jpg]

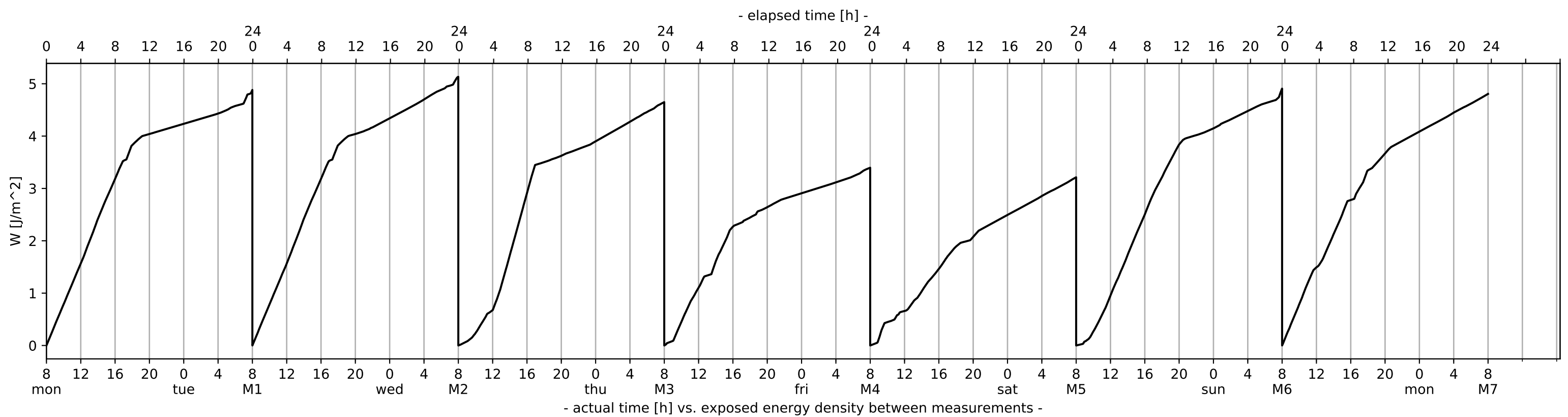

Supplement: Supplementary_material_for_Radiation_Protection_Dosimetry_Manuscript_2019_ncz154 [file supplementary_material_for_radiation_protection_dosimetry_manuscript_2019_ncz154.zip › Supplementary material for Radiation Protection Dosimetry Manuscript 2019/Location1_Figures_3rdWeek/Figure6_GSM_3rdWeek.pdf]

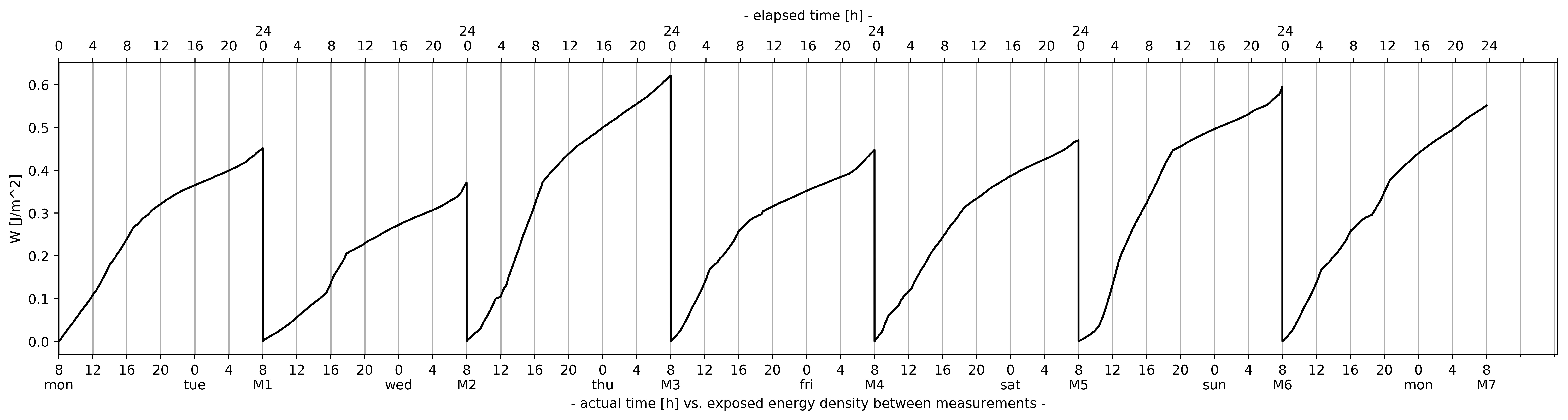

Supplement: Supplementary_material_for_Radiation_Protection_Dosimetry_Manuscript_2019_ncz154 [file supplementary_material_for_radiation_protection_dosimetry_manuscript_2019_ncz154.zip › Supplementary material for Radiation Protection Dosimetry Manuscript 2019/Location1_Figures_3rdWeek/Figure6_UMTS_3rdWeek.jpg]

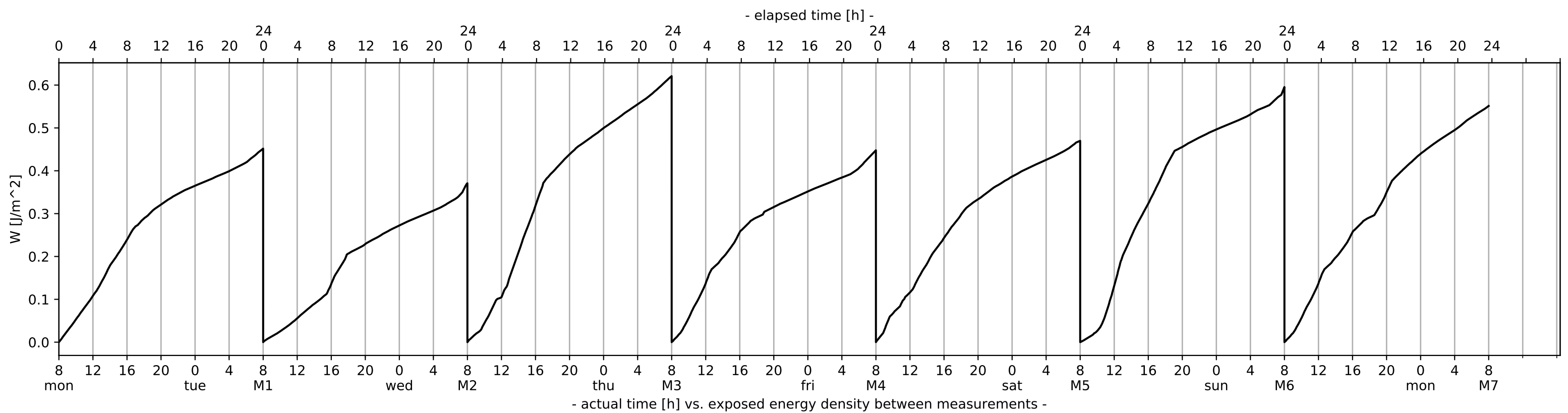

Supplement: Supplementary_material_for_Radiation_Protection_Dosimetry_Manuscript_2019_ncz154 [file supplementary_material_for_radiation_protection_dosimetry_manuscript_2019_ncz154.zip › Supplementary material for Radiation Protection Dosimetry Manuscript 2019/Location1_Figures_3rdWeek/Figure6_UMTS_3rdWeek.pdf]

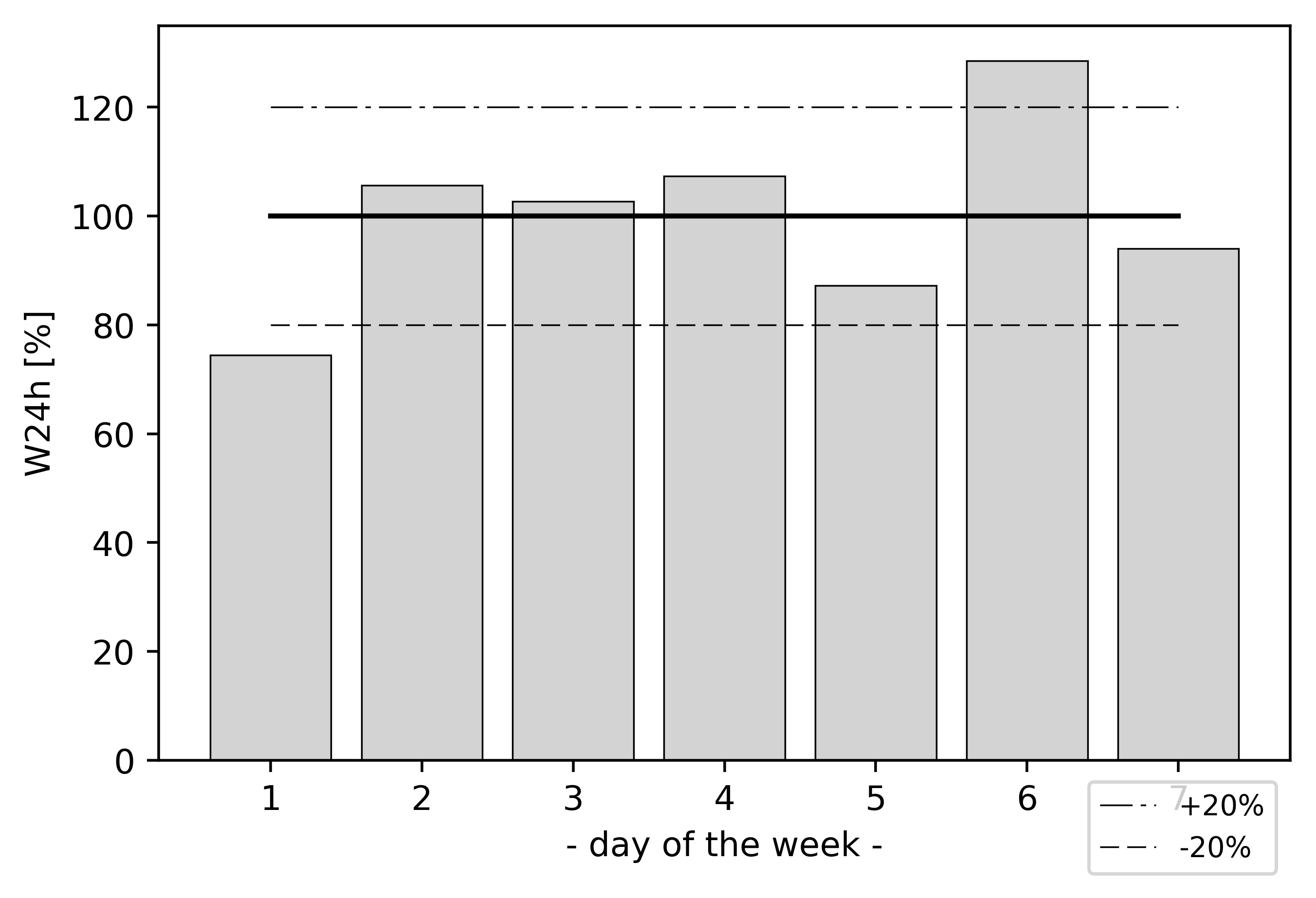

Supplement: Supplementary_material_for_Radiation_Protection_Dosimetry_Manuscript_2019_ncz154 [file supplementary_material_for_radiation_protection_dosimetry_manuscript_2019_ncz154.zip › Supplementary material for Radiation Protection Dosimetry Manuscript 2019/Location1_Figures_3rdWeek/Figure7_DCS_3rdWeek.jpg]

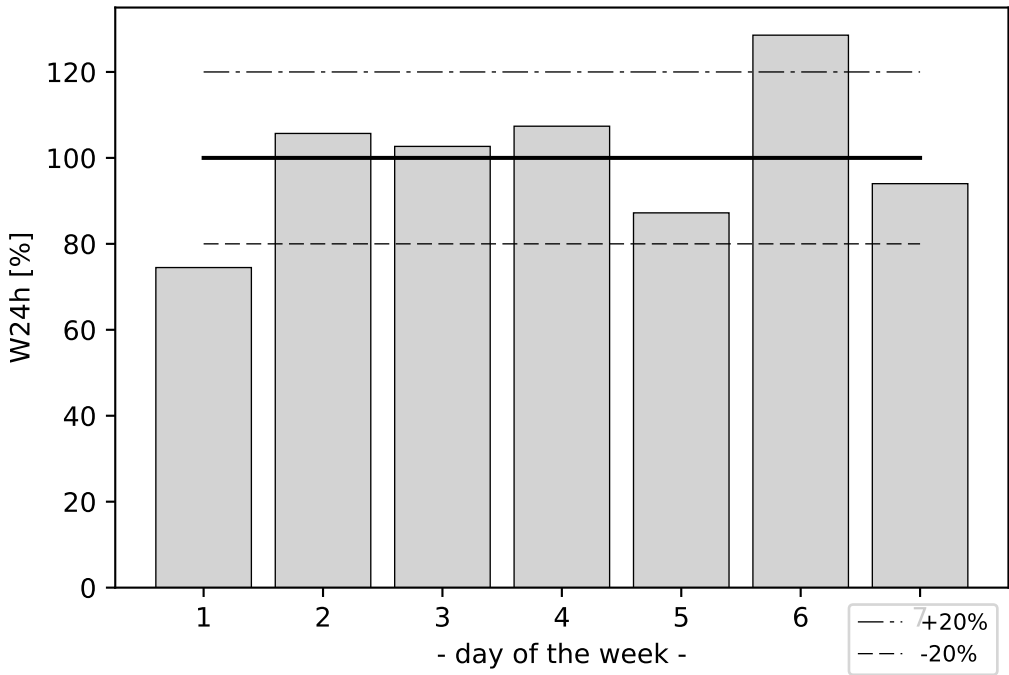

Supplement: Supplementary_material_for_Radiation_Protection_Dosimetry_Manuscript_2019_ncz154 [file supplementary_material_for_radiation_protection_dosimetry_manuscript_2019_ncz154.zip › Supplementary material for Radiation Protection Dosimetry Manuscript 2019/Location1_Figures_3rdWeek/Figure7_DCS_3rdWeek.pdf]

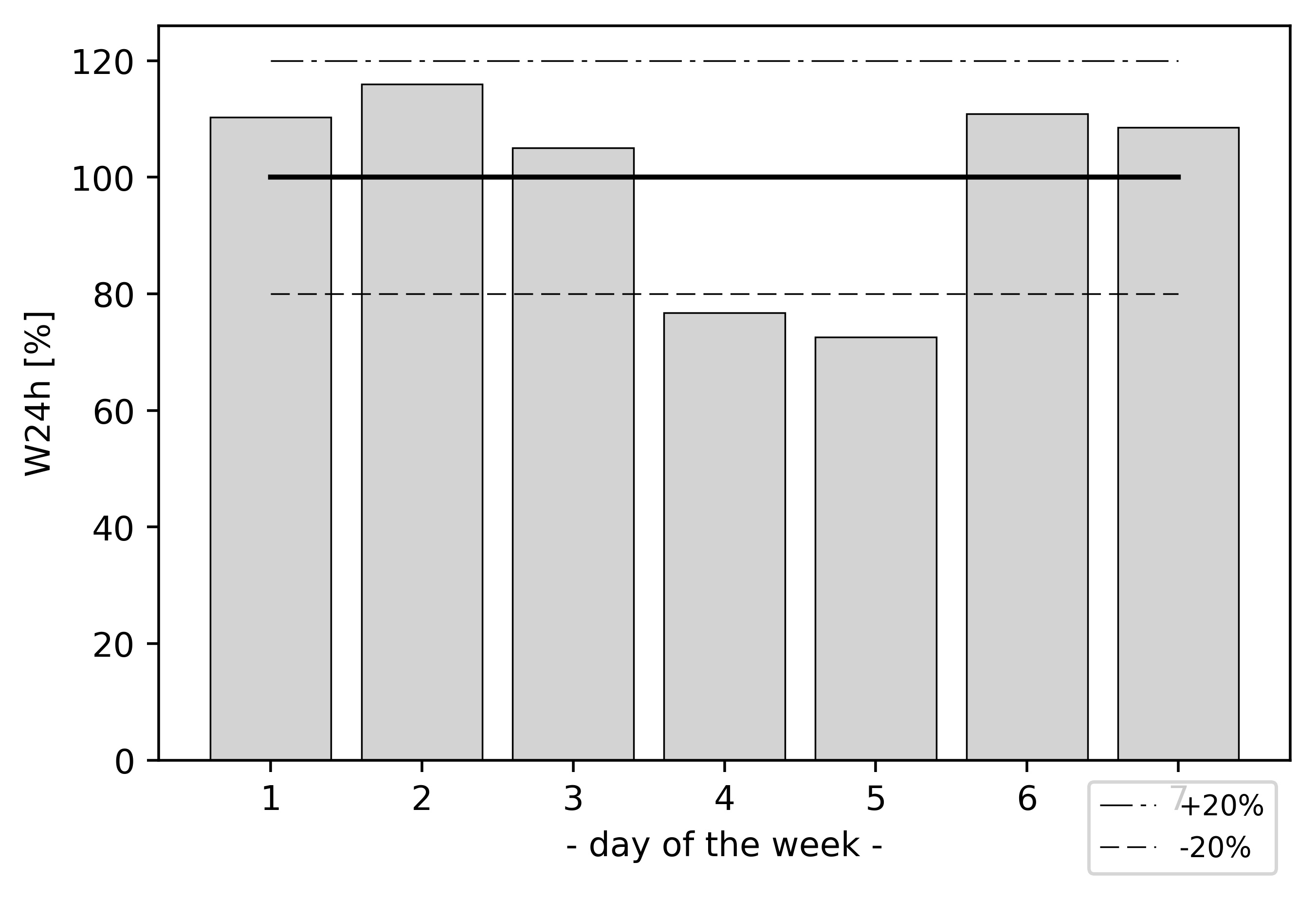

Supplement: Supplementary_material_for_Radiation_Protection_Dosimetry_Manuscript_2019_ncz154 [file supplementary_material_for_radiation_protection_dosimetry_manuscript_2019_ncz154.zip › Supplementary material for Radiation Protection Dosimetry Manuscript 2019/Location1_Figures_3rdWeek/Figure7_GSM_3rdWeek.jpg]

W24h [%]

120

100

80

60

40

20

0

1

2

3

4

5

6

7

- day of the week -

— ··· +20%  
- - - -20%

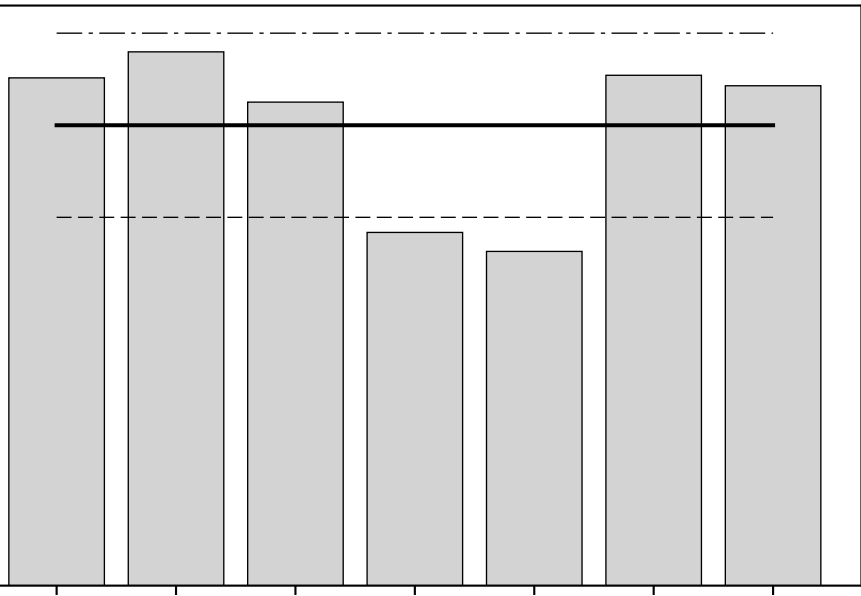

Supplement: Supplementary_material_for_Radiation_Protection_Dosimetry_Manuscript_2019_ncz154 [file supplementary_material_for_radiation_protection_dosimetry_manuscript_2019_ncz154.zip › Supplementary material for Radiation Protection Dosimetry Manuscript 2019/Location1_Figures_3rdWeek/Figure7_GSM_3rdWeek.pdf]

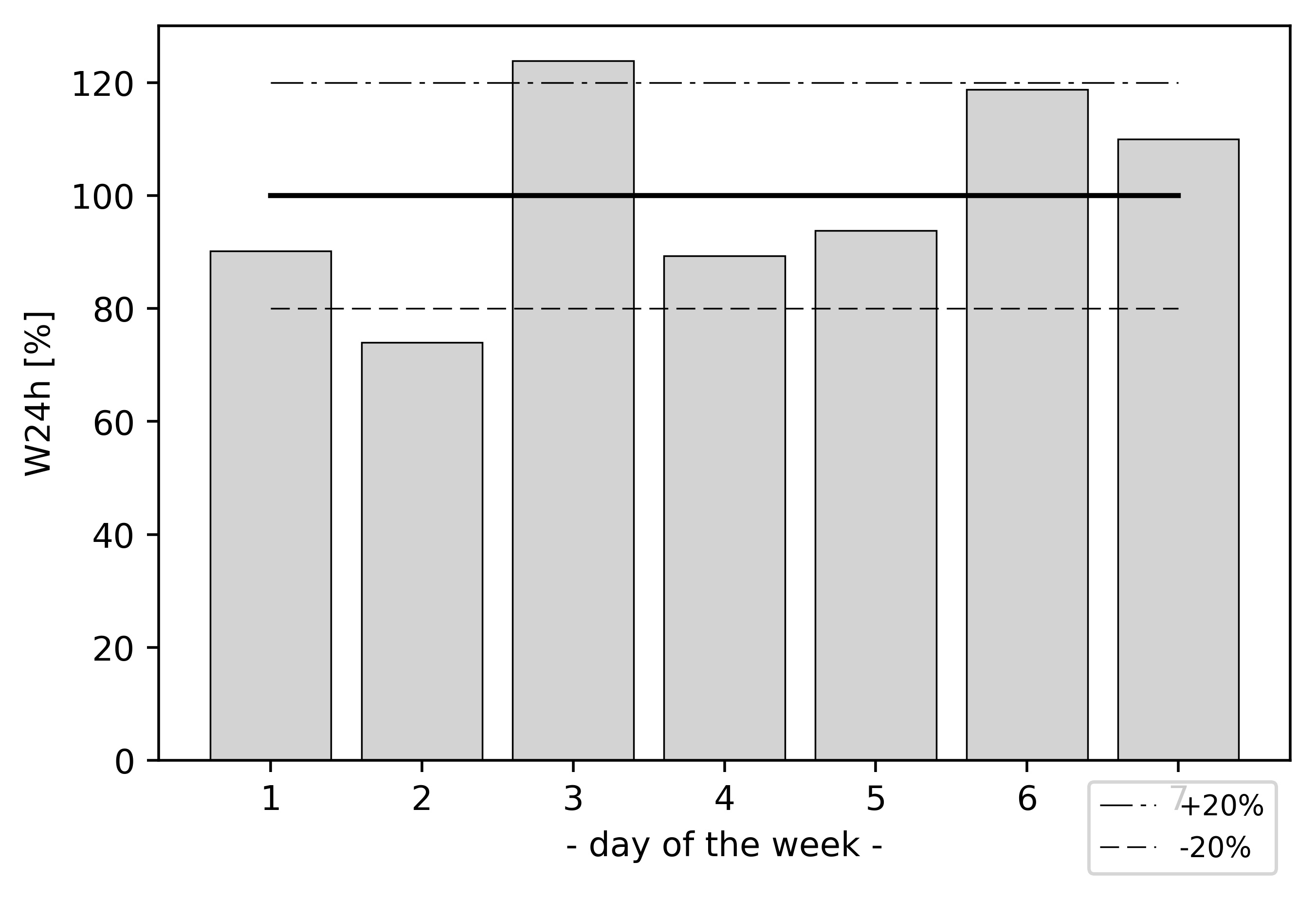

Supplement: Supplementary_material_for_Radiation_Protection_Dosimetry_Manuscript_2019_ncz154 [file supplementary_material_for_radiation_protection_dosimetry_manuscript_2019_ncz154.zip › Supplementary material for Radiation Protection Dosimetry Manuscript 2019/Location1_Figures_3rdWeek/Figure7_UMTS_3rdWeek.jpg]

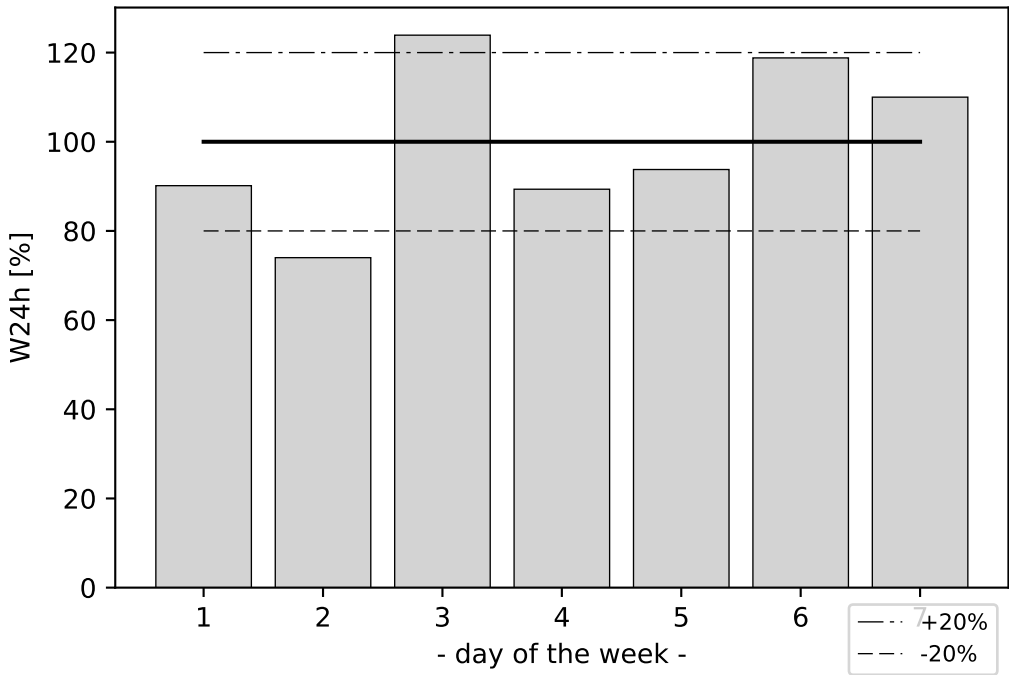

Supplement: Supplementary_material_for_Radiation_Protection_Dosimetry_Manuscript_2019_ncz154 [file supplementary_material_for_radiation_protection_dosimetry_manuscript_2019_ncz154.zip › Supplementary material for Radiation Protection Dosimetry Manuscript 2019/Location1_Figures_3rdWeek/Figure7_UMTS_3rdWeek.pdf]
