# Supplementary figures and images for: APPLICATION OF TIME-AVERAGED AND INTEGRAL-BASED MEASURE FOR MEASUREMENT RESULTS VARIABILITY REDUCTION IN GSM/DCS/UMTS SYSTEMS (part 1 of 2)
Source: Radiat Prot Dosimetry. 2019 Jul 12;187(2):191–214. doi: 10.1093/rpd/ncz154 (PMC7203997; doi:10.1093/rpd/ncz154)

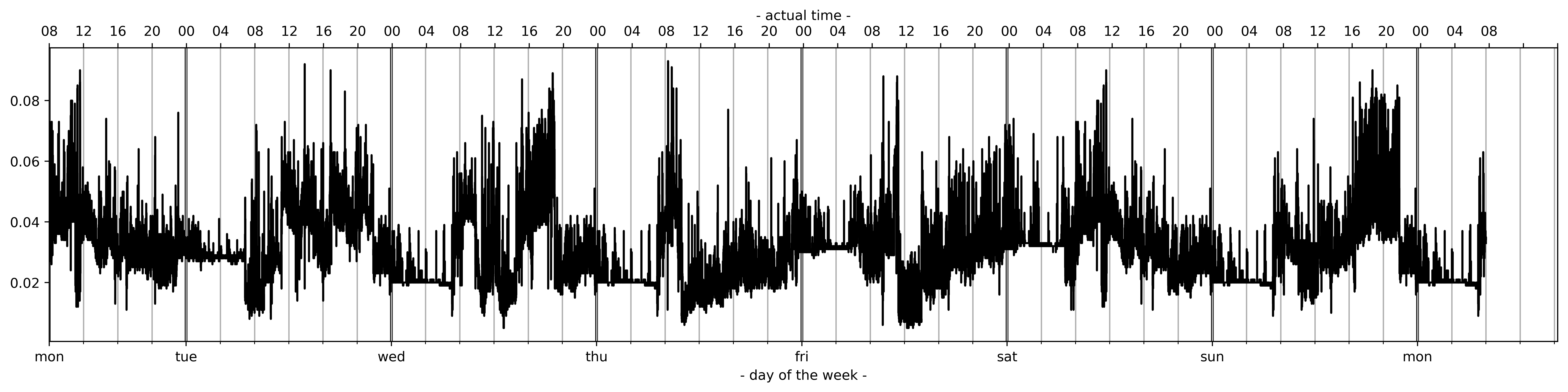

Supplement: Supplementary_material_for_Radiation_Protection_Dosimetry_Manuscript_2019_ncz154 [file supplementary_material_for_radiation_protection_dosimetry_manuscript_2019_ncz154.zip › Supplementary material for Radiation Protection Dosimetry Manuscript 2019/Location1_Figures_1stWeek/Figure1_DCS_1stWeek.jpg]

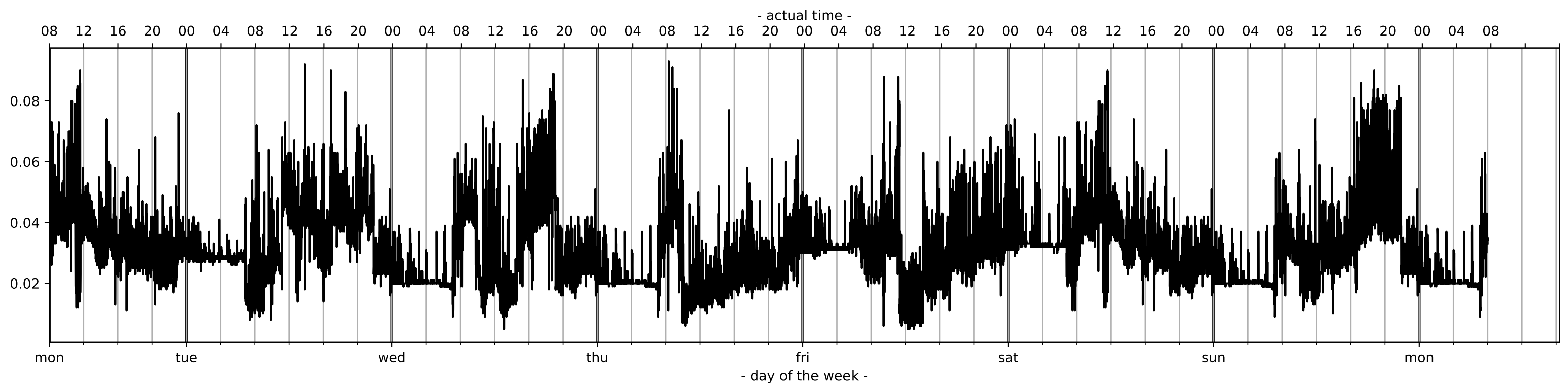

Supplement: Supplementary_material_for_Radiation_Protection_Dosimetry_Manuscript_2019_ncz154 [file supplementary_material_for_radiation_protection_dosimetry_manuscript_2019_ncz154.zip › Supplementary material for Radiation Protection Dosimetry Manuscript 2019/Location1_Figures_1stWeek/Figure1_DCS_1stWeek.pdf]

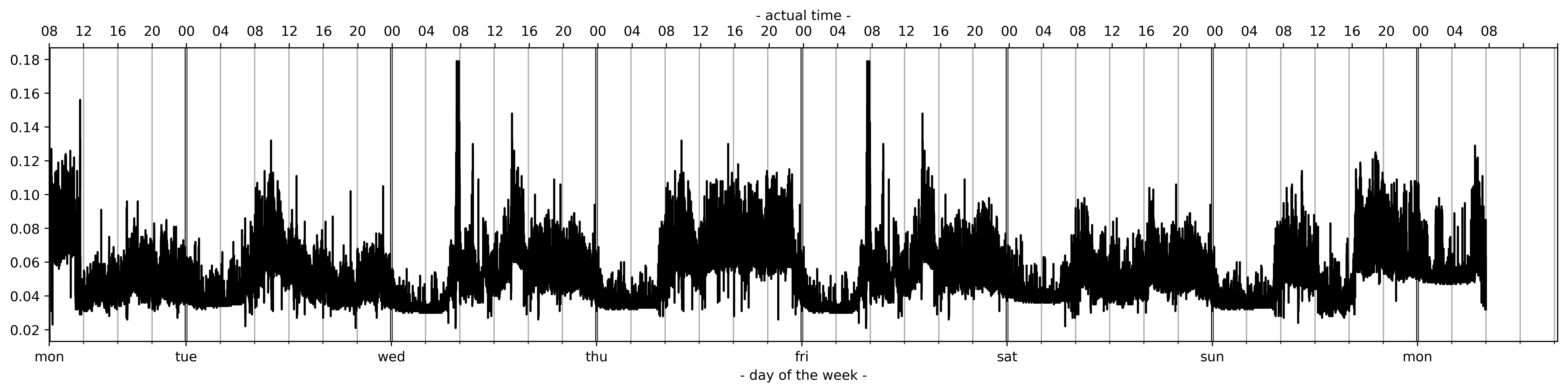

Supplement: Supplementary_material_for_Radiation_Protection_Dosimetry_Manuscript_2019_ncz154 [file supplementary_material_for_radiation_protection_dosimetry_manuscript_2019_ncz154.zip › Supplementary material for Radiation Protection Dosimetry Manuscript 2019/Location1_Figures_1stWeek/Figure1_UMTS_1stWeek.jpg]

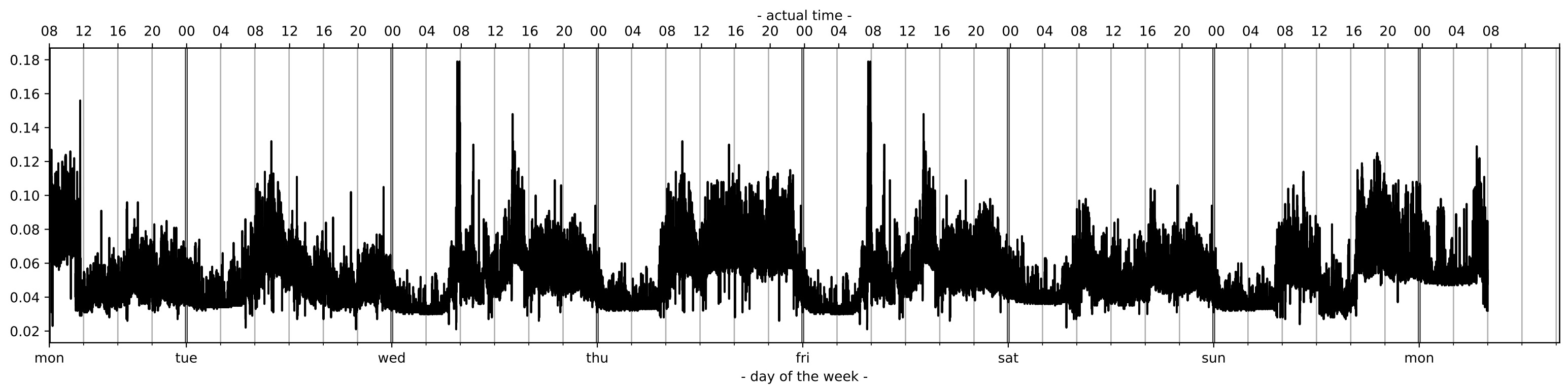

Supplement: Supplementary_material_for_Radiation_Protection_Dosimetry_Manuscript_2019_ncz154 [file supplementary_material_for_radiation_protection_dosimetry_manuscript_2019_ncz154.zip › Supplementary material for Radiation Protection Dosimetry Manuscript 2019/Location1_Figures_1stWeek/Figure1_UMTS_1stWeek.pdf]

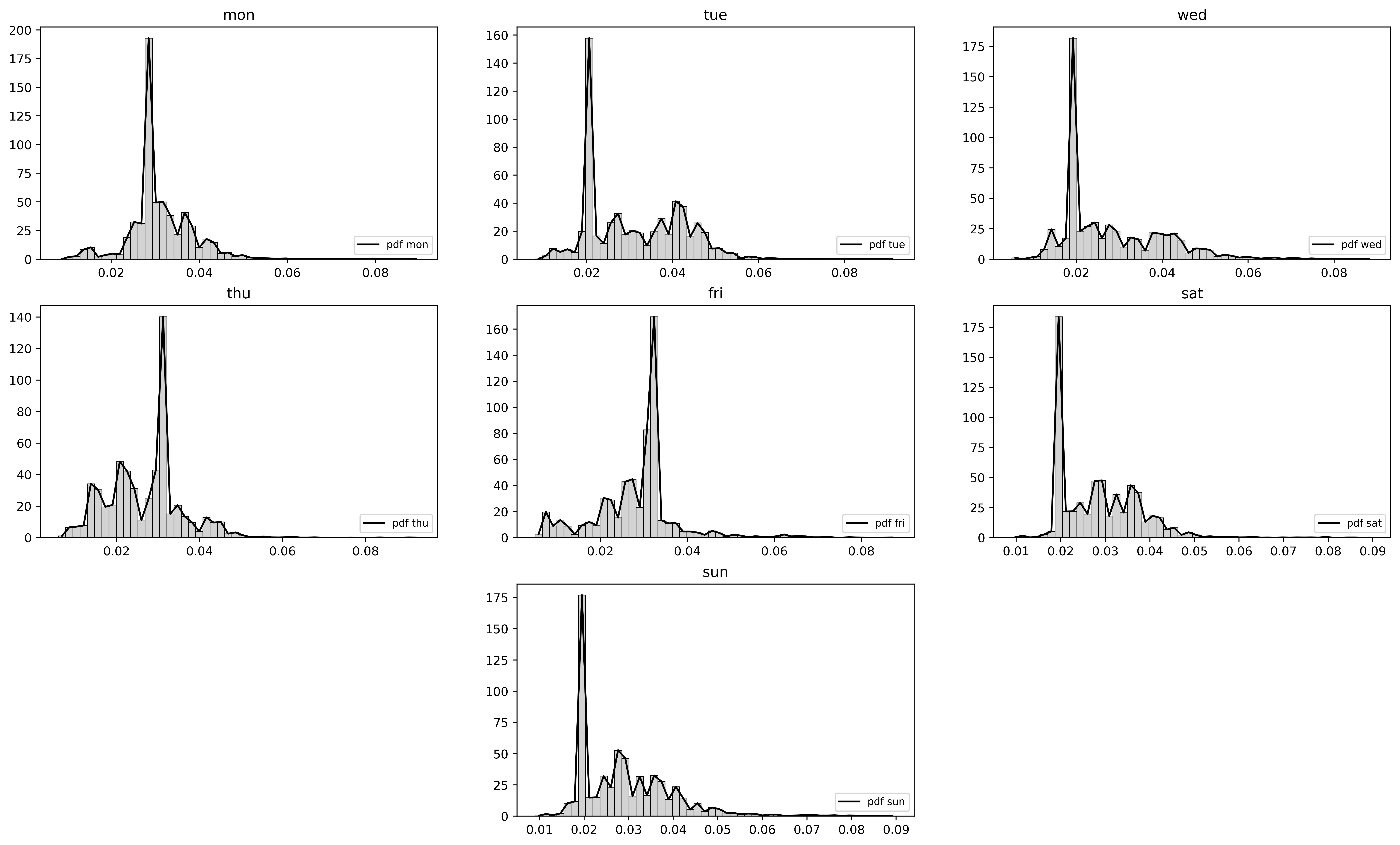

Supplement: Supplementary_material_for_Radiation_Protection_Dosimetry_Manuscript_2019_ncz154 [file supplementary_material_for_radiation_protection_dosimetry_manuscript_2019_ncz154.zip › Supplementary material for Radiation Protection Dosimetry Manuscript 2019/Location1_Figures_1stWeek/Figure2_DCS_1stWeek.jpg]

mon

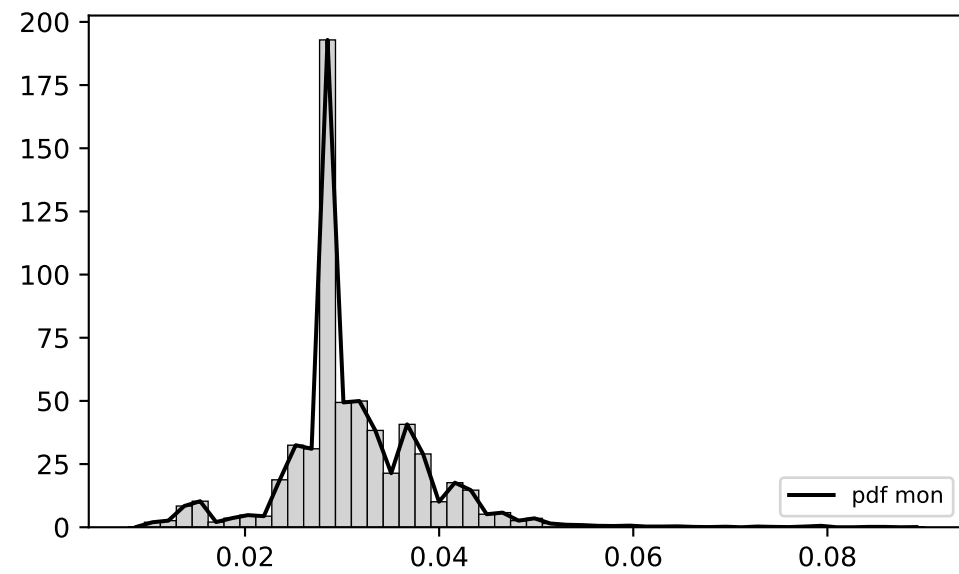

tue

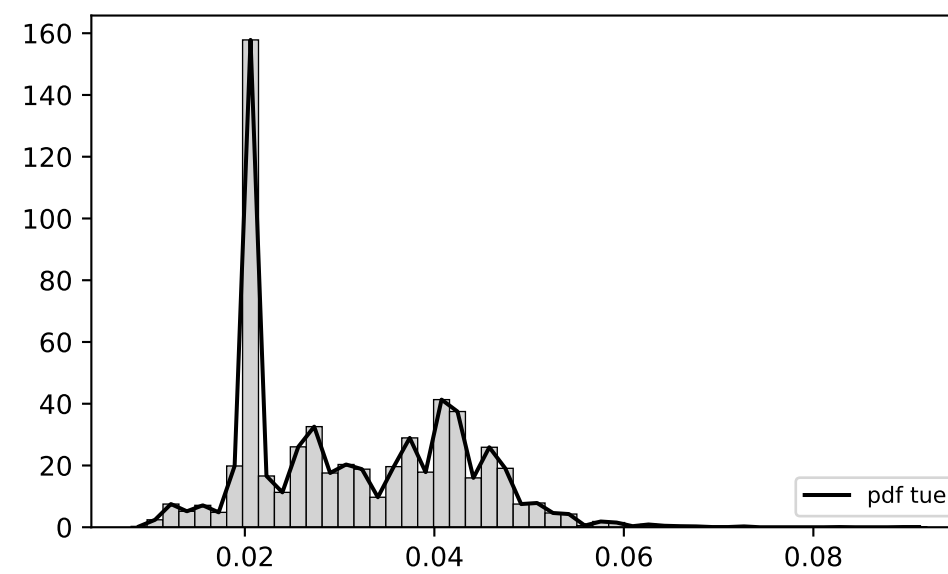

wed

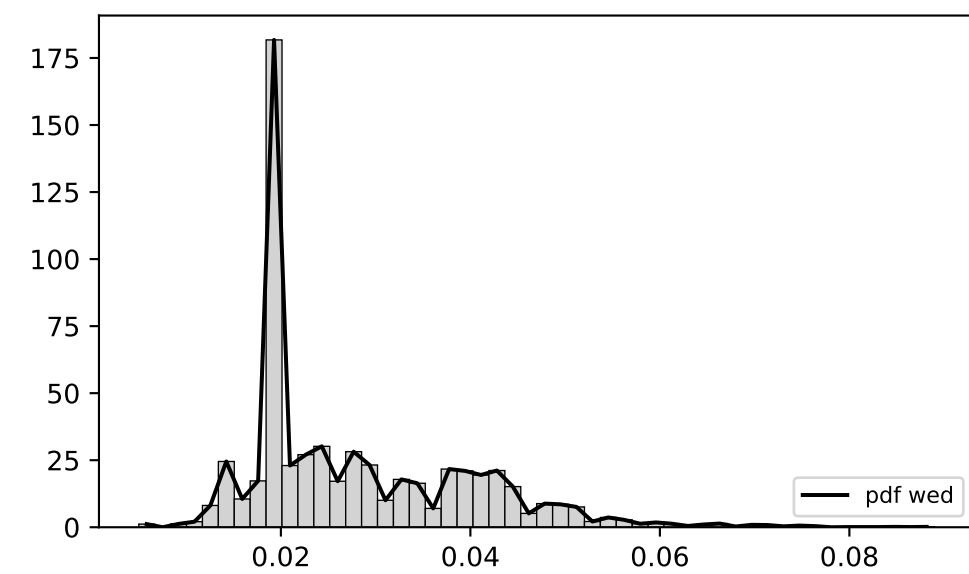

thu

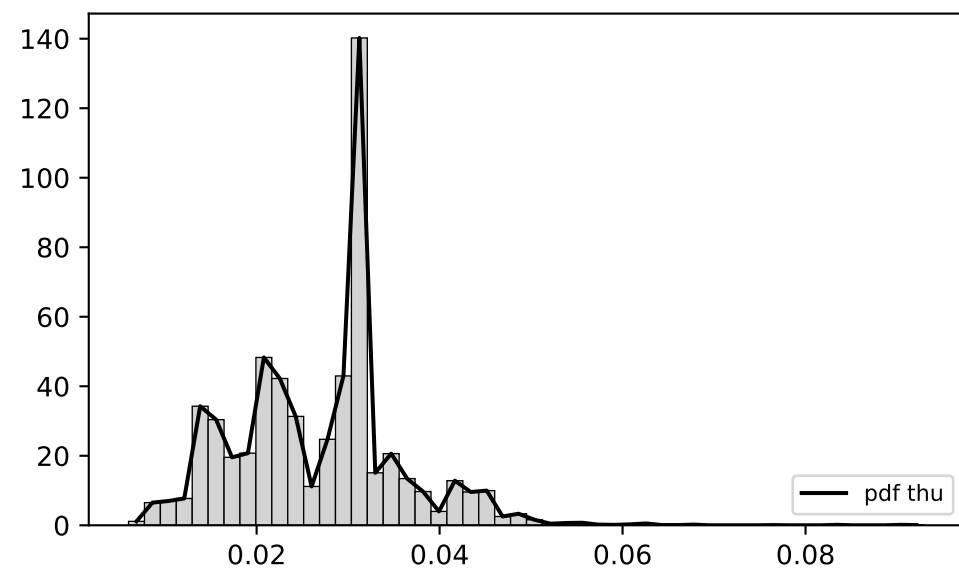

fri

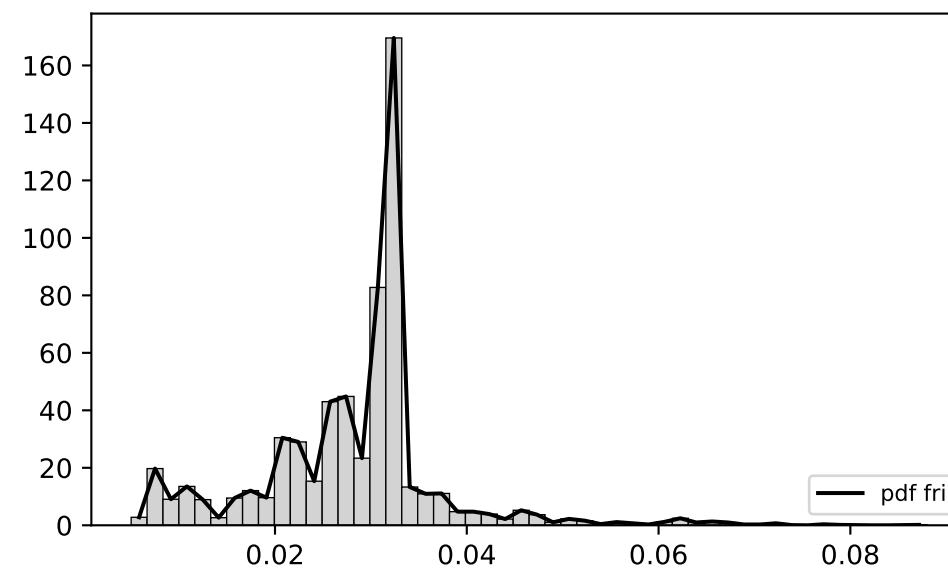

sat

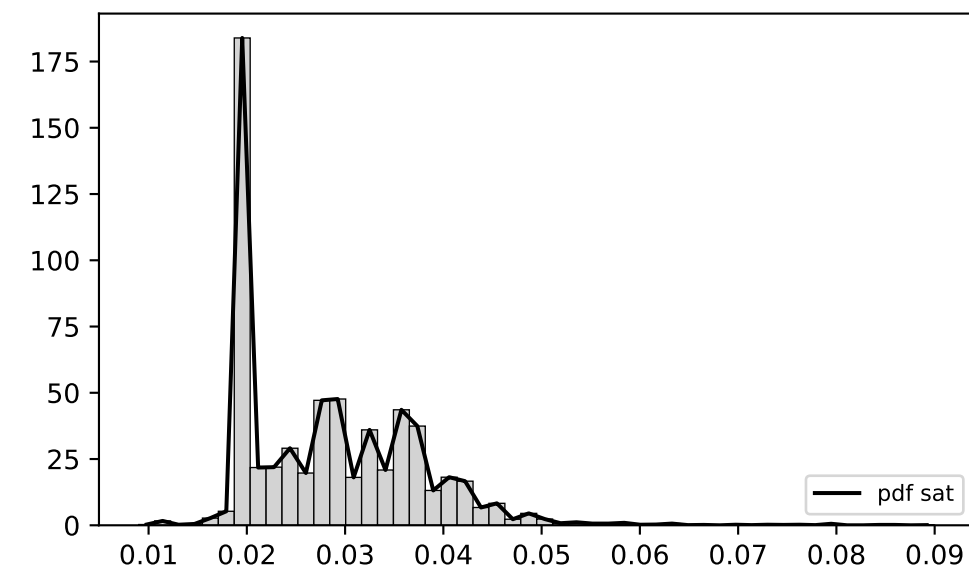

sun

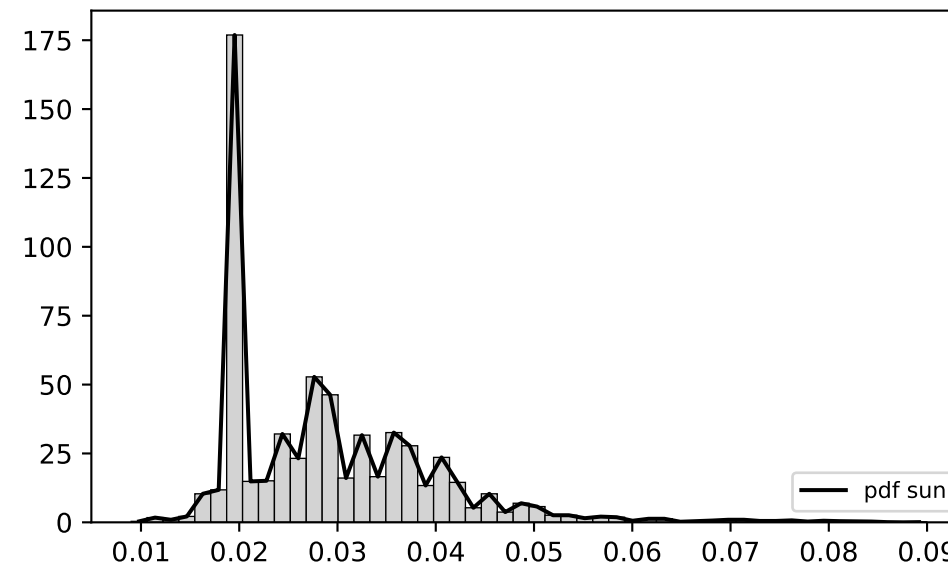

Supplement: Supplementary_material_for_Radiation_Protection_Dosimetry_Manuscript_2019_ncz154 [file supplementary_material_for_radiation_protection_dosimetry_manuscript_2019_ncz154.zip › Supplementary material for Radiation Protection Dosimetry Manuscript 2019/Location1_Figures_1stWeek/Figure2_DCS_1stWeek.pdf]

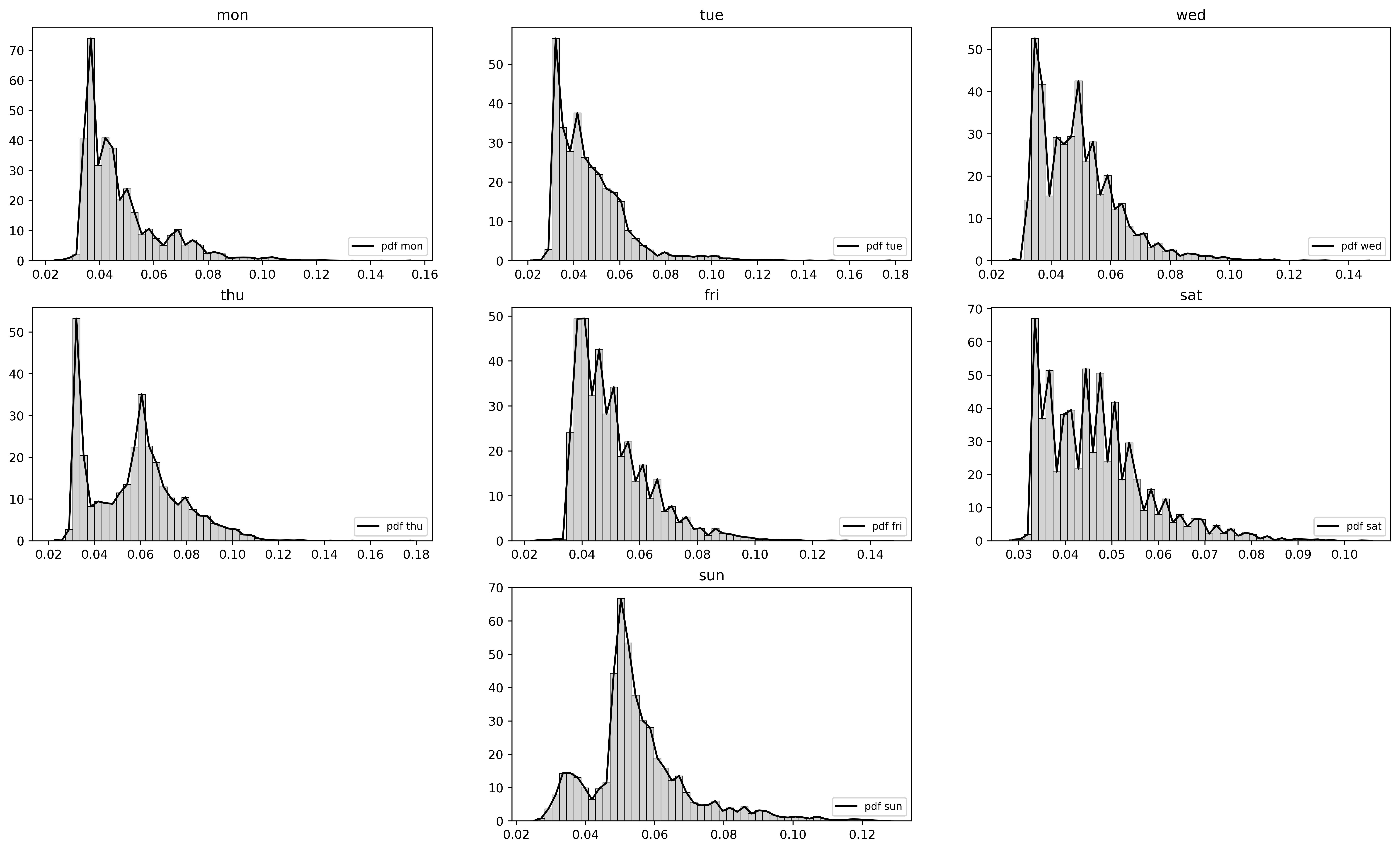

Supplement: Supplementary_material_for_Radiation_Protection_Dosimetry_Manuscript_2019_ncz154 [file supplementary_material_for_radiation_protection_dosimetry_manuscript_2019_ncz154.zip › Supplementary material for Radiation Protection Dosimetry Manuscript 2019/Location1_Figures_1stWeek/Figure2_UMTS_1stWeek.jpg]

mon

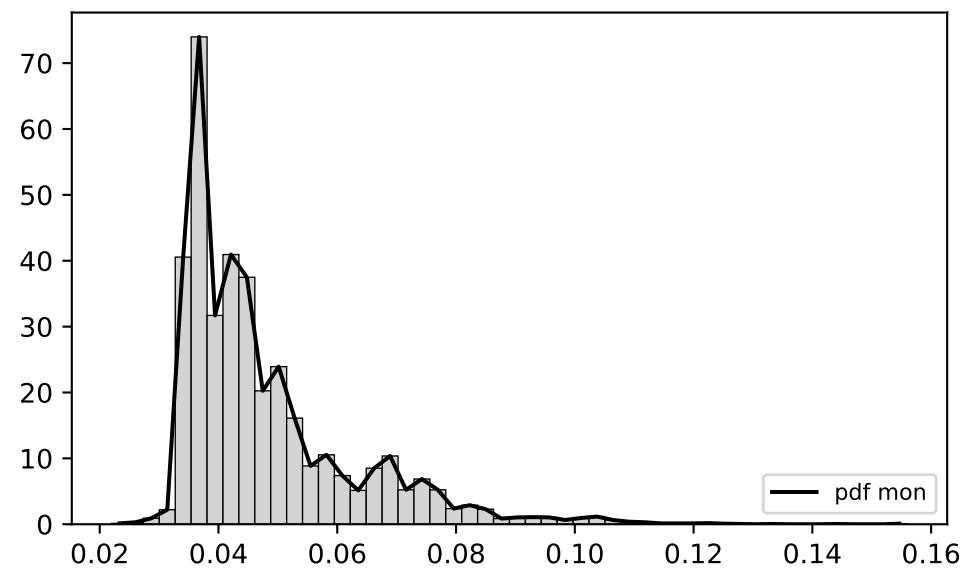

tue

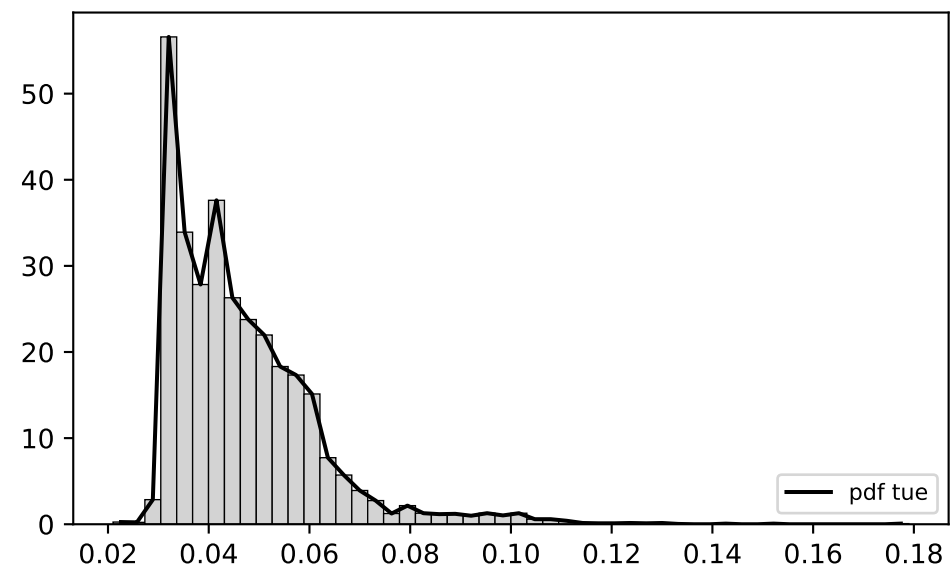

wed

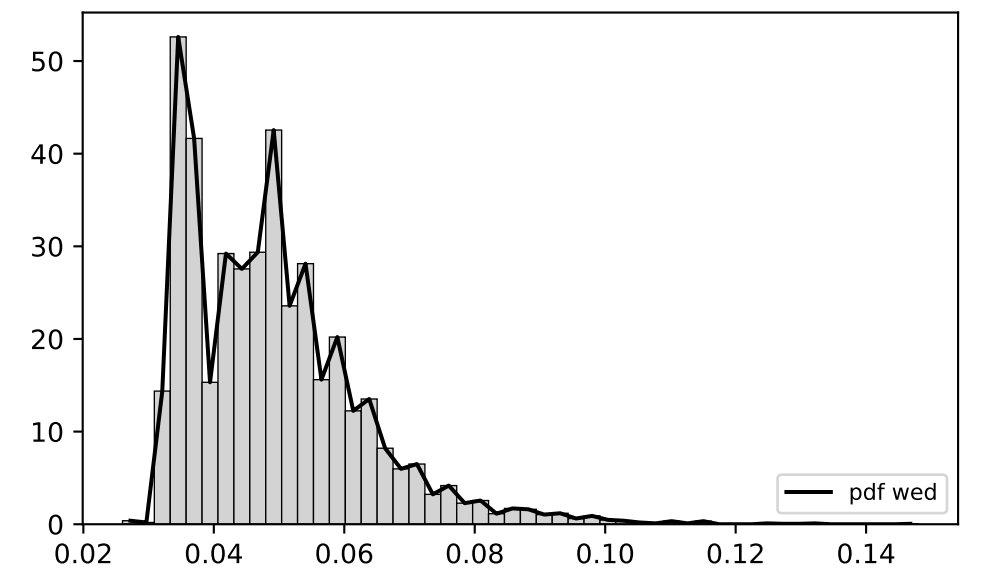

thu

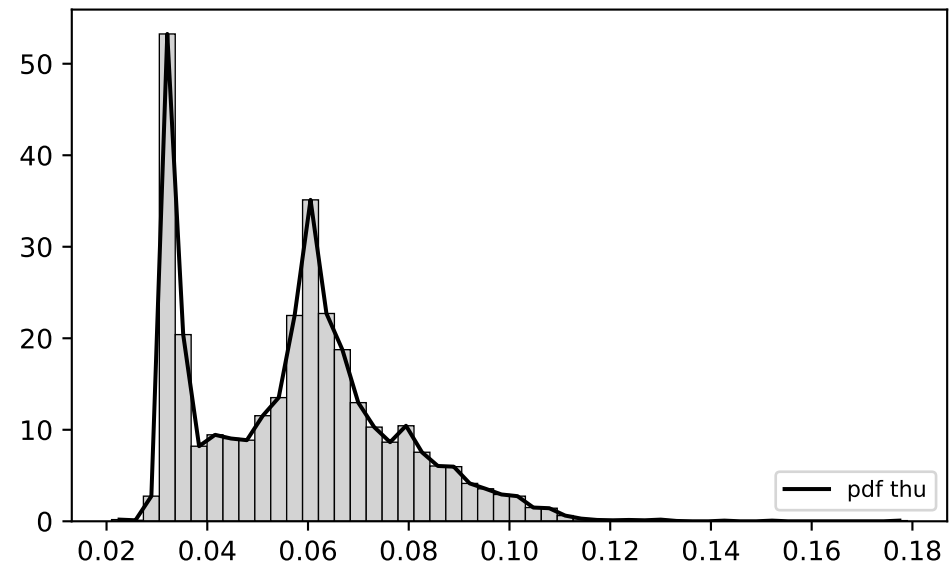

fri

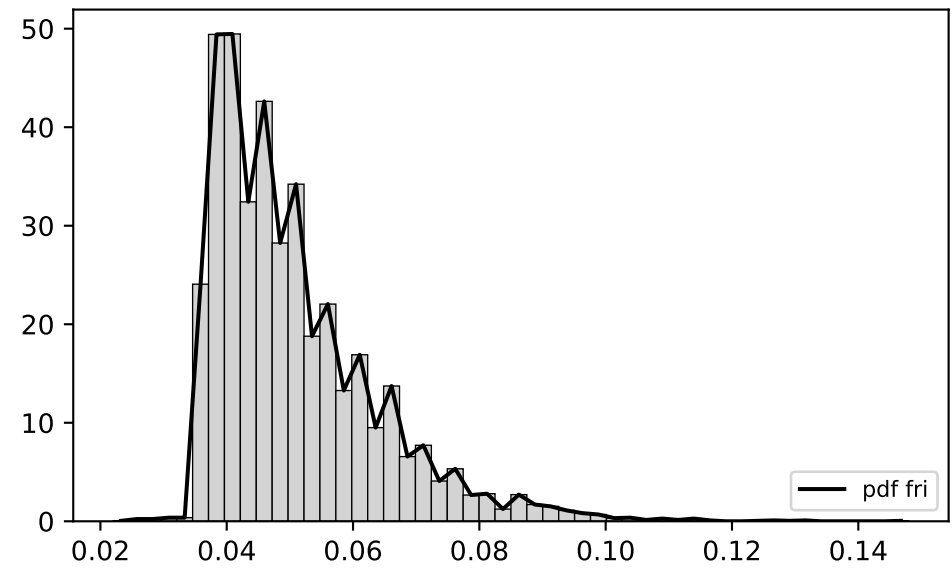

sat

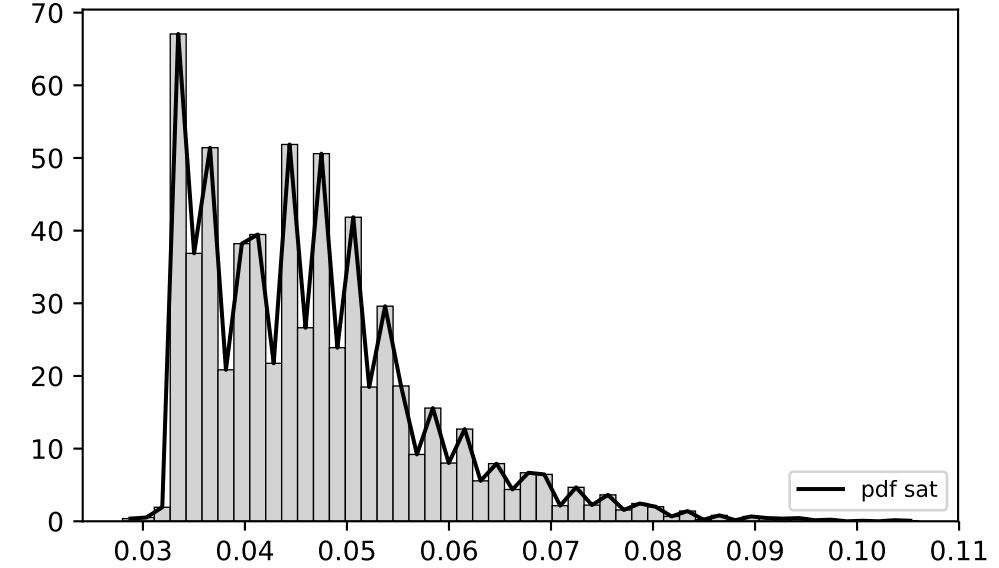

sun

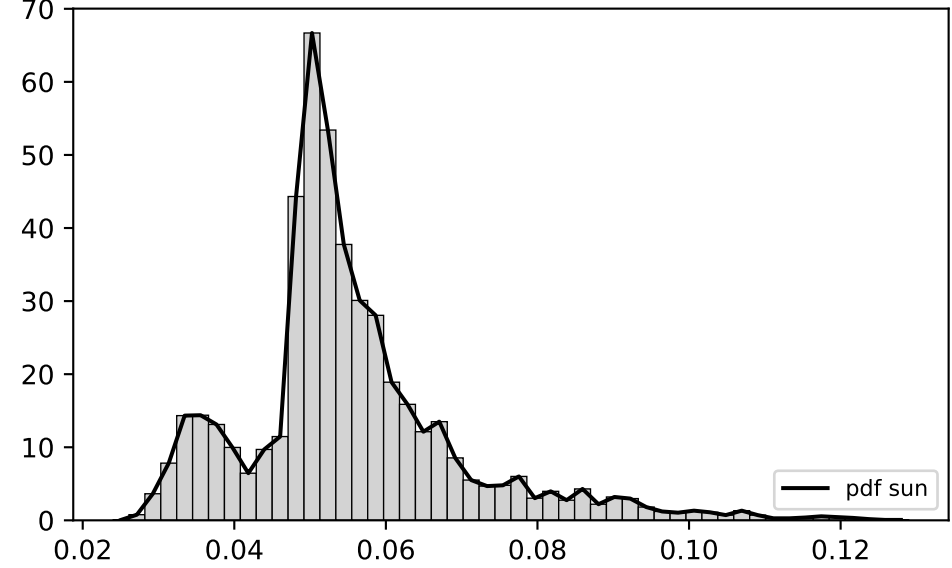

Supplement: Supplementary_material_for_Radiation_Protection_Dosimetry_Manuscript_2019_ncz154 [file supplementary_material_for_radiation_protection_dosimetry_manuscript_2019_ncz154.zip › Supplementary material for Radiation Protection Dosimetry Manuscript 2019/Location1_Figures_1stWeek/Figure2_UMTS_1stWeek.pdf]

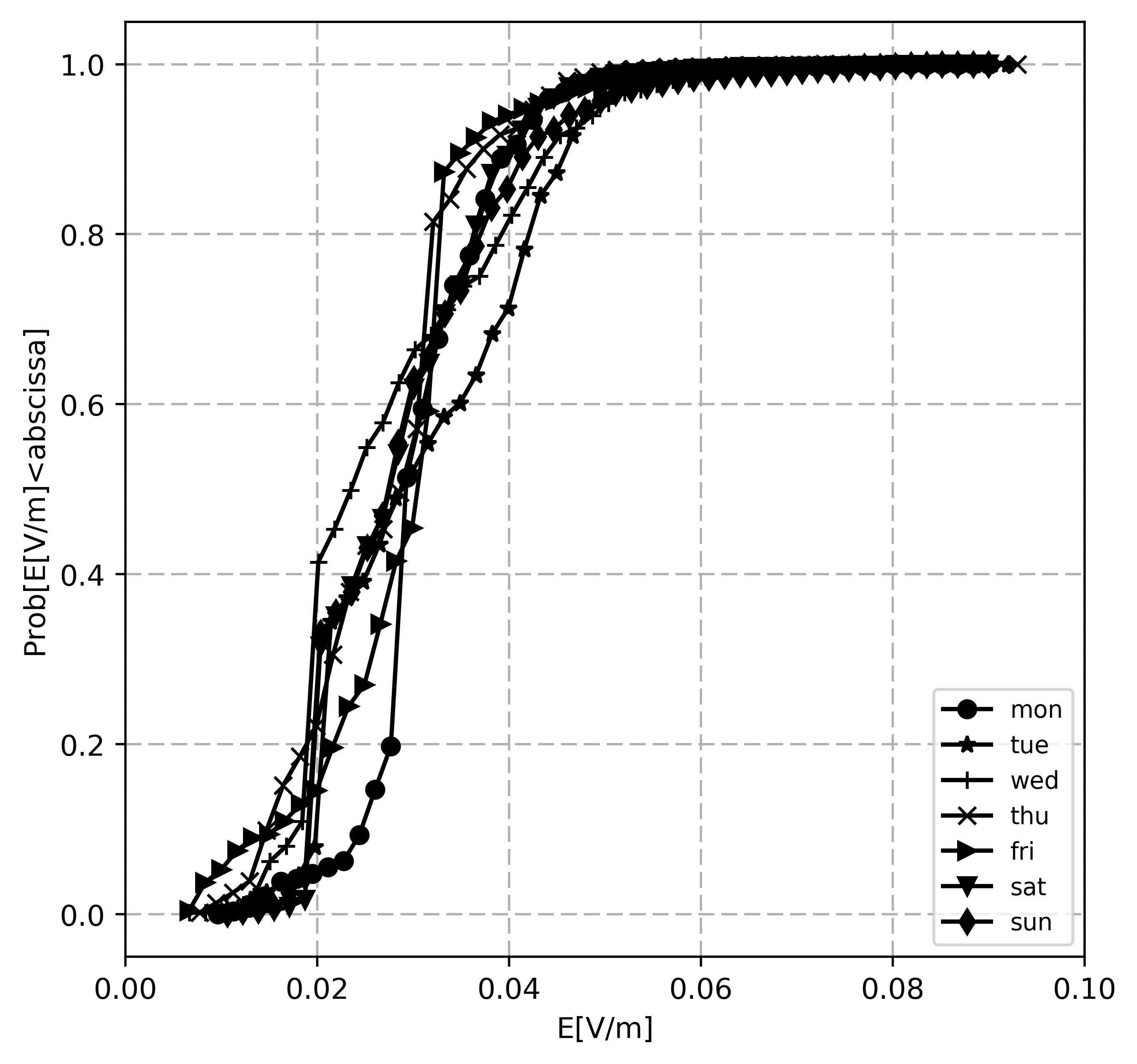

Supplement: Supplementary_material_for_Radiation_Protection_Dosimetry_Manuscript_2019_ncz154 [file supplementary_material_for_radiation_protection_dosimetry_manuscript_2019_ncz154.zip › Supplementary material for Radiation Protection Dosimetry Manuscript 2019/Location1_Figures_1stWeek/Figure3_DCS_1stWeek.jpg]

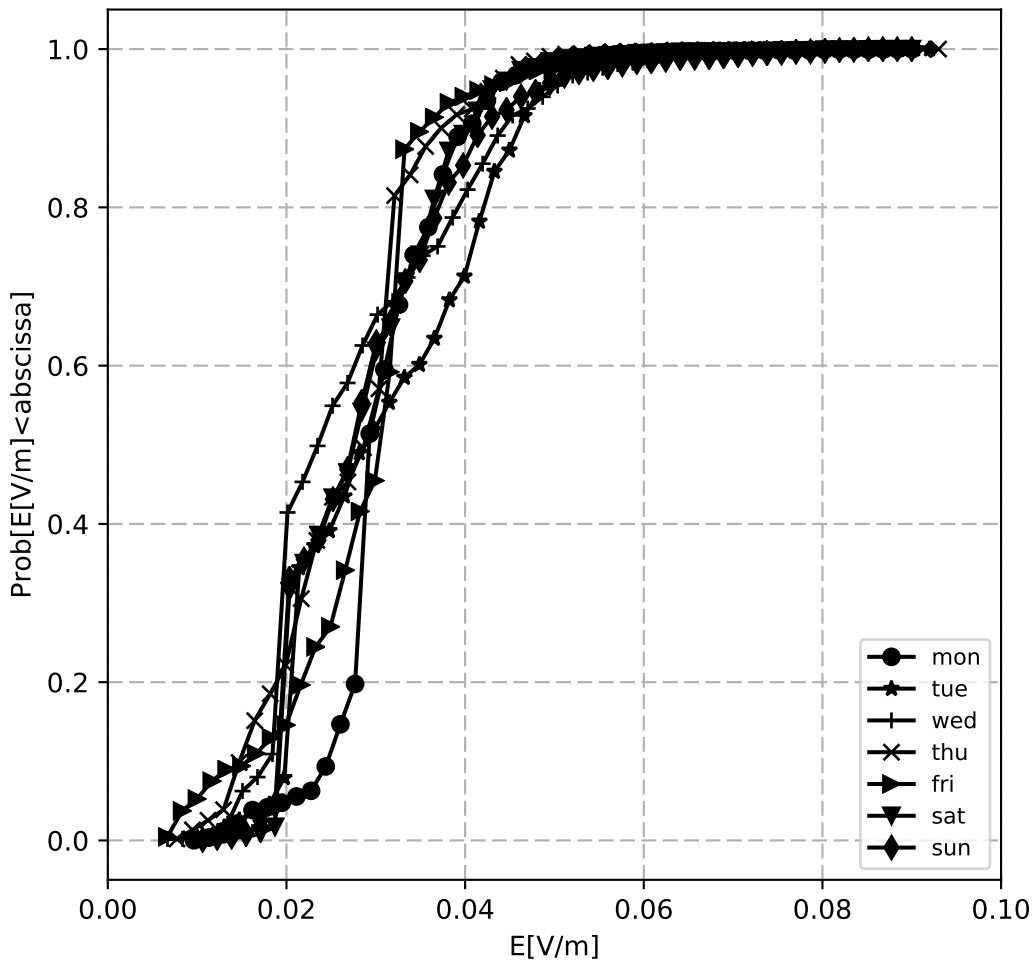

Supplement: Supplementary_material_for_Radiation_Protection_Dosimetry_Manuscript_2019_ncz154 [file supplementary_material_for_radiation_protection_dosimetry_manuscript_2019_ncz154.zip › Supplementary material for Radiation Protection Dosimetry Manuscript 2019/Location1_Figures_1stWeek/Figure3_DCS_1stWeek.pdf]

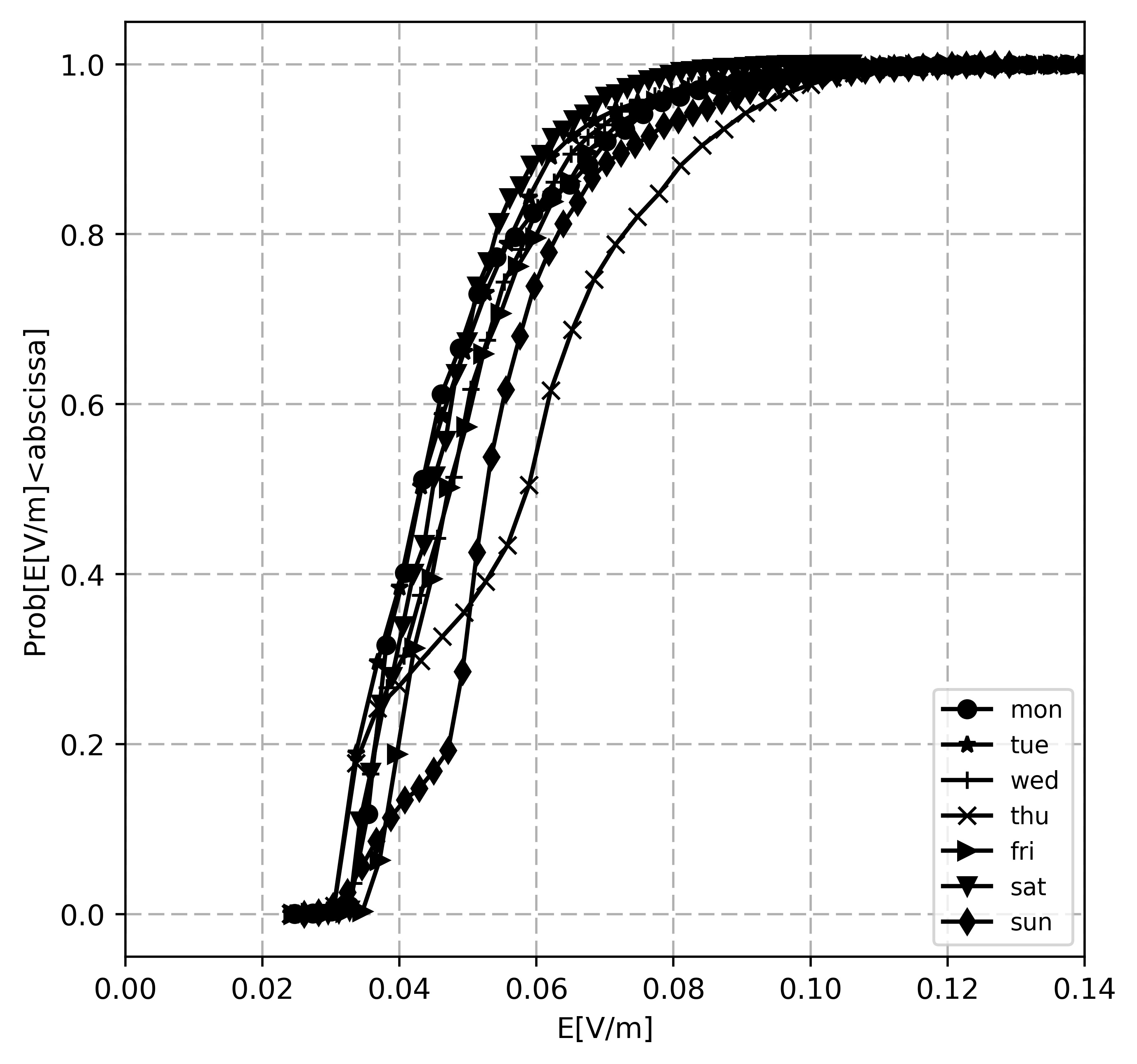

Supplement: Supplementary_material_for_Radiation_Protection_Dosimetry_Manuscript_2019_ncz154 [file supplementary_material_for_radiation_protection_dosimetry_manuscript_2019_ncz154.zip › Supplementary material for Radiation Protection Dosimetry Manuscript 2019/Location1_Figures_1stWeek/Figure3_UMTS_1stWeek.jpg]

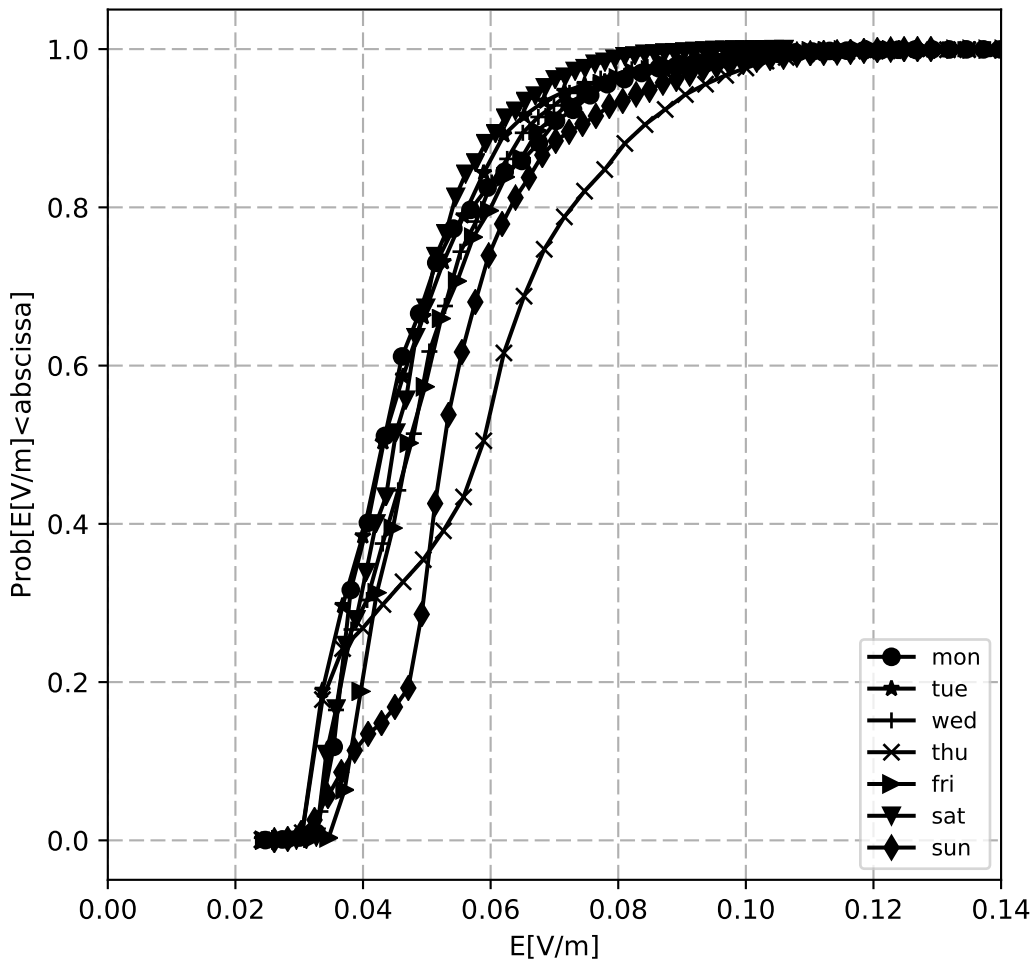

Supplement: Supplementary_material_for_Radiation_Protection_Dosimetry_Manuscript_2019_ncz154 [file supplementary_material_for_radiation_protection_dosimetry_manuscript_2019_ncz154.zip › Supplementary material for Radiation Protection Dosimetry Manuscript 2019/Location1_Figures_1stWeek/Figure3_UMTS_1stWeek.pdf]

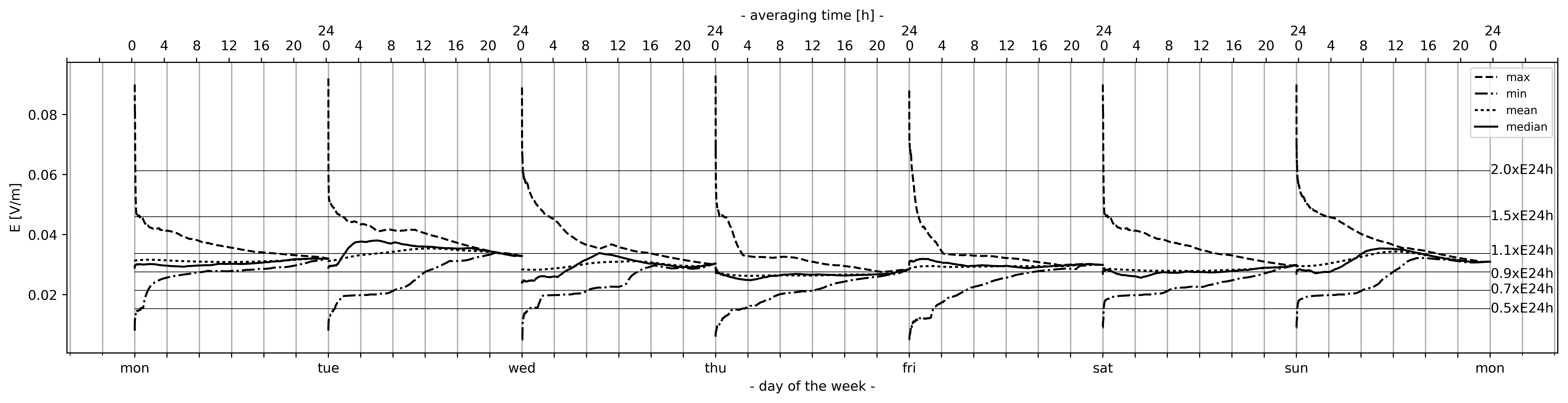

Supplement: Supplementary_material_for_Radiation_Protection_Dosimetry_Manuscript_2019_ncz154 [file supplementary_material_for_radiation_protection_dosimetry_manuscript_2019_ncz154.zip › Supplementary material for Radiation Protection Dosimetry Manuscript 2019/Location1_Figures_1stWeek/Figure4_DCS_1stWeek.jpg]

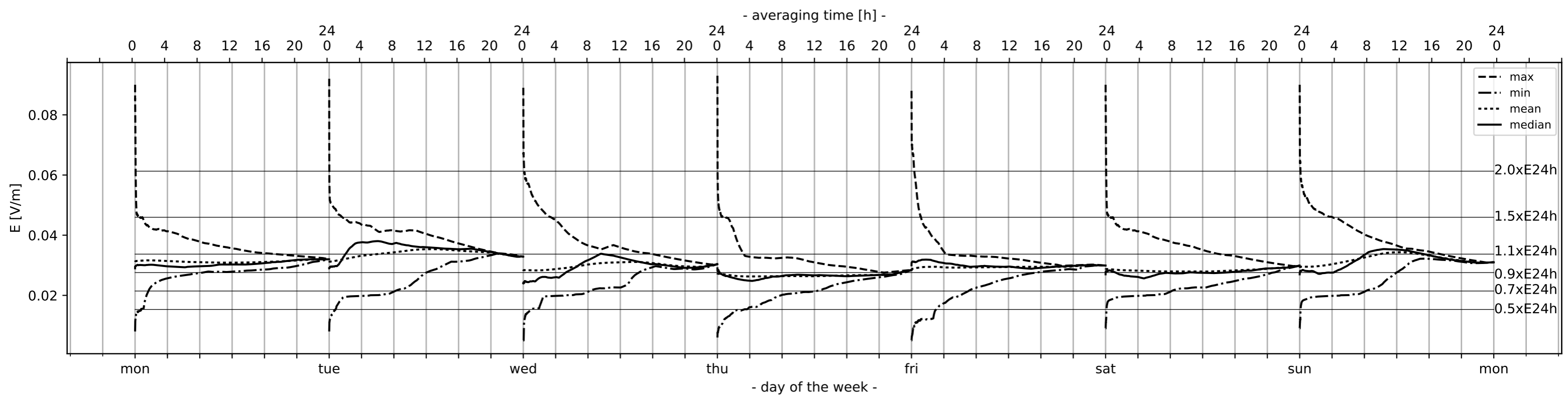

Supplement: Supplementary_material_for_Radiation_Protection_Dosimetry_Manuscript_2019_ncz154 [file supplementary_material_for_radiation_protection_dosimetry_manuscript_2019_ncz154.zip › Supplementary material for Radiation Protection Dosimetry Manuscript 2019/Location1_Figures_1stWeek/Figure4_DCS_1stWeek.pdf]

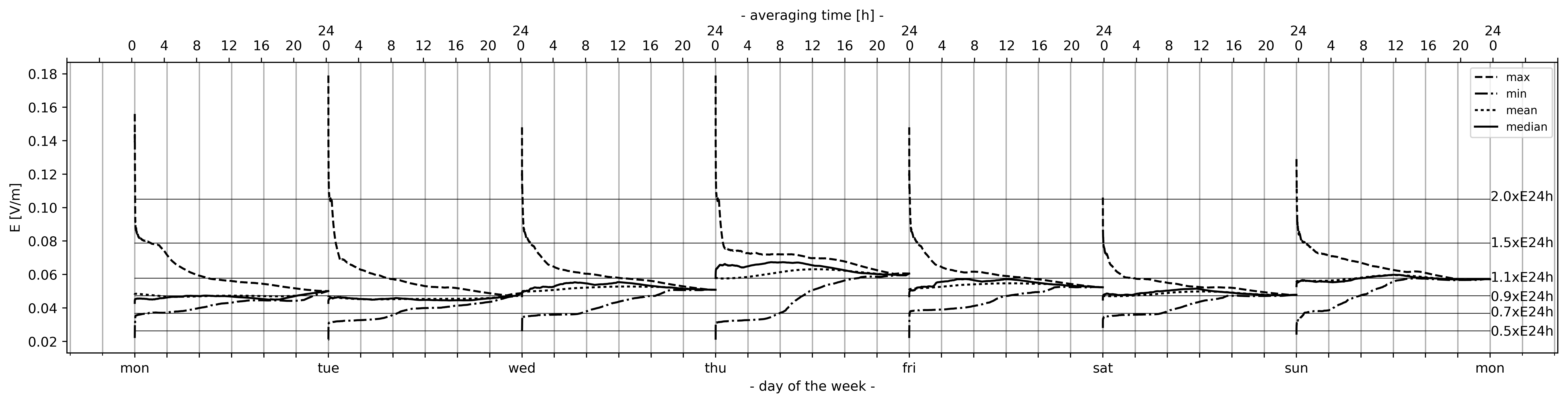

Supplement: Supplementary_material_for_Radiation_Protection_Dosimetry_Manuscript_2019_ncz154 [file supplementary_material_for_radiation_protection_dosimetry_manuscript_2019_ncz154.zip › Supplementary material for Radiation Protection Dosimetry Manuscript 2019/Location1_Figures_1stWeek/Figure4_UMTS_1stWeek.jpg]

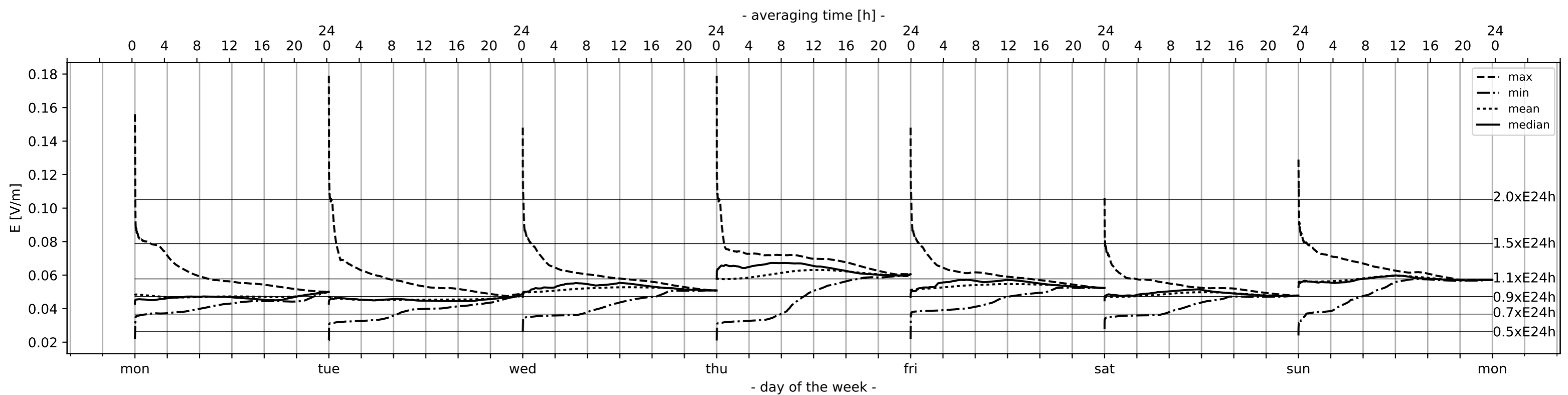

Supplement: Supplementary_material_for_Radiation_Protection_Dosimetry_Manuscript_2019_ncz154 [file supplementary_material_for_radiation_protection_dosimetry_manuscript_2019_ncz154.zip › Supplementary material for Radiation Protection Dosimetry Manuscript 2019/Location1_Figures_1stWeek/Figure4_UMTS_1stWeek.pdf]

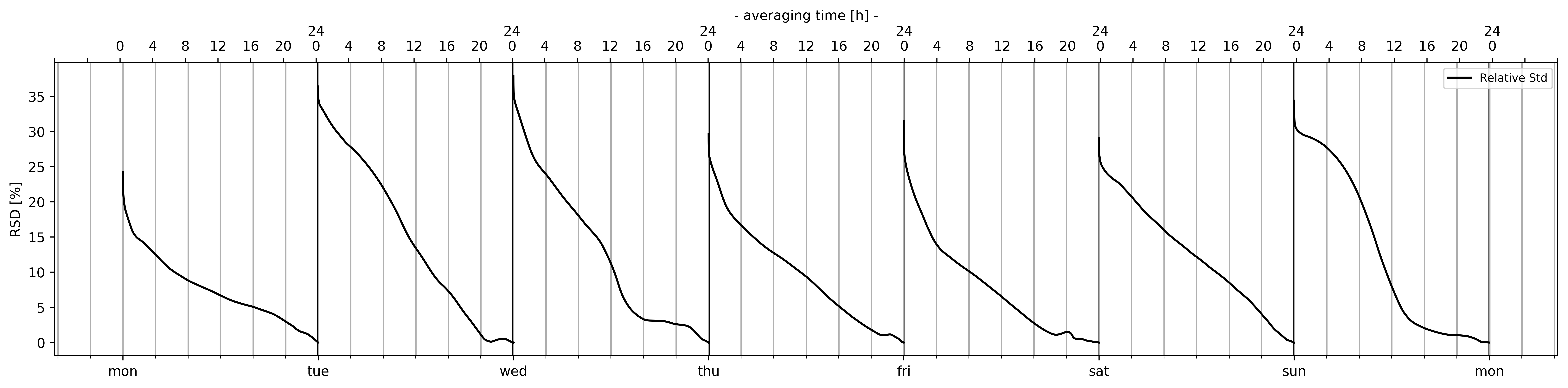

Supplement: Supplementary_material_for_Radiation_Protection_Dosimetry_Manuscript_2019_ncz154 [file supplementary_material_for_radiation_protection_dosimetry_manuscript_2019_ncz154.zip › Supplementary material for Radiation Protection Dosimetry Manuscript 2019/Location1_Figures_1stWeek/Figure5_DCS_1stWeek.jpg]

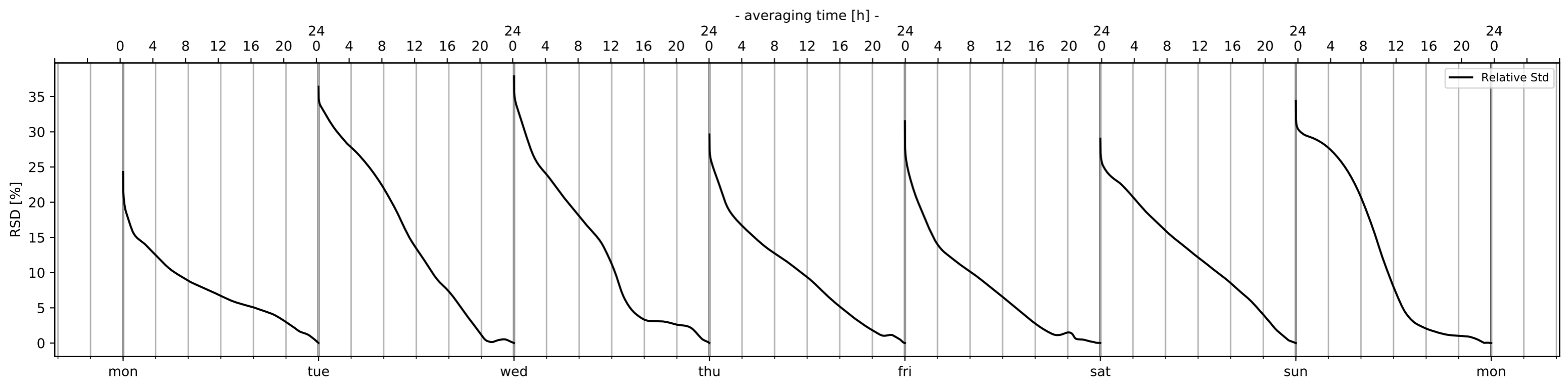

Supplement: Supplementary_material_for_Radiation_Protection_Dosimetry_Manuscript_2019_ncz154 [file supplementary_material_for_radiation_protection_dosimetry_manuscript_2019_ncz154.zip › Supplementary material for Radiation Protection Dosimetry Manuscript 2019/Location1_Figures_1stWeek/Figure5_DCS_1stWeek.pdf]

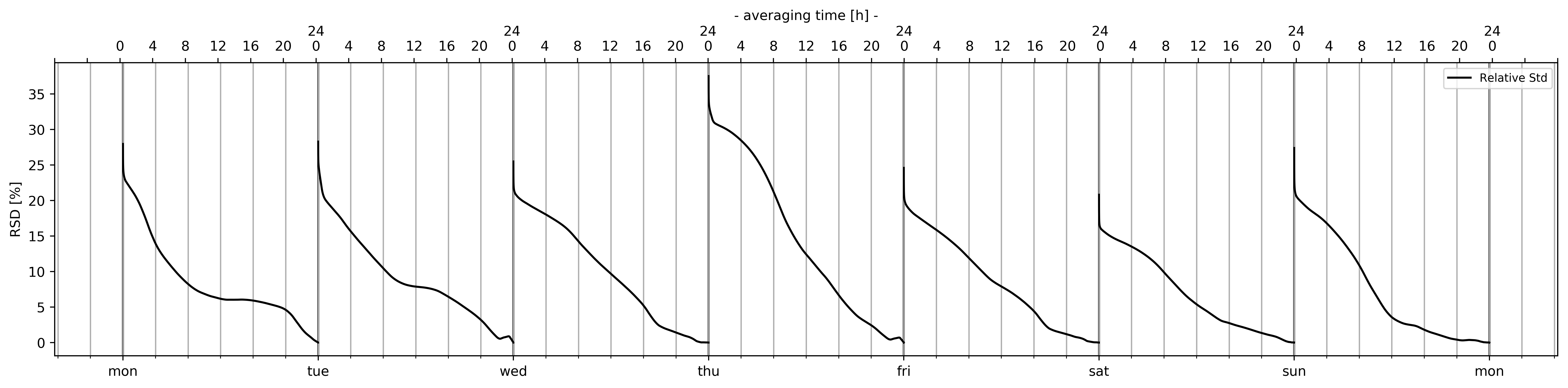

Supplement: Supplementary_material_for_Radiation_Protection_Dosimetry_Manuscript_2019_ncz154 [file supplementary_material_for_radiation_protection_dosimetry_manuscript_2019_ncz154.zip › Supplementary material for Radiation Protection Dosimetry Manuscript 2019/Location1_Figures_1stWeek/Figure5_UMTS_1stWeek.jpg]

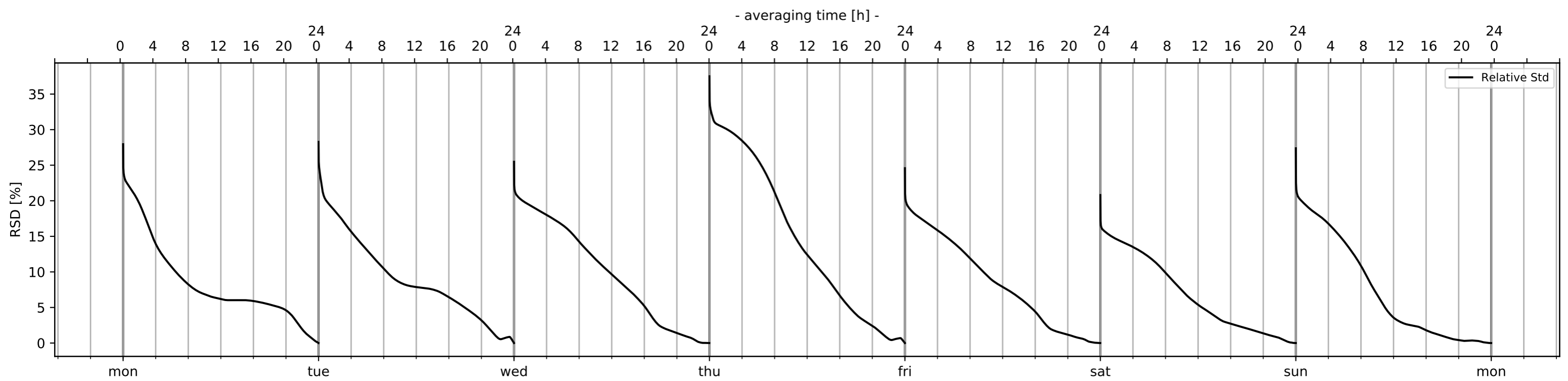

Supplement: Supplementary_material_for_Radiation_Protection_Dosimetry_Manuscript_2019_ncz154 [file supplementary_material_for_radiation_protection_dosimetry_manuscript_2019_ncz154.zip › Supplementary material for Radiation Protection Dosimetry Manuscript 2019/Location1_Figures_1stWeek/Figure5_UMTS_1stWeek.pdf]

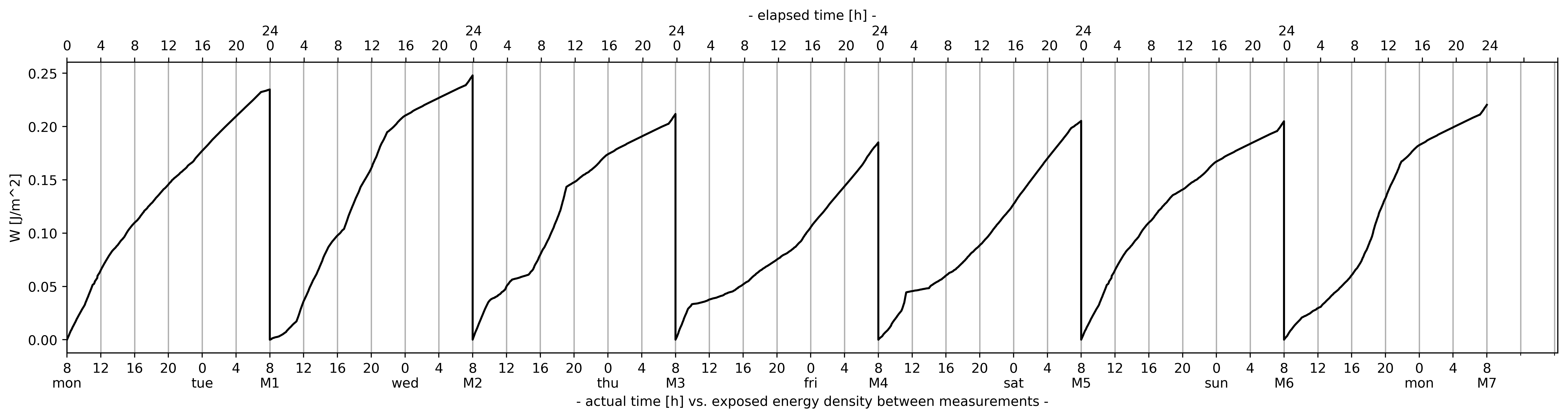

Supplement: Supplementary_material_for_Radiation_Protection_Dosimetry_Manuscript_2019_ncz154 [file supplementary_material_for_radiation_protection_dosimetry_manuscript_2019_ncz154.zip › Supplementary material for Radiation Protection Dosimetry Manuscript 2019/Location1_Figures_1stWeek/Figure6_DCS_1stWeek.jpg]

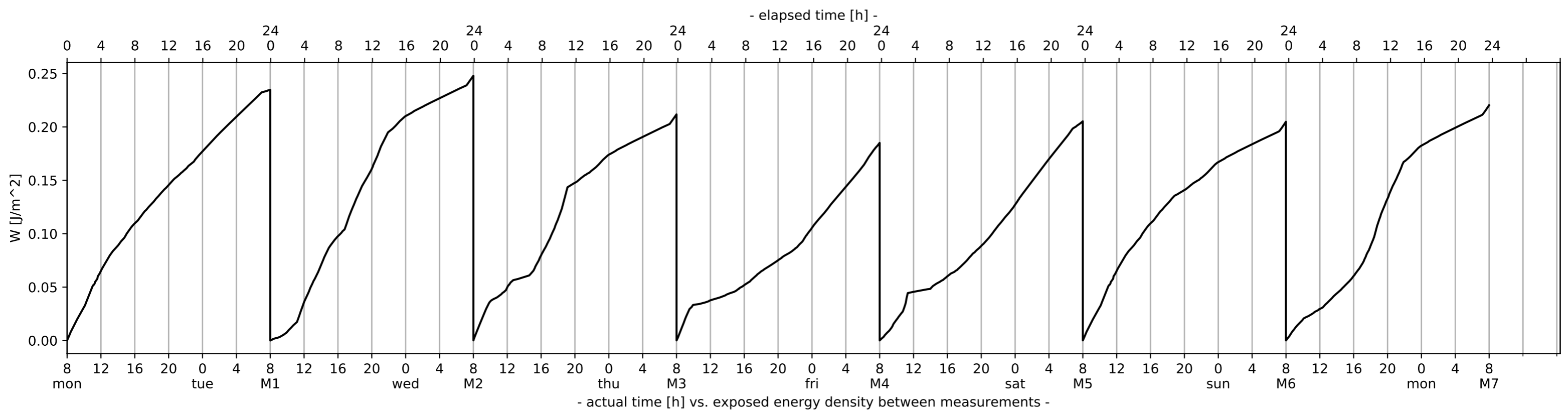

Supplement: Supplementary_material_for_Radiation_Protection_Dosimetry_Manuscript_2019_ncz154 [file supplementary_material_for_radiation_protection_dosimetry_manuscript_2019_ncz154.zip › Supplementary material for Radiation Protection Dosimetry Manuscript 2019/Location1_Figures_1stWeek/Figure6_DCS_1stWeek.pdf]

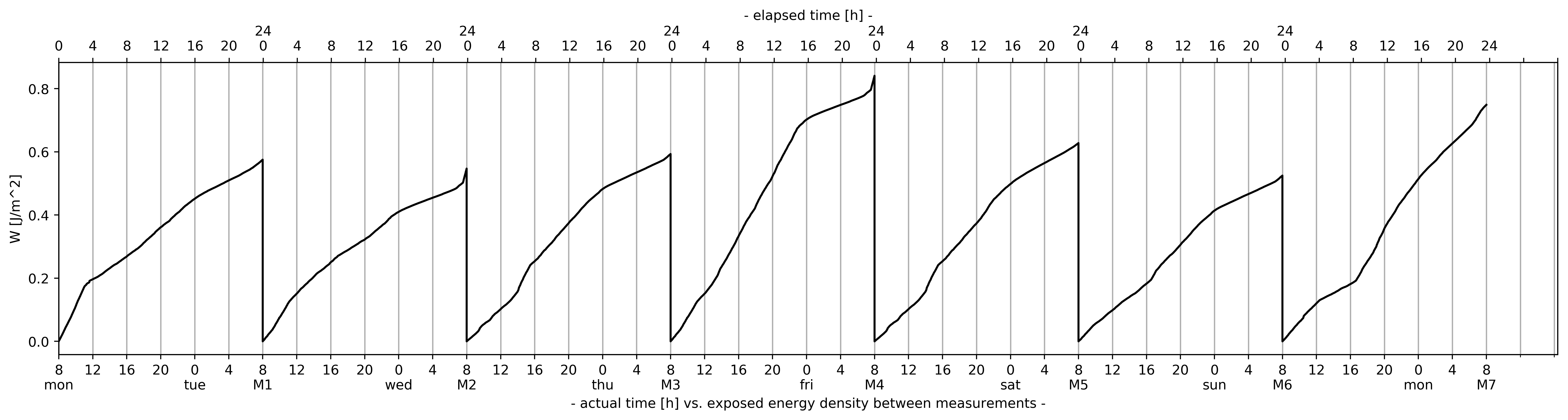

Supplement: Supplementary_material_for_Radiation_Protection_Dosimetry_Manuscript_2019_ncz154 [file supplementary_material_for_radiation_protection_dosimetry_manuscript_2019_ncz154.zip › Supplementary material for Radiation Protection Dosimetry Manuscript 2019/Location1_Figures_1stWeek/Figure6_UMTS_1stWeek.jpg]

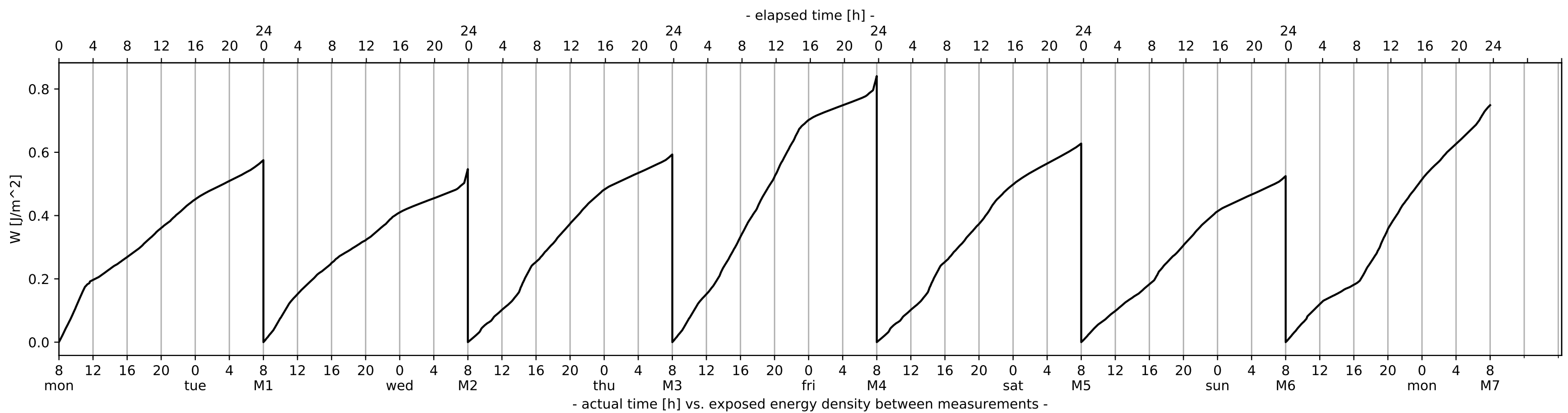

Supplement: Supplementary_material_for_Radiation_Protection_Dosimetry_Manuscript_2019_ncz154 [file supplementary_material_for_radiation_protection_dosimetry_manuscript_2019_ncz154.zip › Supplementary material for Radiation Protection Dosimetry Manuscript 2019/Location1_Figures_1stWeek/Figure6_UMTS_1stWeek.pdf]

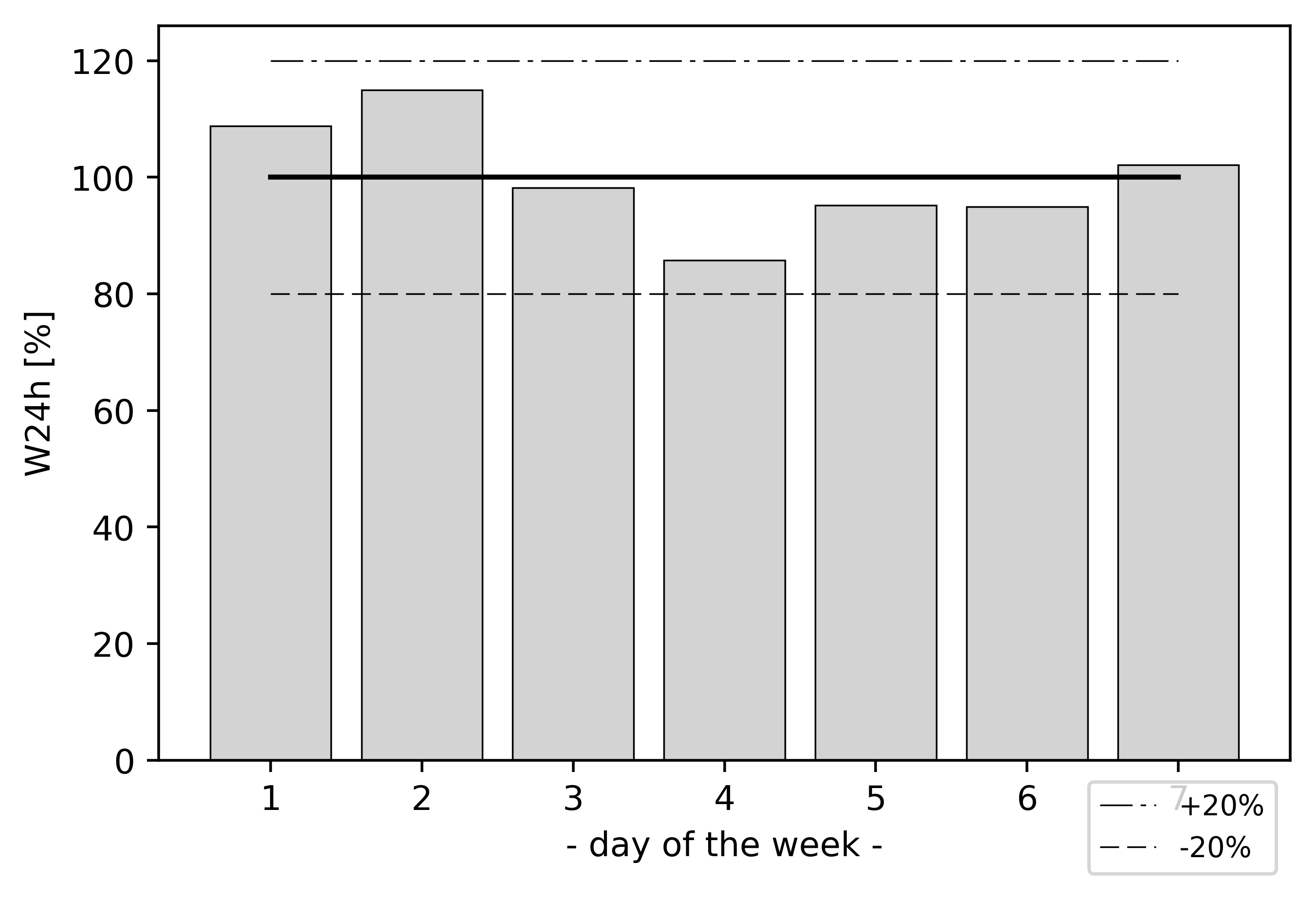

Supplement: Supplementary_material_for_Radiation_Protection_Dosimetry_Manuscript_2019_ncz154 [file supplementary_material_for_radiation_protection_dosimetry_manuscript_2019_ncz154.zip › Supplementary material for Radiation Protection Dosimetry Manuscript 2019/Location1_Figures_1stWeek/Figure7_DCS_1stWeek.jpg]

W24h [%]

120  
100  
80  
60  
40  
20  
0

1

2

3

4

5

6

- day of the week -

— ··· +20%  
— ··· -20%

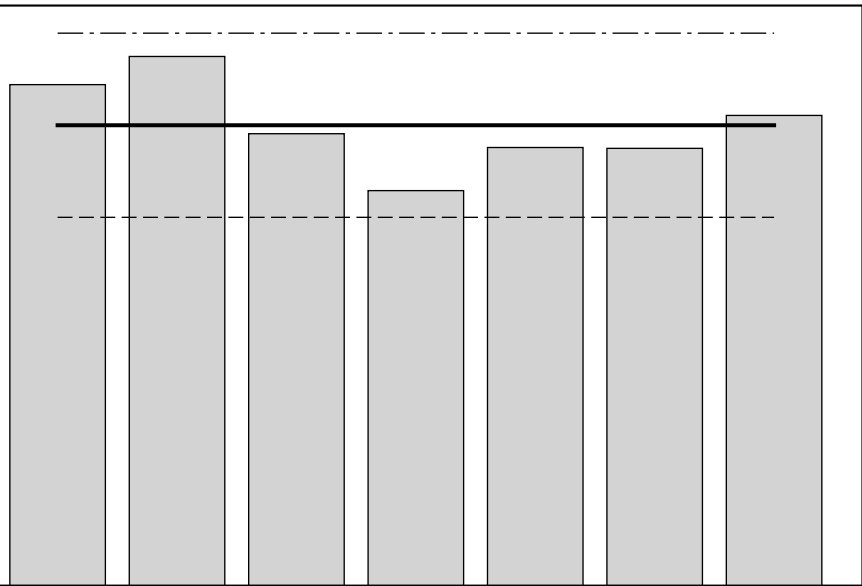

Supplement: Supplementary_material_for_Radiation_Protection_Dosimetry_Manuscript_2019_ncz154 [file supplementary_material_for_radiation_protection_dosimetry_manuscript_2019_ncz154.zip › Supplementary material for Radiation Protection Dosimetry Manuscript 2019/Location1_Figures_1stWeek/Figure7_DCS_1stWeek.pdf]

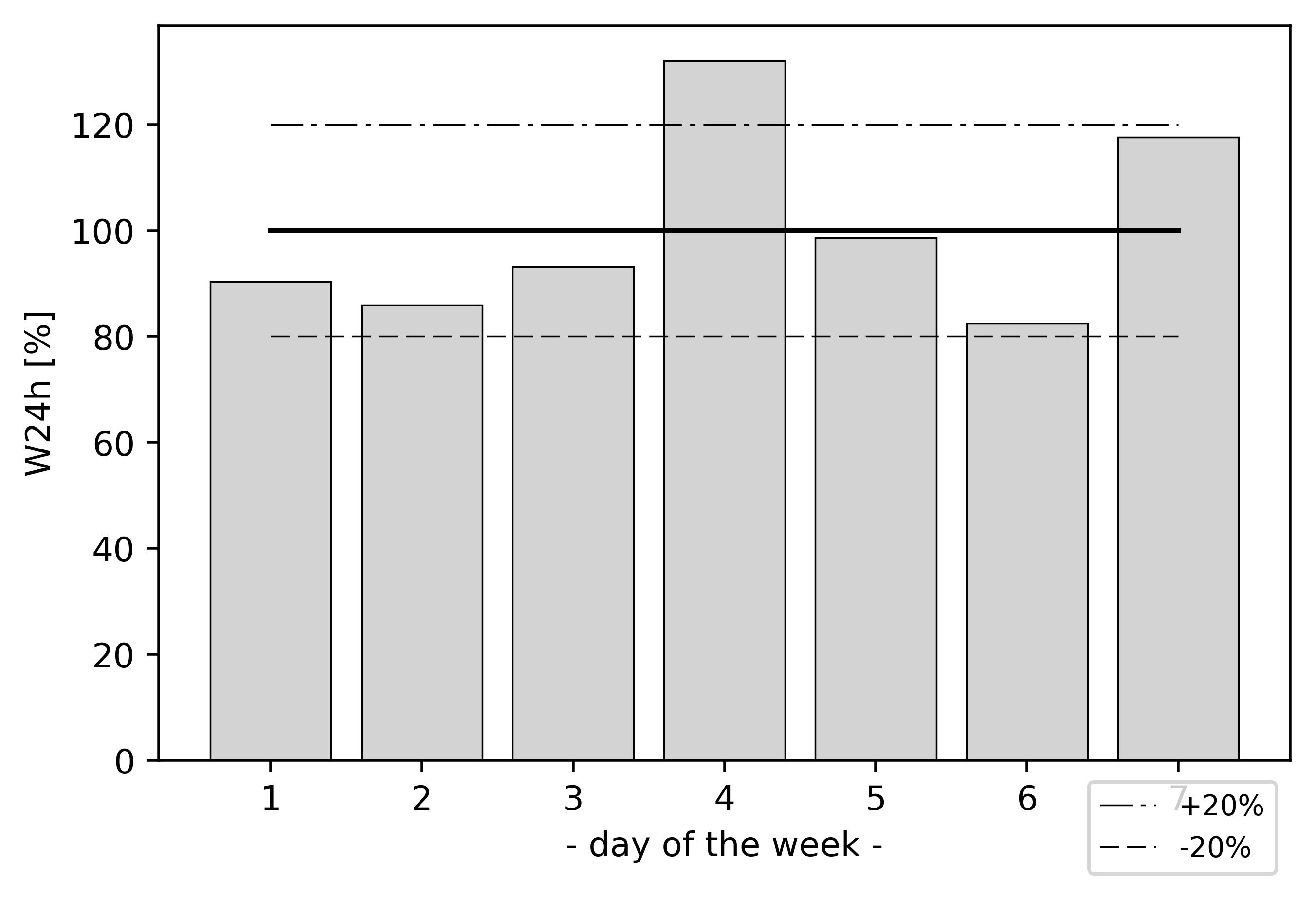

Supplement: Supplementary_material_for_Radiation_Protection_Dosimetry_Manuscript_2019_ncz154 [file supplementary_material_for_radiation_protection_dosimetry_manuscript_2019_ncz154.zip › Supplementary material for Radiation Protection Dosimetry Manuscript 2019/Location1_Figures_1stWeek/Figure7_UMTS_1stWeek.jpg]

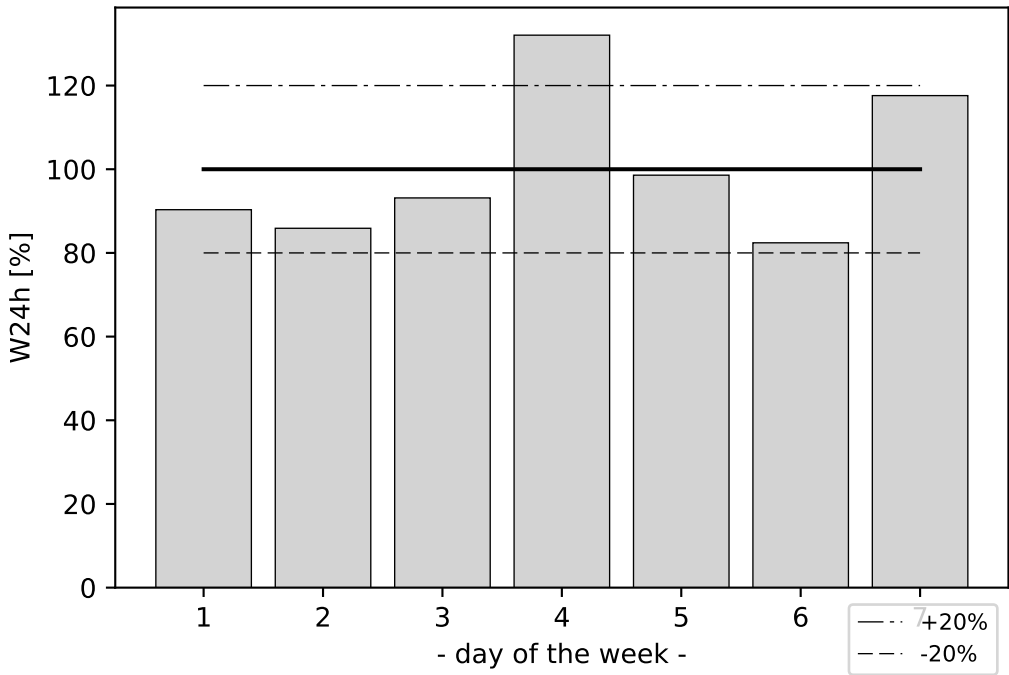

Supplement: Supplementary_material_for_Radiation_Protection_Dosimetry_Manuscript_2019_ncz154 [file supplementary_material_for_radiation_protection_dosimetry_manuscript_2019_ncz154.zip › Supplementary material for Radiation Protection Dosimetry Manuscript 2019/Location1_Figures_1stWeek/Figure7_UMTS_1stWeek.pdf]

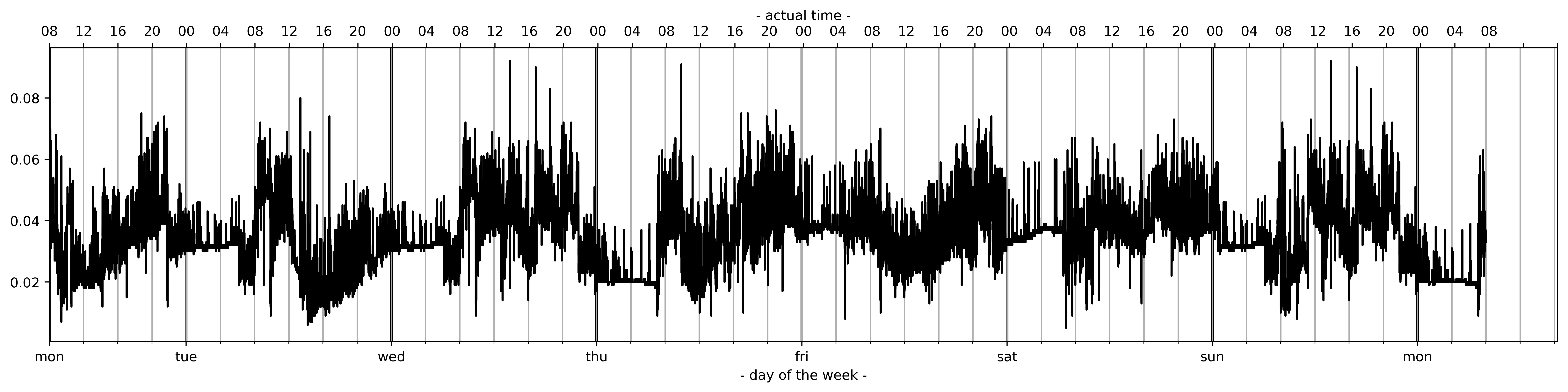

Supplement: Supplementary_material_for_Radiation_Protection_Dosimetry_Manuscript_2019_ncz154 [file supplementary_material_for_radiation_protection_dosimetry_manuscript_2019_ncz154.zip › Supplementary material for Radiation Protection Dosimetry Manuscript 2019/Location1_Figures_2ndWeek/Figure1_DCS_2ndWeek.jpg]

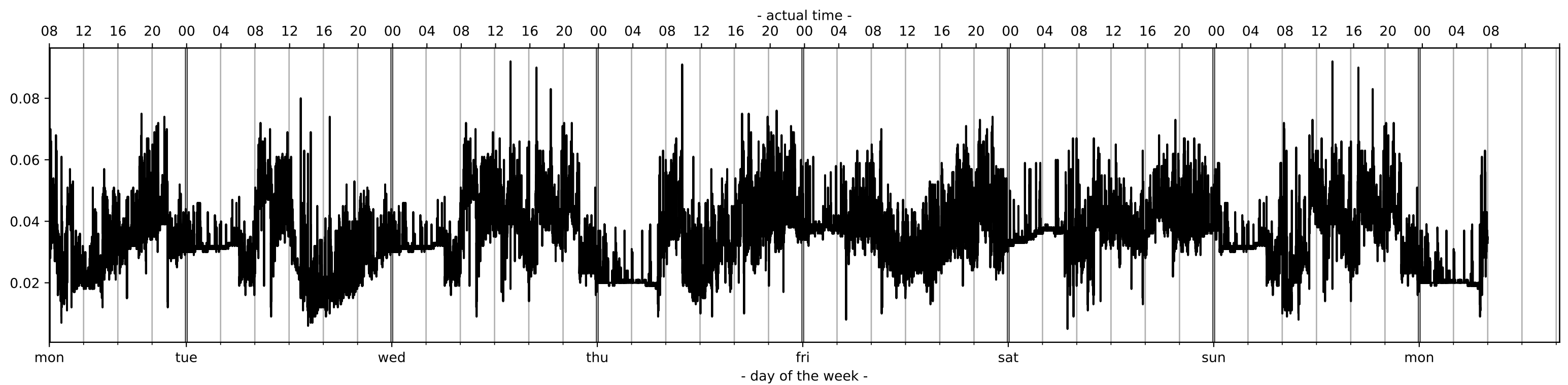

Supplement: Supplementary_material_for_Radiation_Protection_Dosimetry_Manuscript_2019_ncz154 [file supplementary_material_for_radiation_protection_dosimetry_manuscript_2019_ncz154.zip › Supplementary material for Radiation Protection Dosimetry Manuscript 2019/Location1_Figures_2ndWeek/Figure1_DCS_2ndWeek.pdf]

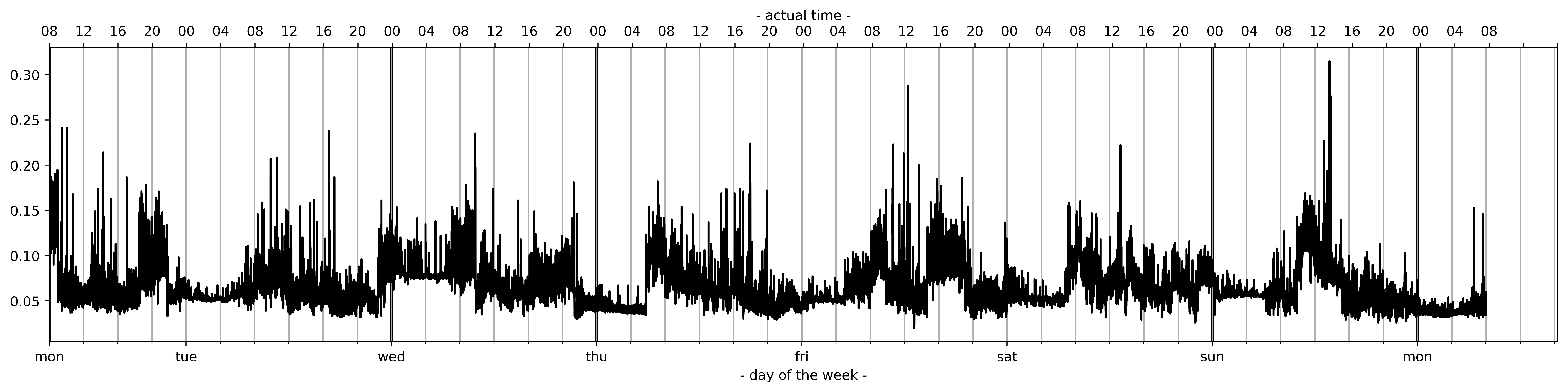

Supplement: Supplementary_material_for_Radiation_Protection_Dosimetry_Manuscript_2019_ncz154 [file supplementary_material_for_radiation_protection_dosimetry_manuscript_2019_ncz154.zip › Supplementary material for Radiation Protection Dosimetry Manuscript 2019/Location1_Figures_2ndWeek/Figure1_GSM_2ndWeek.jpg]

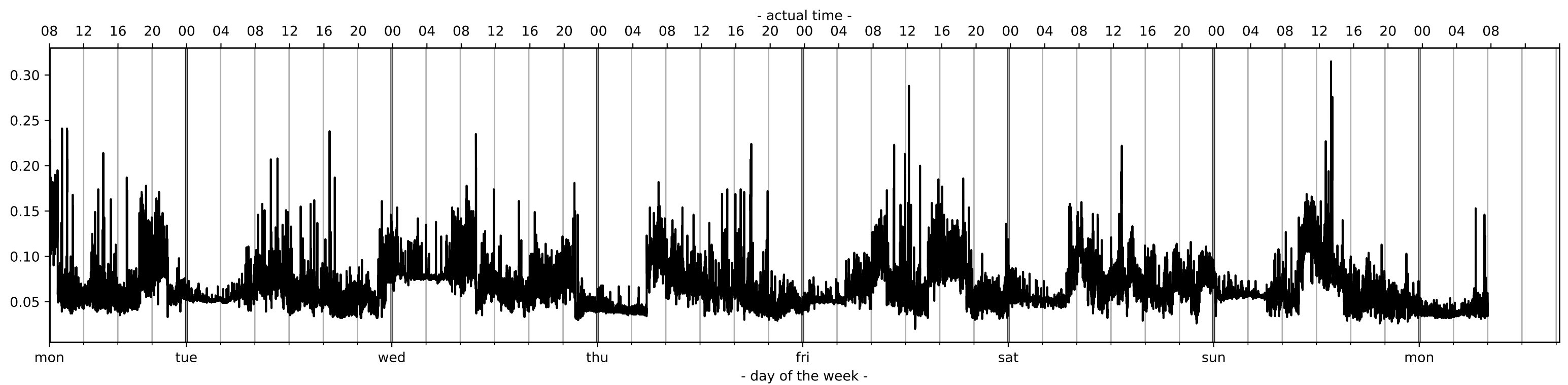

Supplement: Supplementary_material_for_Radiation_Protection_Dosimetry_Manuscript_2019_ncz154 [file supplementary_material_for_radiation_protection_dosimetry_manuscript_2019_ncz154.zip › Supplementary material for Radiation Protection Dosimetry Manuscript 2019/Location1_Figures_2ndWeek/Figure1_GSM_2ndWeek.pdf]

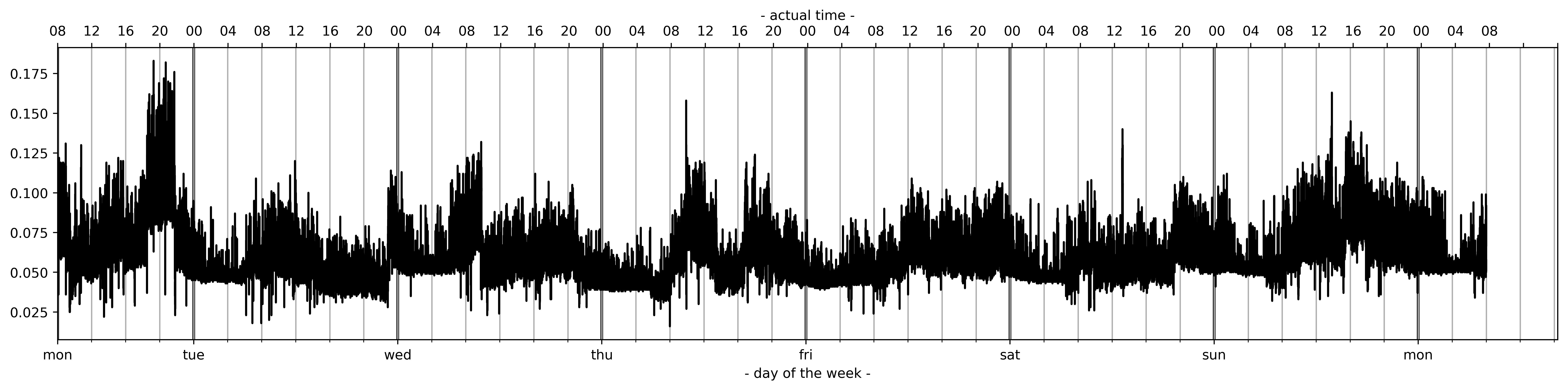

Supplement: Supplementary_material_for_Radiation_Protection_Dosimetry_Manuscript_2019_ncz154 [file supplementary_material_for_radiation_protection_dosimetry_manuscript_2019_ncz154.zip › Supplementary material for Radiation Protection Dosimetry Manuscript 2019/Location1_Figures_2ndWeek/Figure1_UMTS_2ndWeek.jpg]

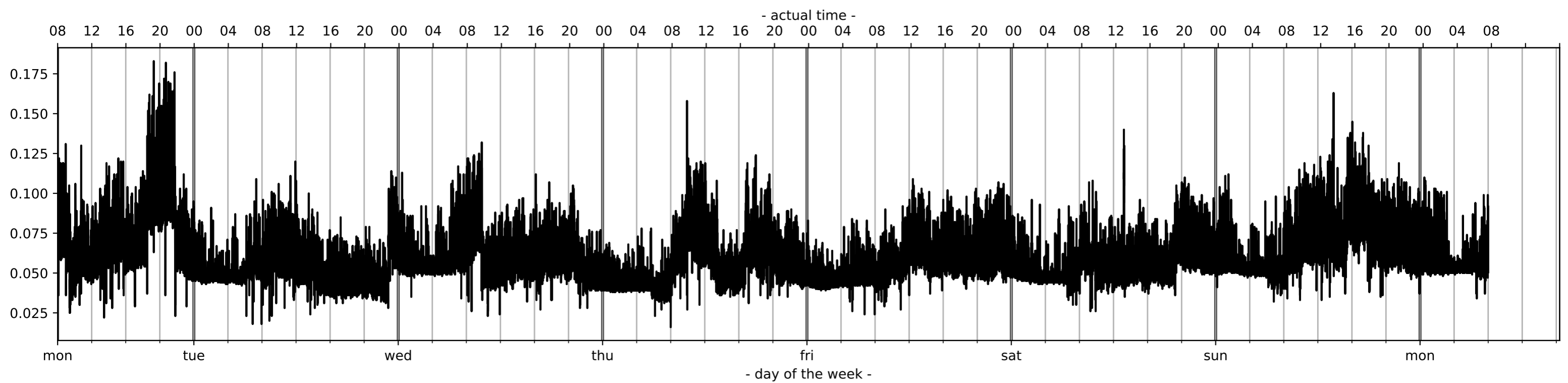

Supplement: Supplementary_material_for_Radiation_Protection_Dosimetry_Manuscript_2019_ncz154 [file supplementary_material_for_radiation_protection_dosimetry_manuscript_2019_ncz154.zip › Supplementary material for Radiation Protection Dosimetry Manuscript 2019/Location1_Figures_2ndWeek/Figure1_UMTS_2ndWeek.pdf]

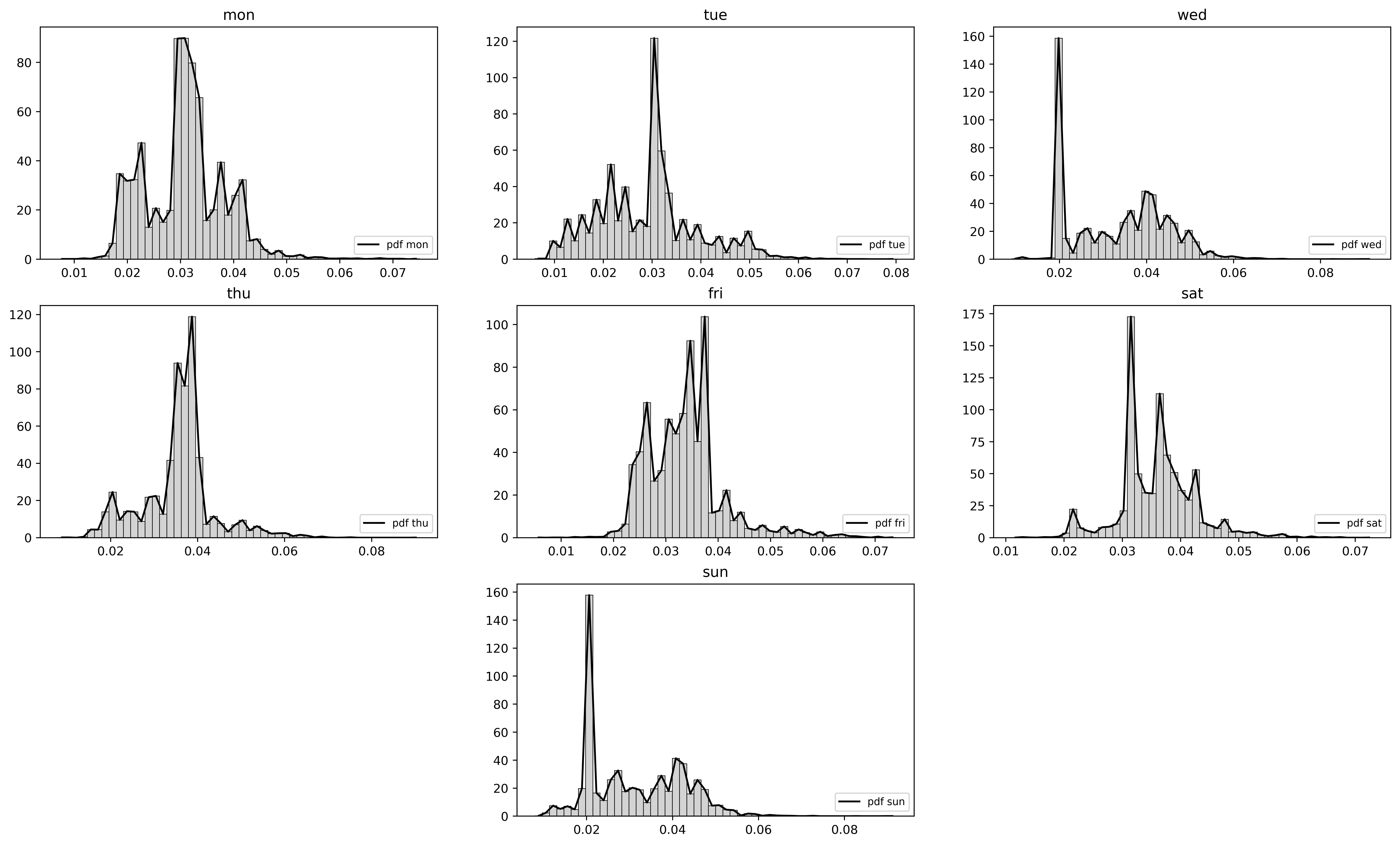

Supplement: Supplementary_material_for_Radiation_Protection_Dosimetry_Manuscript_2019_ncz154 [file supplementary_material_for_radiation_protection_dosimetry_manuscript_2019_ncz154.zip › Supplementary material for Radiation Protection Dosimetry Manuscript 2019/Location1_Figures_2ndWeek/Figure2_DCS_2ndWeek.jpg]

mon

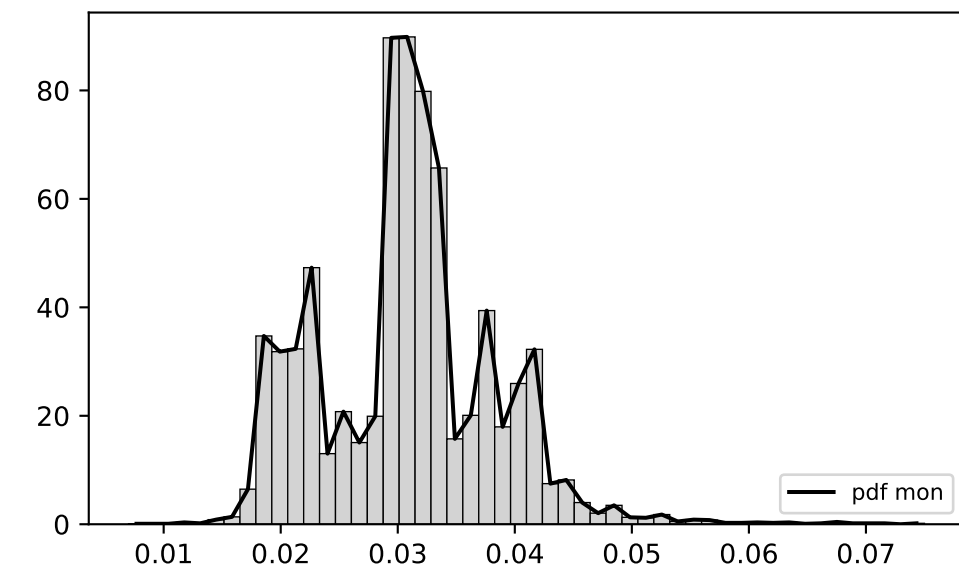

tue

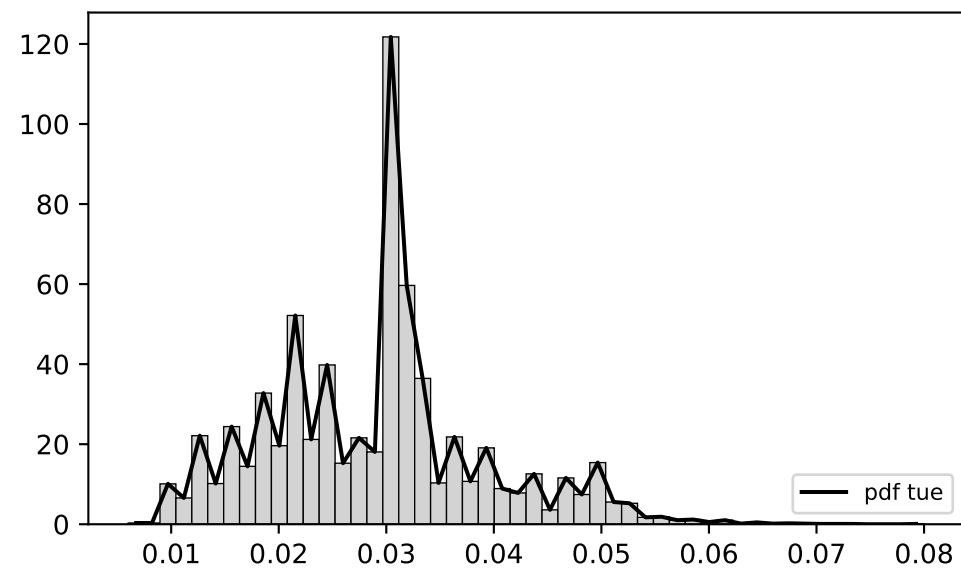

wed

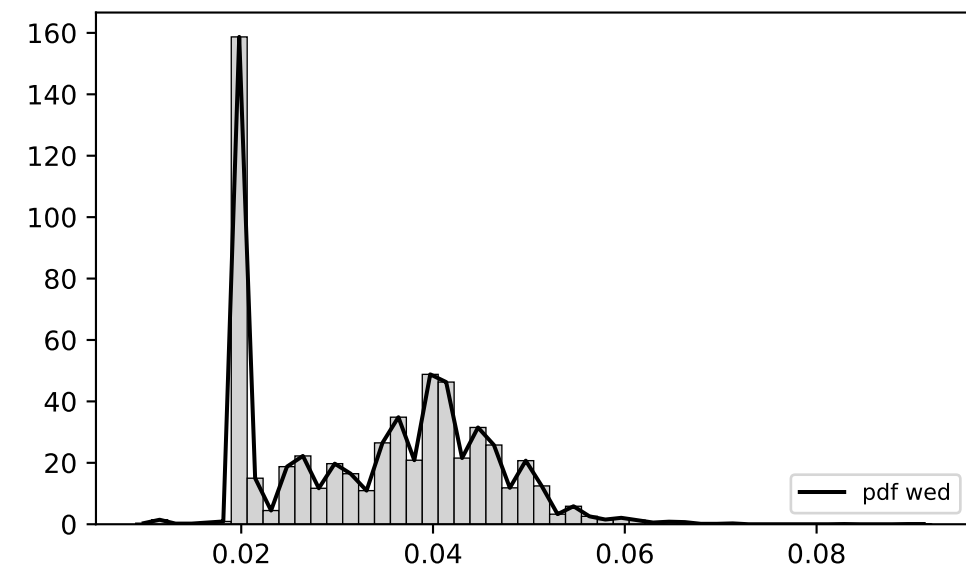

thu

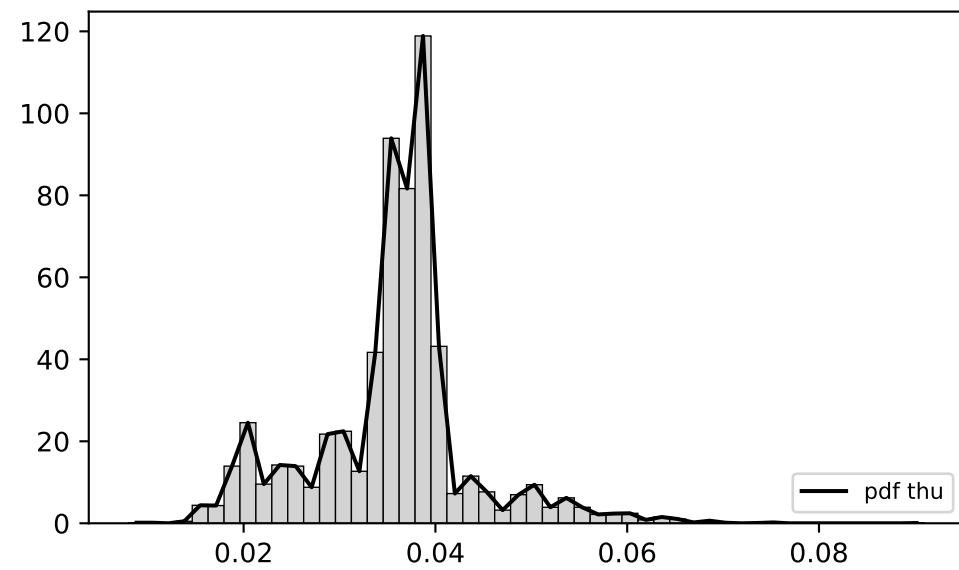

fri

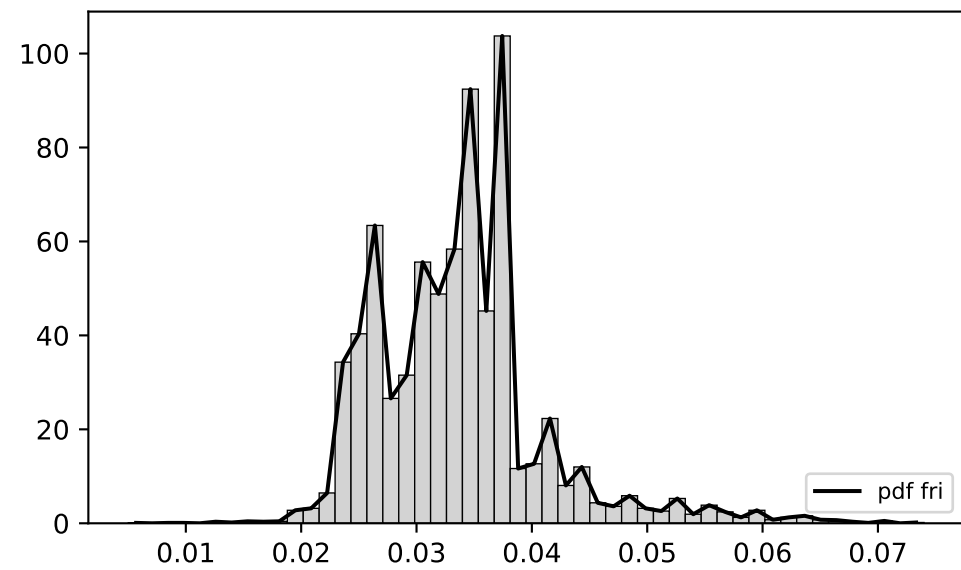

sat

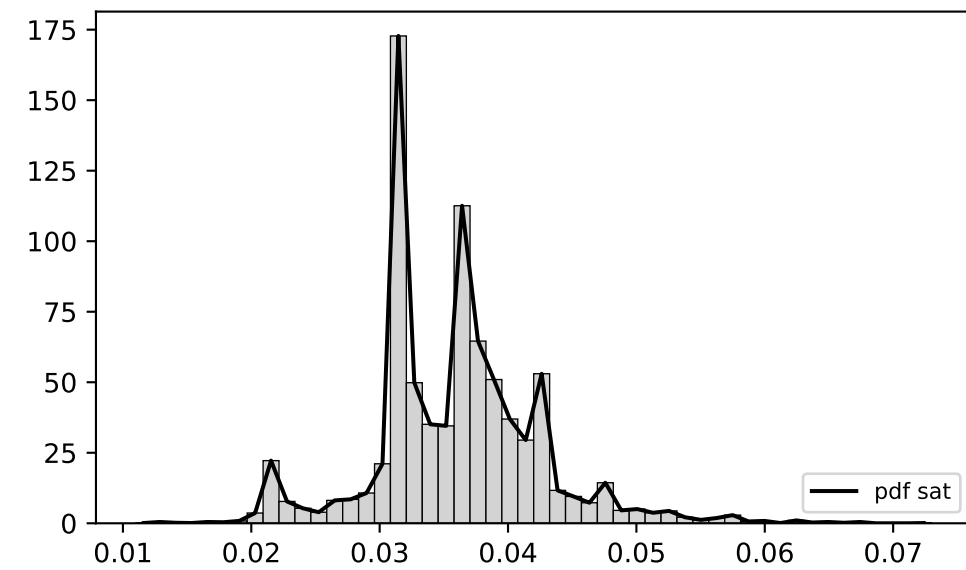

sun

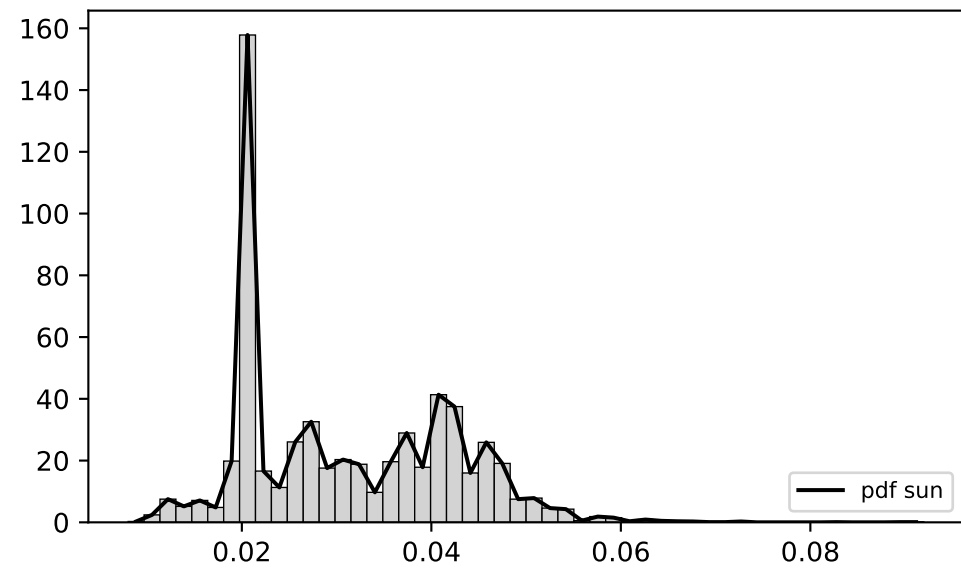

Supplement: Supplementary_material_for_Radiation_Protection_Dosimetry_Manuscript_2019_ncz154 [file supplementary_material_for_radiation_protection_dosimetry_manuscript_2019_ncz154.zip › Supplementary material for Radiation Protection Dosimetry Manuscript 2019/Location1_Figures_2ndWeek/Figure2_DCS_2ndWeek.pdf]

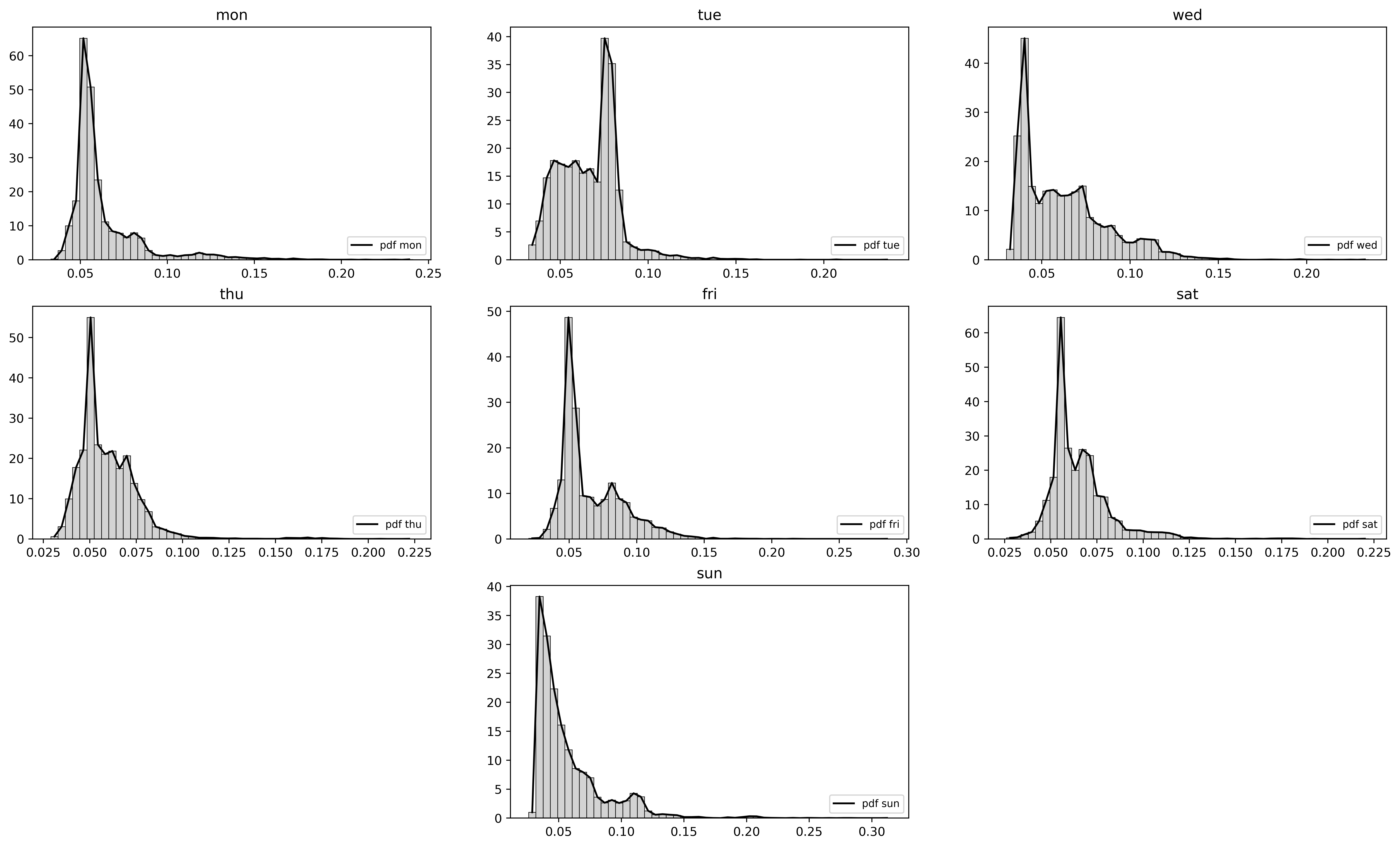

Supplement: Supplementary_material_for_Radiation_Protection_Dosimetry_Manuscript_2019_ncz154 [file supplementary_material_for_radiation_protection_dosimetry_manuscript_2019_ncz154.zip › Supplementary material for Radiation Protection Dosimetry Manuscript 2019/Location1_Figures_2ndWeek/Figure2_GSM_2ndWeek.jpg]

mon

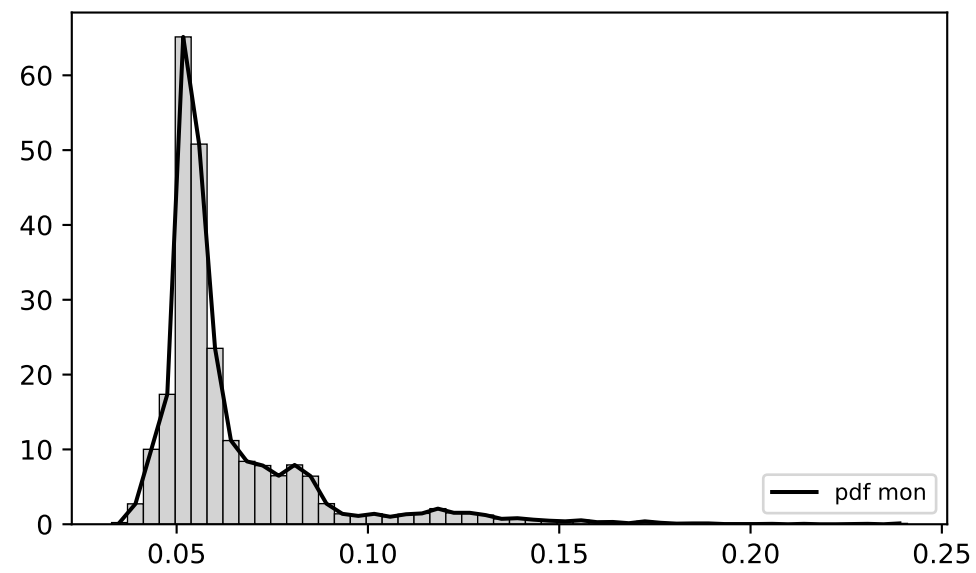

tue

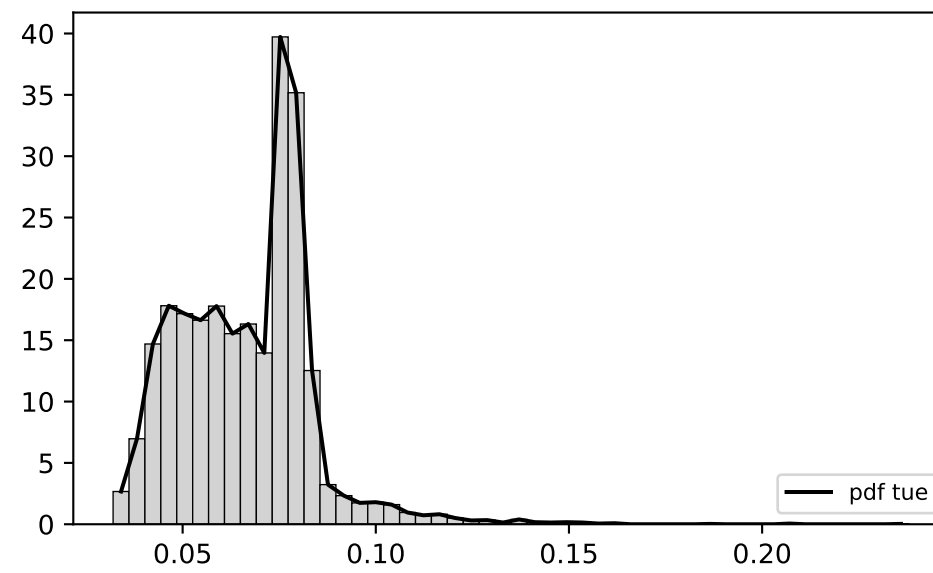

wed

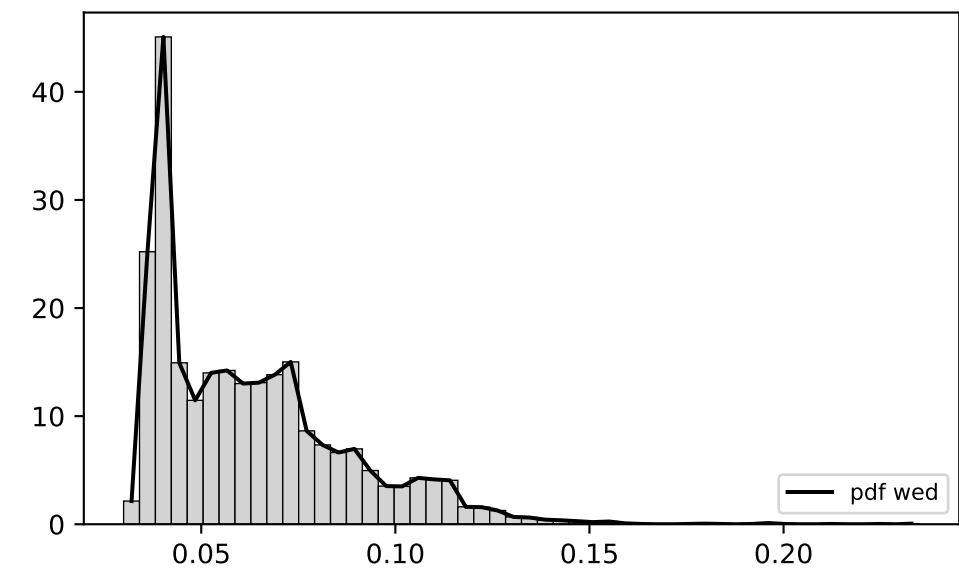

thu

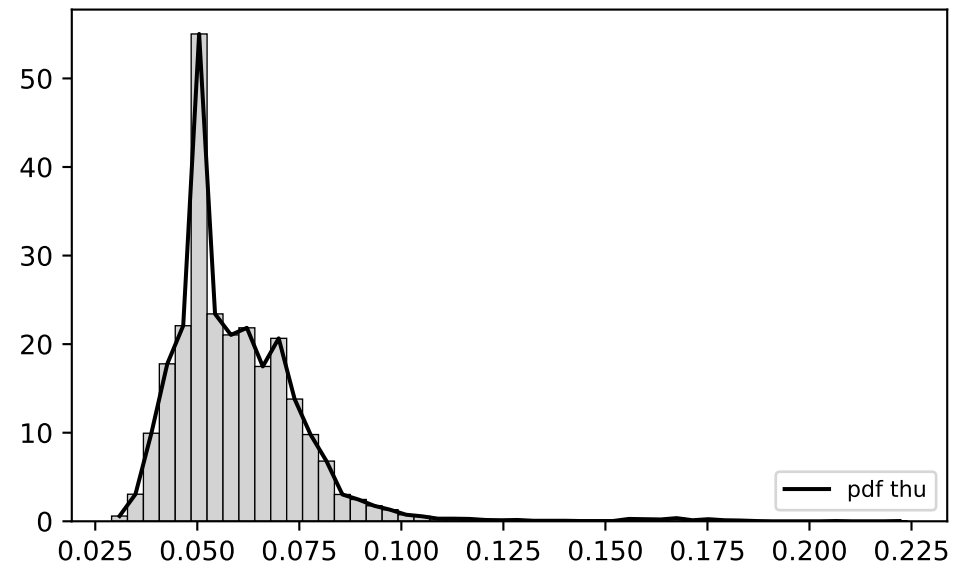

fri

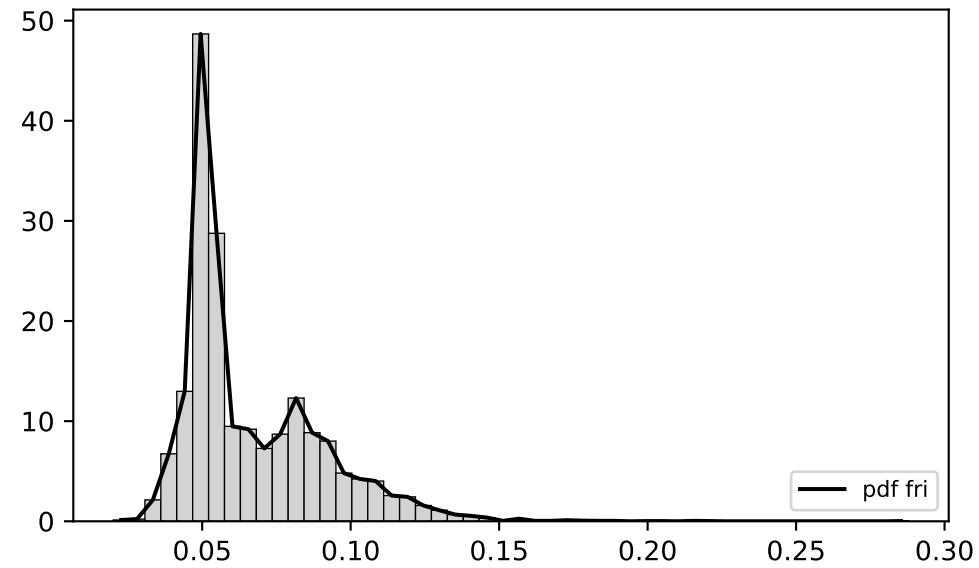

sat

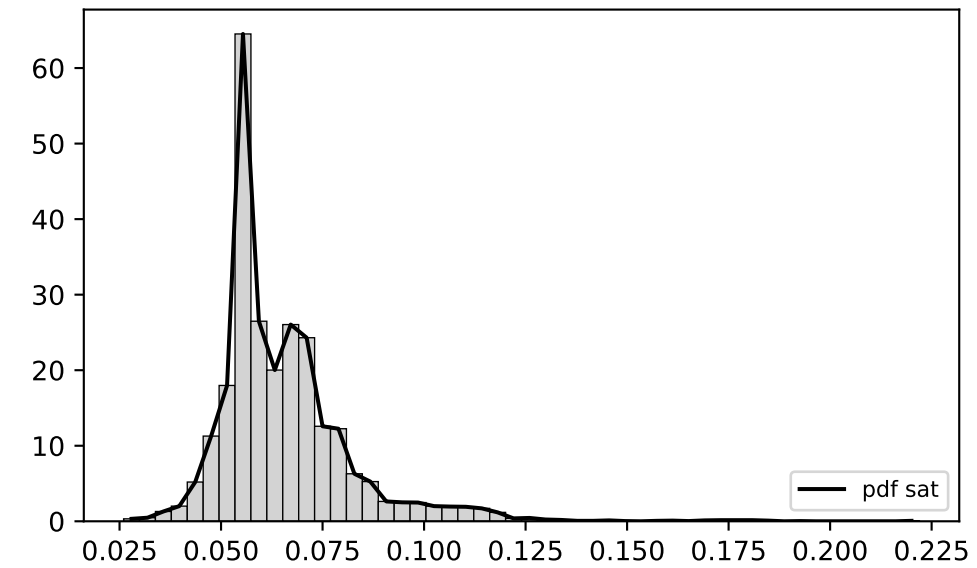

sun

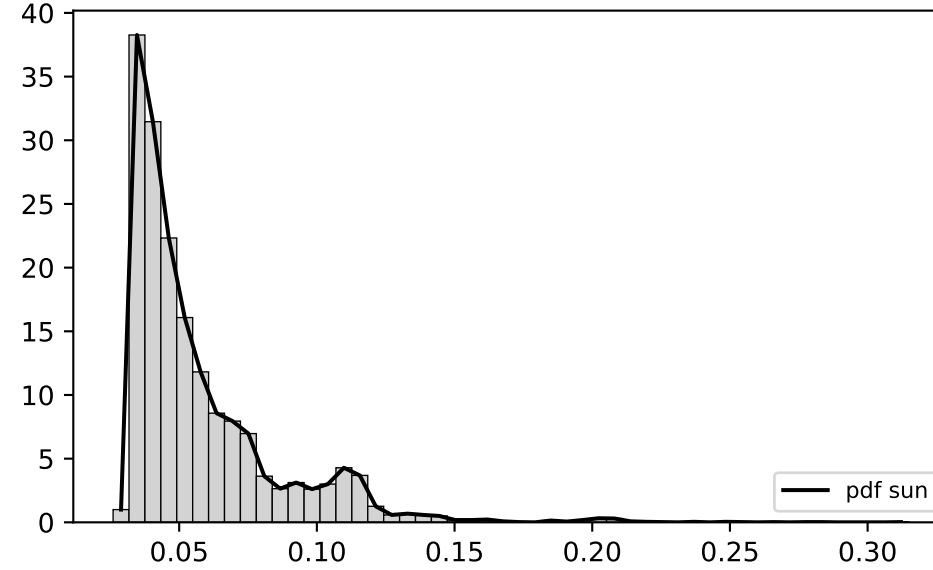

Supplement: Supplementary_material_for_Radiation_Protection_Dosimetry_Manuscript_2019_ncz154 [file supplementary_material_for_radiation_protection_dosimetry_manuscript_2019_ncz154.zip › Supplementary material for Radiation Protection Dosimetry Manuscript 2019/Location1_Figures_2ndWeek/Figure2_GSM_2ndWeek.pdf]

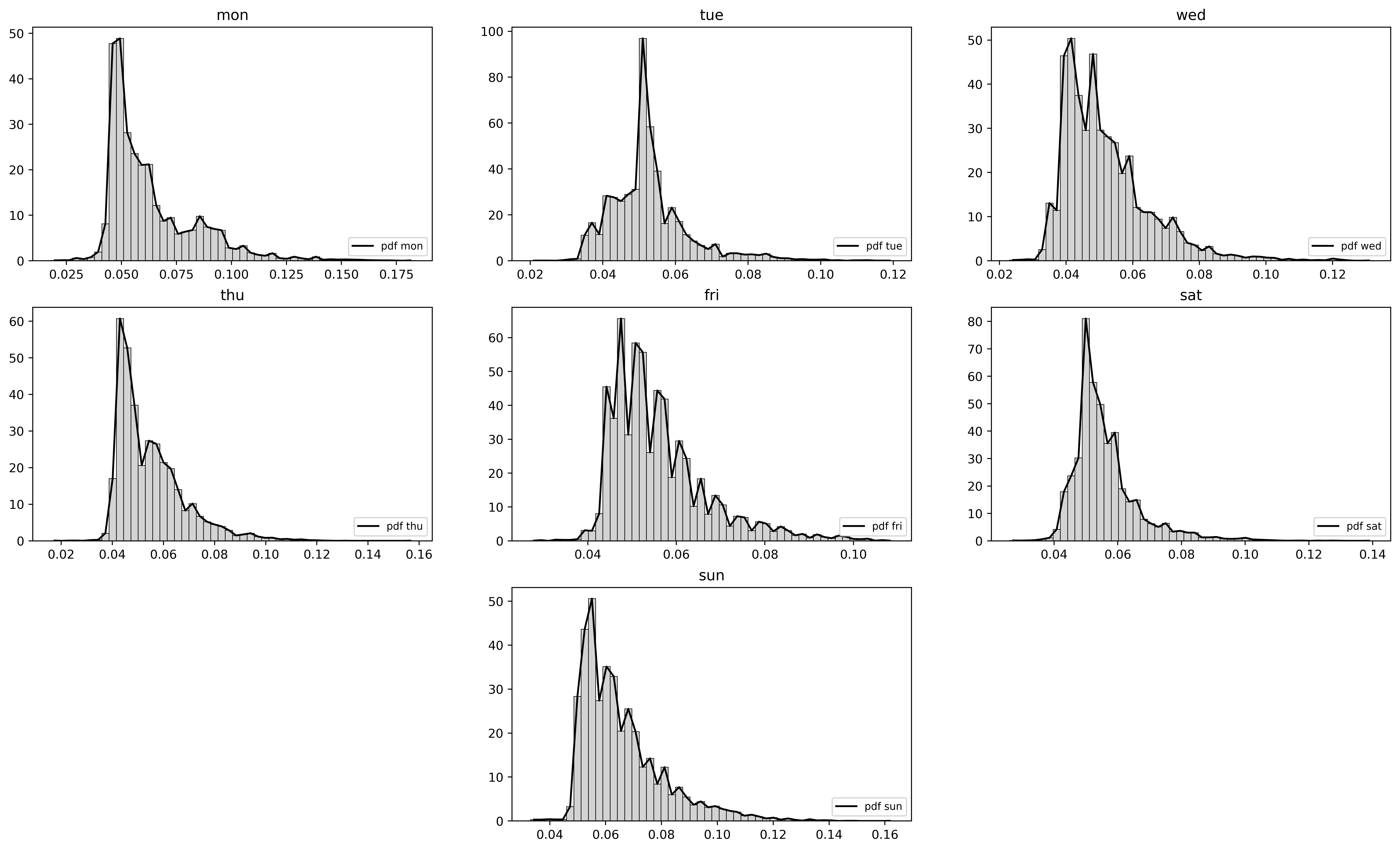

Supplement: Supplementary_material_for_Radiation_Protection_Dosimetry_Manuscript_2019_ncz154 [file supplementary_material_for_radiation_protection_dosimetry_manuscript_2019_ncz154.zip › Supplementary material for Radiation Protection Dosimetry Manuscript 2019/Location1_Figures_2ndWeek/Figure2_UMTS_2ndWeek.jpg]

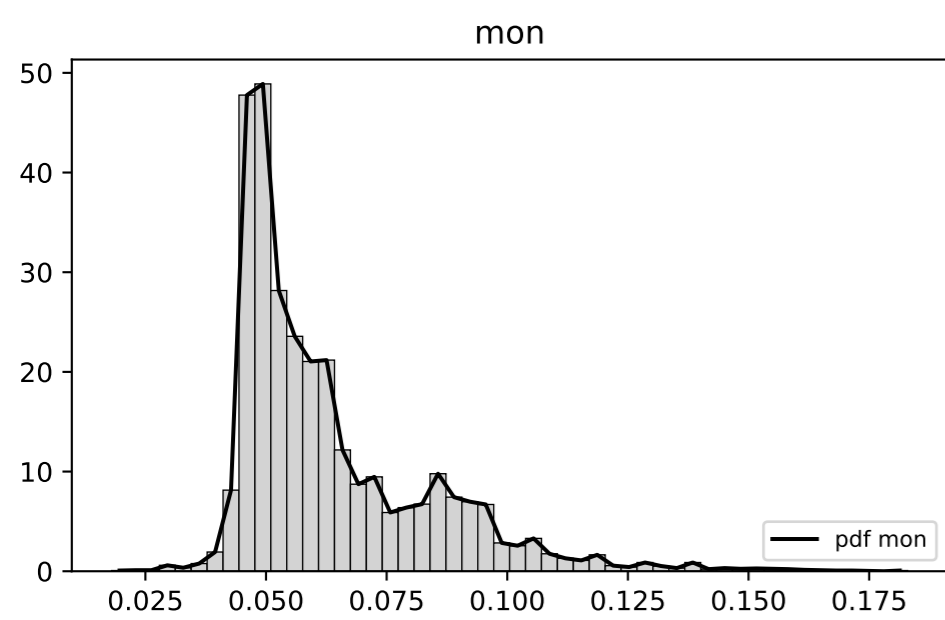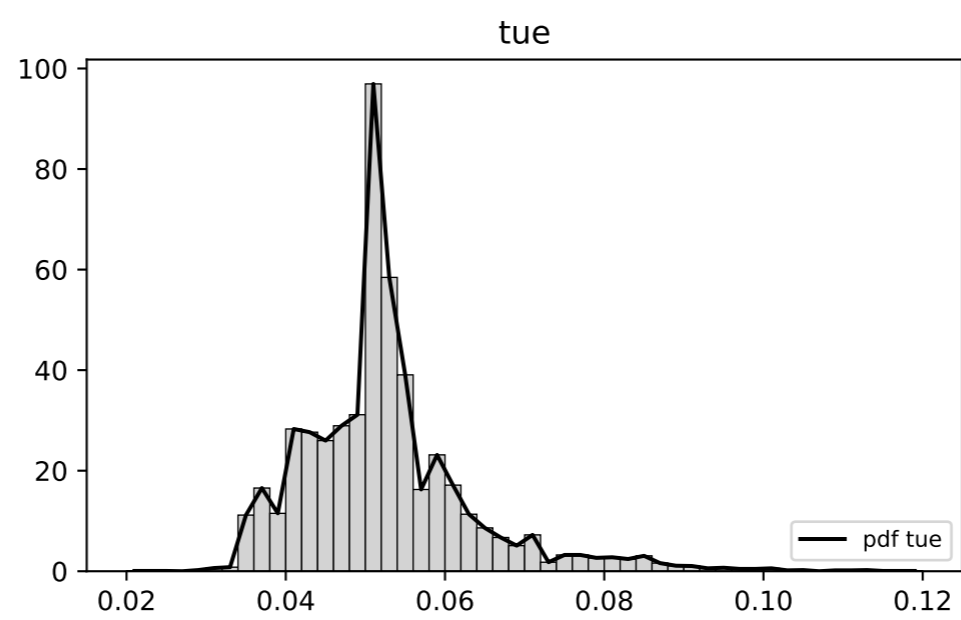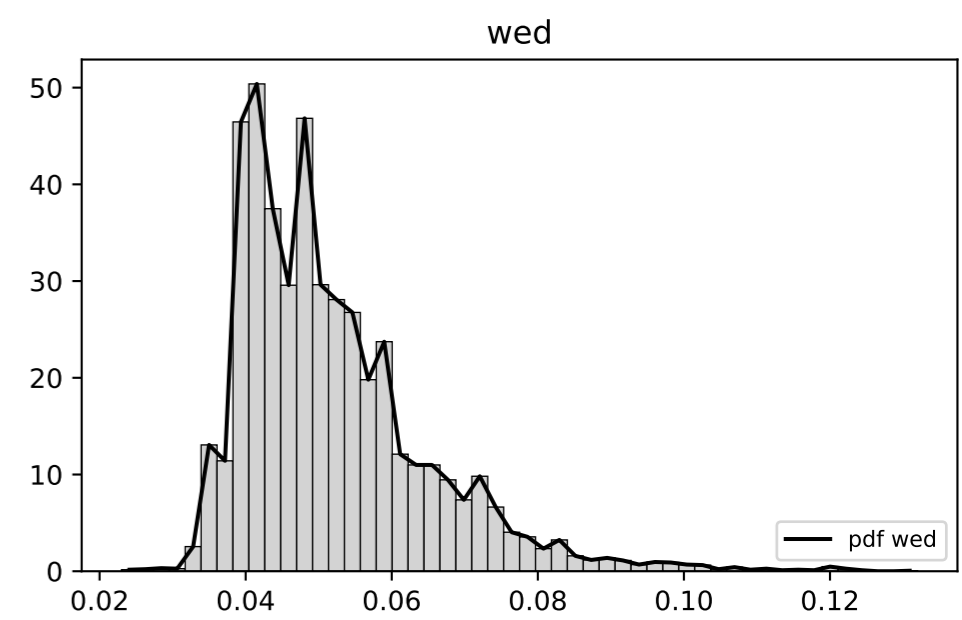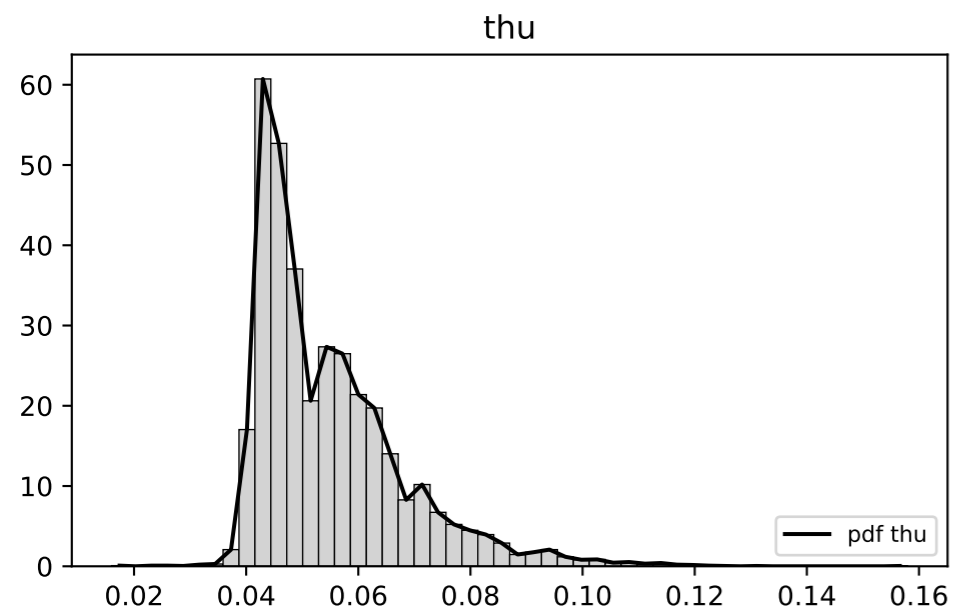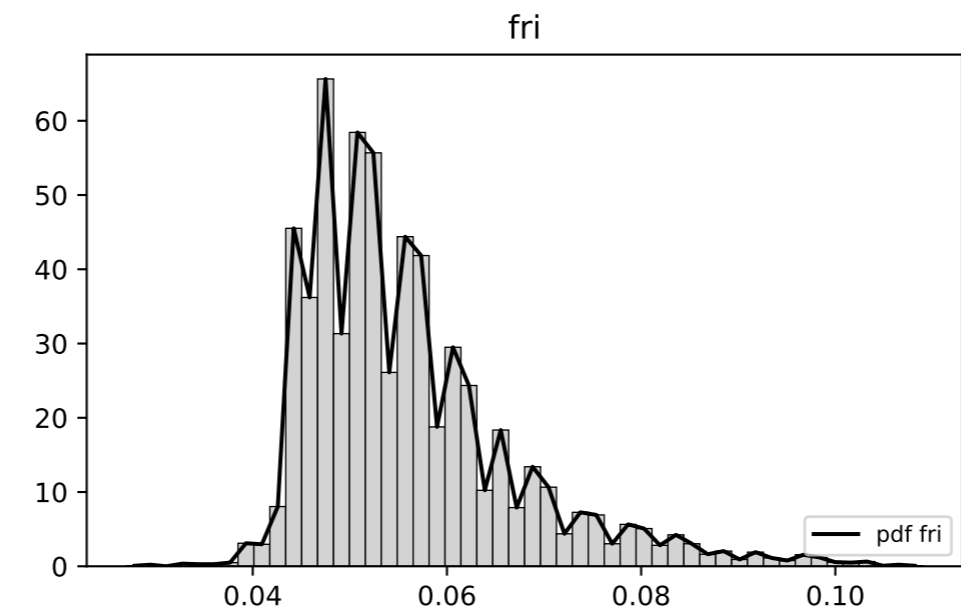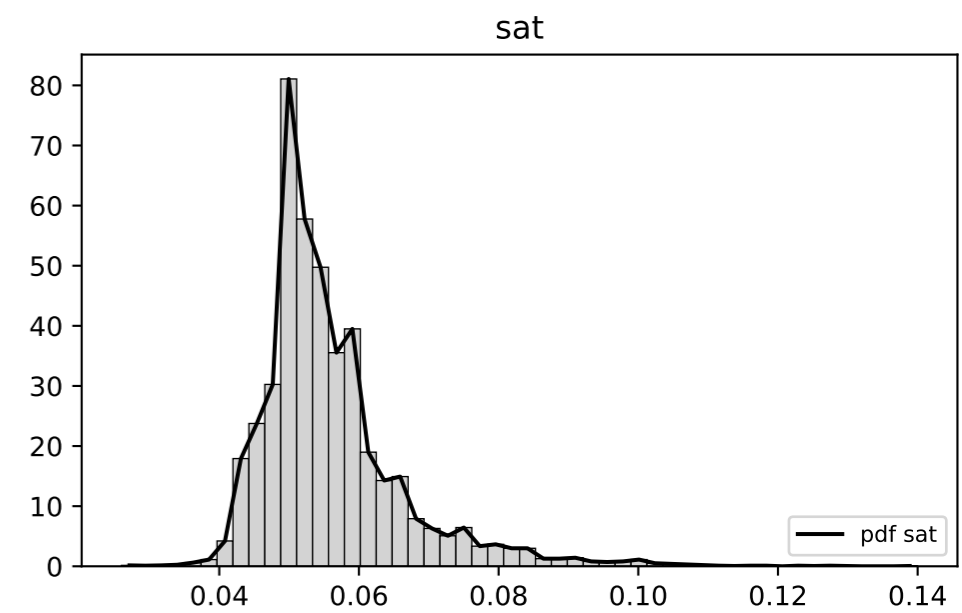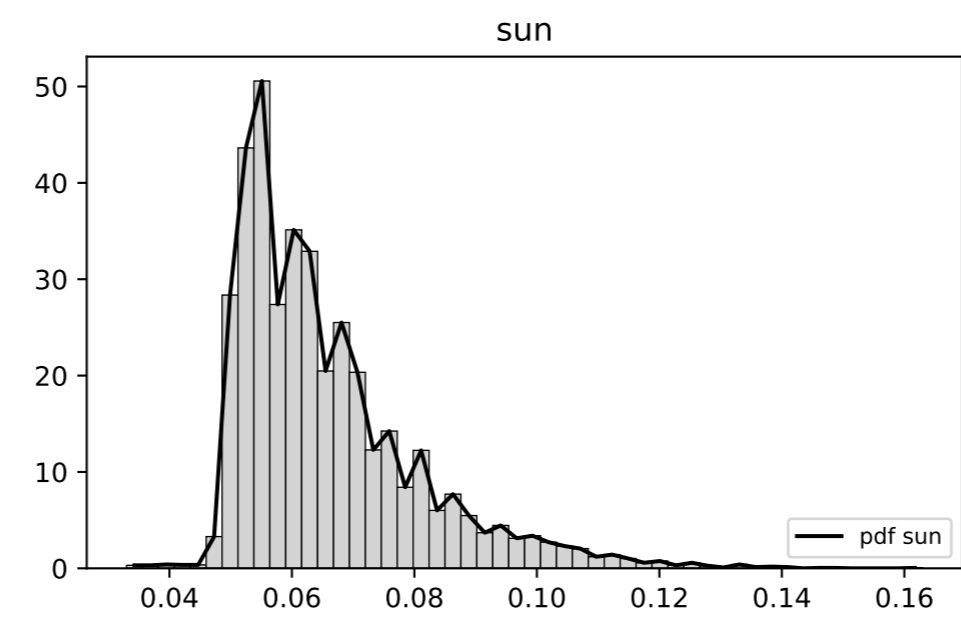

Supplement: Supplementary_material_for_Radiation_Protection_Dosimetry_Manuscript_2019_ncz154 [file supplementary_material_for_radiation_protection_dosimetry_manuscript_2019_ncz154.zip › Supplementary material for Radiation Protection Dosimetry Manuscript 2019/Location1_Figures_2ndWeek/Figure2_UMTS_2ndWeek.pdf]

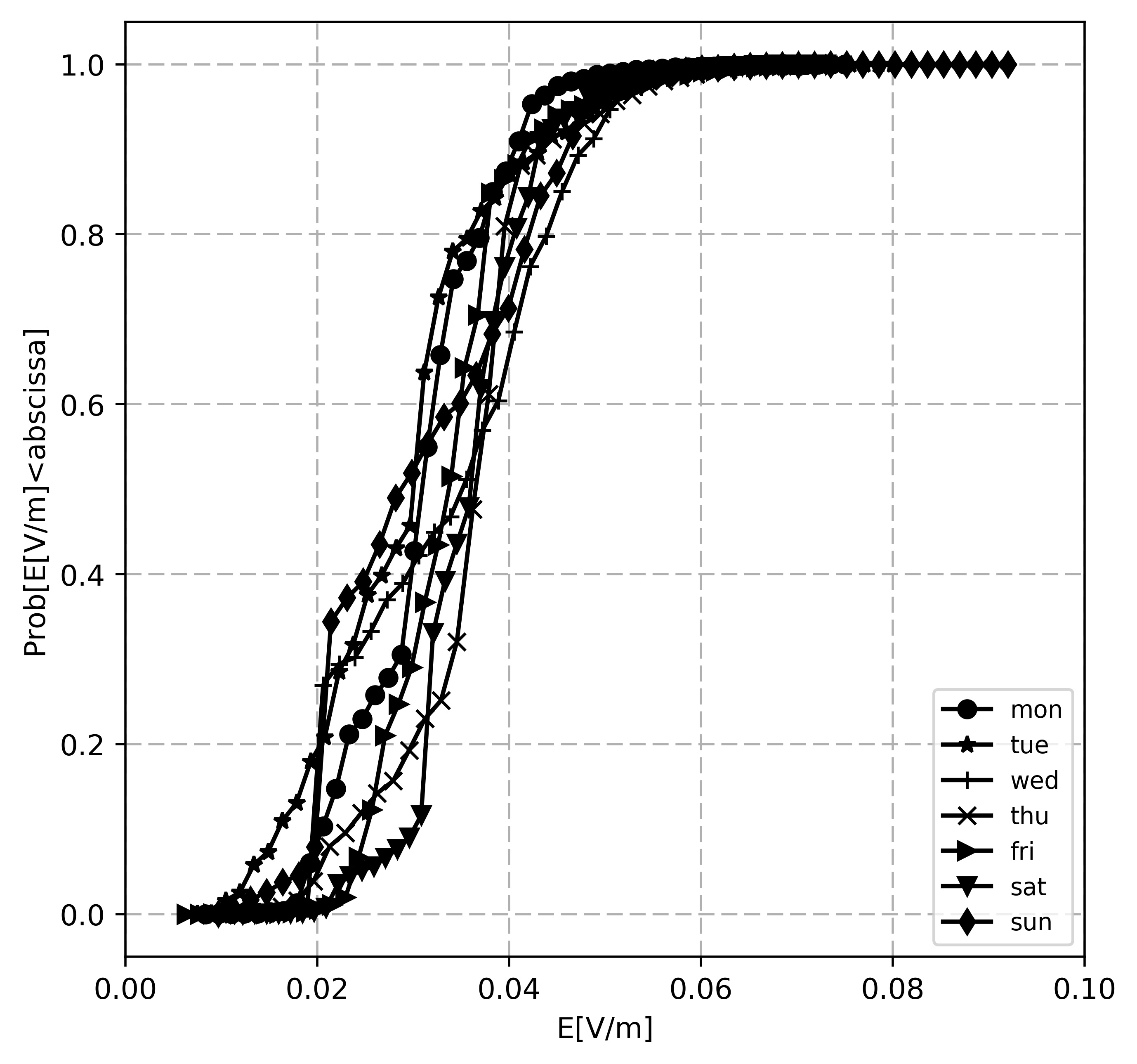

Supplement: Supplementary_material_for_Radiation_Protection_Dosimetry_Manuscript_2019_ncz154 [file supplementary_material_for_radiation_protection_dosimetry_manuscript_2019_ncz154.zip › Supplementary material for Radiation Protection Dosimetry Manuscript 2019/Location1_Figures_2ndWeek/Figure3_DCS_2ndWeek.jpg]

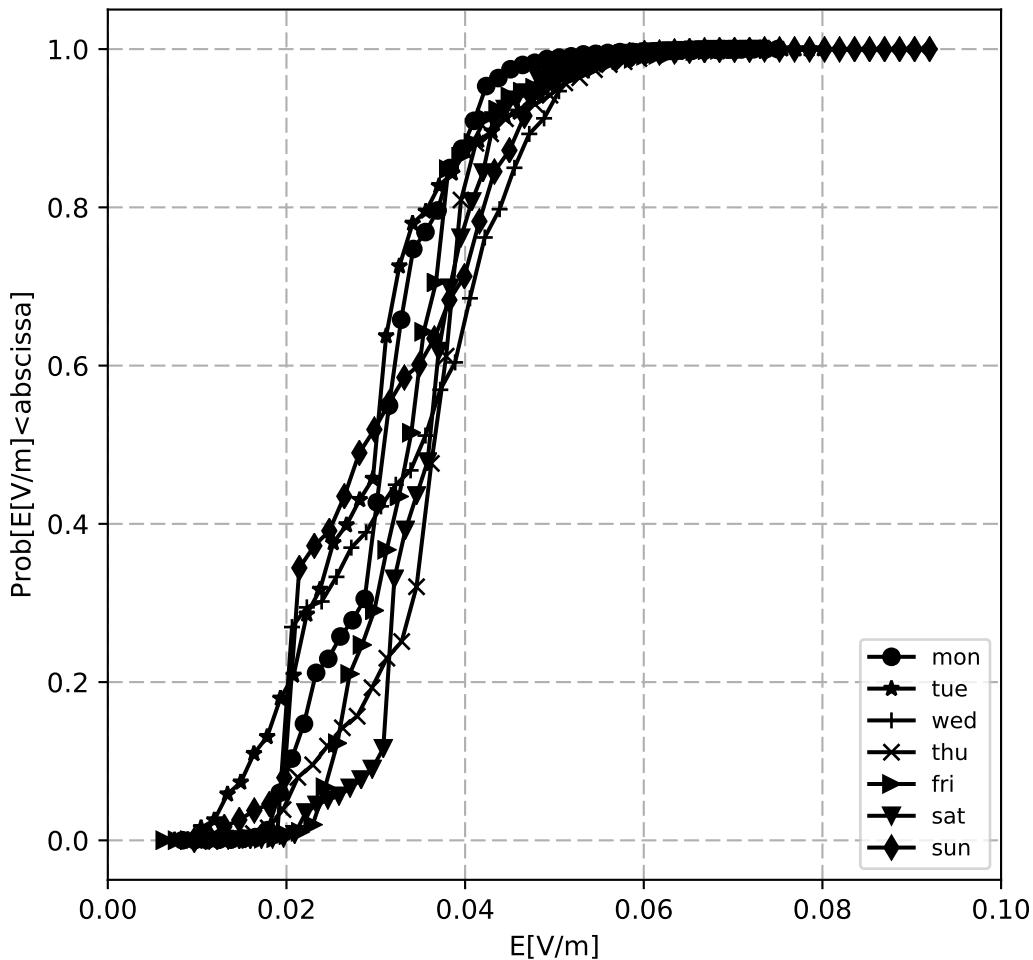

Supplement: Supplementary_material_for_Radiation_Protection_Dosimetry_Manuscript_2019_ncz154 [file supplementary_material_for_radiation_protection_dosimetry_manuscript_2019_ncz154.zip › Supplementary material for Radiation Protection Dosimetry Manuscript 2019/Location1_Figures_2ndWeek/Figure3_DCS_2ndWeek.pdf]

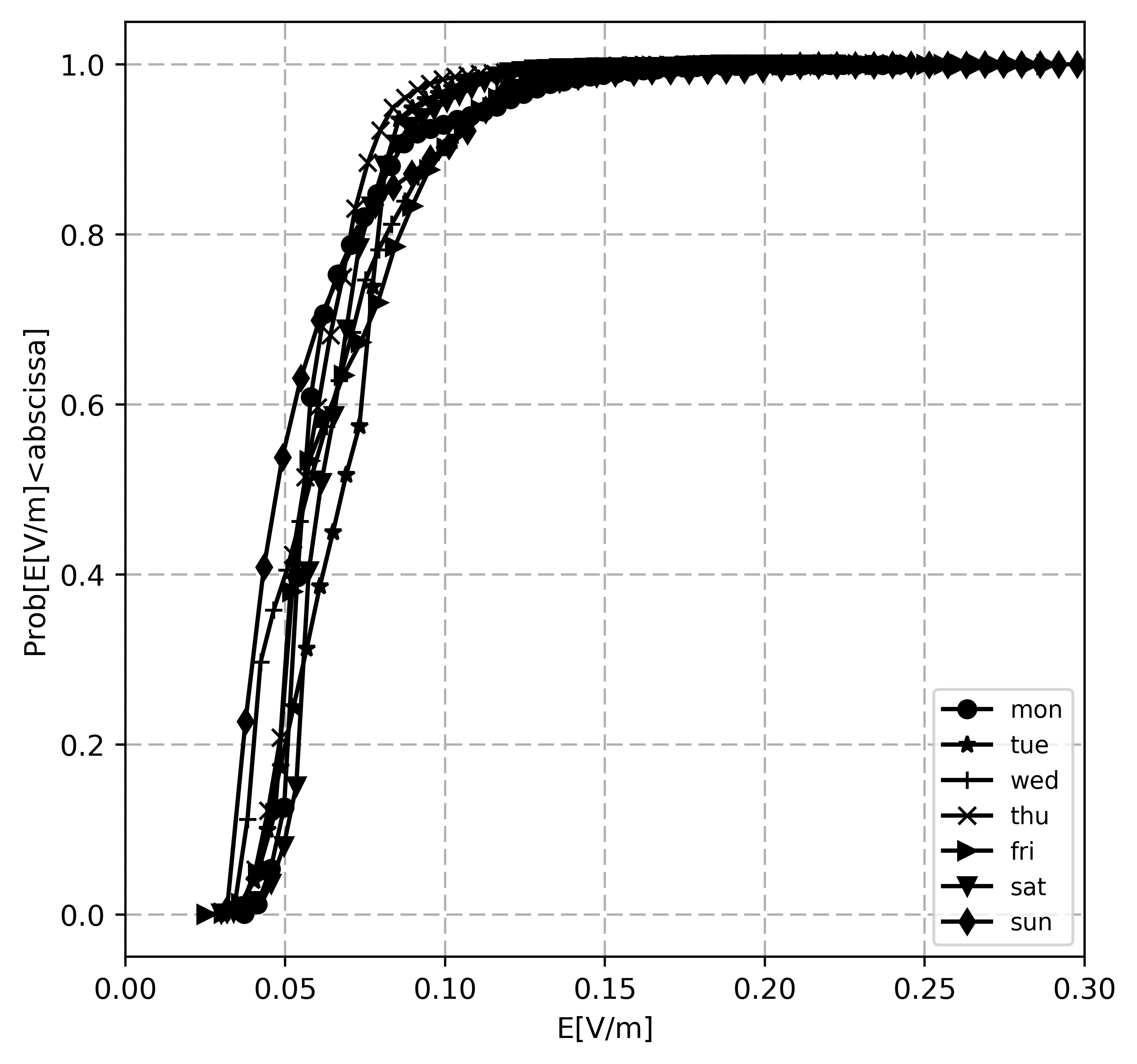

Supplement: Supplementary_material_for_Radiation_Protection_Dosimetry_Manuscript_2019_ncz154 [file supplementary_material_for_radiation_protection_dosimetry_manuscript_2019_ncz154.zip › Supplementary material for Radiation Protection Dosimetry Manuscript 2019/Location1_Figures_2ndWeek/Figure3_GSM_2ndWeek.jpg]

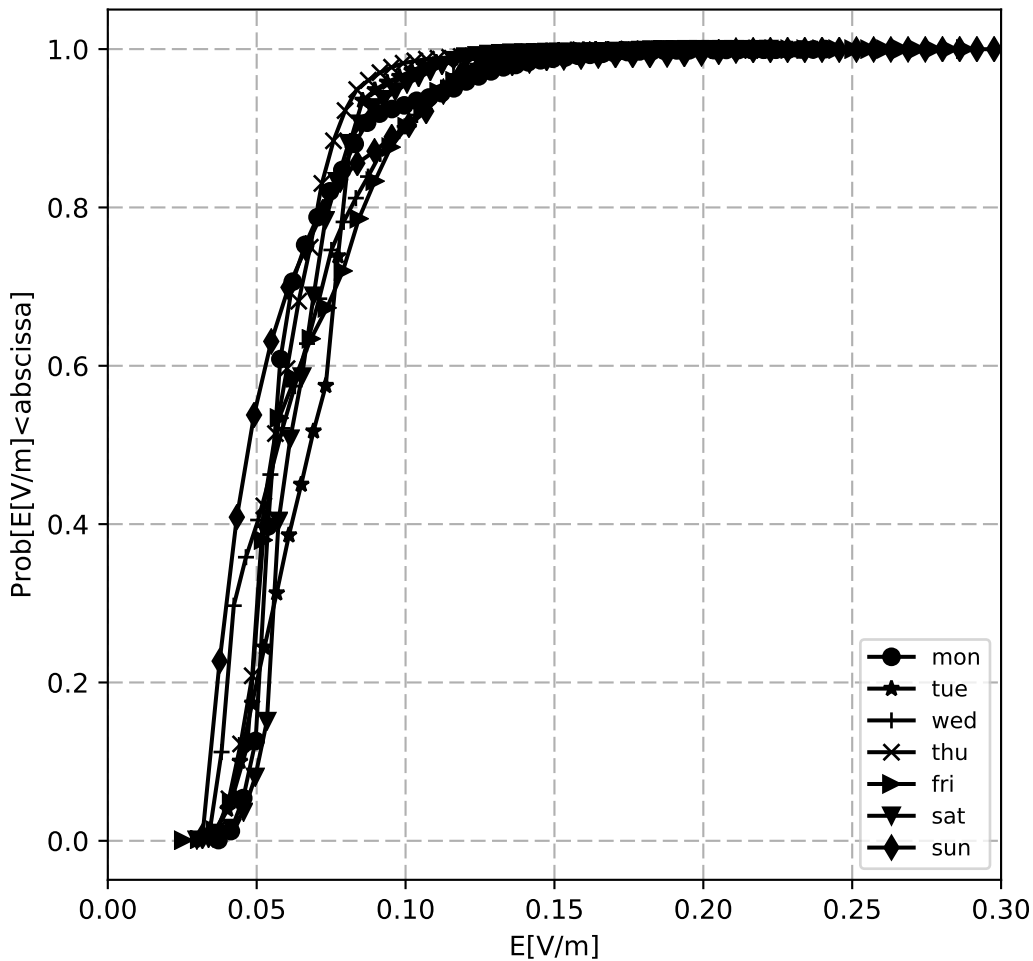

Supplement: Supplementary_material_for_Radiation_Protection_Dosimetry_Manuscript_2019_ncz154 [file supplementary_material_for_radiation_protection_dosimetry_manuscript_2019_ncz154.zip › Supplementary material for Radiation Protection Dosimetry Manuscript 2019/Location1_Figures_2ndWeek/Figure3_GSM_2ndWeek.pdf]

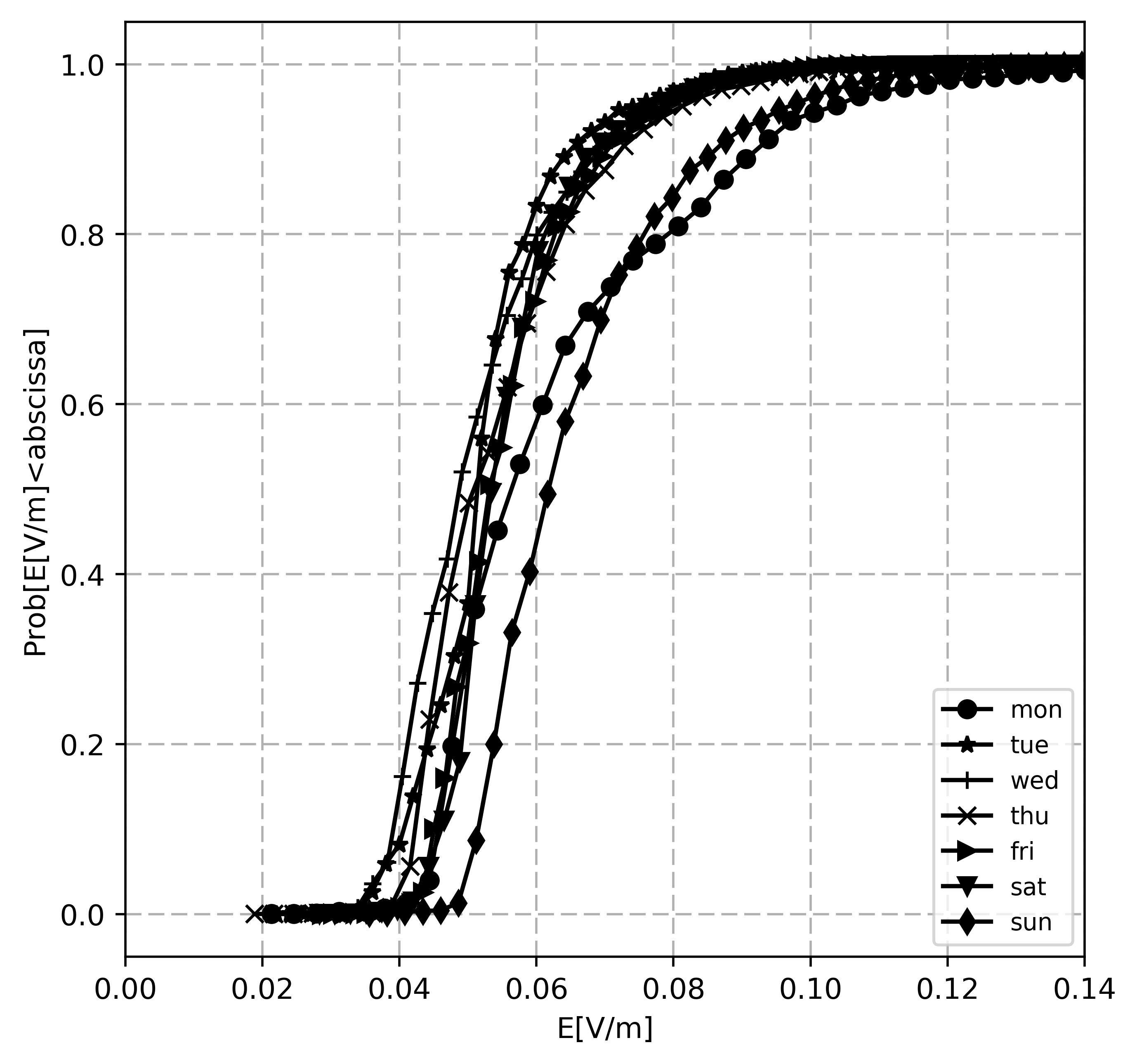

Supplement: Supplementary_material_for_Radiation_Protection_Dosimetry_Manuscript_2019_ncz154 [file supplementary_material_for_radiation_protection_dosimetry_manuscript_2019_ncz154.zip › Supplementary material for Radiation Protection Dosimetry Manuscript 2019/Location1_Figures_2ndWeek/Figure3_UMTS_2ndWeek.jpg]

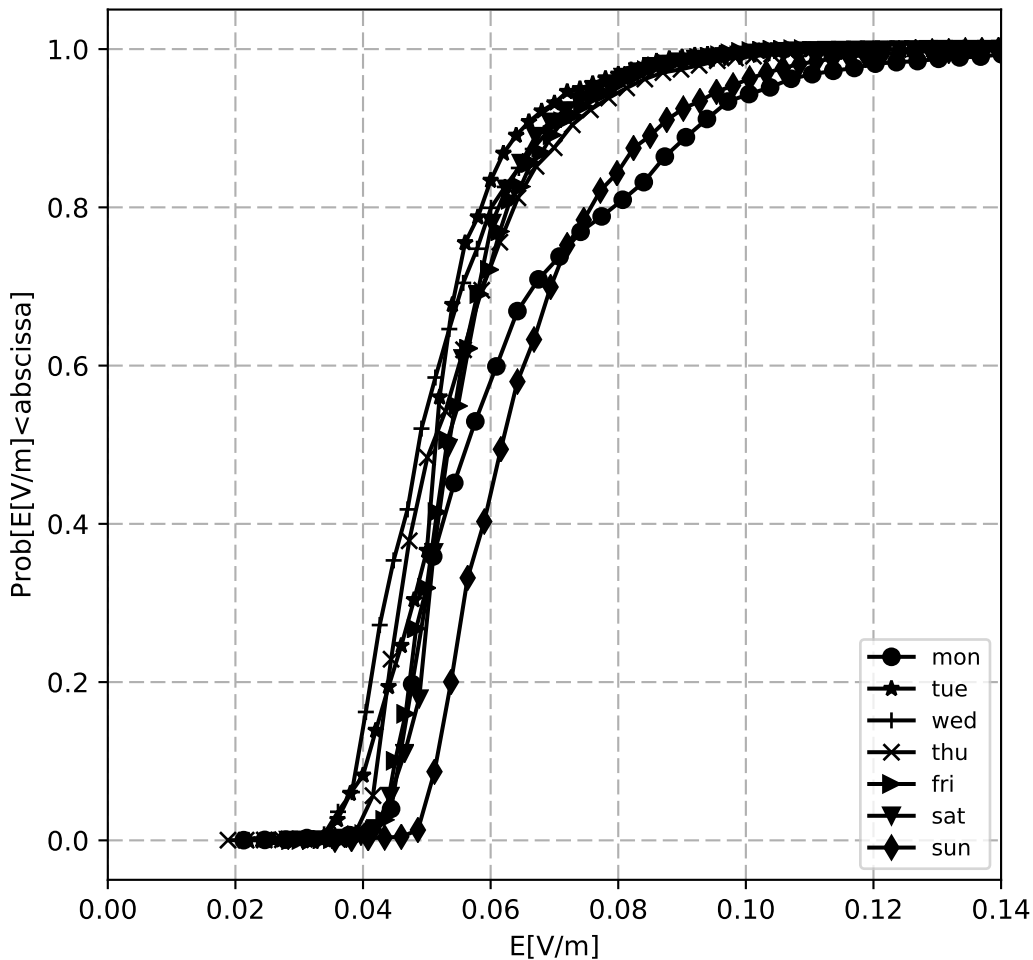

Supplement: Supplementary_material_for_Radiation_Protection_Dosimetry_Manuscript_2019_ncz154 [file supplementary_material_for_radiation_protection_dosimetry_manuscript_2019_ncz154.zip › Supplementary material for Radiation Protection Dosimetry Manuscript 2019/Location1_Figures_2ndWeek/Figure3_UMTS_2ndWeek.pdf]

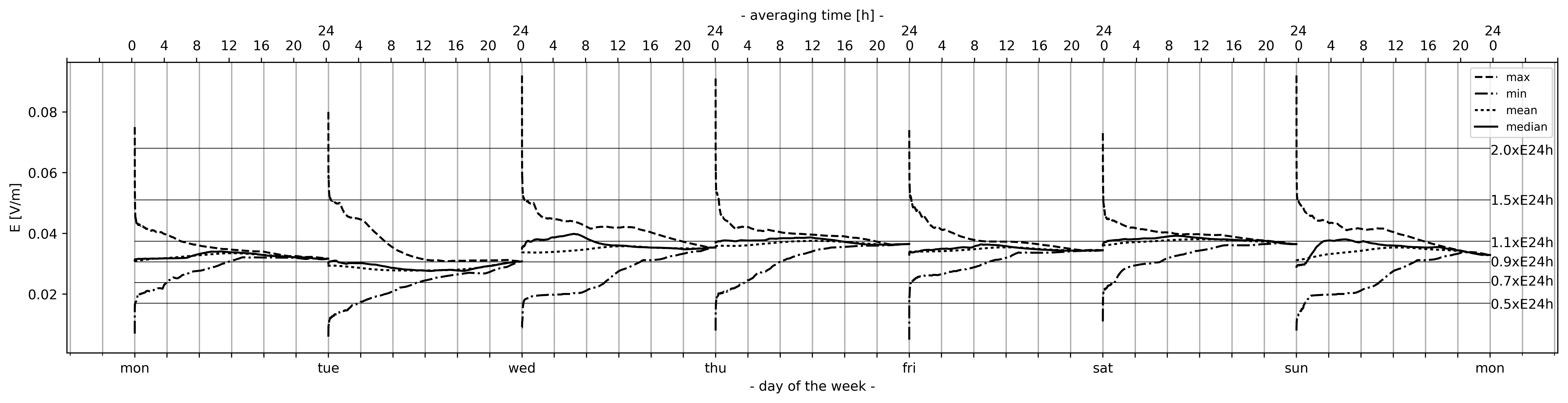

Supplement: Supplementary_material_for_Radiation_Protection_Dosimetry_Manuscript_2019_ncz154 [file supplementary_material_for_radiation_protection_dosimetry_manuscript_2019_ncz154.zip › Supplementary material for Radiation Protection Dosimetry Manuscript 2019/Location1_Figures_2ndWeek/Figure4_DCS_2ndWeek.jpg]

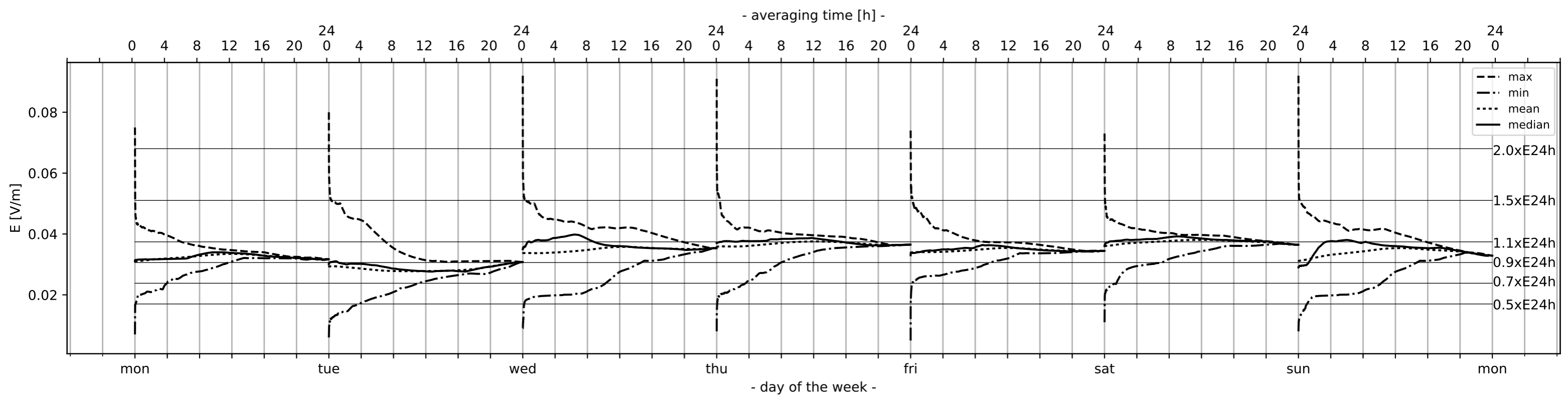

Supplement: Supplementary_material_for_Radiation_Protection_Dosimetry_Manuscript_2019_ncz154 [file supplementary_material_for_radiation_protection_dosimetry_manuscript_2019_ncz154.zip › Supplementary material for Radiation Protection Dosimetry Manuscript 2019/Location1_Figures_2ndWeek/Figure4_DCS_2ndWeek.pdf]

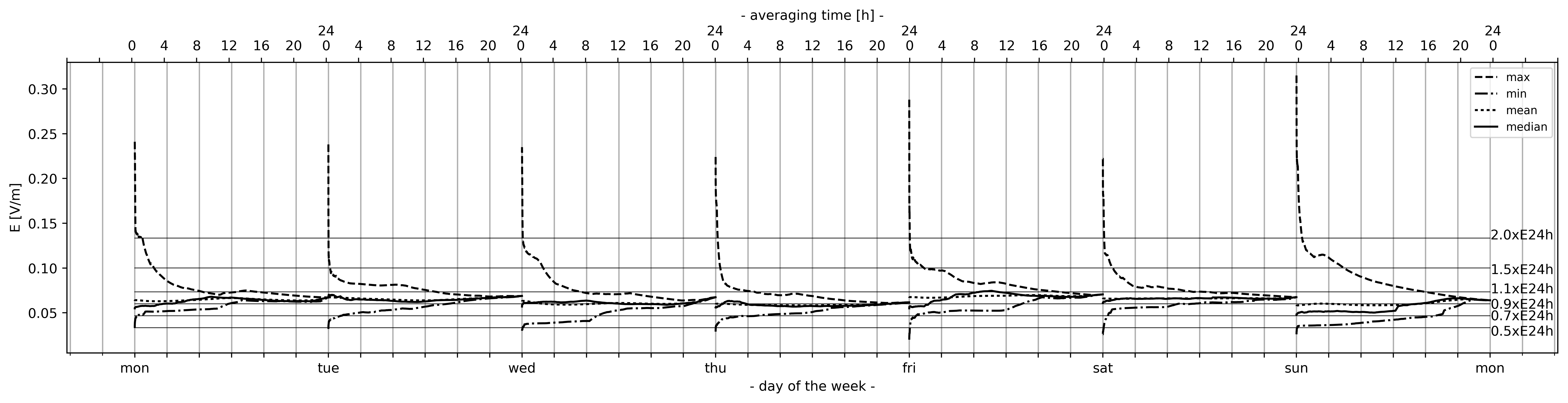

Supplement: Supplementary_material_for_Radiation_Protection_Dosimetry_Manuscript_2019_ncz154 [file supplementary_material_for_radiation_protection_dosimetry_manuscript_2019_ncz154.zip › Supplementary material for Radiation Protection Dosimetry Manuscript 2019/Location1_Figures_2ndWeek/Figure4_GSM_2ndWeek.jpg]

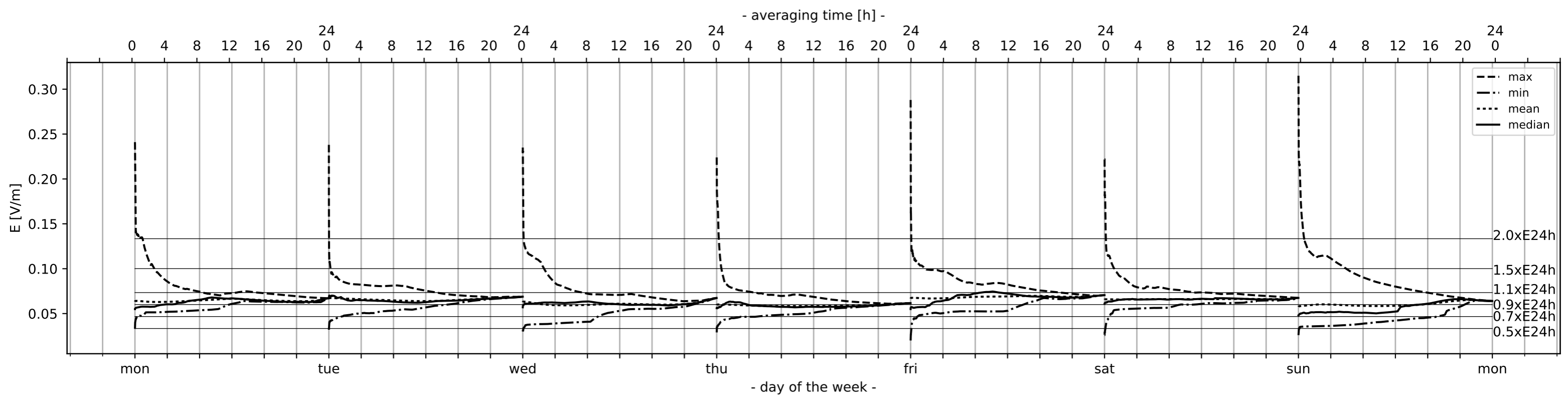

Supplement: Supplementary_material_for_Radiation_Protection_Dosimetry_Manuscript_2019_ncz154 [file supplementary_material_for_radiation_protection_dosimetry_manuscript_2019_ncz154.zip › Supplementary material for Radiation Protection Dosimetry Manuscript 2019/Location1_Figures_2ndWeek/Figure4_GSM_2ndWeek.pdf]

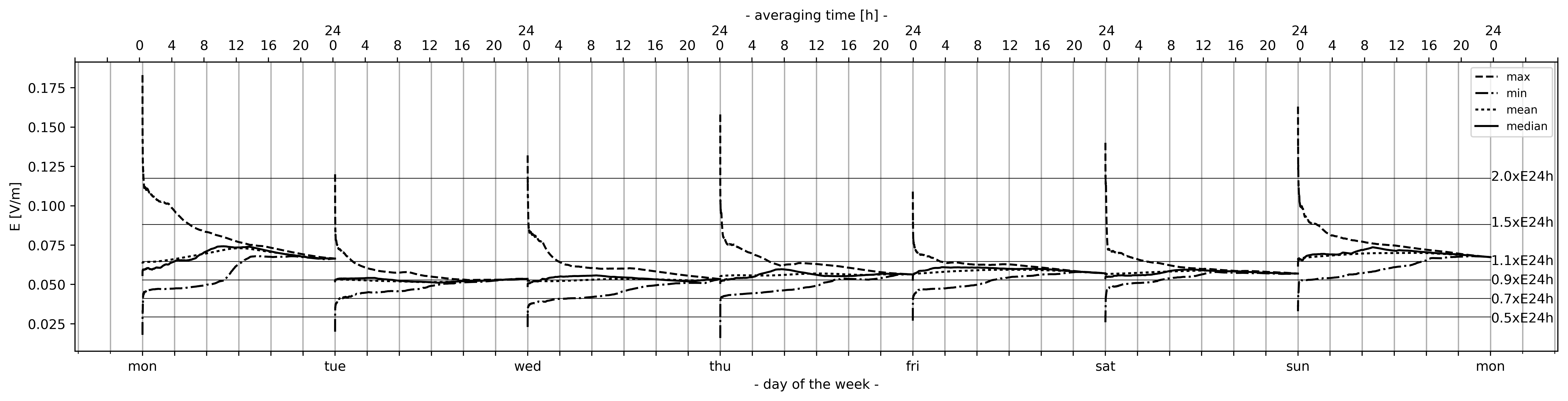

Supplement: Supplementary_material_for_Radiation_Protection_Dosimetry_Manuscript_2019_ncz154 [file supplementary_material_for_radiation_protection_dosimetry_manuscript_2019_ncz154.zip › Supplementary material for Radiation Protection Dosimetry Manuscript 2019/Location1_Figures_2ndWeek/Figure4_UMTS_2ndWeek.jpg]

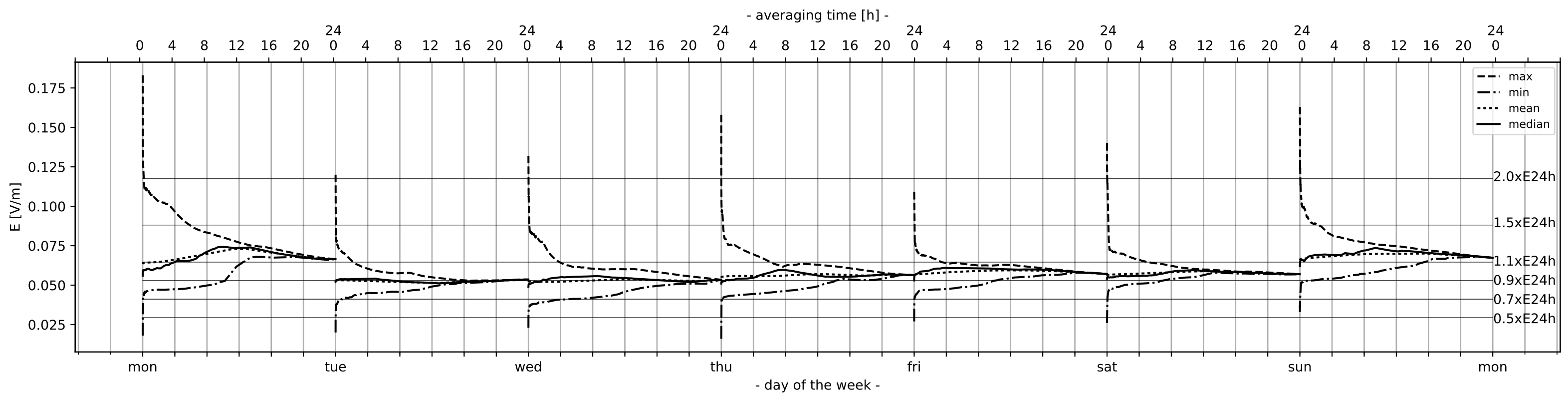

Supplement: Supplementary_material_for_Radiation_Protection_Dosimetry_Manuscript_2019_ncz154 [file supplementary_material_for_radiation_protection_dosimetry_manuscript_2019_ncz154.zip › Supplementary material for Radiation Protection Dosimetry Manuscript 2019/Location1_Figures_2ndWeek/Figure4_UMTS_2ndWeek.pdf]

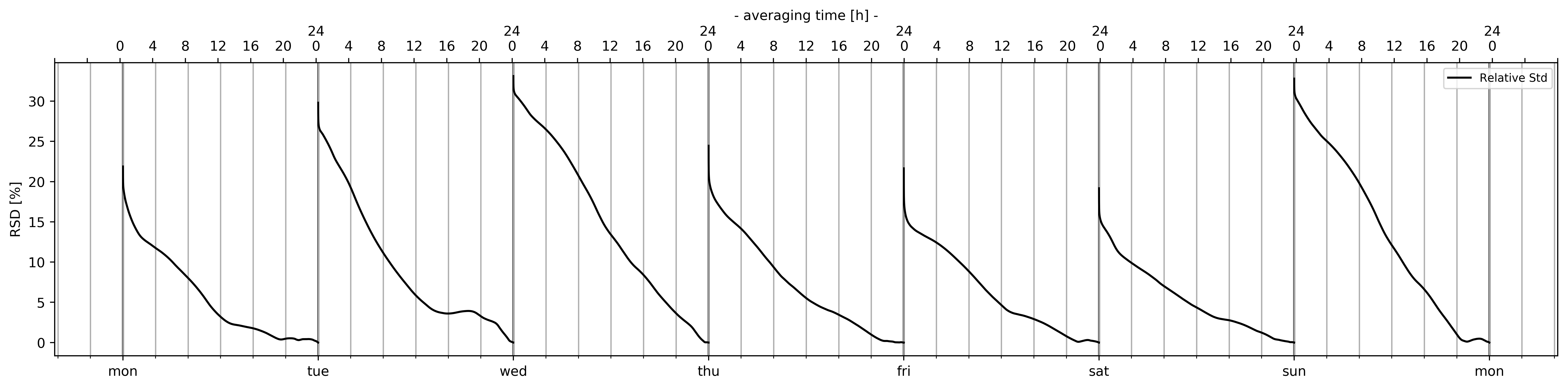

Supplement: Supplementary_material_for_Radiation_Protection_Dosimetry_Manuscript_2019_ncz154 [file supplementary_material_for_radiation_protection_dosimetry_manuscript_2019_ncz154.zip › Supplementary material for Radiation Protection Dosimetry Manuscript 2019/Location1_Figures_2ndWeek/Figure5_DCS_2ndWeek.jpg]

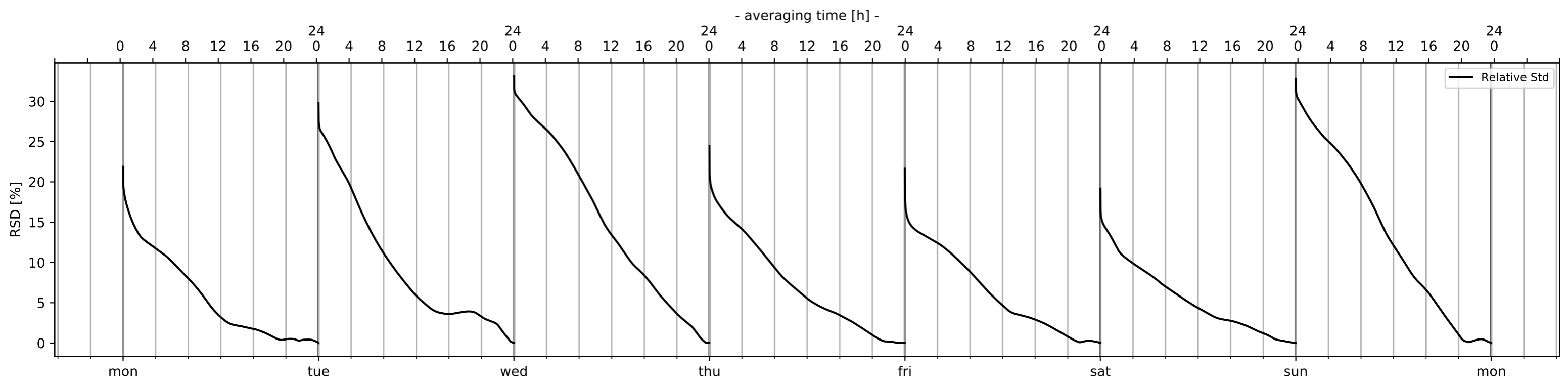

Supplement: Supplementary_material_for_Radiation_Protection_Dosimetry_Manuscript_2019_ncz154 [file supplementary_material_for_radiation_protection_dosimetry_manuscript_2019_ncz154.zip › Supplementary material for Radiation Protection Dosimetry Manuscript 2019/Location1_Figures_2ndWeek/Figure5_DCS_2ndWeek.pdf]

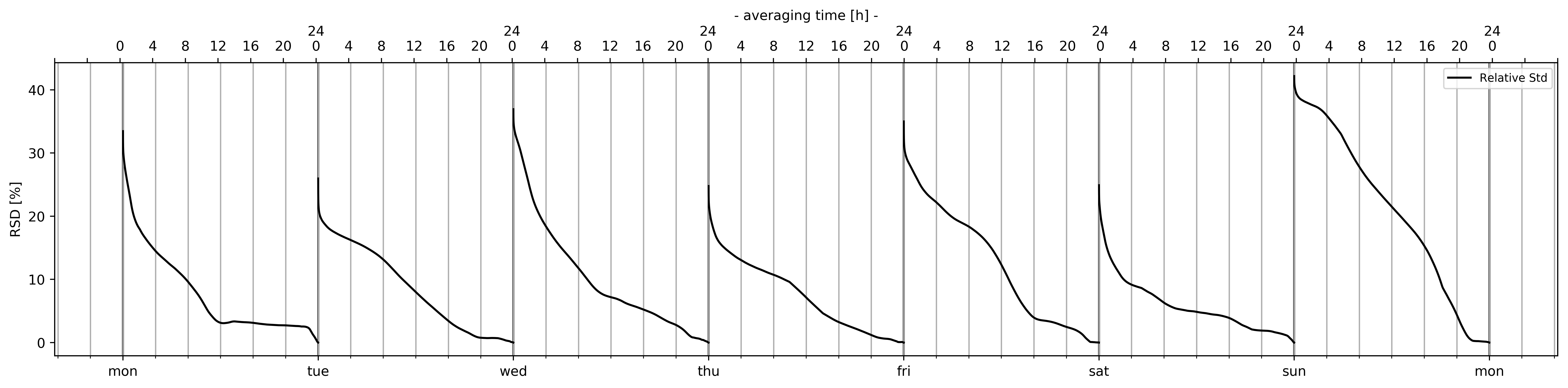

Supplement: Supplementary_material_for_Radiation_Protection_Dosimetry_Manuscript_2019_ncz154 [file supplementary_material_for_radiation_protection_dosimetry_manuscript_2019_ncz154.zip › Supplementary material for Radiation Protection Dosimetry Manuscript 2019/Location1_Figures_2ndWeek/Figure5_GSM_2ndWeek.jpg]

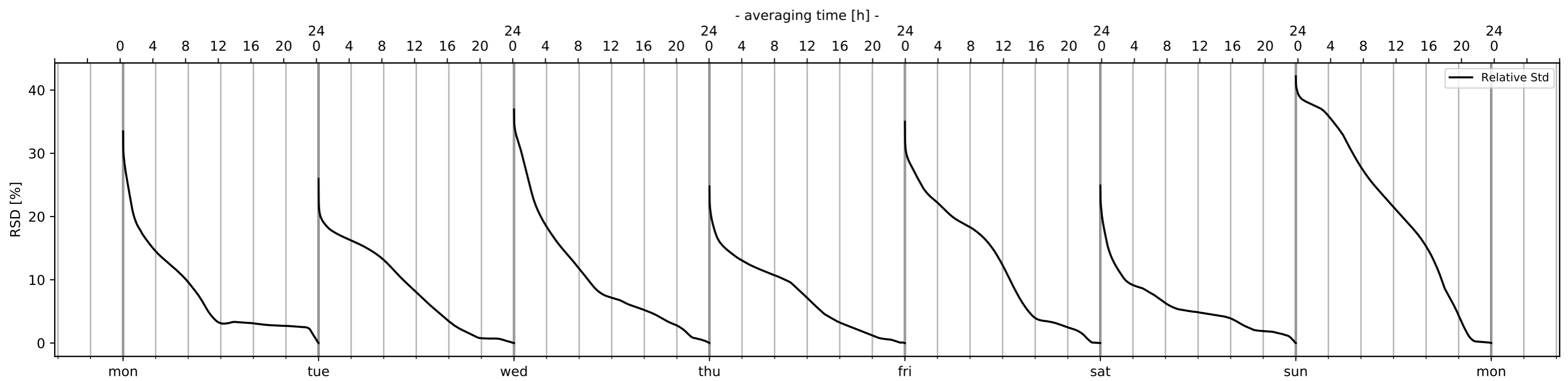

Supplement: Supplementary_material_for_Radiation_Protection_Dosimetry_Manuscript_2019_ncz154 [file supplementary_material_for_radiation_protection_dosimetry_manuscript_2019_ncz154.zip › Supplementary material for Radiation Protection Dosimetry Manuscript 2019/Location1_Figures_2ndWeek/Figure5_GSM_2ndWeek.pdf]

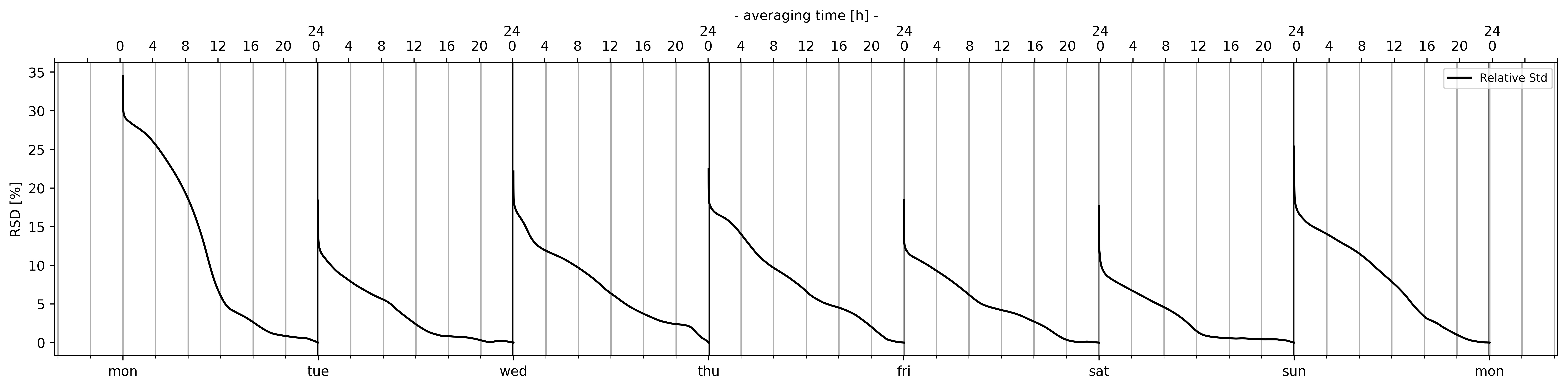

Supplement: Supplementary_material_for_Radiation_Protection_Dosimetry_Manuscript_2019_ncz154 [file supplementary_material_for_radiation_protection_dosimetry_manuscript_2019_ncz154.zip › Supplementary material for Radiation Protection Dosimetry Manuscript 2019/Location1_Figures_2ndWeek/Figure5_UMTS_2ndWeek.jpg]

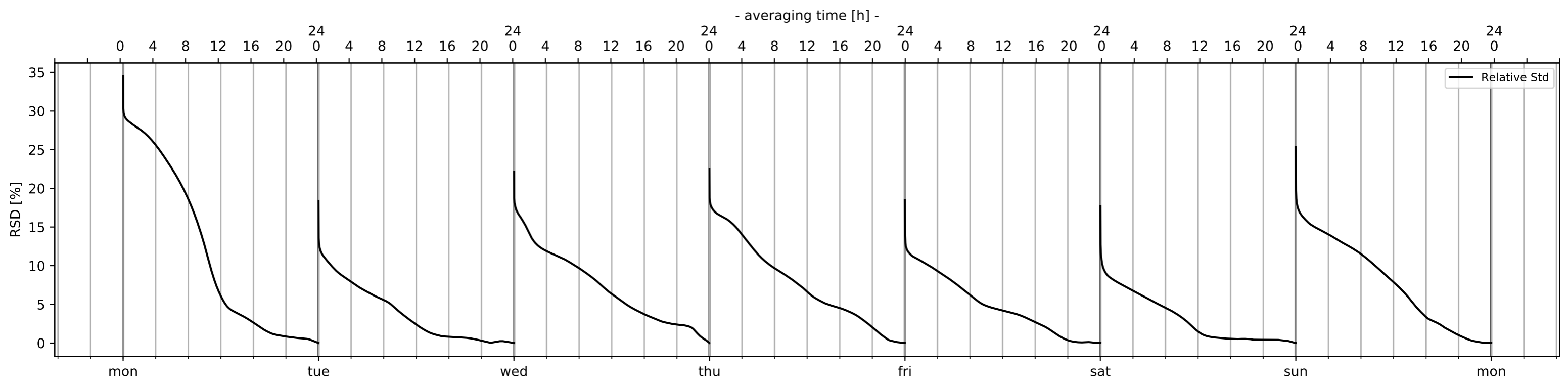

Supplement: Supplementary_material_for_Radiation_Protection_Dosimetry_Manuscript_2019_ncz154 [file supplementary_material_for_radiation_protection_dosimetry_manuscript_2019_ncz154.zip › Supplementary material for Radiation Protection Dosimetry Manuscript 2019/Location1_Figures_2ndWeek/Figure5_UMTS_2ndWeek.pdf]

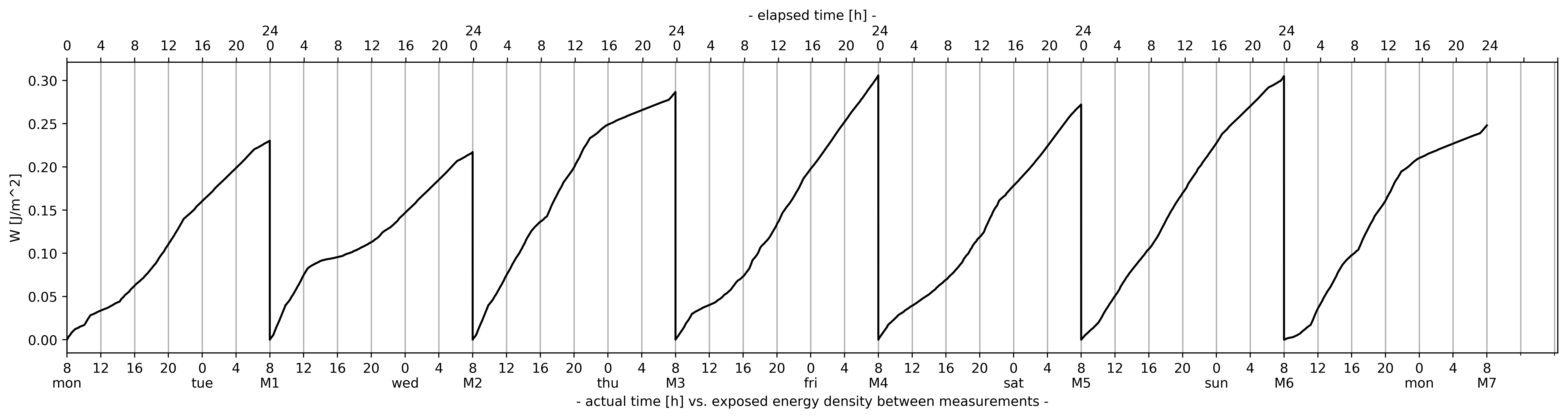

Supplement: Supplementary_material_for_Radiation_Protection_Dosimetry_Manuscript_2019_ncz154 [file supplementary_material_for_radiation_protection_dosimetry_manuscript_2019_ncz154.zip › Supplementary material for Radiation Protection Dosimetry Manuscript 2019/Location1_Figures_2ndWeek/Figure6_DCS_2ndWeek.jpg]

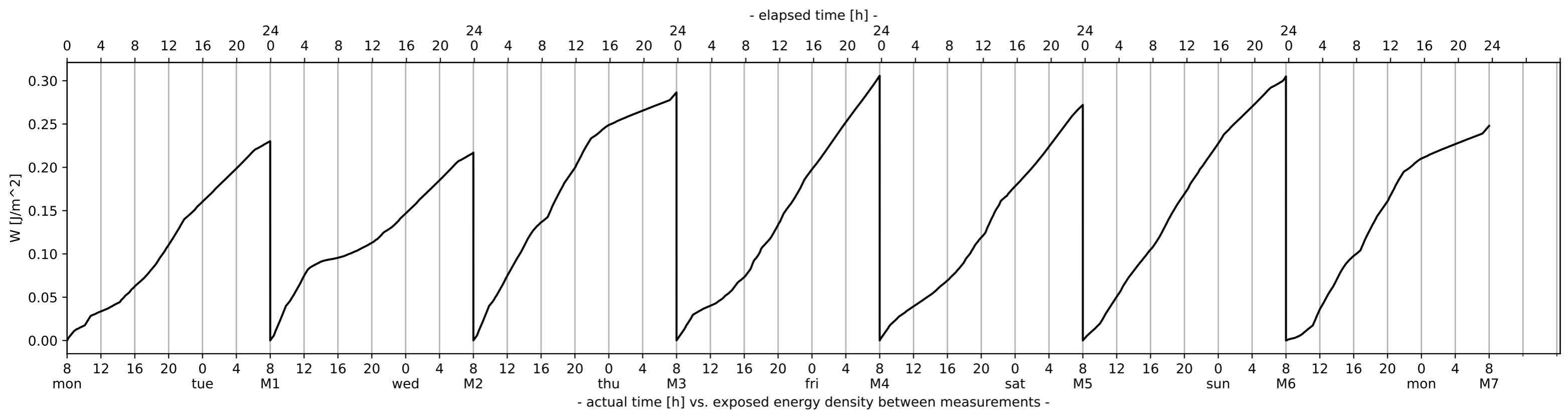

Supplement: Supplementary_material_for_Radiation_Protection_Dosimetry_Manuscript_2019_ncz154 [file supplementary_material_for_radiation_protection_dosimetry_manuscript_2019_ncz154.zip › Supplementary material for Radiation Protection Dosimetry Manuscript 2019/Location1_Figures_2ndWeek/Figure6_DCS_2ndWeek.pdf]

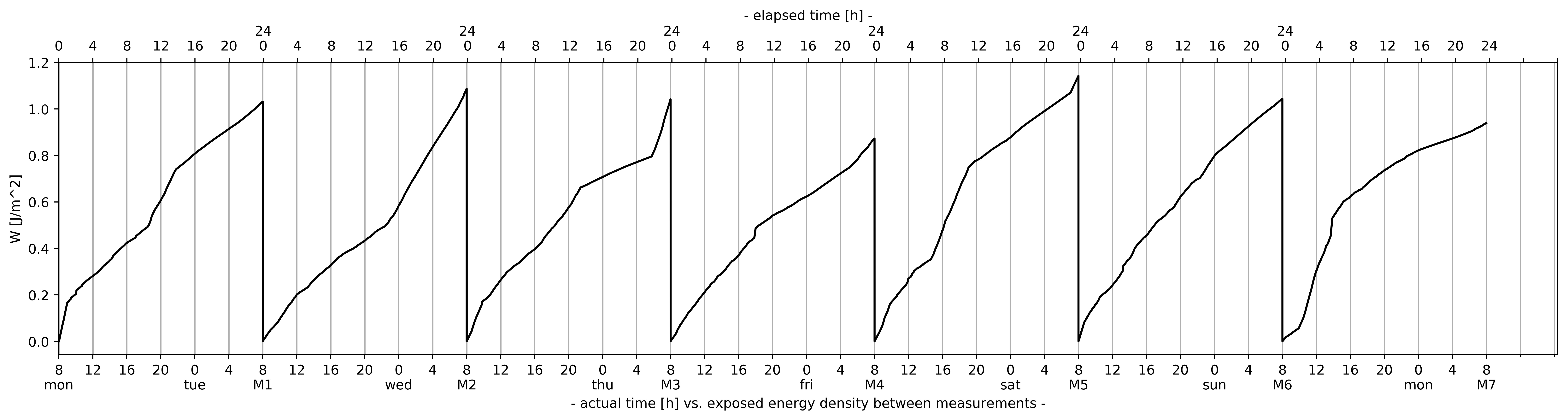

Supplement: Supplementary_material_for_Radiation_Protection_Dosimetry_Manuscript_2019_ncz154 [file supplementary_material_for_radiation_protection_dosimetry_manuscript_2019_ncz154.zip › Supplementary material for Radiation Protection Dosimetry Manuscript 2019/Location1_Figures_2ndWeek/Figure6_GSM_2ndWeek.jpg]

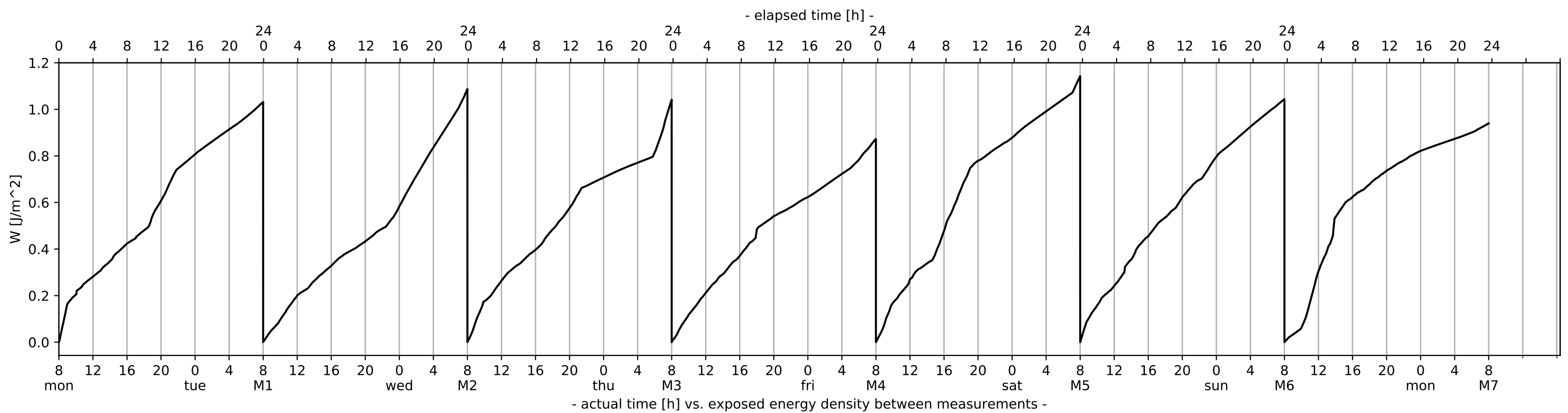

Supplement: Supplementary_material_for_Radiation_Protection_Dosimetry_Manuscript_2019_ncz154 [file supplementary_material_for_radiation_protection_dosimetry_manuscript_2019_ncz154.zip › Supplementary material for Radiation Protection Dosimetry Manuscript 2019/Location1_Figures_2ndWeek/Figure6_GSM_2ndWeek.pdf]

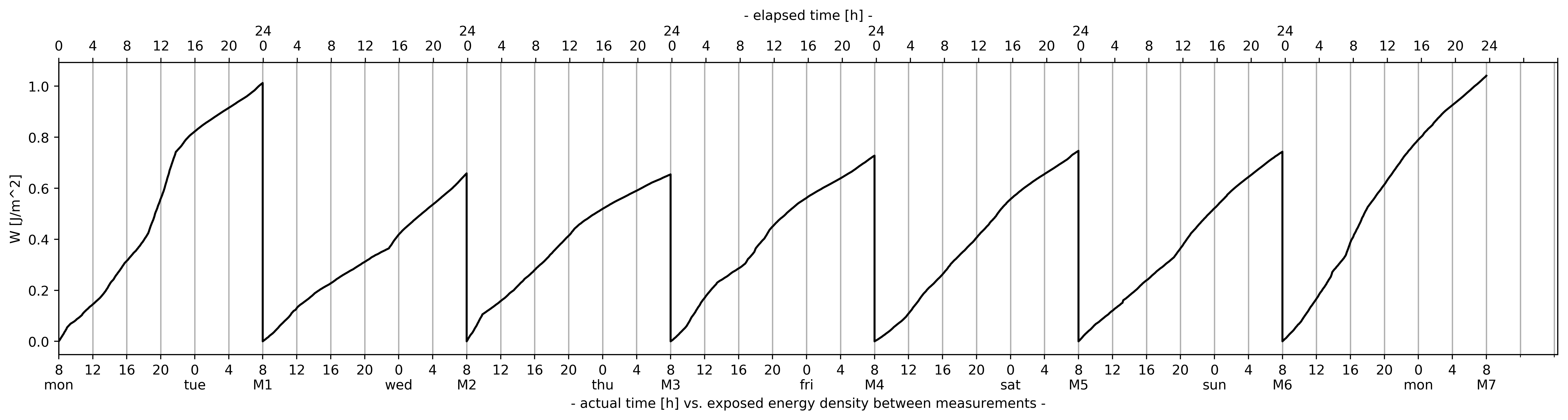

Supplement: Supplementary_material_for_Radiation_Protection_Dosimetry_Manuscript_2019_ncz154 [file supplementary_material_for_radiation_protection_dosimetry_manuscript_2019_ncz154.zip › Supplementary material for Radiation Protection Dosimetry Manuscript 2019/Location1_Figures_2ndWeek/Figure6_UMTS_2ndWeek.jpg]

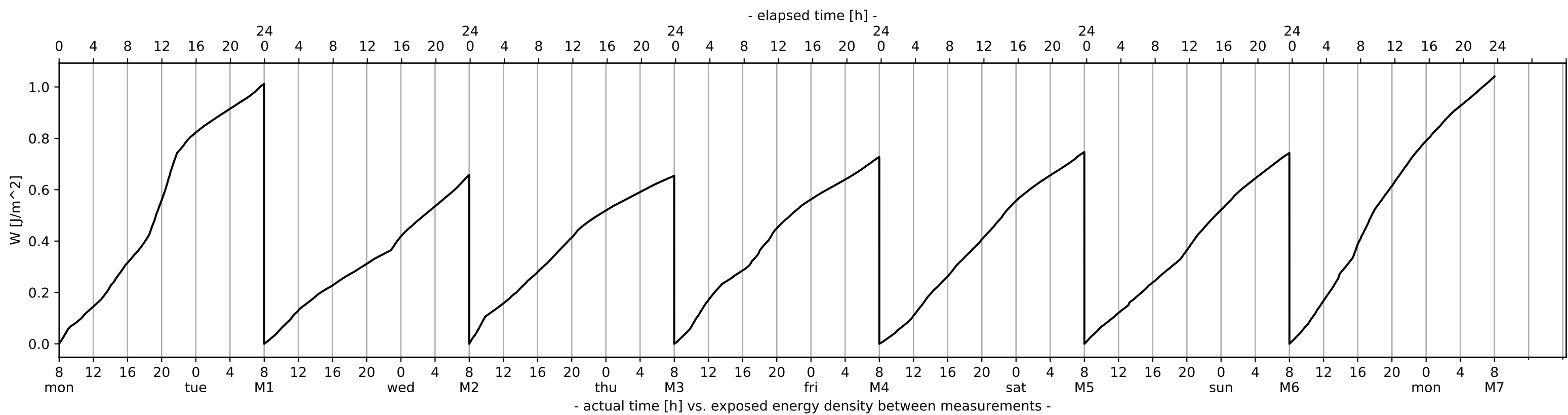

Supplement: Supplementary_material_for_Radiation_Protection_Dosimetry_Manuscript_2019_ncz154 [file supplementary_material_for_radiation_protection_dosimetry_manuscript_2019_ncz154.zip › Supplementary material for Radiation Protection Dosimetry Manuscript 2019/Location1_Figures_2ndWeek/Figure6_UMTS_2ndWeek.pdf]

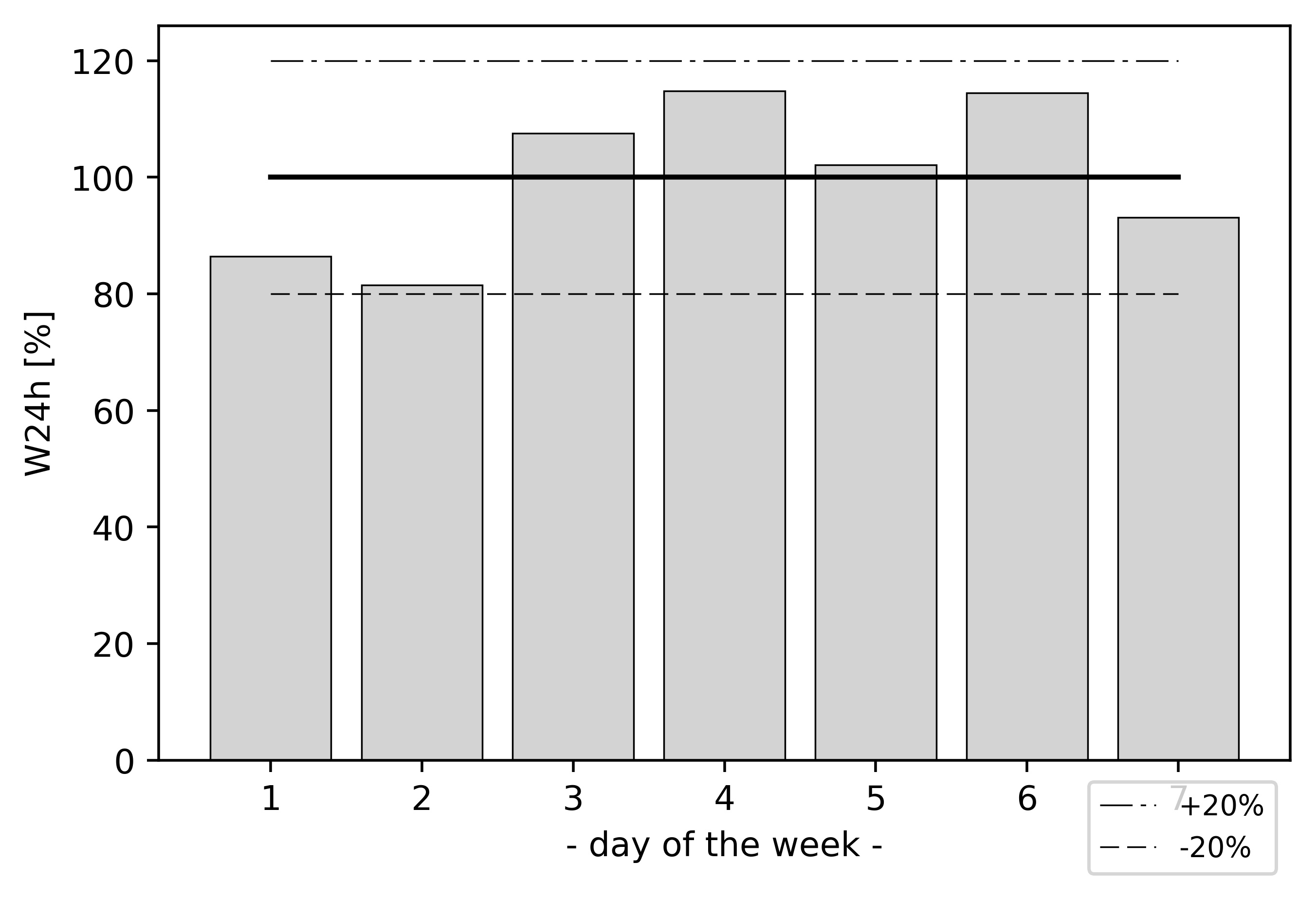

Supplement: Supplementary_material_for_Radiation_Protection_Dosimetry_Manuscript_2019_ncz154 [file supplementary_material_for_radiation_protection_dosimetry_manuscript_2019_ncz154.zip › Supplementary material for Radiation Protection Dosimetry Manuscript 2019/Location1_Figures_2ndWeek/Figure7_DCS_2ndWeek.jpg]

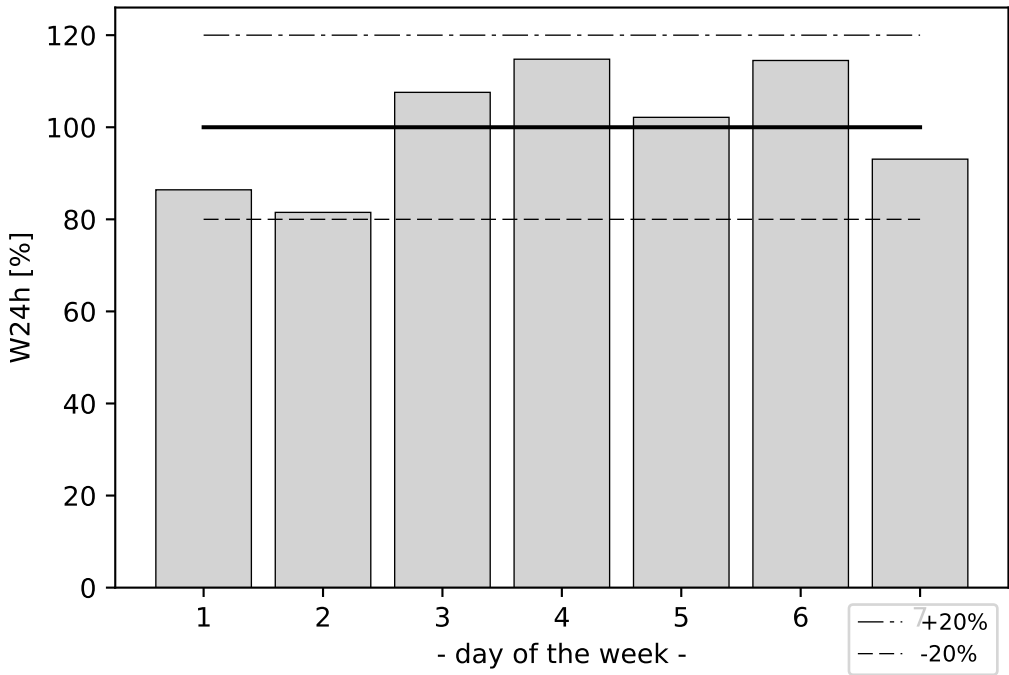

Supplement: Supplementary_material_for_Radiation_Protection_Dosimetry_Manuscript_2019_ncz154 [file supplementary_material_for_radiation_protection_dosimetry_manuscript_2019_ncz154.zip › Supplementary material for Radiation Protection Dosimetry Manuscript 2019/Location1_Figures_2ndWeek/Figure7_DCS_2ndWeek.pdf]

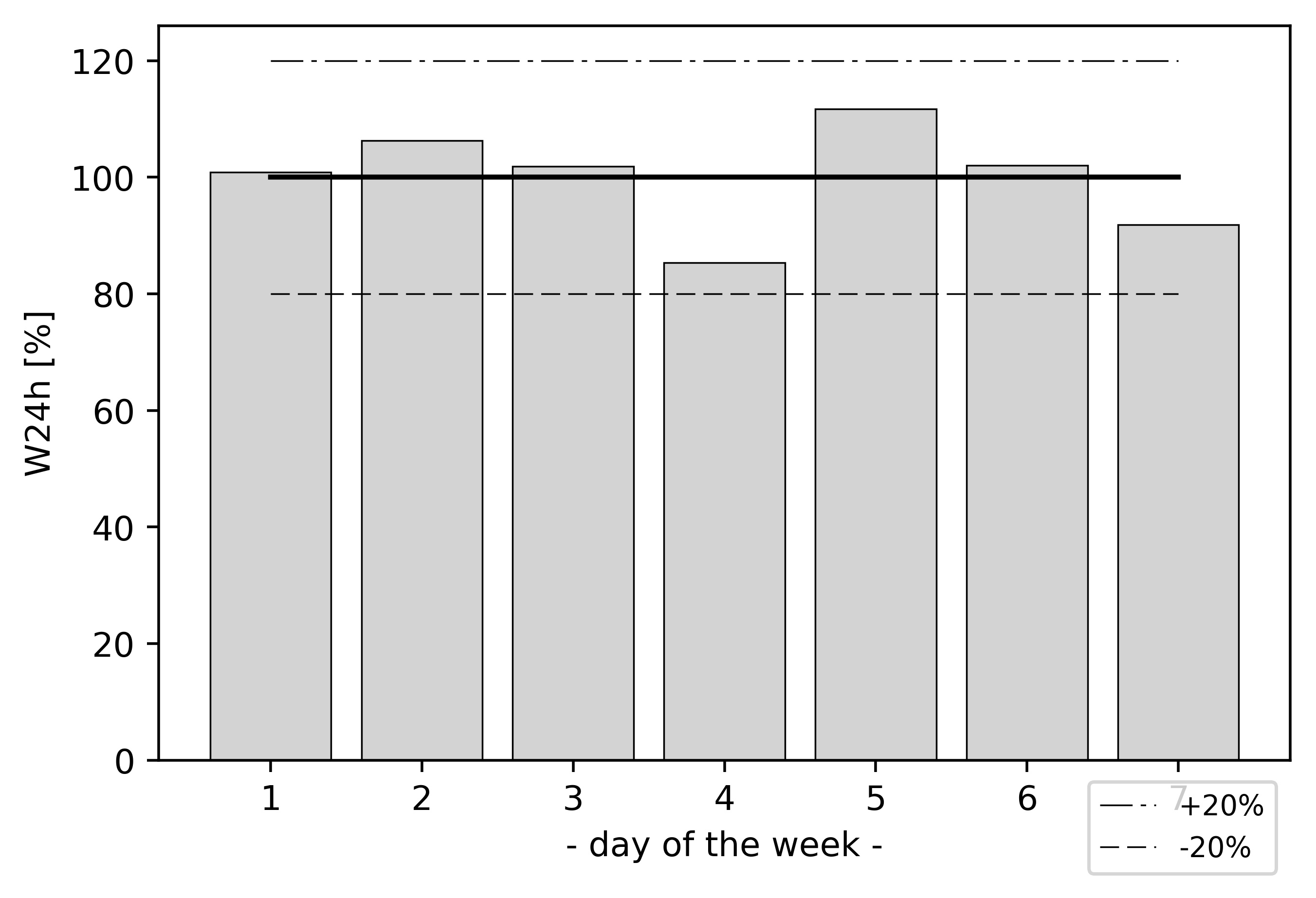

Supplement: Supplementary_material_for_Radiation_Protection_Dosimetry_Manuscript_2019_ncz154 [file supplementary_material_for_radiation_protection_dosimetry_manuscript_2019_ncz154.zip › Supplementary material for Radiation Protection Dosimetry Manuscript 2019/Location1_Figures_2ndWeek/Figure7_GSM_2ndWeek.jpg]

W24h [%]

120

100

80

60

40

20

0

1

2

3

4

5

6

- day of the week -

— ··· +20%

— ··· -20%

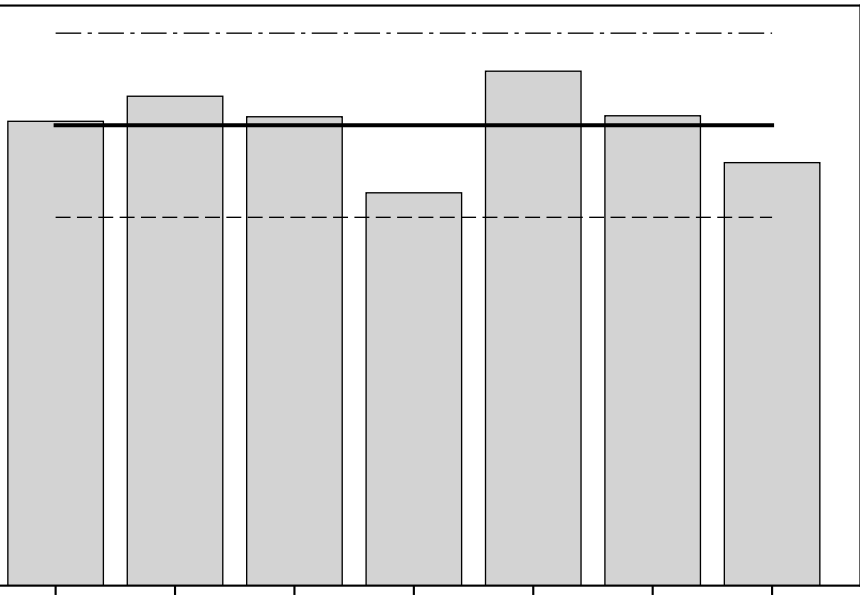

Supplement: Supplementary_material_for_Radiation_Protection_Dosimetry_Manuscript_2019_ncz154 [file supplementary_material_for_radiation_protection_dosimetry_manuscript_2019_ncz154.zip › Supplementary material for Radiation Protection Dosimetry Manuscript 2019/Location1_Figures_2ndWeek/Figure7_GSM_2ndWeek.pdf]

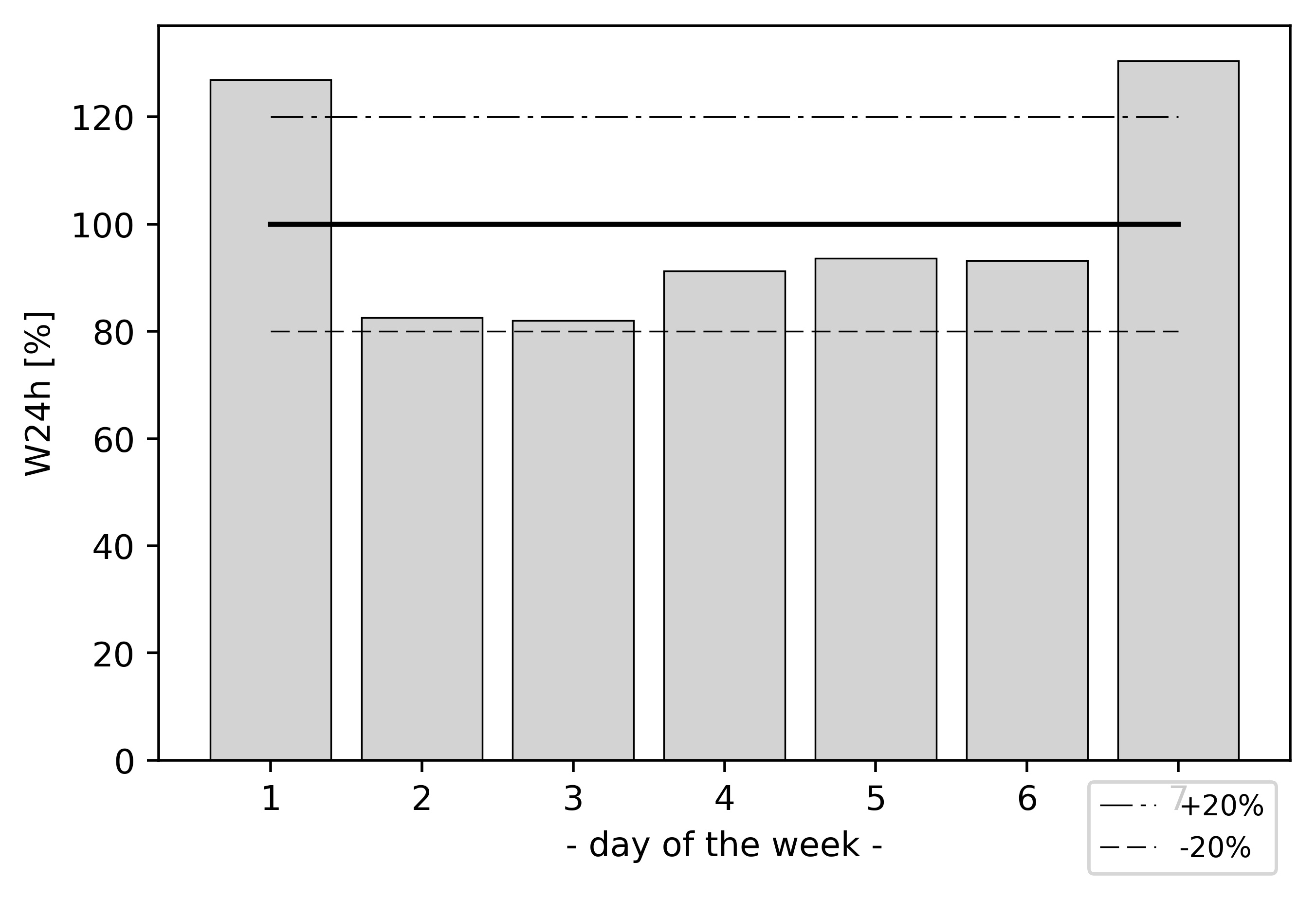

Supplement: Supplementary_material_for_Radiation_Protection_Dosimetry_Manuscript_2019_ncz154 [file supplementary_material_for_radiation_protection_dosimetry_manuscript_2019_ncz154.zip › Supplementary material for Radiation Protection Dosimetry Manuscript 2019/Location1_Figures_2ndWeek/Figure7_UMTS_2ndWeek.jpg]

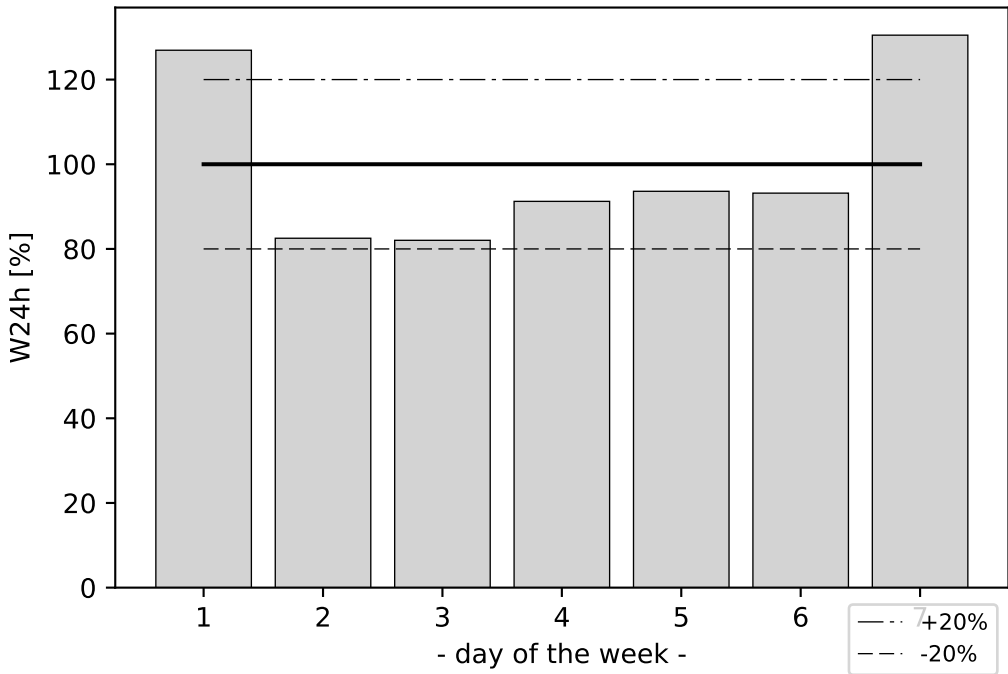

Supplement: Supplementary_material_for_Radiation_Protection_Dosimetry_Manuscript_2019_ncz154 [file supplementary_material_for_radiation_protection_dosimetry_manuscript_2019_ncz154.zip › Supplementary material for Radiation Protection Dosimetry Manuscript 2019/Location1_Figures_2ndWeek/Figure7_UMTS_2ndWeek.pdf]

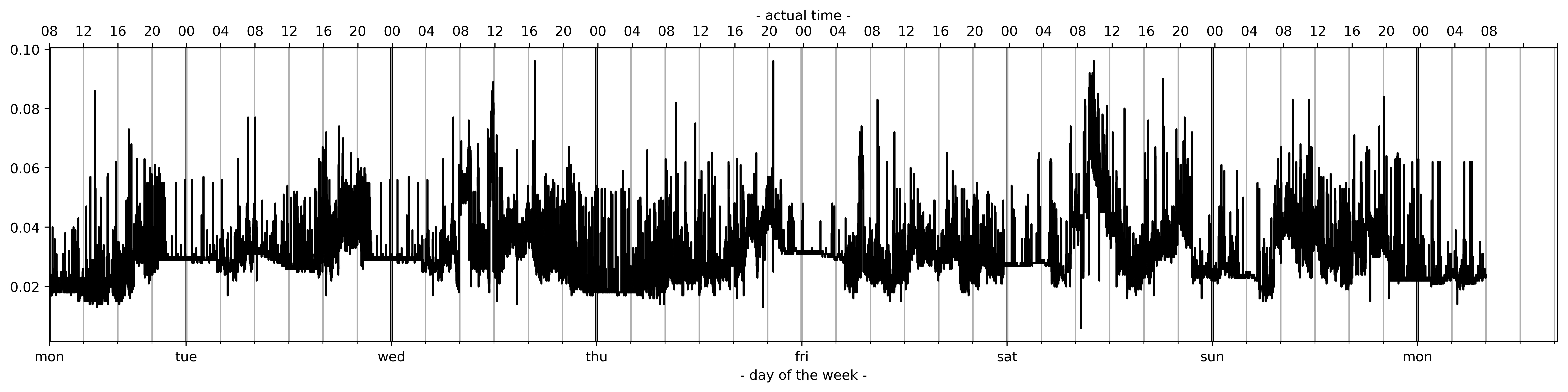

Supplement: Supplementary_material_for_Radiation_Protection_Dosimetry_Manuscript_2019_ncz154 [file supplementary_material_for_radiation_protection_dosimetry_manuscript_2019_ncz154.zip › Supplementary material for Radiation Protection Dosimetry Manuscript 2019/Location1_Figures_3rdWeek/Figure1_DCS_3rdWeek.jpg]

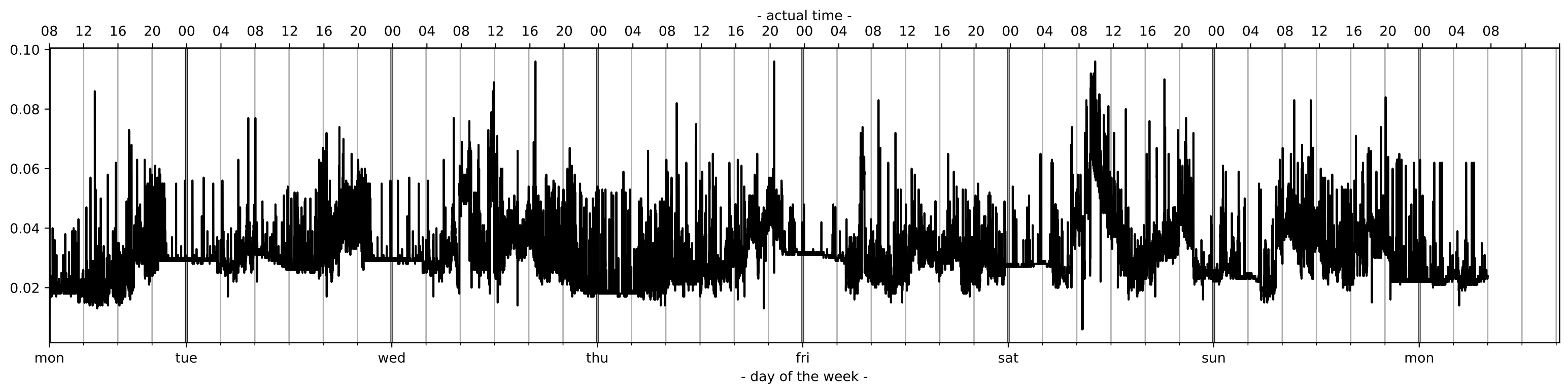

Supplement: Supplementary_material_for_Radiation_Protection_Dosimetry_Manuscript_2019_ncz154 [file supplementary_material_for_radiation_protection_dosimetry_manuscript_2019_ncz154.zip › Supplementary material for Radiation Protection Dosimetry Manuscript 2019/Location1_Figures_3rdWeek/Figure1_DCS_3rdWeek.pdf]

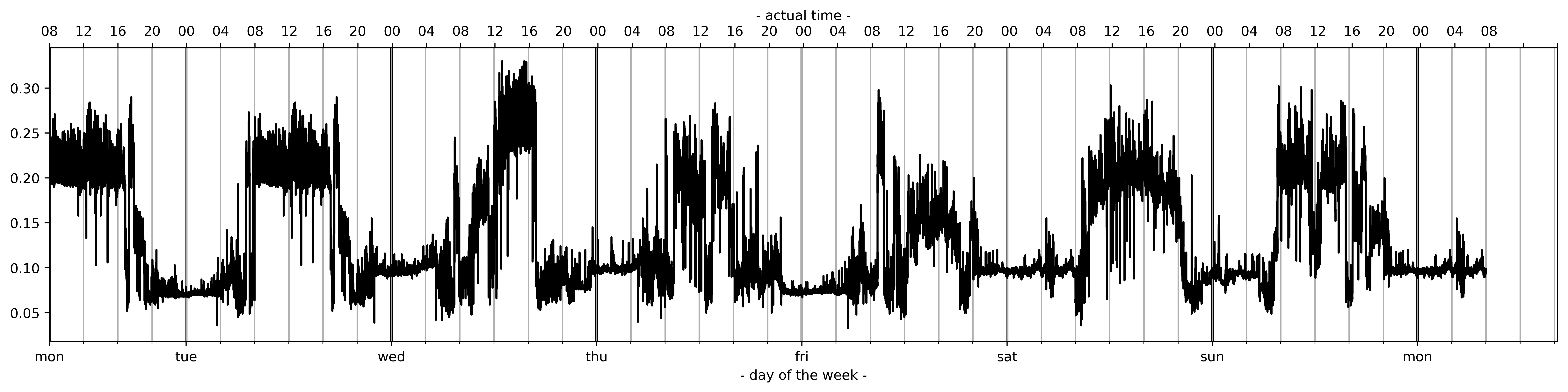

Supplement: Supplementary_material_for_Radiation_Protection_Dosimetry_Manuscript_2019_ncz154 [file supplementary_material_for_radiation_protection_dosimetry_manuscript_2019_ncz154.zip › Supplementary material for Radiation Protection Dosimetry Manuscript 2019/Location1_Figures_3rdWeek/Figure1_GSM_3rdWeek.jpg]

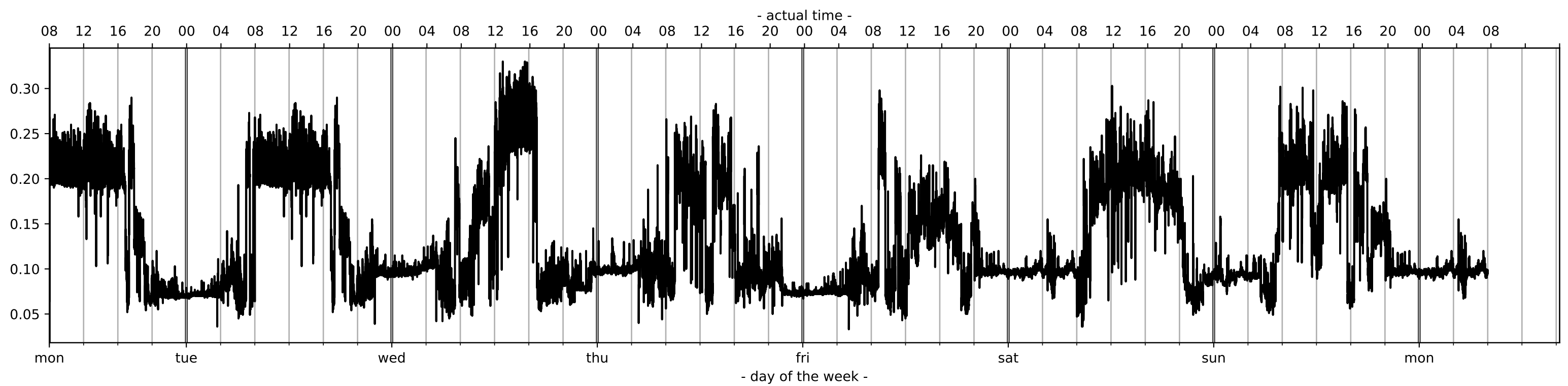

Supplement: Supplementary_material_for_Radiation_Protection_Dosimetry_Manuscript_2019_ncz154 [file supplementary_material_for_radiation_protection_dosimetry_manuscript_2019_ncz154.zip › Supplementary material for Radiation Protection Dosimetry Manuscript 2019/Location1_Figures_3rdWeek/Figure1_GSM_3rdWeek.pdf]

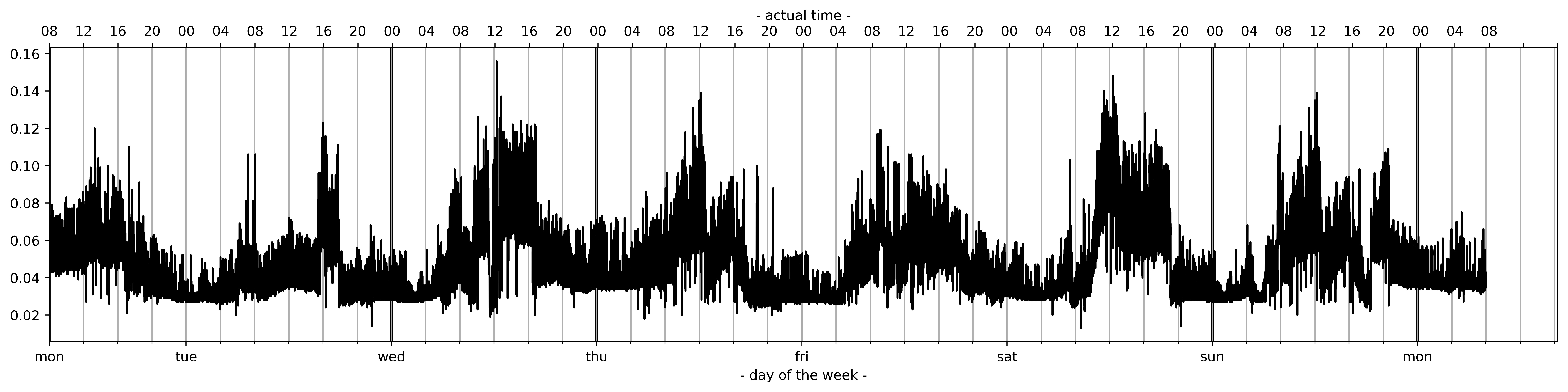

Supplement: Supplementary_material_for_Radiation_Protection_Dosimetry_Manuscript_2019_ncz154 [file supplementary_material_for_radiation_protection_dosimetry_manuscript_2019_ncz154.zip › Supplementary material for Radiation Protection Dosimetry Manuscript 2019/Location1_Figures_3rdWeek/Figure1_UMTS_3rdWeek.jpg]

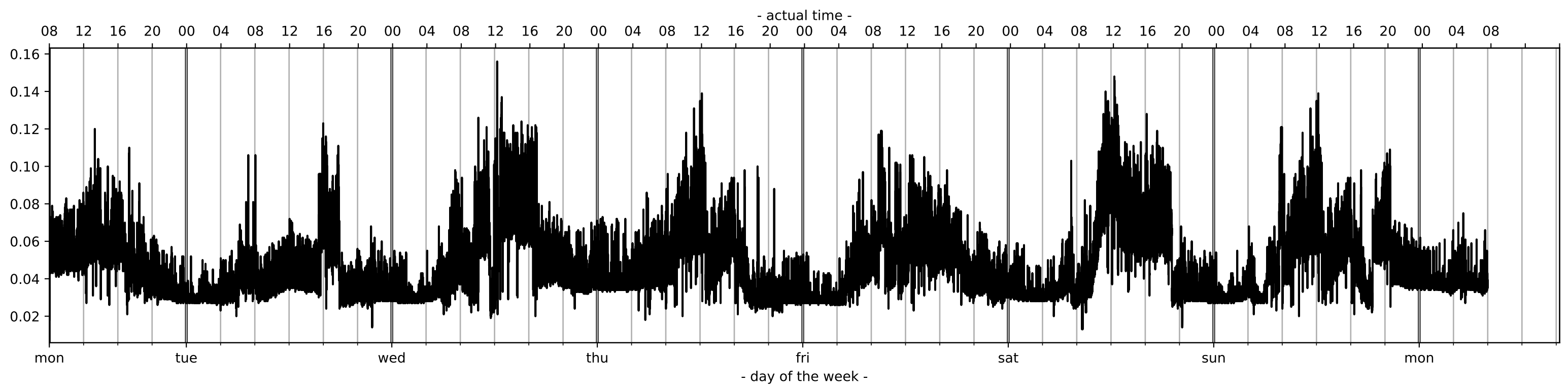

Supplement: Supplementary_material_for_Radiation_Protection_Dosimetry_Manuscript_2019_ncz154 [file supplementary_material_for_radiation_protection_dosimetry_manuscript_2019_ncz154.zip › Supplementary material for Radiation Protection Dosimetry Manuscript 2019/Location1_Figures_3rdWeek/Figure1_UMTS_3rdWeek.pdf]

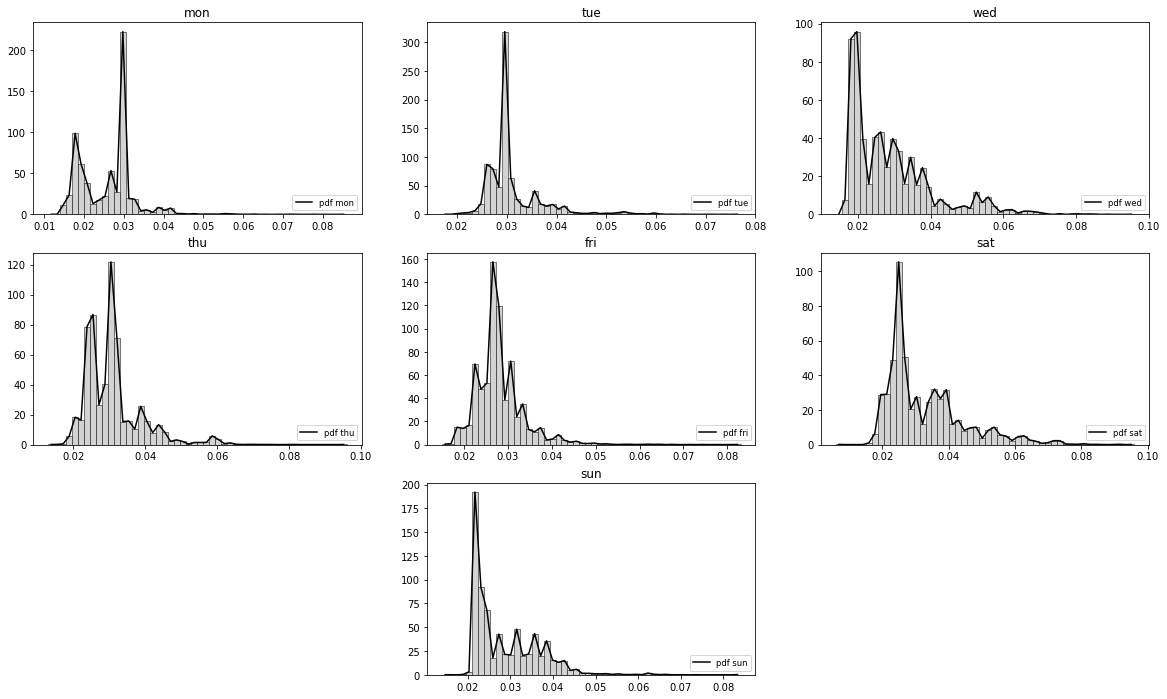

Supplement: Supplementary_material_for_Radiation_Protection_Dosimetry_Manuscript_2019_ncz154 [file supplementary_material_for_radiation_protection_dosimetry_manuscript_2019_ncz154.zip › Supplementary material for Radiation Protection Dosimetry Manuscript 2019/Location1_Figures_3rdWeek/Figure2_DCS_3rdWeek.jpg]

mon

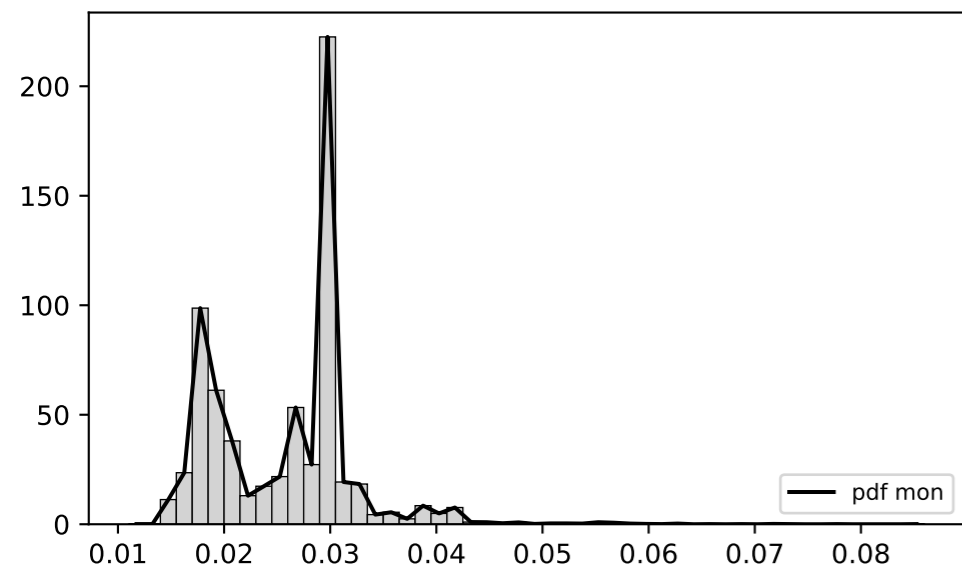

tue

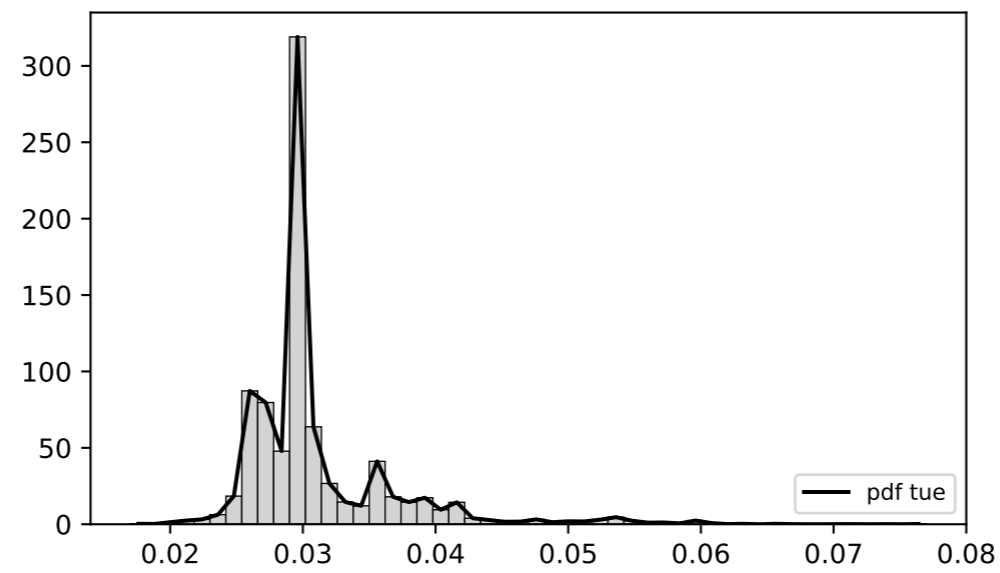

wed

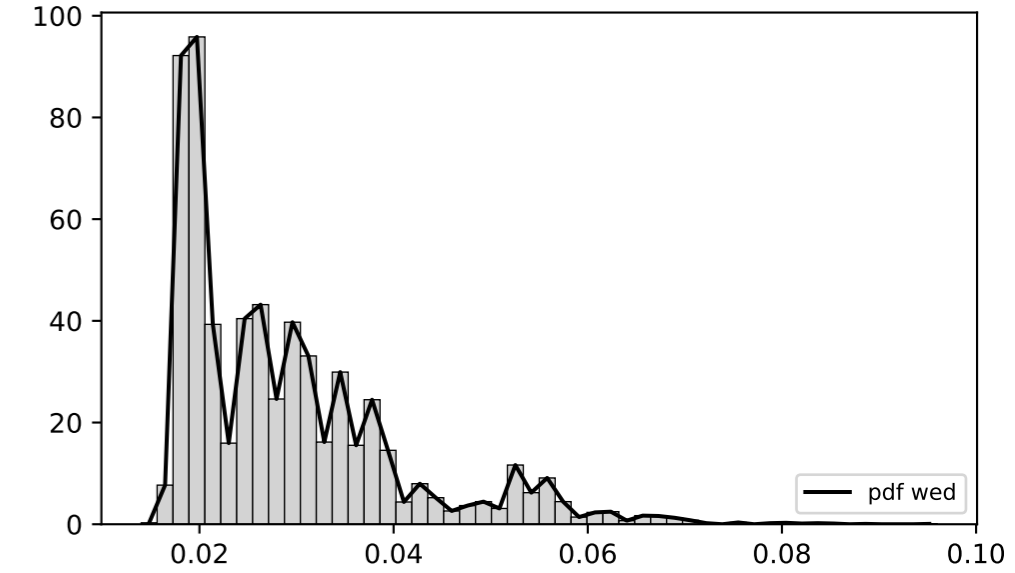

thu

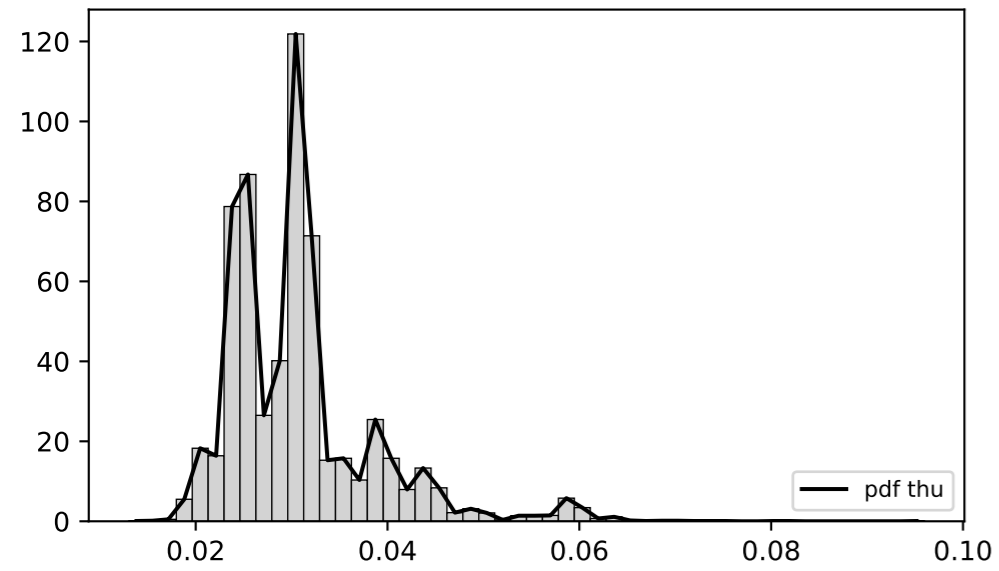

fri

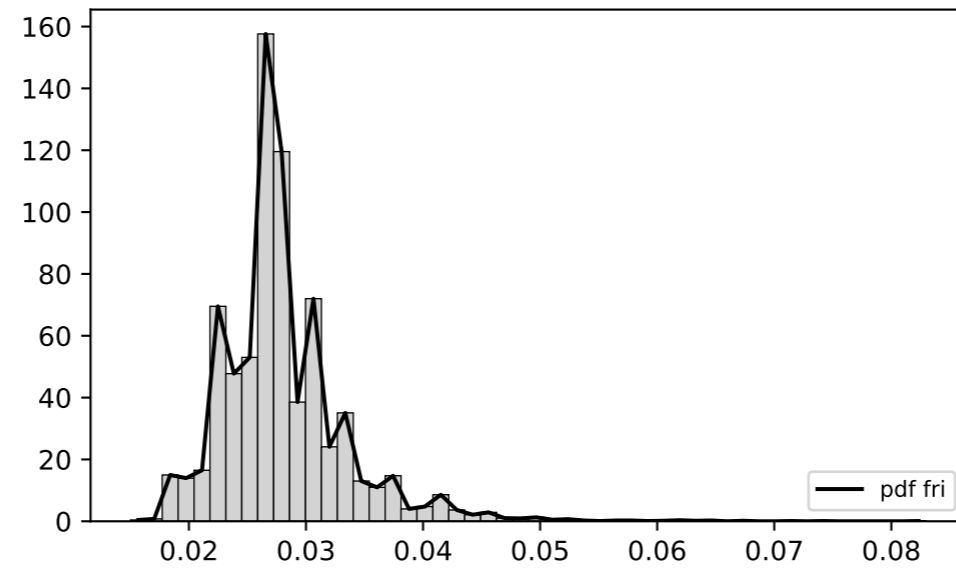

sat

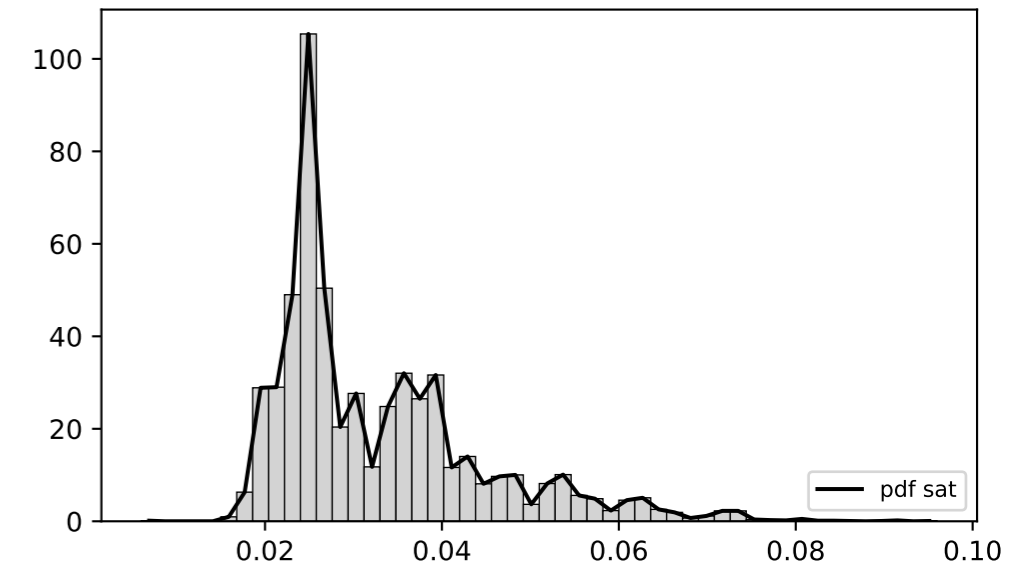

sun

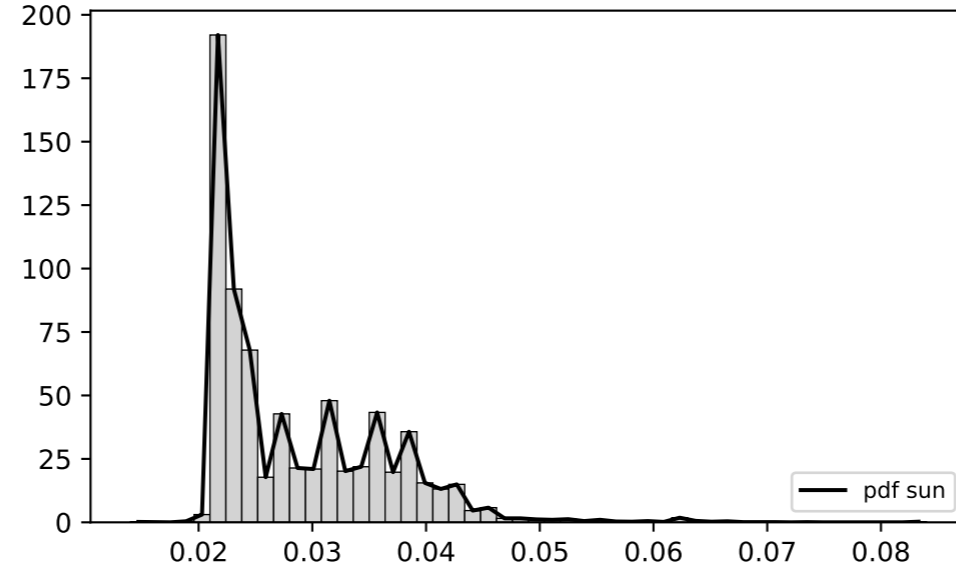

Supplement: Supplementary_material_for_Radiation_Protection_Dosimetry_Manuscript_2019_ncz154 [file supplementary_material_for_radiation_protection_dosimetry_manuscript_2019_ncz154.zip › Supplementary material for Radiation Protection Dosimetry Manuscript 2019/Location1_Figures_3rdWeek/Figure2_DCS_3rdWeek.pdf]

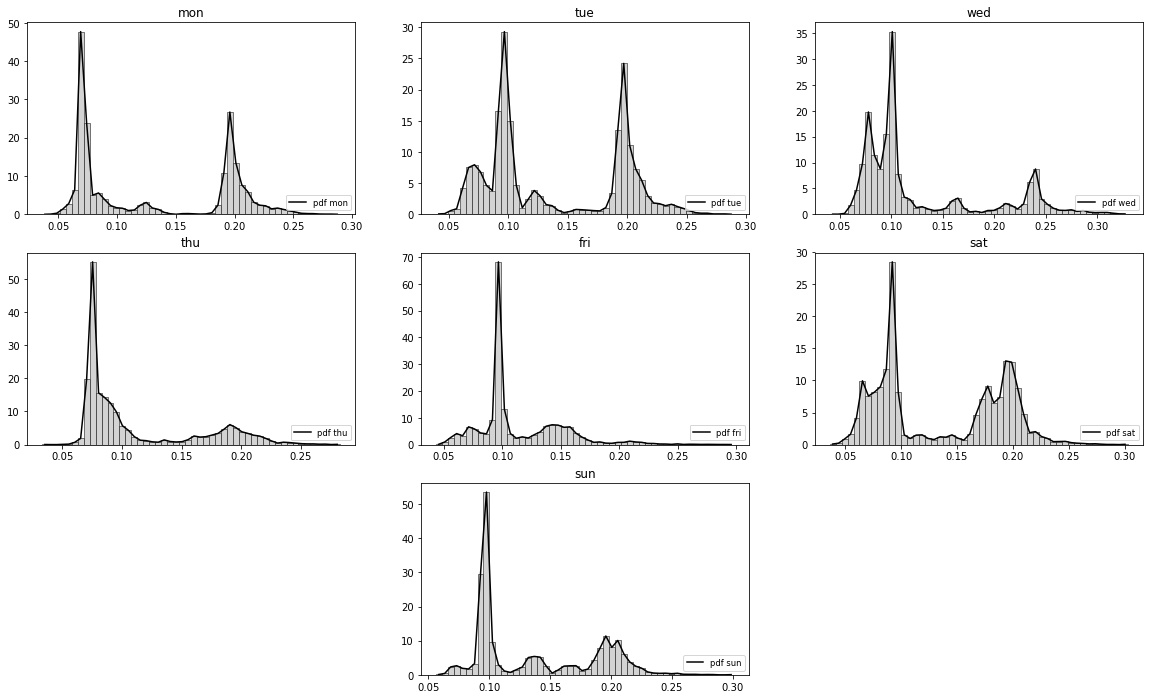

Supplement: Supplementary_material_for_Radiation_Protection_Dosimetry_Manuscript_2019_ncz154 [file supplementary_material_for_radiation_protection_dosimetry_manuscript_2019_ncz154.zip › Supplementary material for Radiation Protection Dosimetry Manuscript 2019/Location1_Figures_3rdWeek/Figure2_GSM_3rdWeek.jpg]

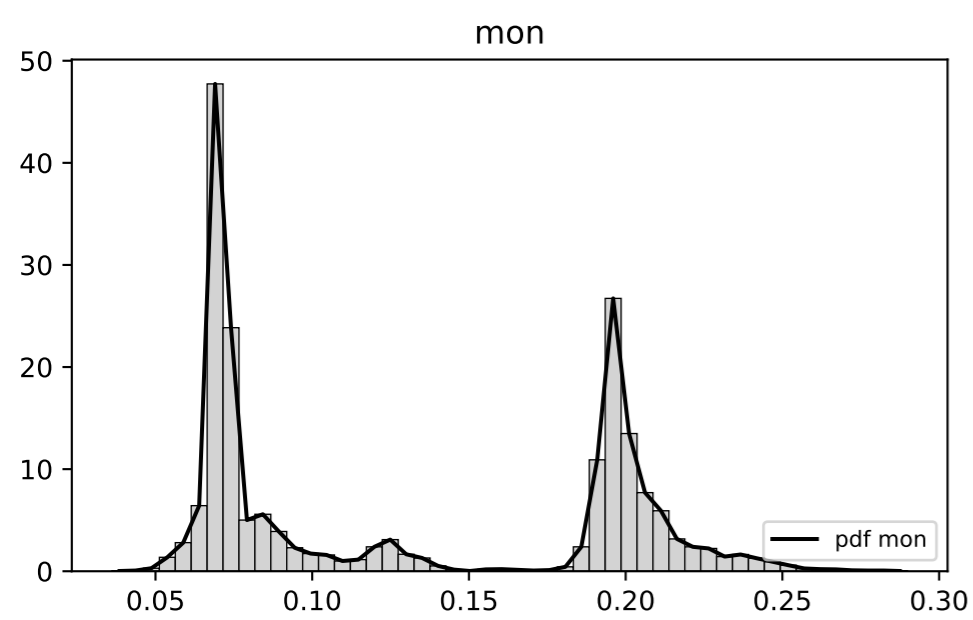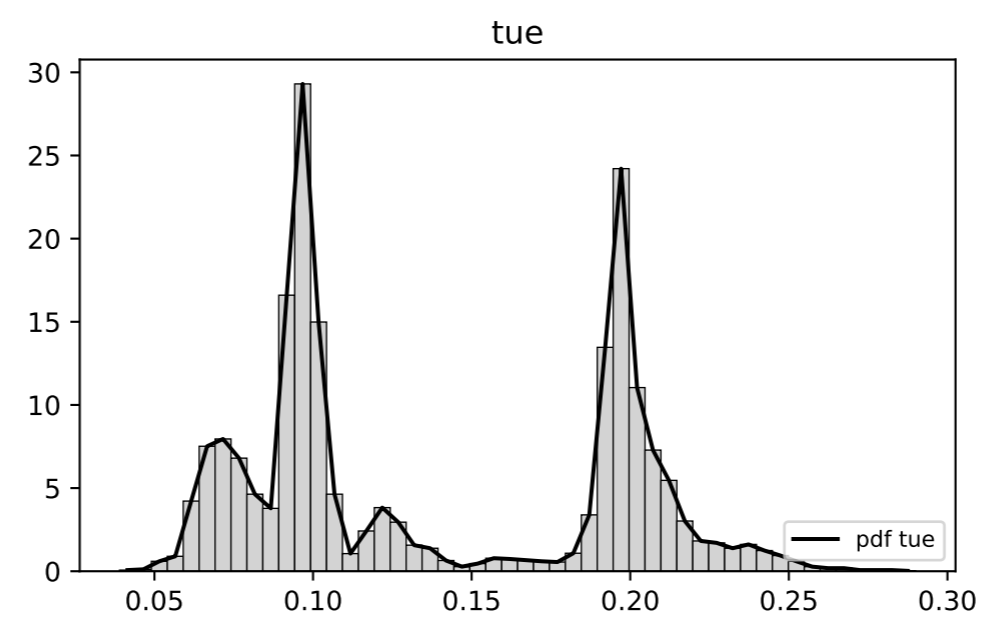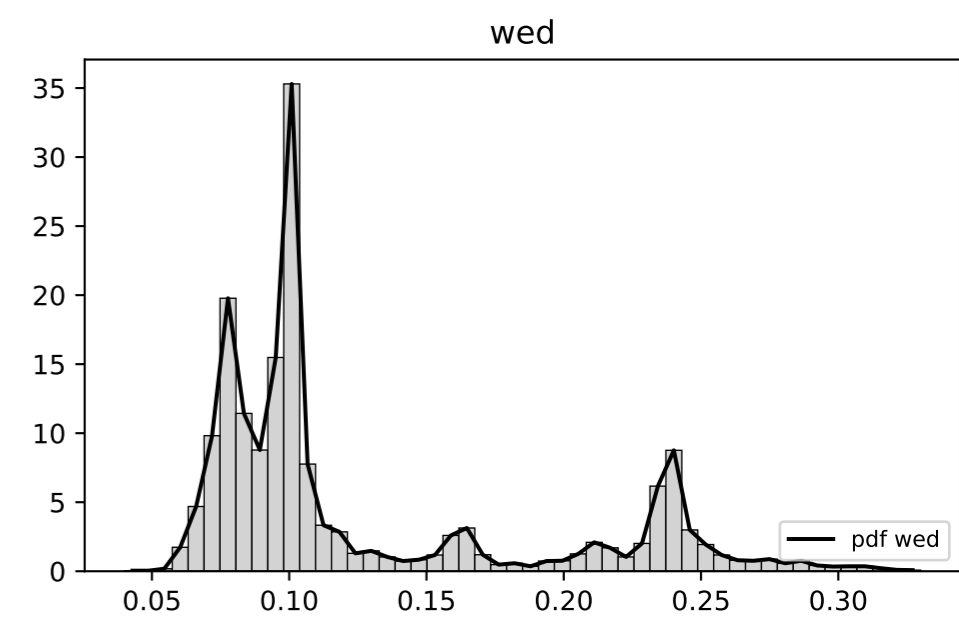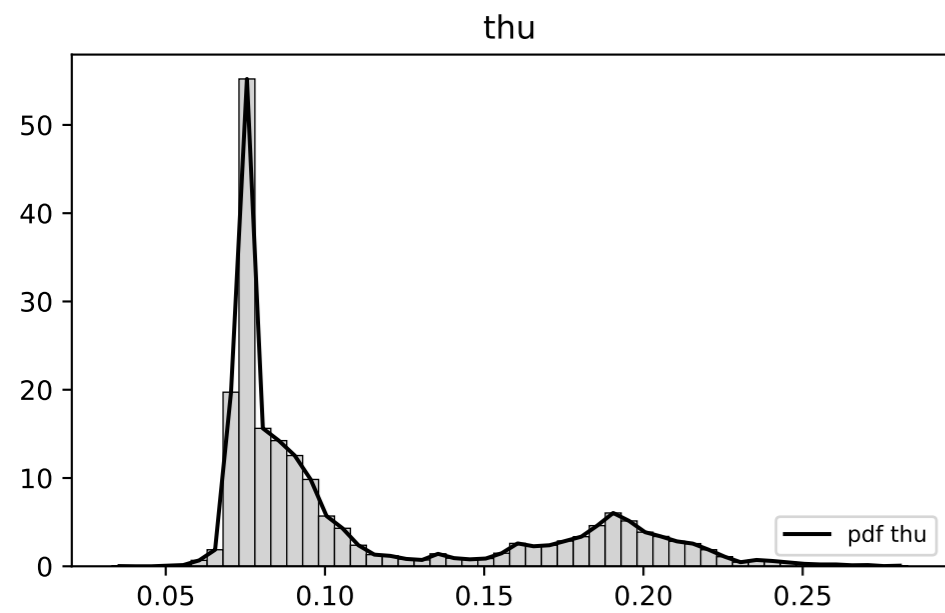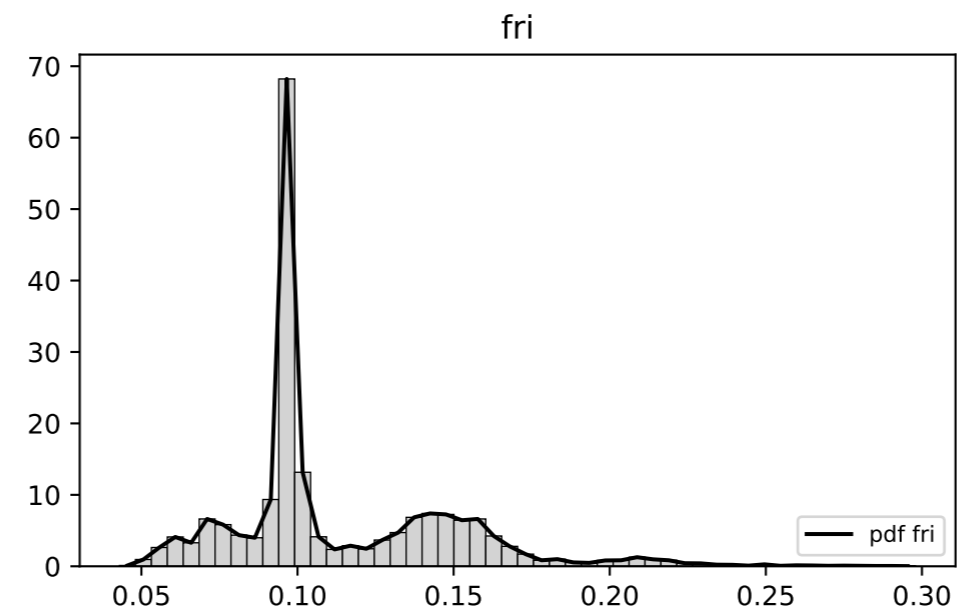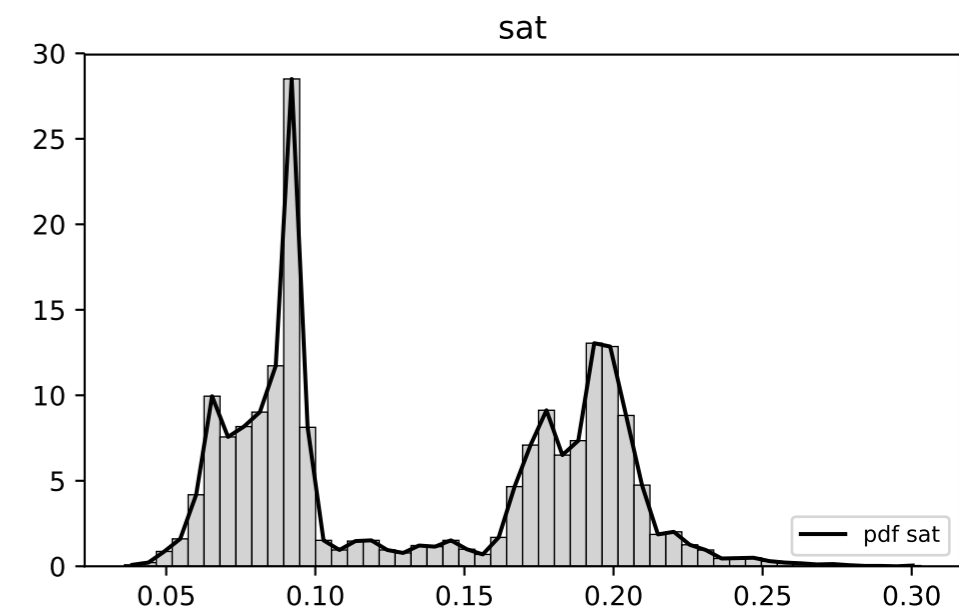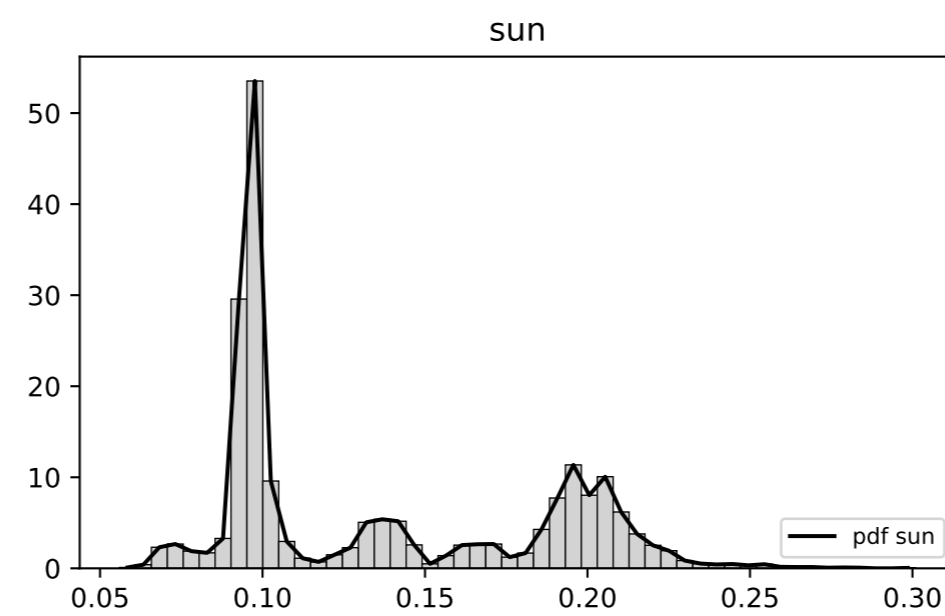

Supplement: Supplementary_material_for_Radiation_Protection_Dosimetry_Manuscript_2019_ncz154 [file supplementary_material_for_radiation_protection_dosimetry_manuscript_2019_ncz154.zip › Supplementary material for Radiation Protection Dosimetry Manuscript 2019/Location1_Figures_3rdWeek/Figure2_GSM_3rdWeek.pdf]

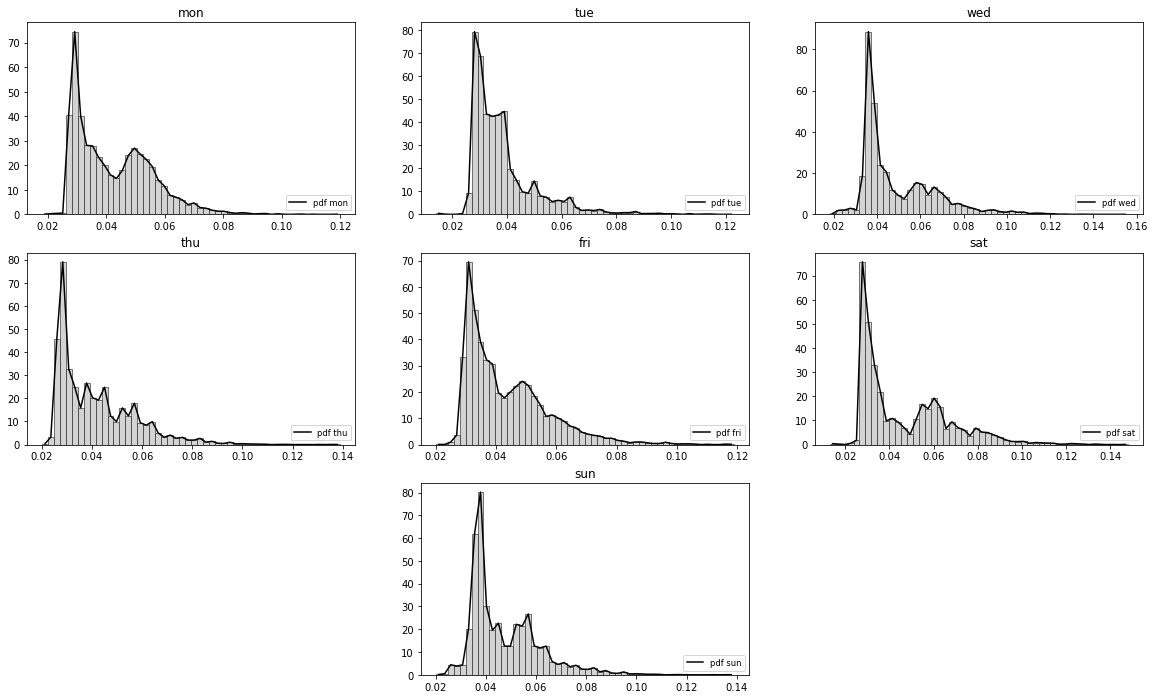

Supplement: Supplementary_material_for_Radiation_Protection_Dosimetry_Manuscript_2019_ncz154 [file supplementary_material_for_radiation_protection_dosimetry_manuscript_2019_ncz154.zip › Supplementary material for Radiation Protection Dosimetry Manuscript 2019/Location1_Figures_3rdWeek/Figure2_UMTS_3rdWeek.jpg]

mon

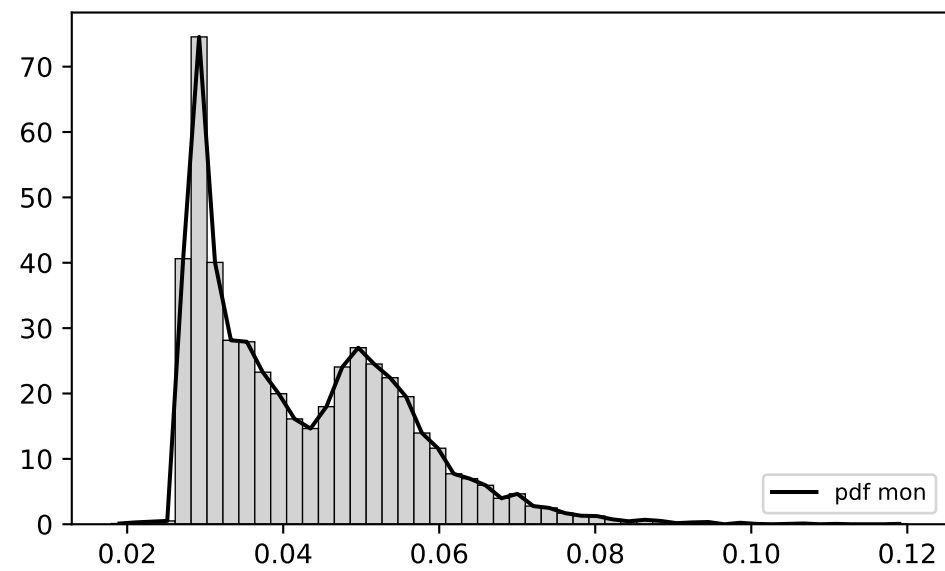

tue

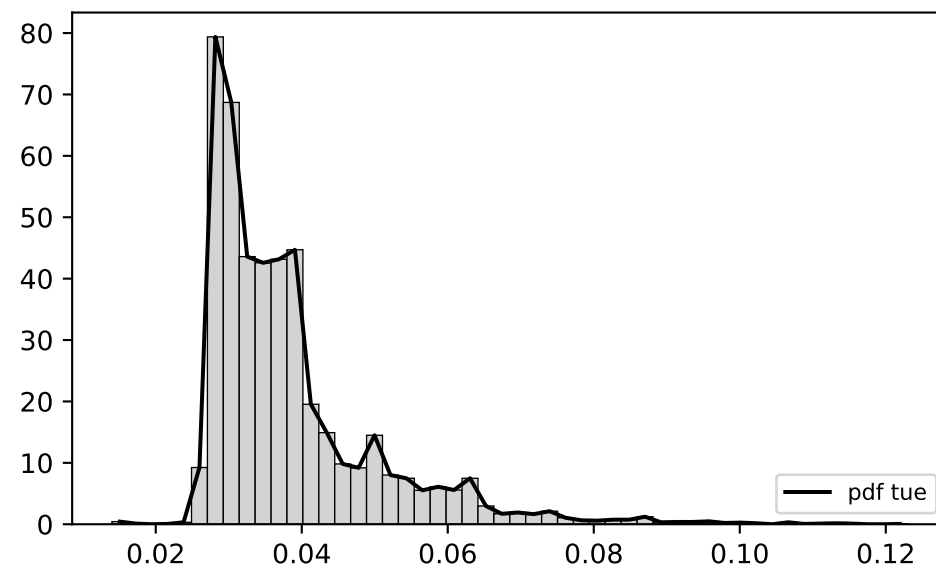

wed

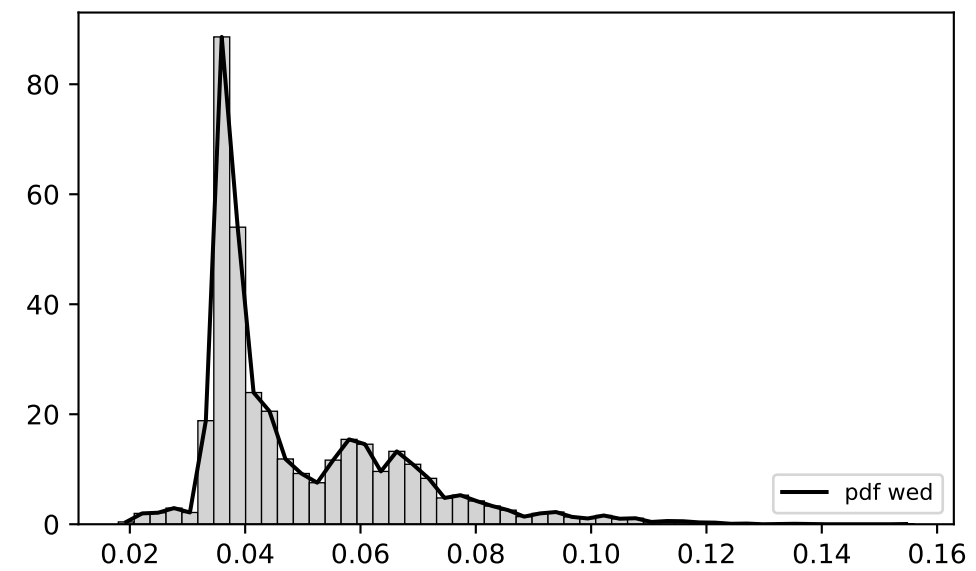

thu

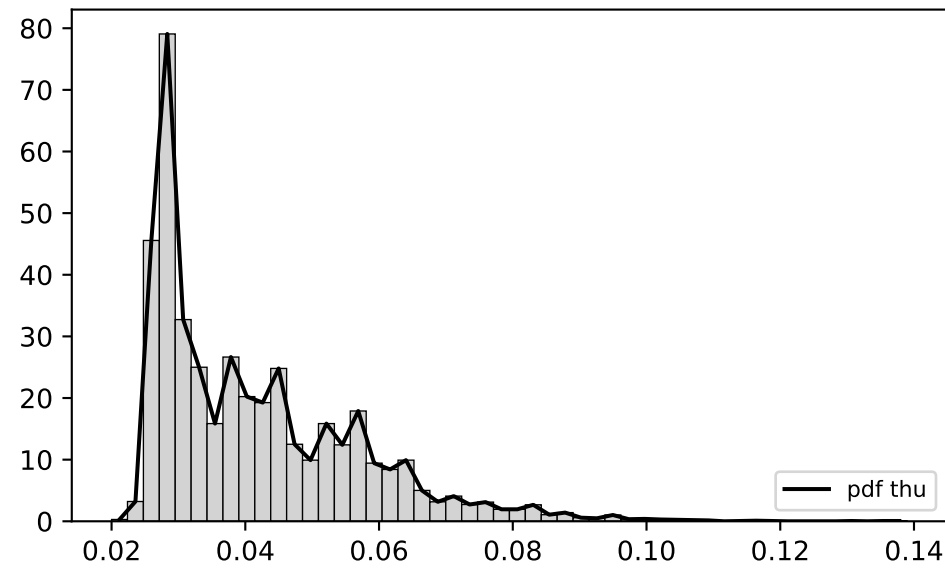

fri

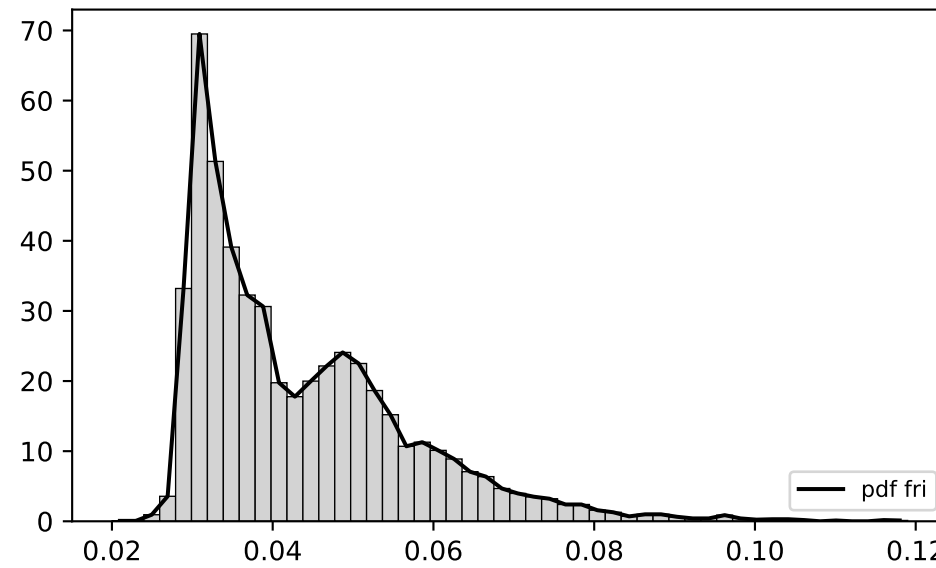

sat

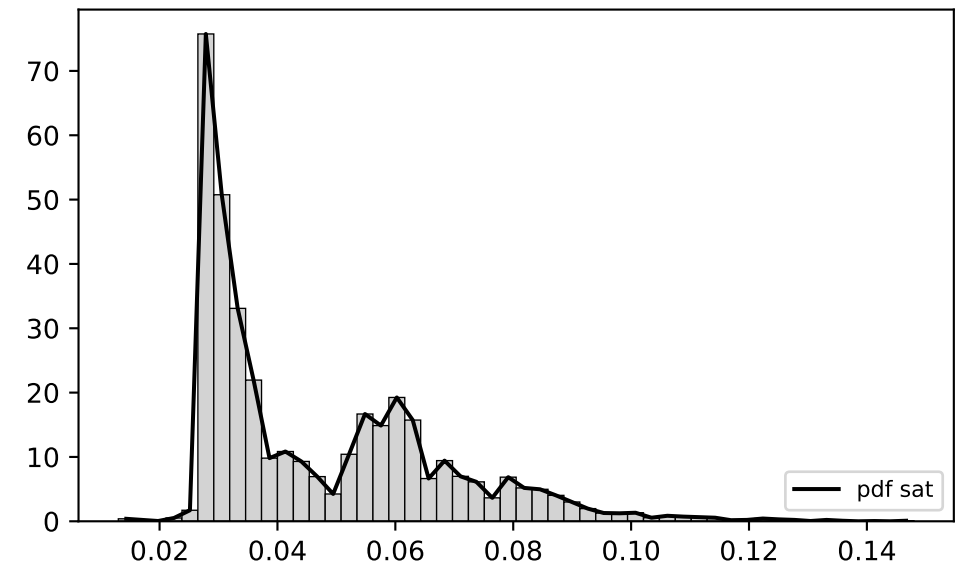

sun

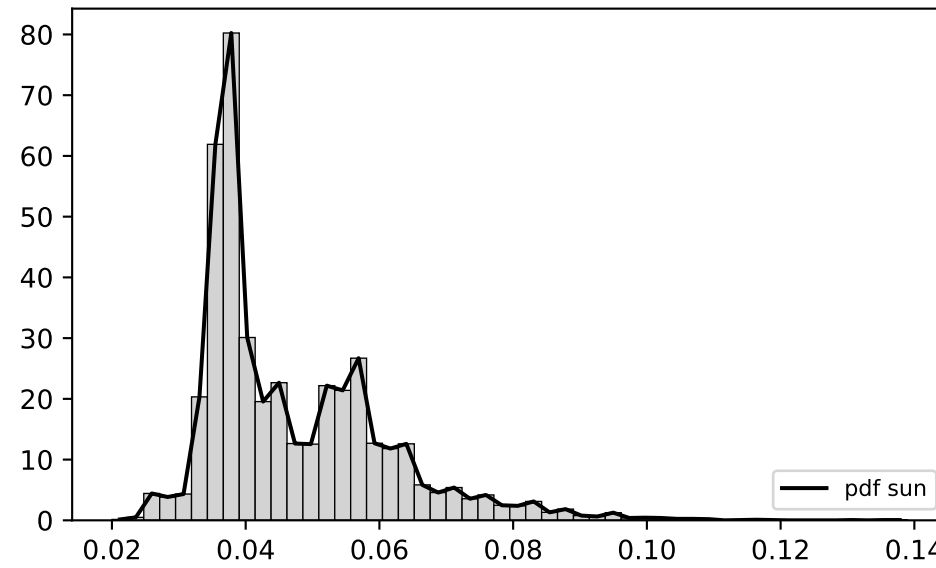

Supplement: Supplementary_material_for_Radiation_Protection_Dosimetry_Manuscript_2019_ncz154 [file supplementary_material_for_radiation_protection_dosimetry_manuscript_2019_ncz154.zip › Supplementary material for Radiation Protection Dosimetry Manuscript 2019/Location1_Figures_3rdWeek/Figure2_UMTS_3rdWeek.pdf]

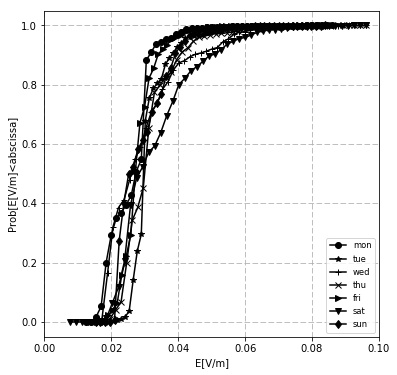

Supplement: Supplementary_material_for_Radiation_Protection_Dosimetry_Manuscript_2019_ncz154 [file supplementary_material_for_radiation_protection_dosimetry_manuscript_2019_ncz154.zip › Supplementary material for Radiation Protection Dosimetry Manuscript 2019/Location1_Figures_3rdWeek/Figure3_DCS_3rdWeek.jpg]

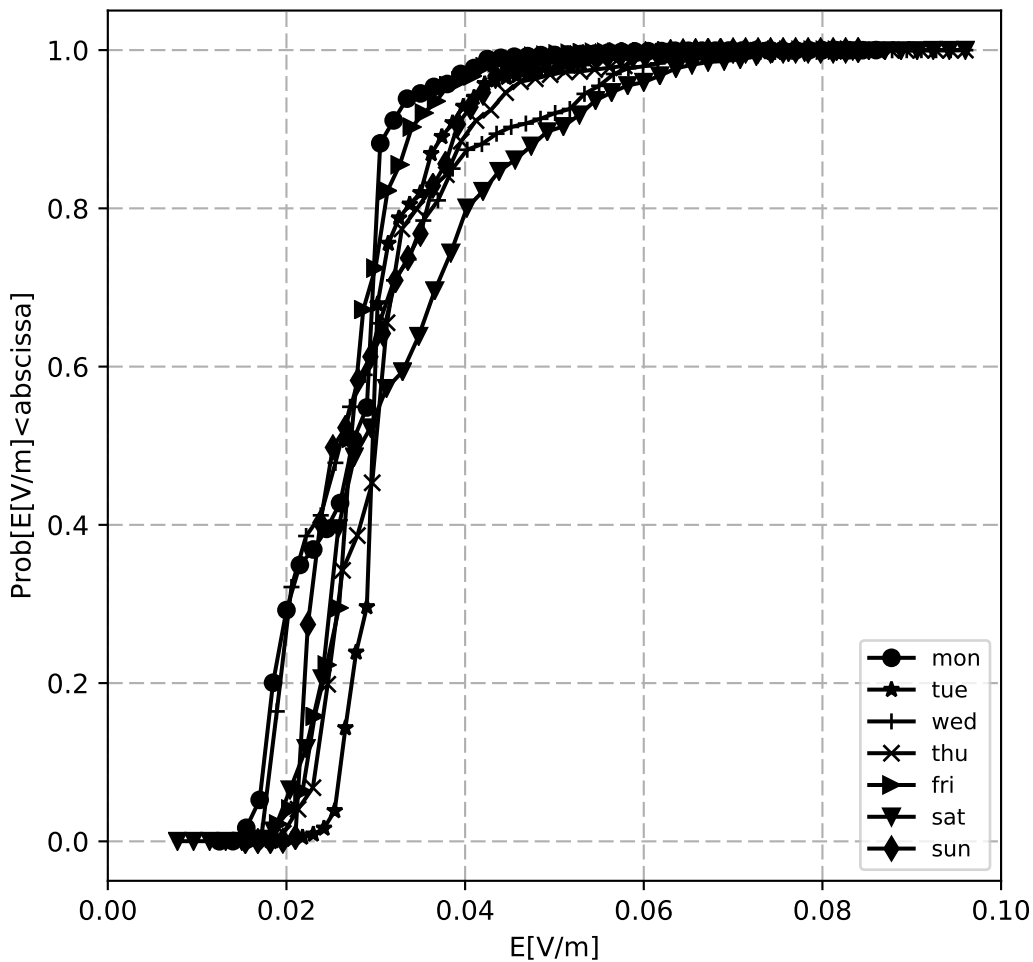

Supplement: Supplementary_material_for_Radiation_Protection_Dosimetry_Manuscript_2019_ncz154 [file supplementary_material_for_radiation_protection_dosimetry_manuscript_2019_ncz154.zip › Supplementary material for Radiation Protection Dosimetry Manuscript 2019/Location1_Figures_3rdWeek/Figure3_DCS_3rdWeek.pdf]

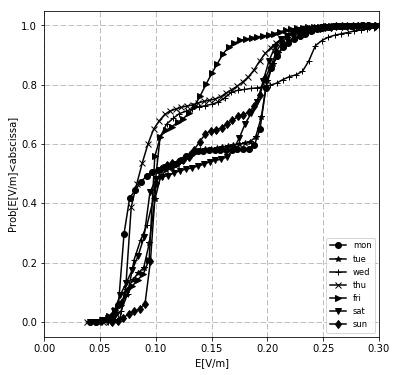

Supplement: Supplementary_material_for_Radiation_Protection_Dosimetry_Manuscript_2019_ncz154 [file supplementary_material_for_radiation_protection_dosimetry_manuscript_2019_ncz154.zip › Supplementary material for Radiation Protection Dosimetry Manuscript 2019/Location1_Figures_3rdWeek/Figure3_GSM_3rdWeek.jpg]

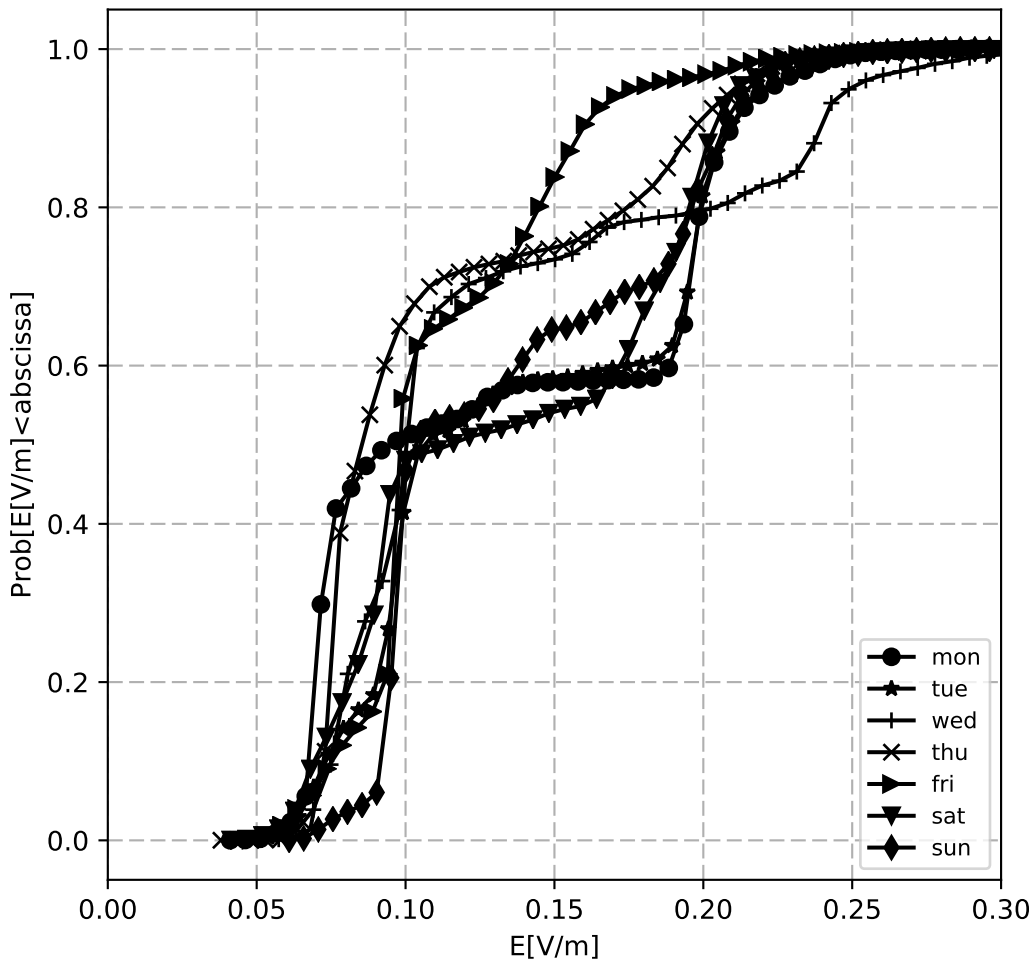

Supplement: Supplementary_material_for_Radiation_Protection_Dosimetry_Manuscript_2019_ncz154 [file supplementary_material_for_radiation_protection_dosimetry_manuscript_2019_ncz154.zip › Supplementary material for Radiation Protection Dosimetry Manuscript 2019/Location1_Figures_3rdWeek/Figure3_GSM_3rdWeek.pdf]

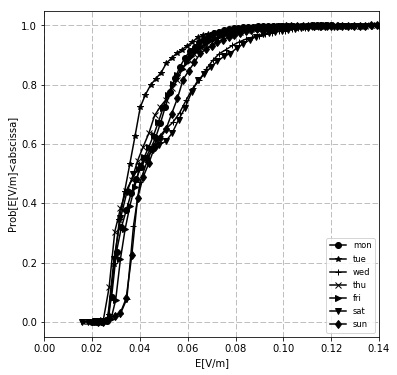

Supplement: Supplementary_material_for_Radiation_Protection_Dosimetry_Manuscript_2019_ncz154 [file supplementary_material_for_radiation_protection_dosimetry_manuscript_2019_ncz154.zip › Supplementary material for Radiation Protection Dosimetry Manuscript 2019/Location1_Figures_3rdWeek/Figure3_UMTS_3rdWeek.jpg]

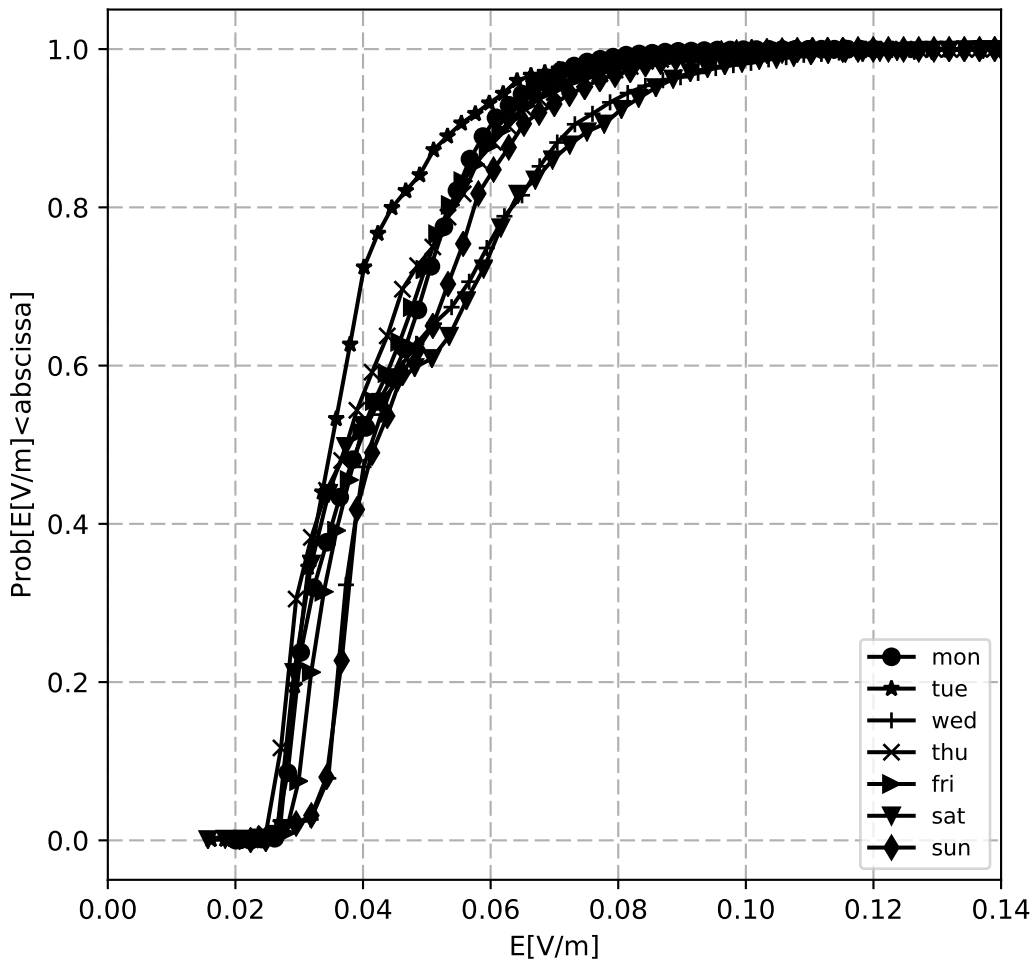

Supplement: Supplementary_material_for_Radiation_Protection_Dosimetry_Manuscript_2019_ncz154 [file supplementary_material_for_radiation_protection_dosimetry_manuscript_2019_ncz154.zip › Supplementary material for Radiation Protection Dosimetry Manuscript 2019/Location1_Figures_3rdWeek/Figure3_UMTS_3rdWeek.pdf]

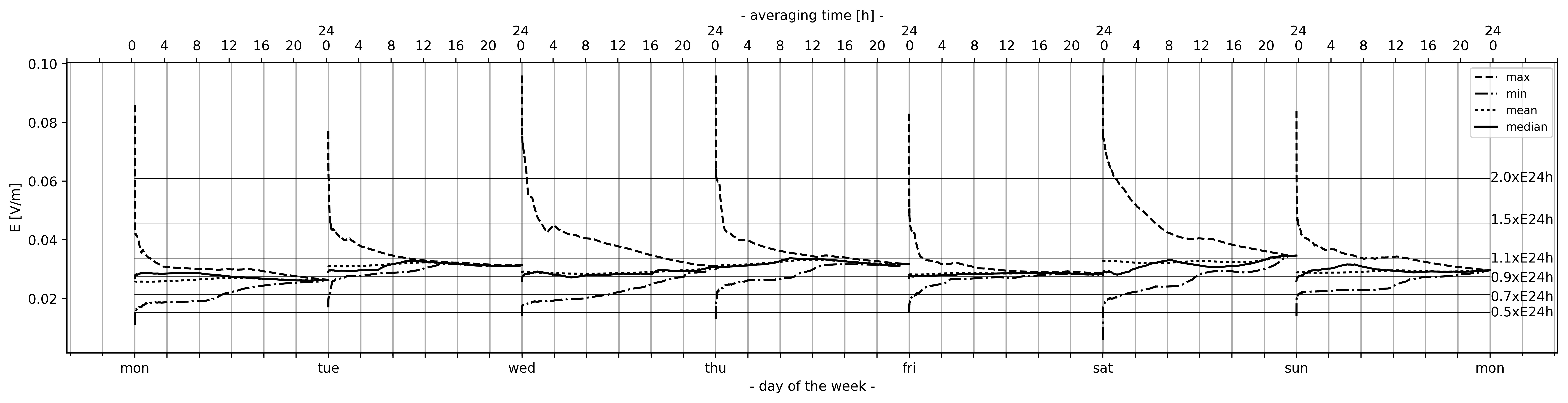

Supplement: Supplementary_material_for_Radiation_Protection_Dosimetry_Manuscript_2019_ncz154 [file supplementary_material_for_radiation_protection_dosimetry_manuscript_2019_ncz154.zip › Supplementary material for Radiation Protection Dosimetry Manuscript 2019/Location1_Figures_3rdWeek/Figure4_DCS_3rdWeek.jpg]

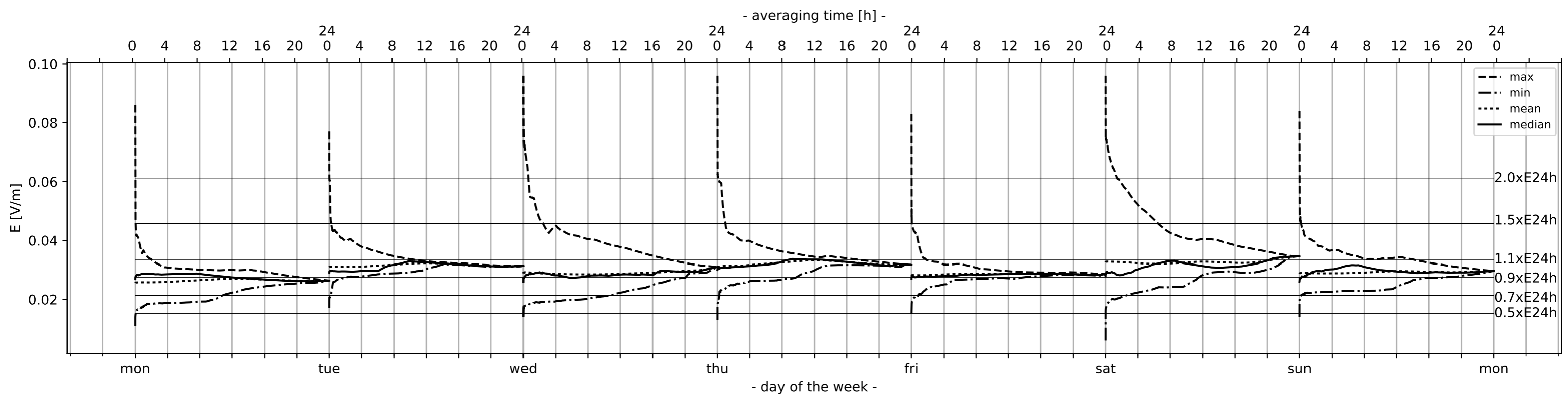

Supplement: Supplementary_material_for_Radiation_Protection_Dosimetry_Manuscript_2019_ncz154 [file supplementary_material_for_radiation_protection_dosimetry_manuscript_2019_ncz154.zip › Supplementary material for Radiation Protection Dosimetry Manuscript 2019/Location1_Figures_3rdWeek/Figure4_DCS_3rdWeek.pdf]

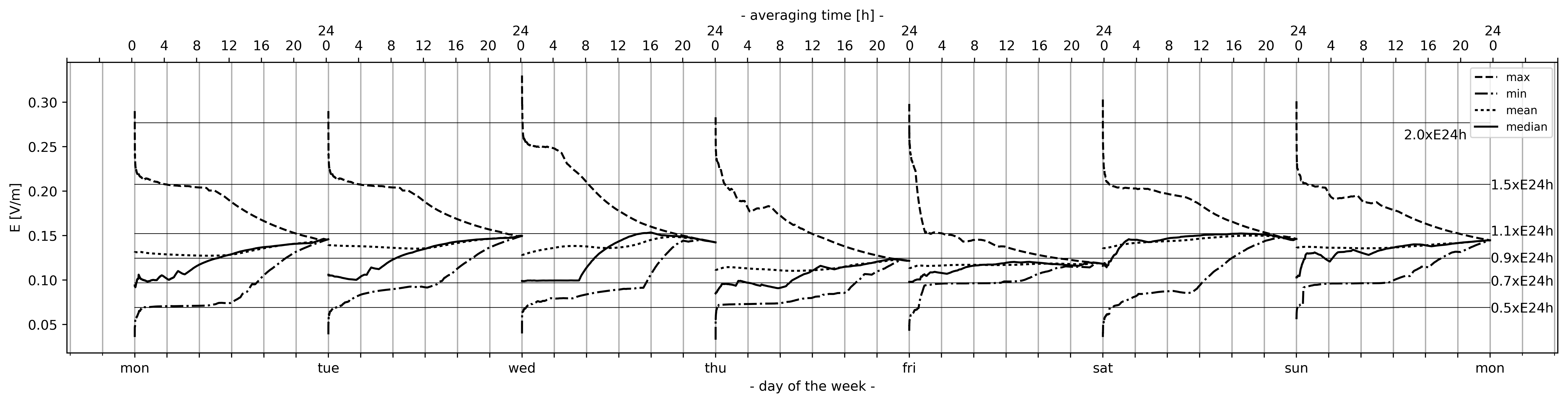

Supplement: Supplementary_material_for_Radiation_Protection_Dosimetry_Manuscript_2019_ncz154 [file supplementary_material_for_radiation_protection_dosimetry_manuscript_2019_ncz154.zip › Supplementary material for Radiation Protection Dosimetry Manuscript 2019/Location1_Figures_3rdWeek/Figure4_GSM_3rdWeek.jpg]

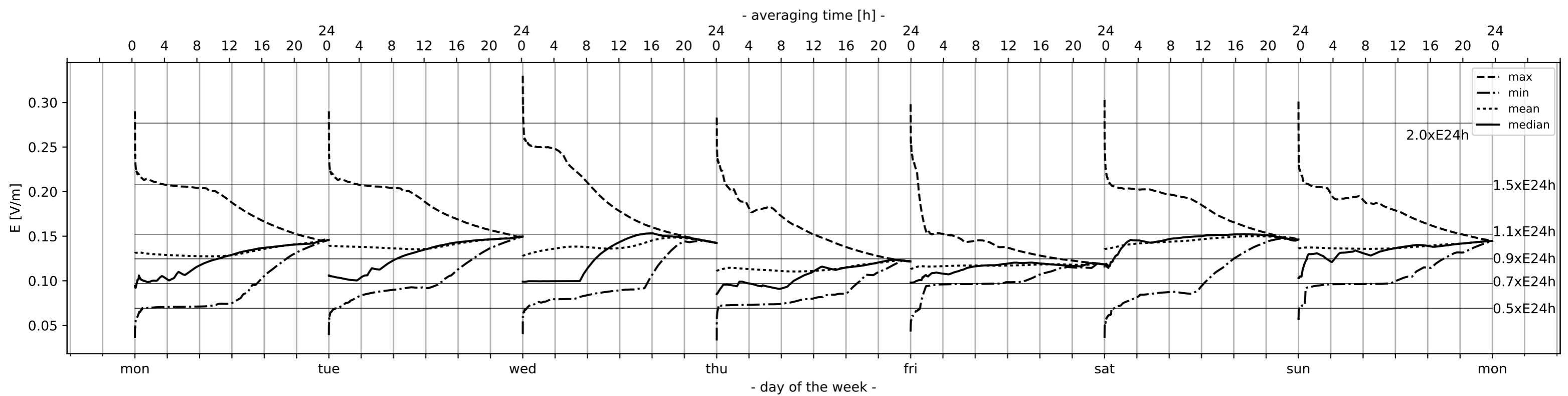

Supplement: Supplementary_material_for_Radiation_Protection_Dosimetry_Manuscript_2019_ncz154 [file supplementary_material_for_radiation_protection_dosimetry_manuscript_2019_ncz154.zip › Supplementary material for Radiation Protection Dosimetry Manuscript 2019/Location1_Figures_3rdWeek/Figure4_GSM_3rdWeek.pdf]

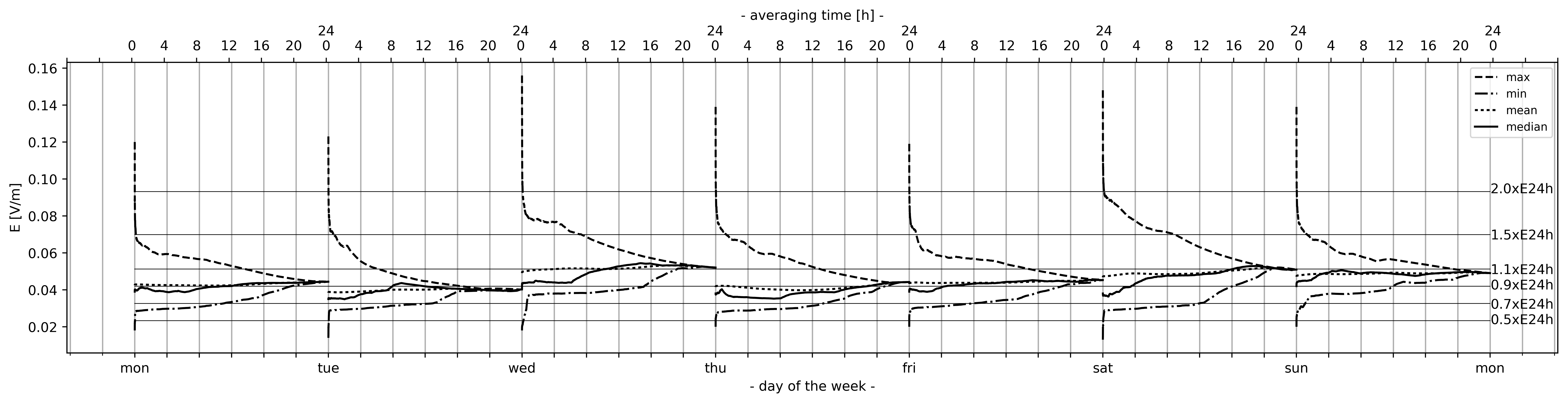

Supplement: Supplementary_material_for_Radiation_Protection_Dosimetry_Manuscript_2019_ncz154 [file supplementary_material_for_radiation_protection_dosimetry_manuscript_2019_ncz154.zip › Supplementary material for Radiation Protection Dosimetry Manuscript 2019/Location1_Figures_3rdWeek/Figure4_UMTS_3rdWeek.jpg]

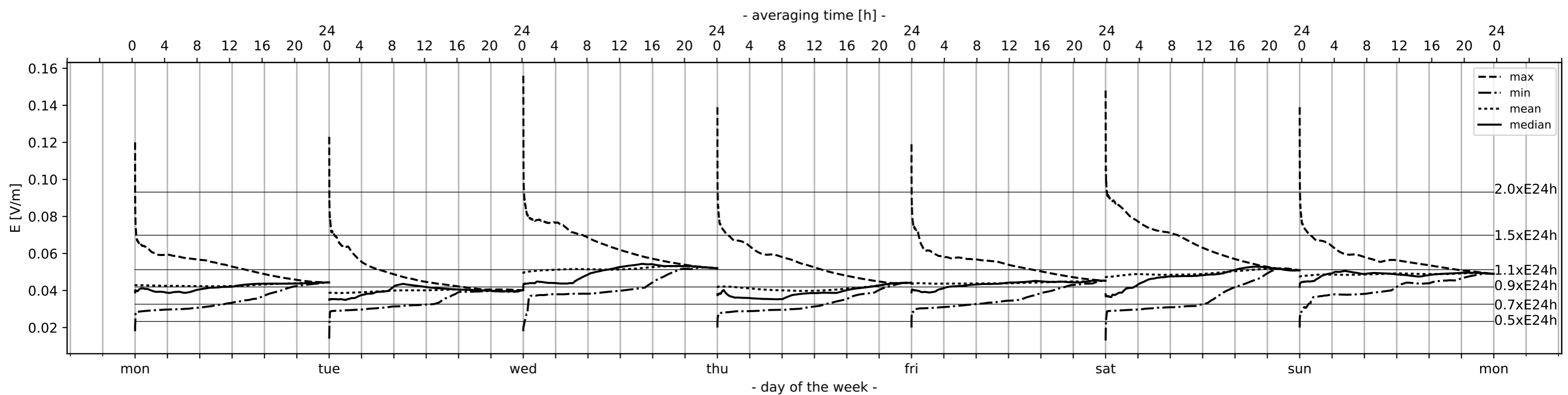

Supplement: Supplementary_material_for_Radiation_Protection_Dosimetry_Manuscript_2019_ncz154 [file supplementary_material_for_radiation_protection_dosimetry_manuscript_2019_ncz154.zip › Supplementary material for Radiation Protection Dosimetry Manuscript 2019/Location1_Figures_3rdWeek/Figure4_UMTS_3rdWeek.pdf]

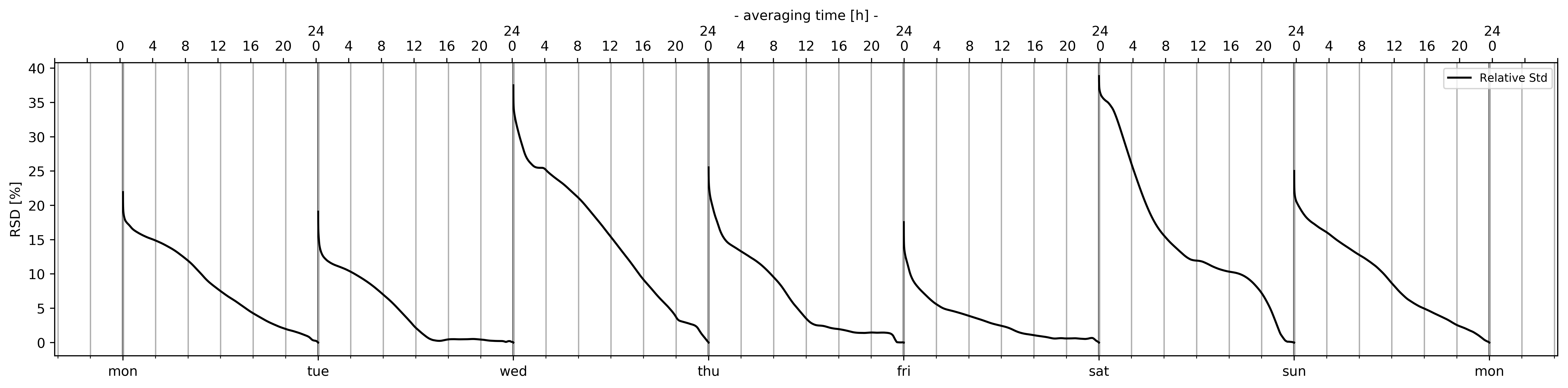

Supplement: Supplementary_material_for_Radiation_Protection_Dosimetry_Manuscript_2019_ncz154 [file supplementary_material_for_radiation_protection_dosimetry_manuscript_2019_ncz154.zip › Supplementary material for Radiation Protection Dosimetry Manuscript 2019/Location1_Figures_3rdWeek/Figure5_DCS_3rdWeek.jpg]

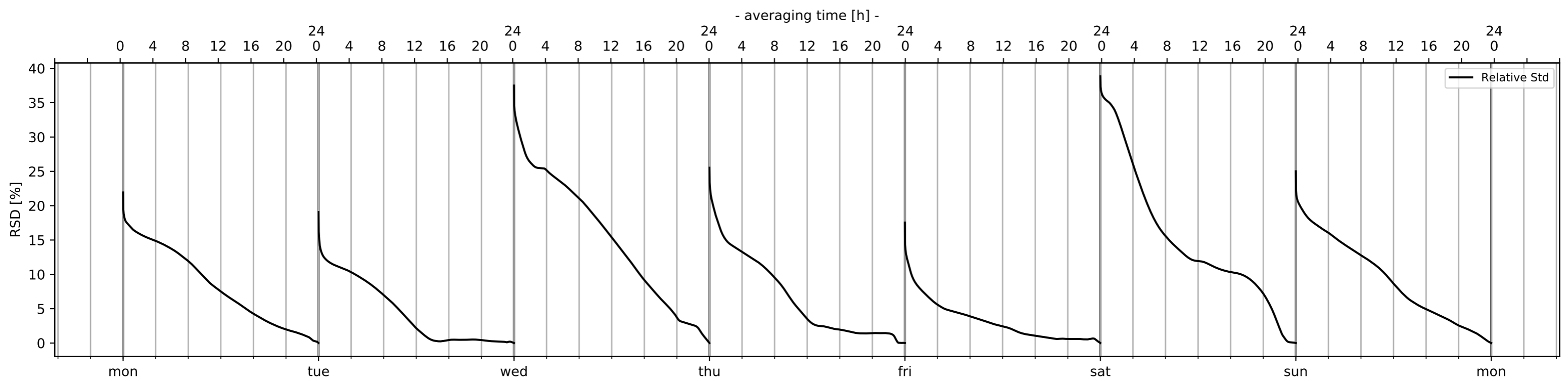

Supplement: Supplementary_material_for_Radiation_Protection_Dosimetry_Manuscript_2019_ncz154 [file supplementary_material_for_radiation_protection_dosimetry_manuscript_2019_ncz154.zip › Supplementary material for Radiation Protection Dosimetry Manuscript 2019/Location1_Figures_3rdWeek/Figure5_DCS_3rdWeek.pdf]

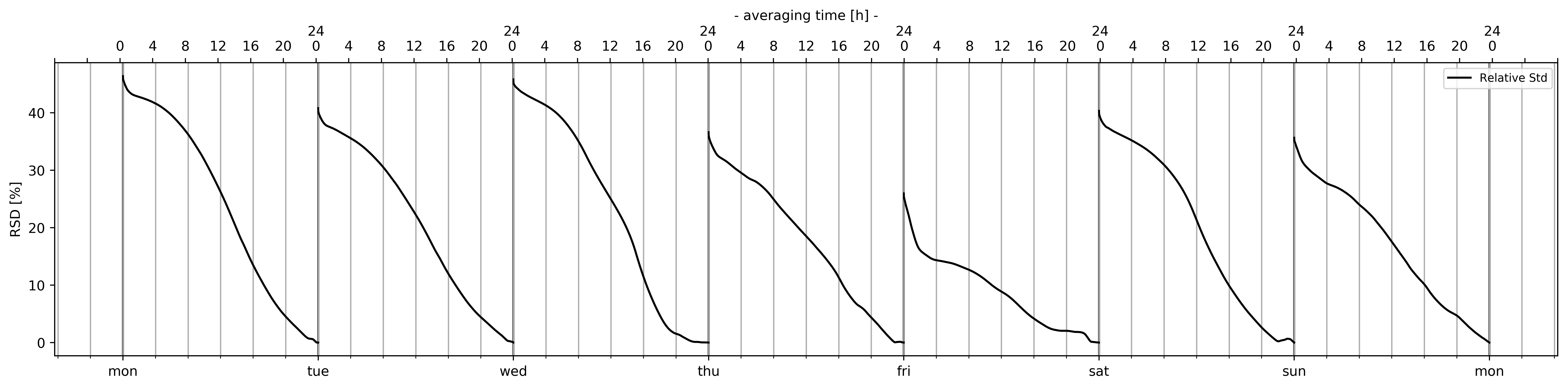

Supplement: Supplementary_material_for_Radiation_Protection_Dosimetry_Manuscript_2019_ncz154 [file supplementary_material_for_radiation_protection_dosimetry_manuscript_2019_ncz154.zip › Supplementary material for Radiation Protection Dosimetry Manuscript 2019/Location1_Figures_3rdWeek/Figure5_GSM_3rdWeek.jpg]

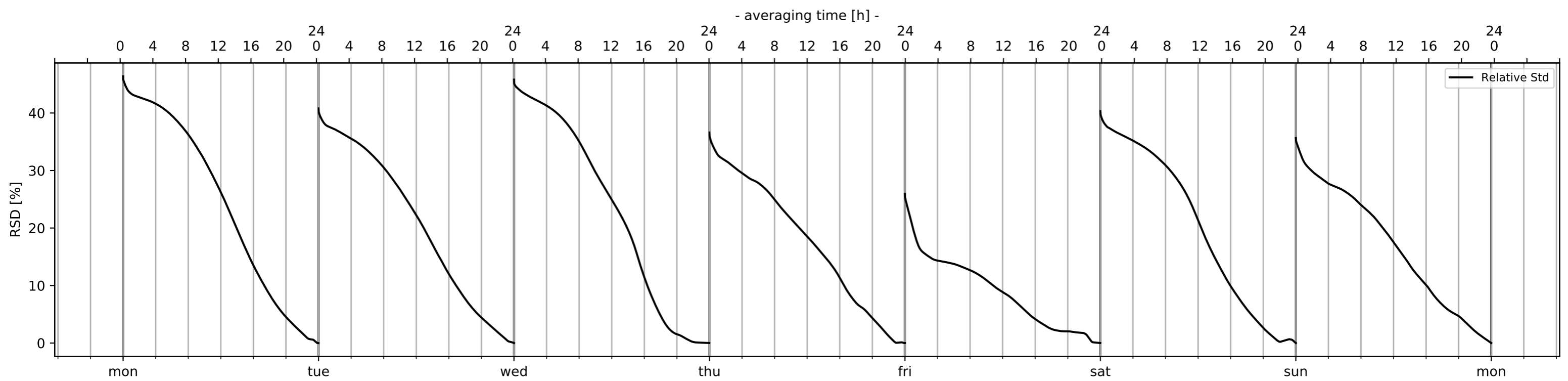

Supplement: Supplementary_material_for_Radiation_Protection_Dosimetry_Manuscript_2019_ncz154 [file supplementary_material_for_radiation_protection_dosimetry_manuscript_2019_ncz154.zip › Supplementary material for Radiation Protection Dosimetry Manuscript 2019/Location1_Figures_3rdWeek/Figure5_GSM_3rdWeek.pdf]

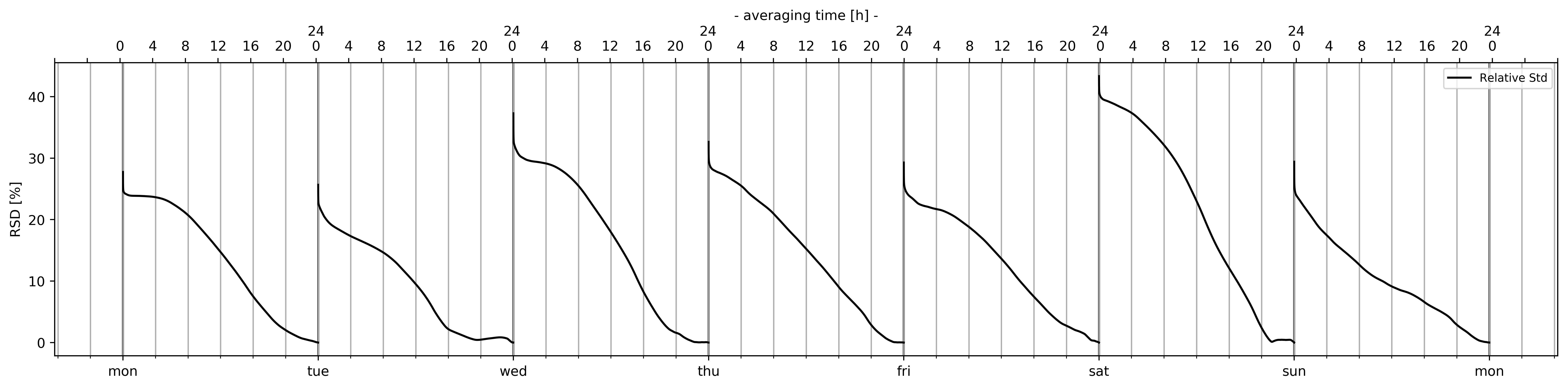

Supplement: Supplementary_material_for_Radiation_Protection_Dosimetry_Manuscript_2019_ncz154 [file supplementary_material_for_radiation_protection_dosimetry_manuscript_2019_ncz154.zip › Supplementary material for Radiation Protection Dosimetry Manuscript 2019/Location1_Figures_3rdWeek/Figure5_UMTS_3rdWeek.jpg]

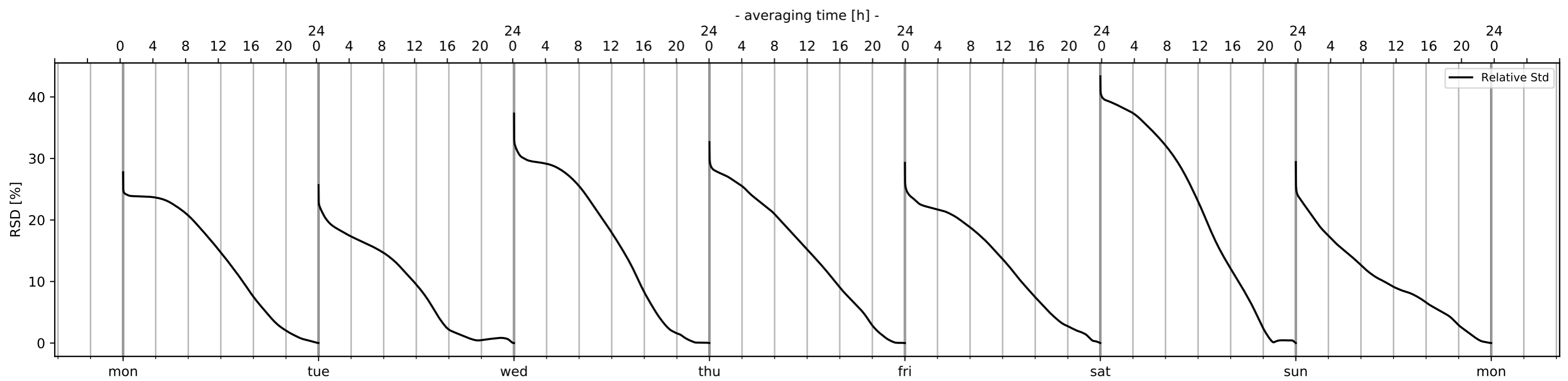

Supplement: Supplementary_material_for_Radiation_Protection_Dosimetry_Manuscript_2019_ncz154 [file supplementary_material_for_radiation_protection_dosimetry_manuscript_2019_ncz154.zip › Supplementary material for Radiation Protection Dosimetry Manuscript 2019/Location1_Figures_3rdWeek/Figure5_UMTS_3rdWeek.pdf]
